# Supplementary material for: Sensitive Detection of Cell-Free Tumour DNA Using Optimised Targeted Sequencing Can Predict Prognosis in Gastro-Oesophageal Cancer
Source: Cancers (Basel). 2023 Feb 11;15(4):1160. doi: 10.3390/cancers15041160 (PMC9954085; doi:10.3390/cancers15041160)

Supplementary figure S6

1D and 2D plots and concentration graphs from Quantasoft for ddPCR on 12 patients

ddPCR; digital droplet PCR, pre; diagnostic plasma sample, post; plasma sample from after surgery, NK; negative control (wild type), NTC; no template control, pos ctrl; positive control (from tissue DNA)

P28 Final- pre and post

Gene targets : TP53,p.R273H, c.818G>A and KRAS p.G13D, c.38G>A

Protocol : Exp 12

Assays: 0.7X TP53,p.R273H mut (FAM) and 1X dark probe for TP53,p.R273H

0.3X KRAS p.G13D mut (FAM) and 1X KRAS WT p.G13D (HEX)

Annealing temp: 60°C

0.7X TP53, p.R273H

0.3X KRAS p.G13D

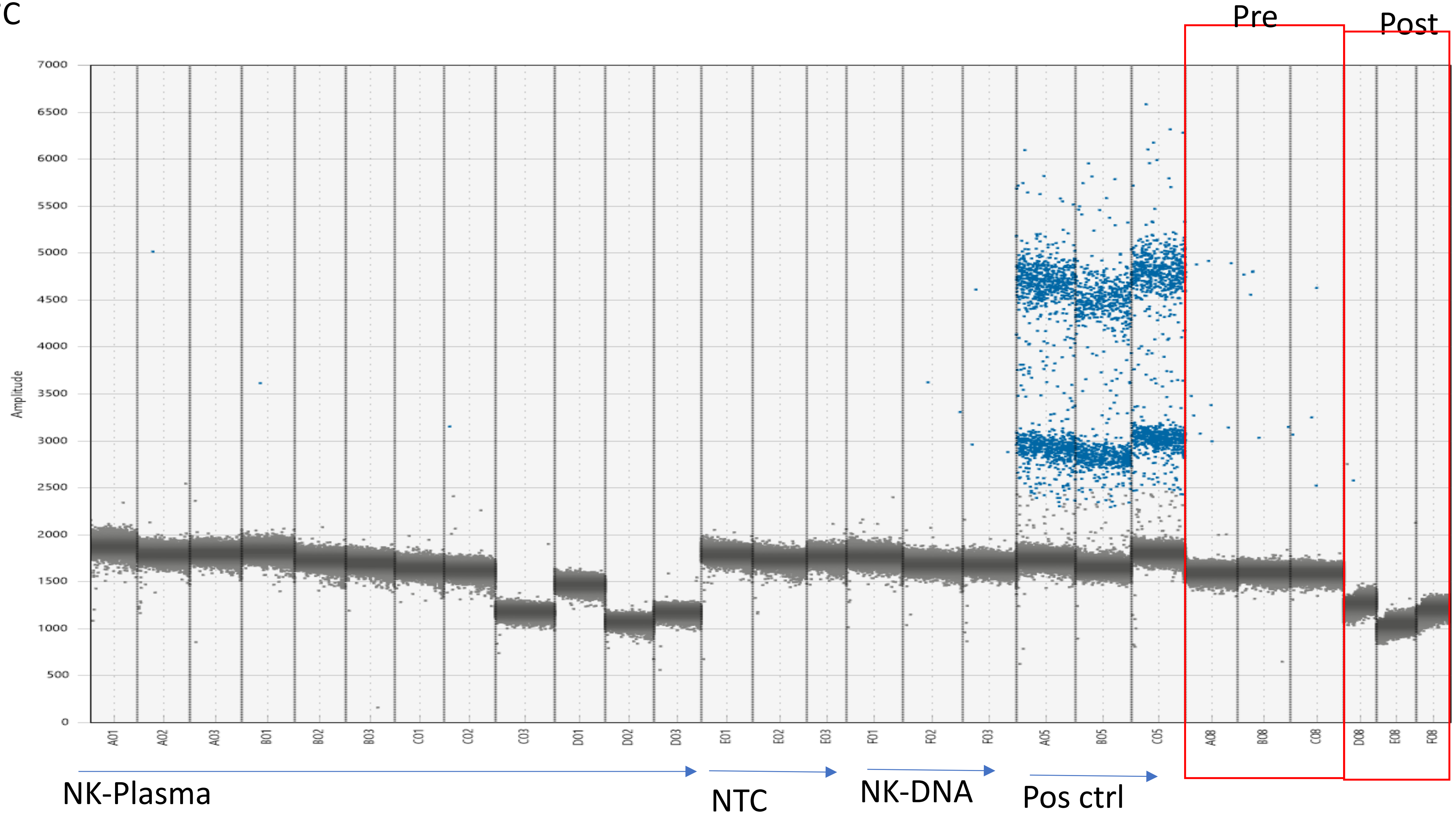

P28 Final- pre and post

Gene targets : TP53,p.R273H, c.818G>A and KRAS p.G13D, c.38G>A

Protocol : Exp 12

Assays: 1X dark probe for TP53,p.R273H and 1X KRAS WT p.G13D (HEX)

Annealing temp: 60°C

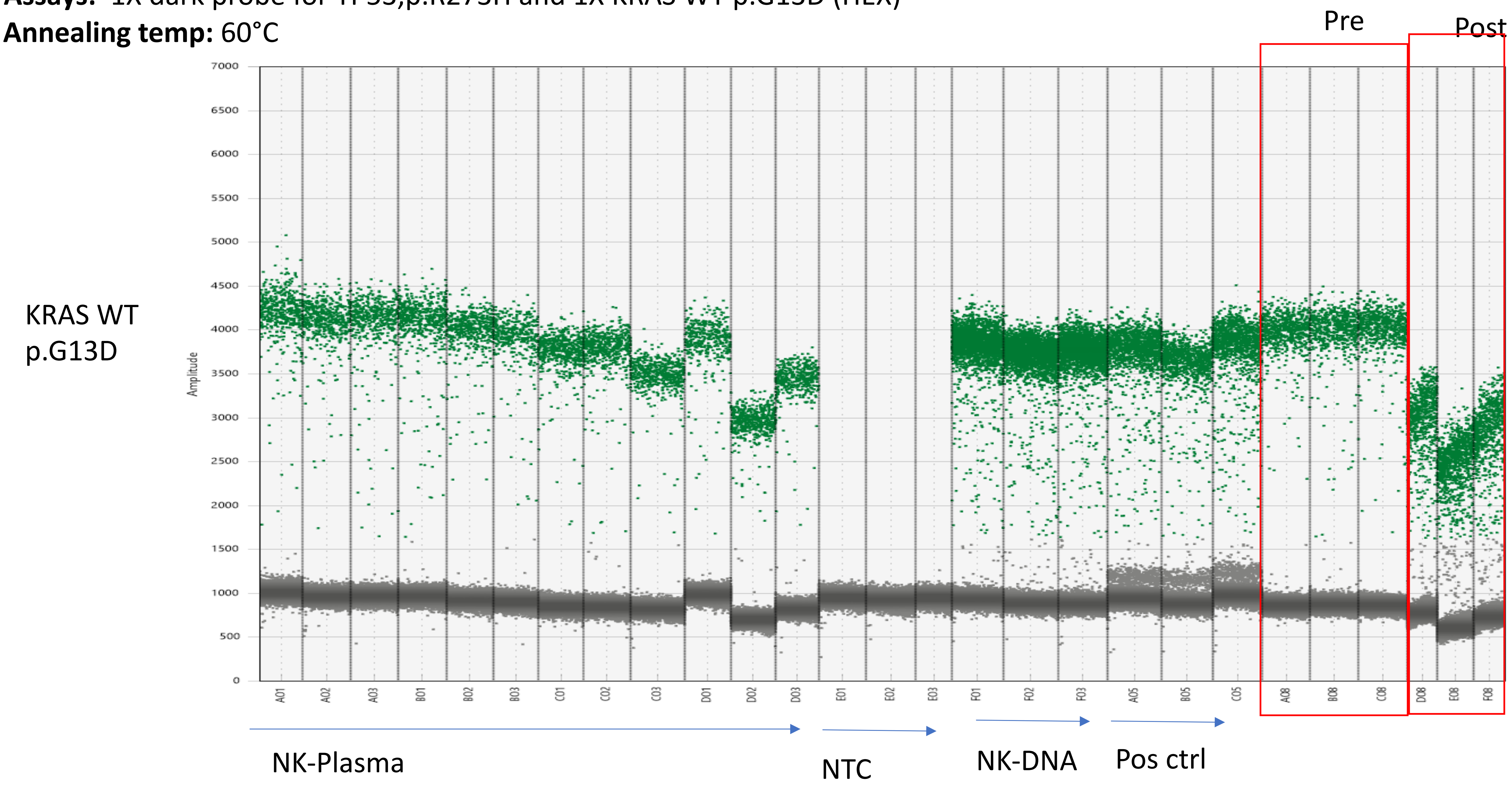

P28 Final- fp1 and fp2

Gene targets : TP53,p.R273H, c.818G>A and KRAS p.G13D, c.38G>A

Assays: 0.7X TP53,p.R273H mut (FAM) and 1X dark probe for TP53,p.R273H
0.3X KRAS p.G13D mut (FAM) and 1X KRAS WT p.G13D (HEX)

Protocol : Exp 12

Annealing temp: 60°C

0.7X TP53, p.R273H

0.3X KRAS p.G13D

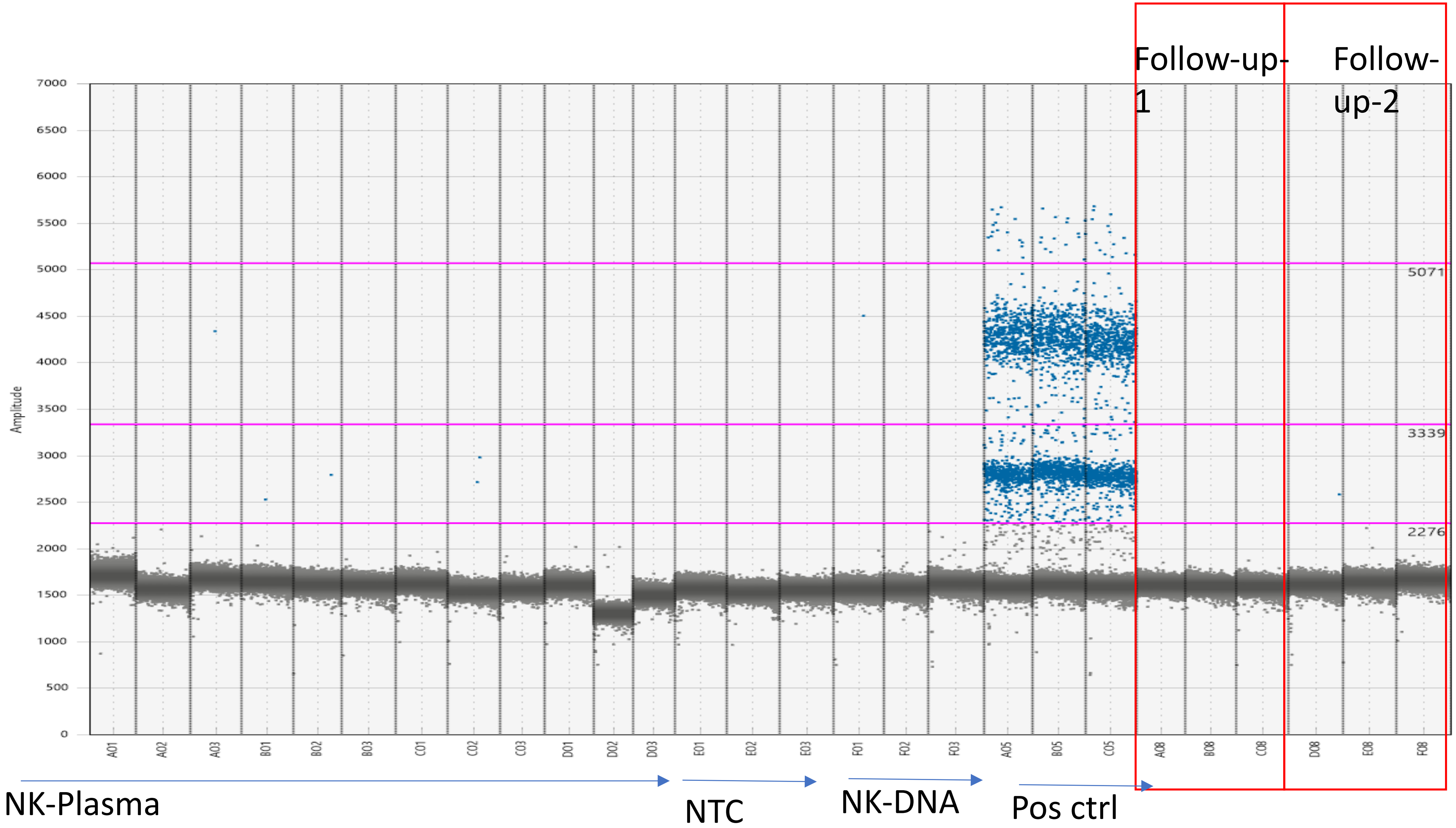

P28 Final- fp1 and fp2- WT

Gene targets : TP53,p.R273H, c.818G>A and KRAS p.G13D, c.38G>A

Assays: 1X dark probe for TP53,p.R273H and 1X KRAS WT p.G13D (HEX)

Protocol : Exp 12

Annealing temp: 60°C

KRAS WT p.G13D (HEX)

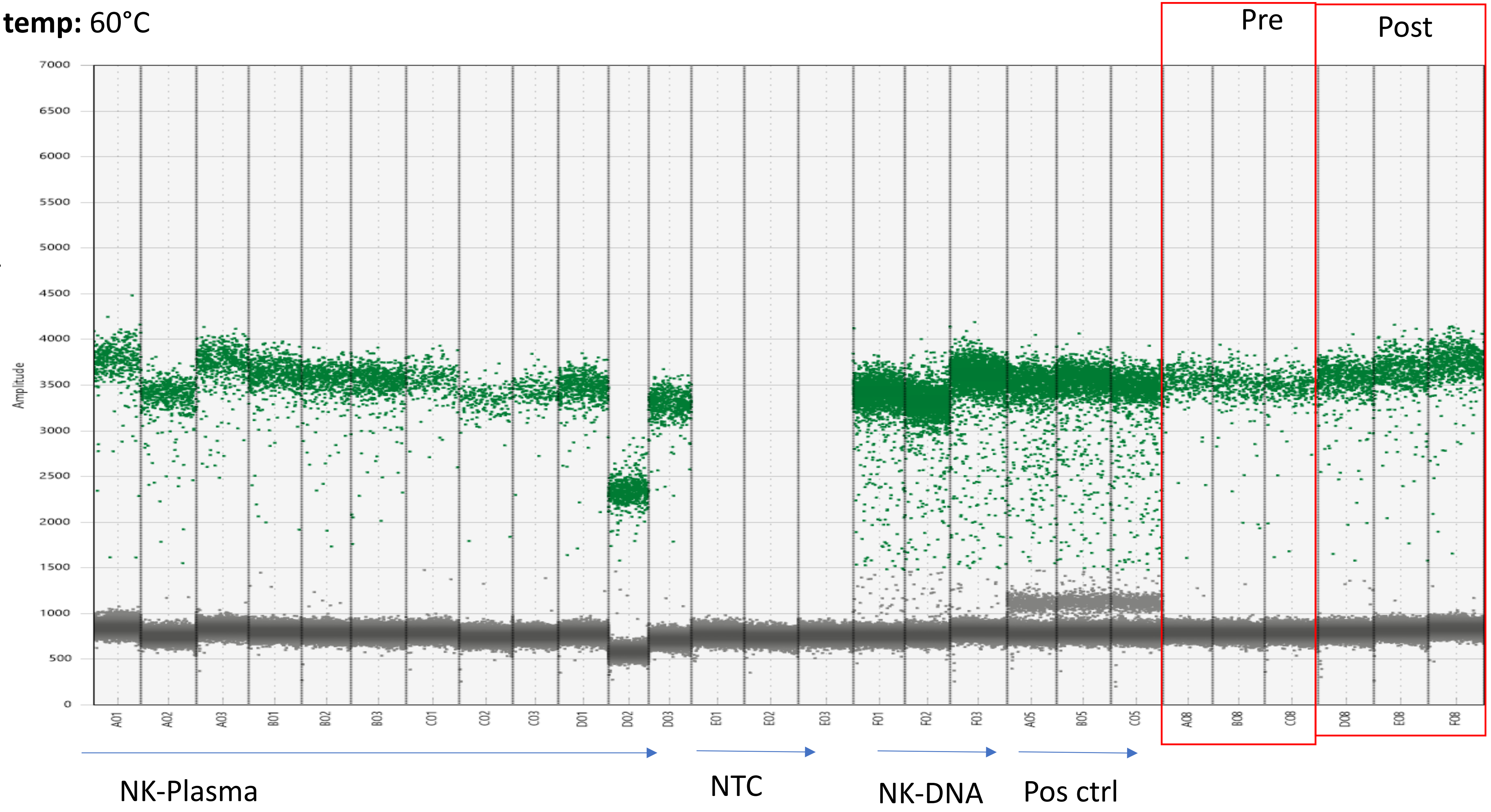

Positive – control

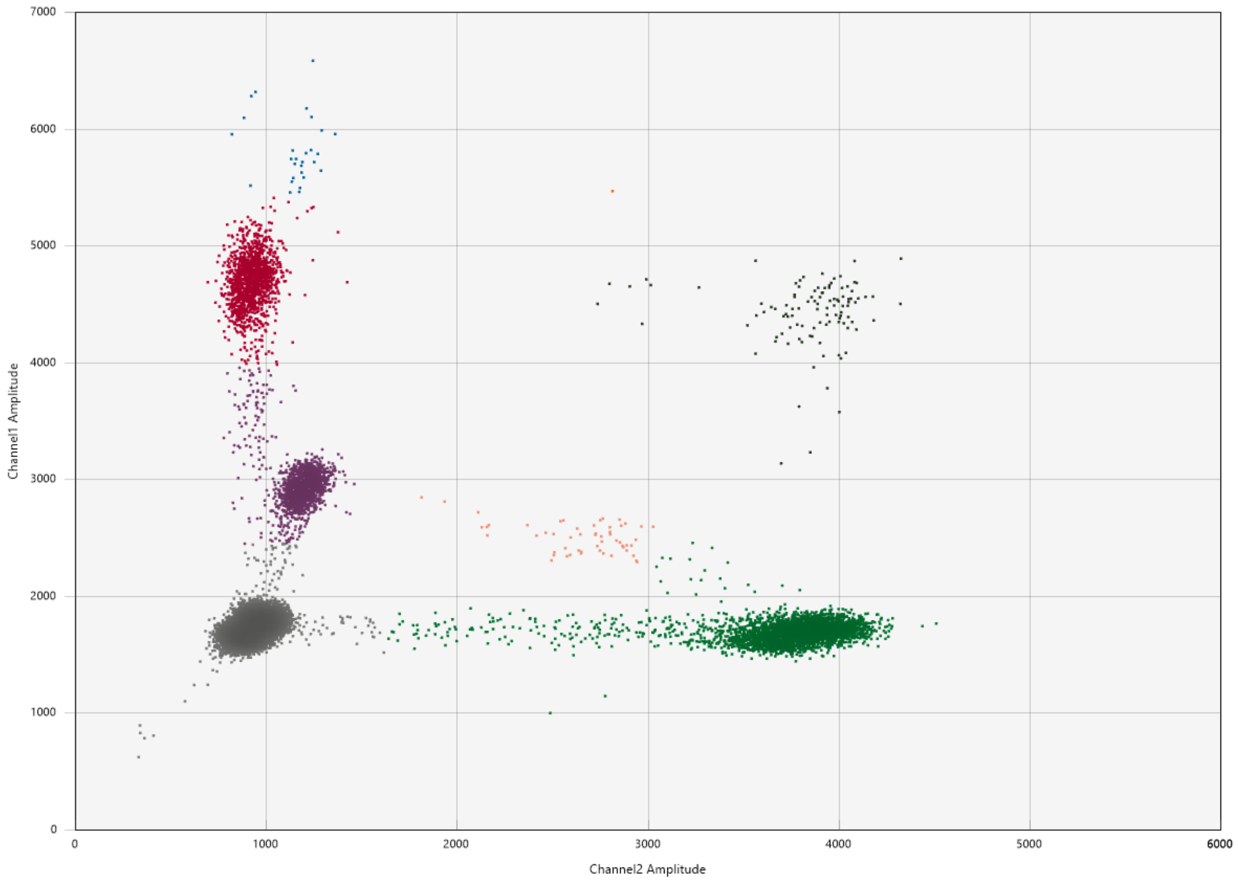

NK-Plasma

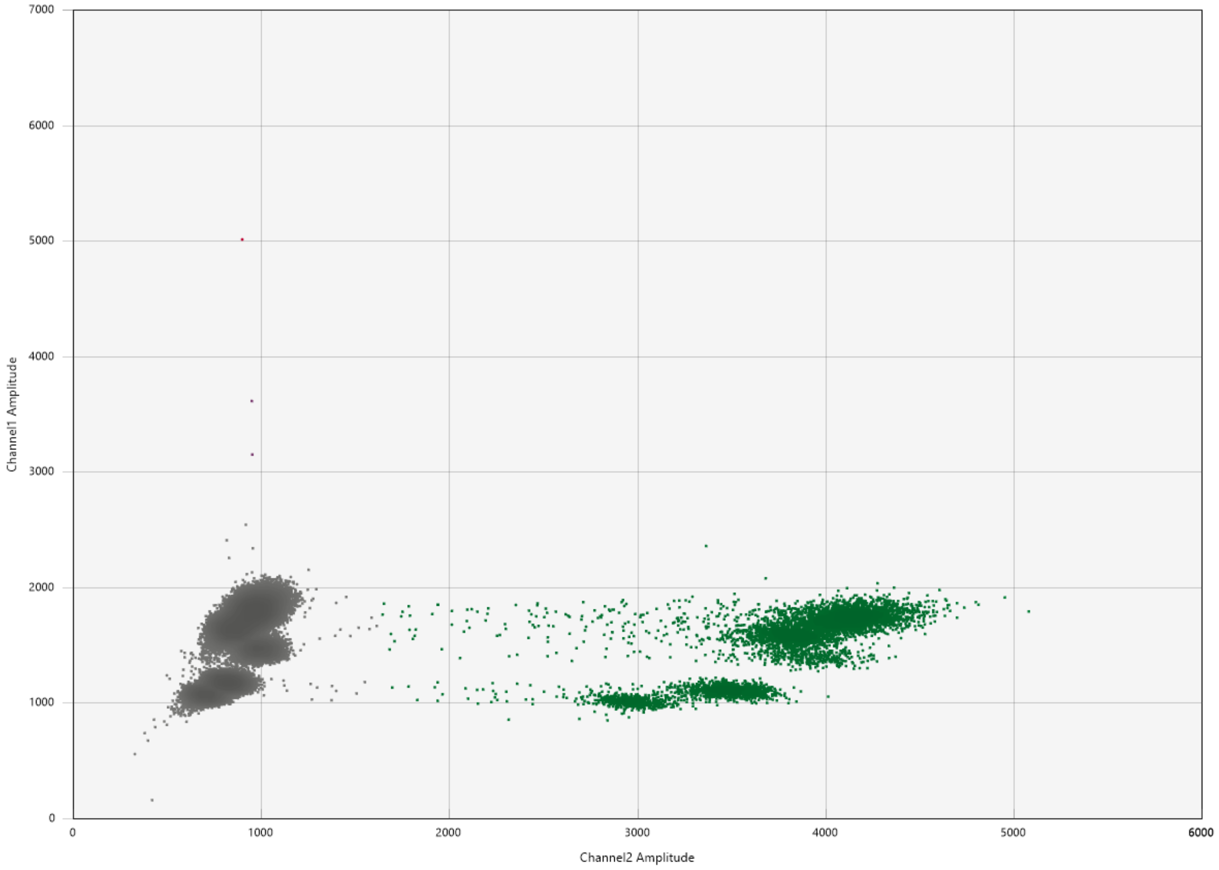

Pre-surgery

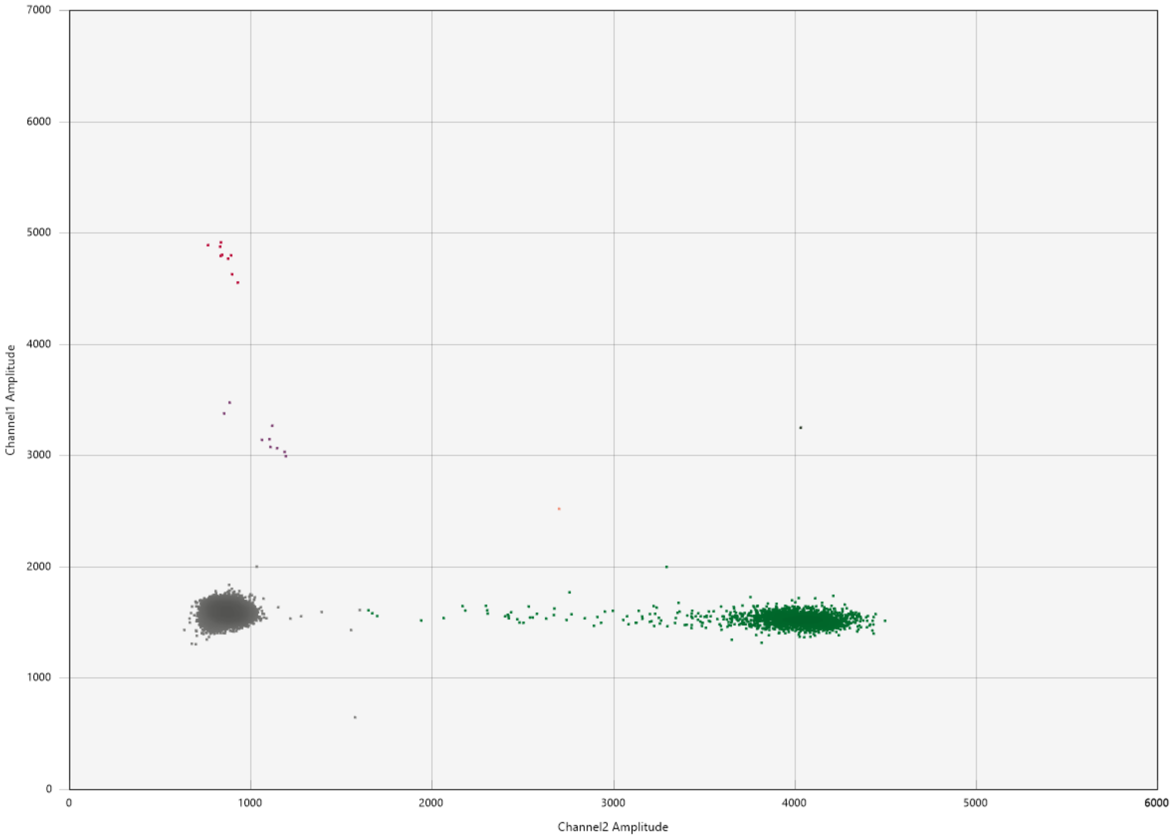

Post-surgery

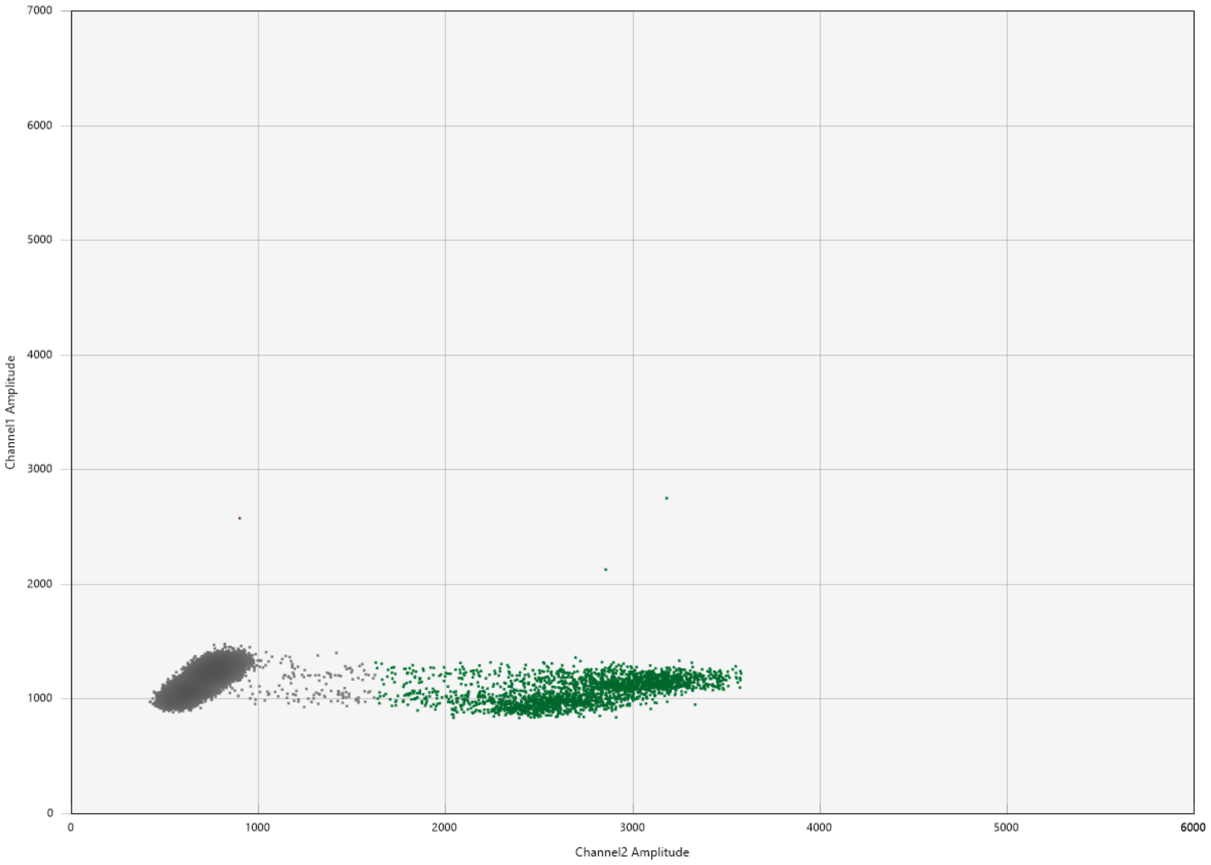

Follow-up 1

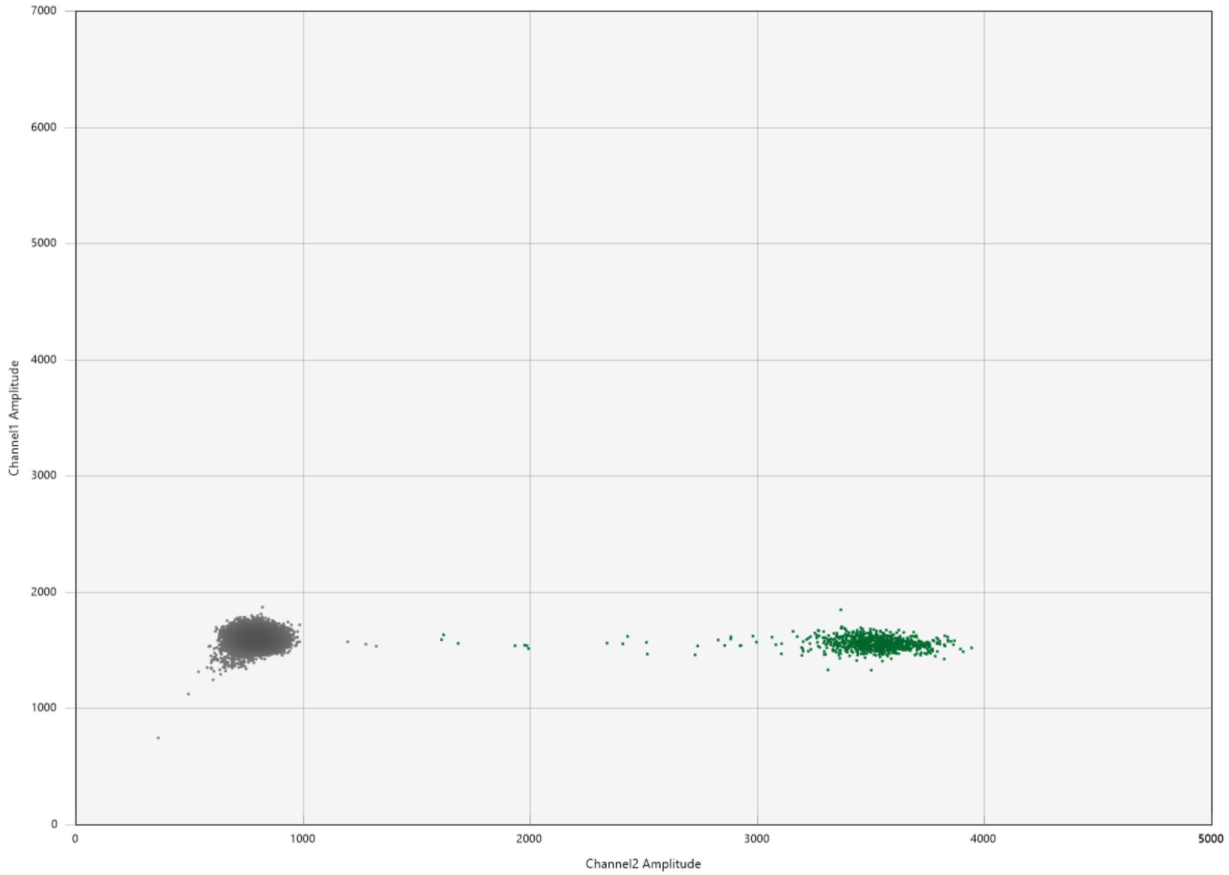

Follow-up 2

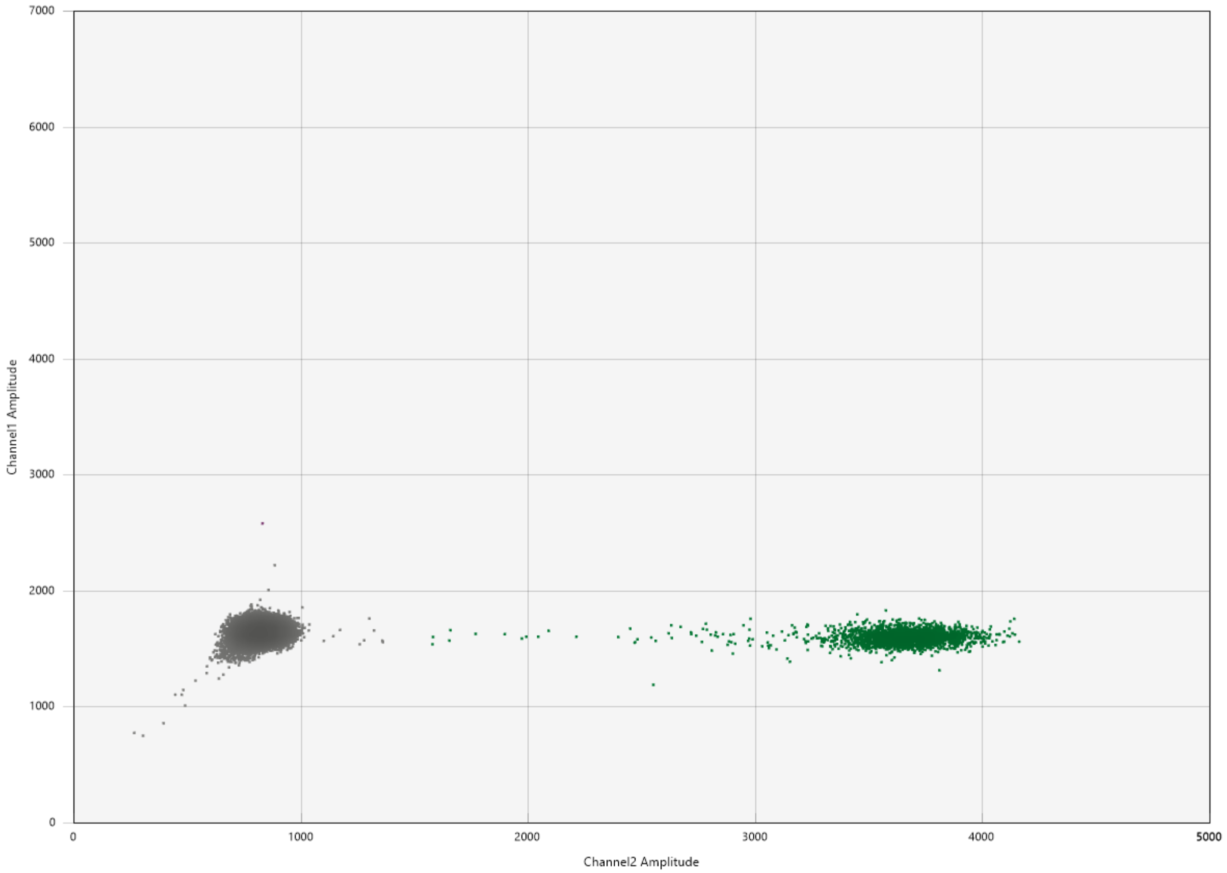

Concentration plots

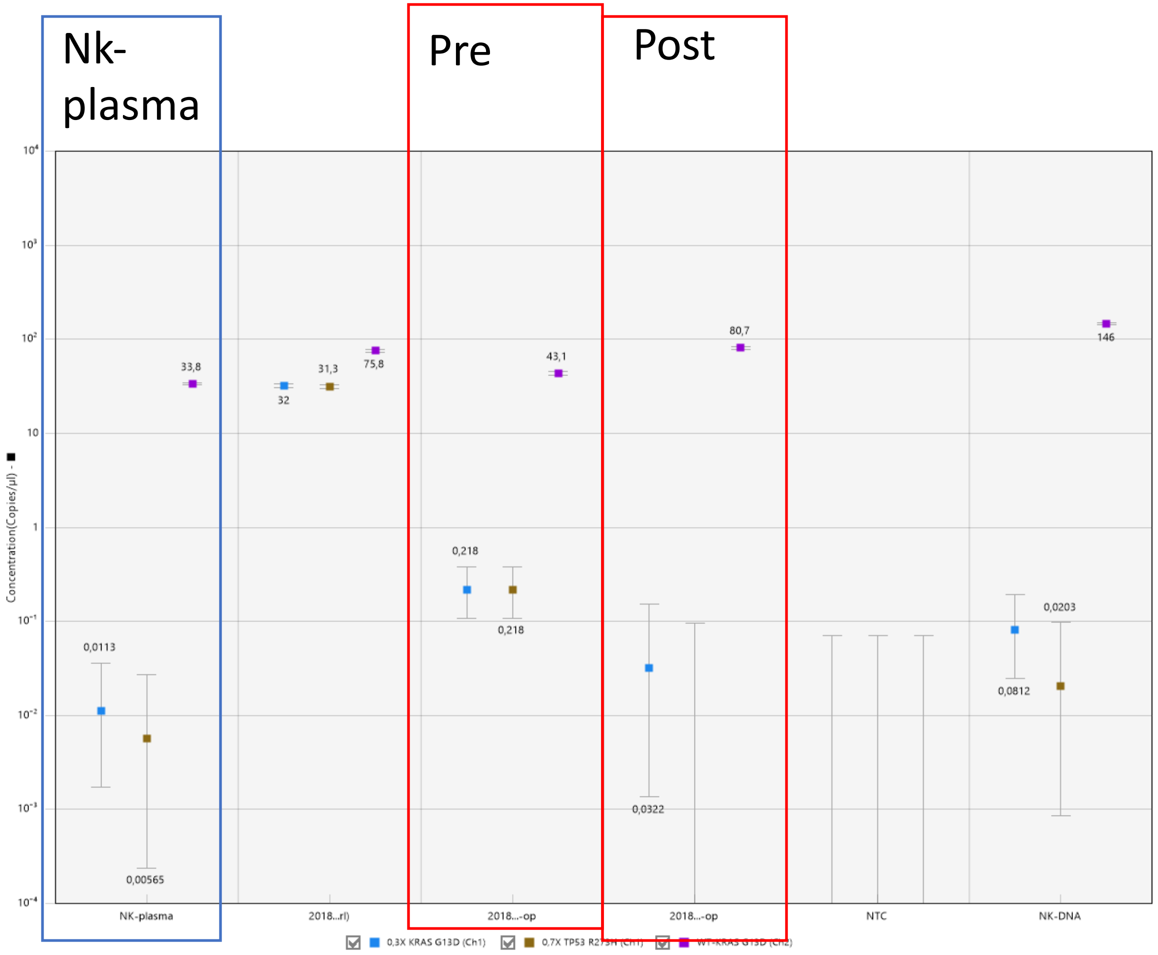

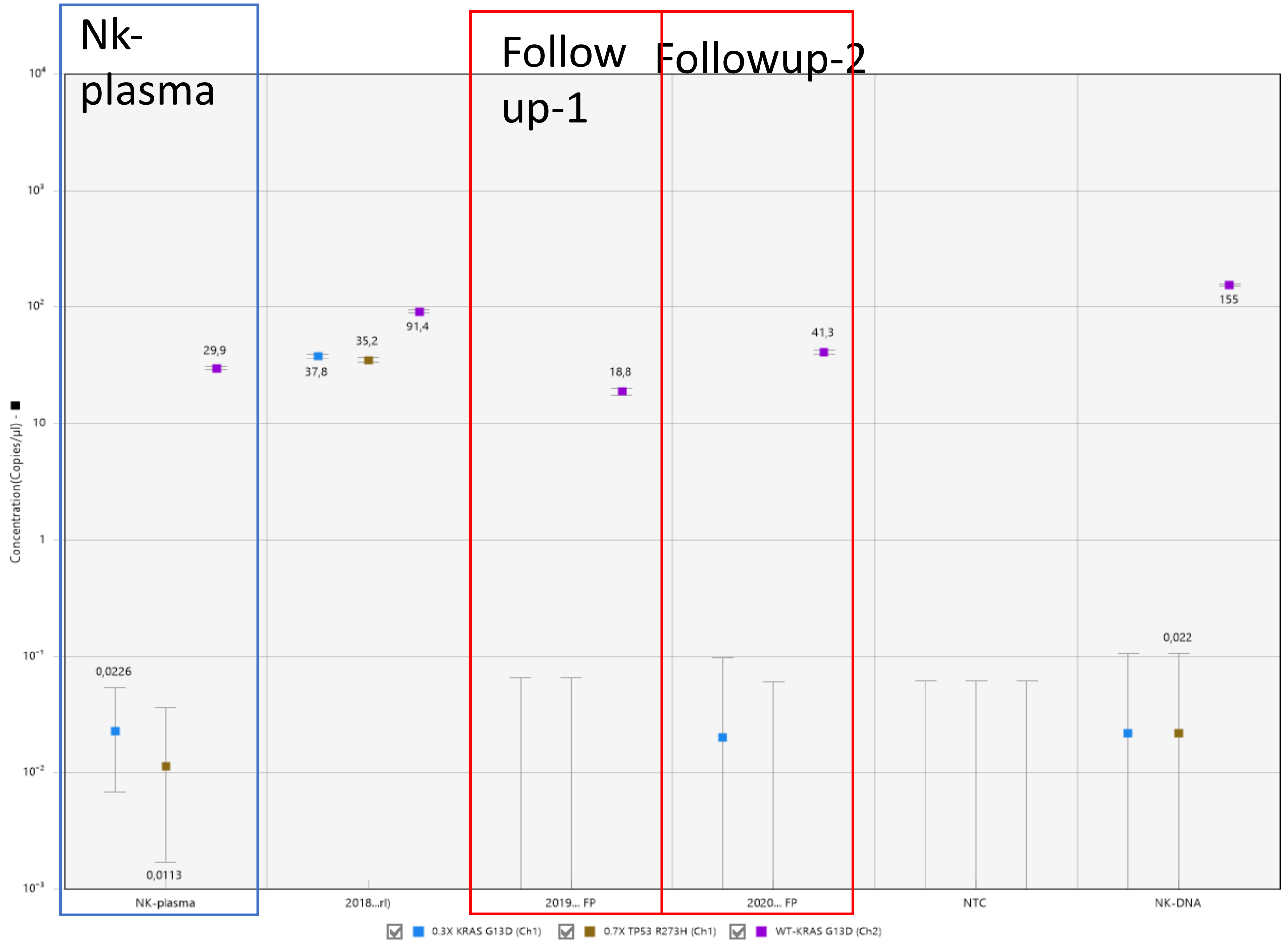

P27 - Final

Gene targets : PIK3CA, p.H1047R,c.3140A>G and TP53, p.R273C, c.817C>T

Annealing temp: 55°C

Protocol: Exp 21

Assays: 0.5X PIK3CA, p.H1047R mut (FAM) and 1X PIK3CA WT, p.H1047R (HEX)
1X TP53, p.R273C mut (FAM) and 1X dark probe for TP53, p.R273C

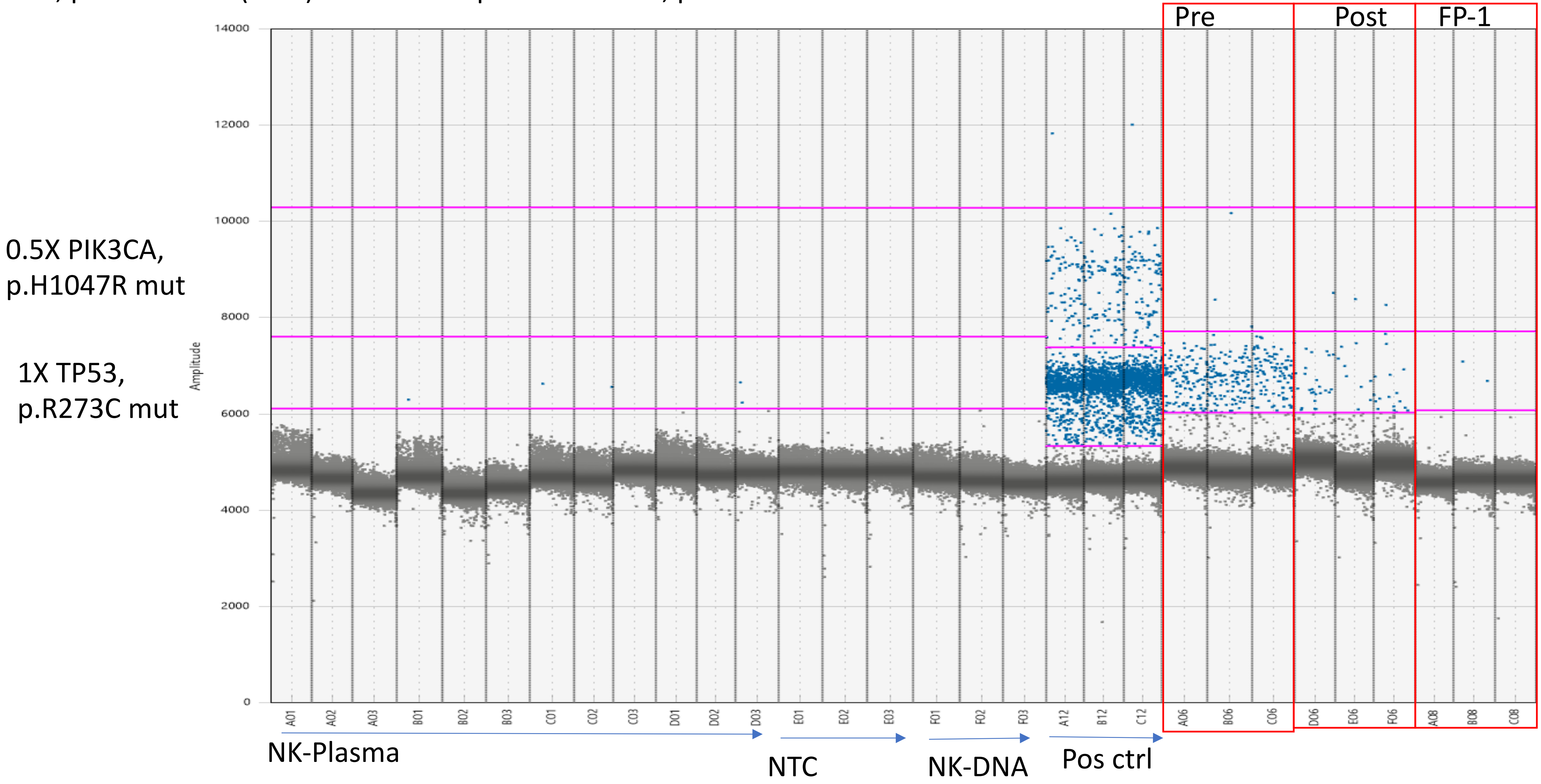

P27 - Final -WT

Gene targets : PIK3CA, p.H1047R,c.3140A>G and TP53, p.R273C, c.817C>T

Assays: 1X PIK3CA WT, p.H1047R (HEX) and 1X dark probe for TP53, p.R273C

Annealing temp: 55°C
Protocol: Exp 21

0.5X PIK3CA
WT,
p.H1047R

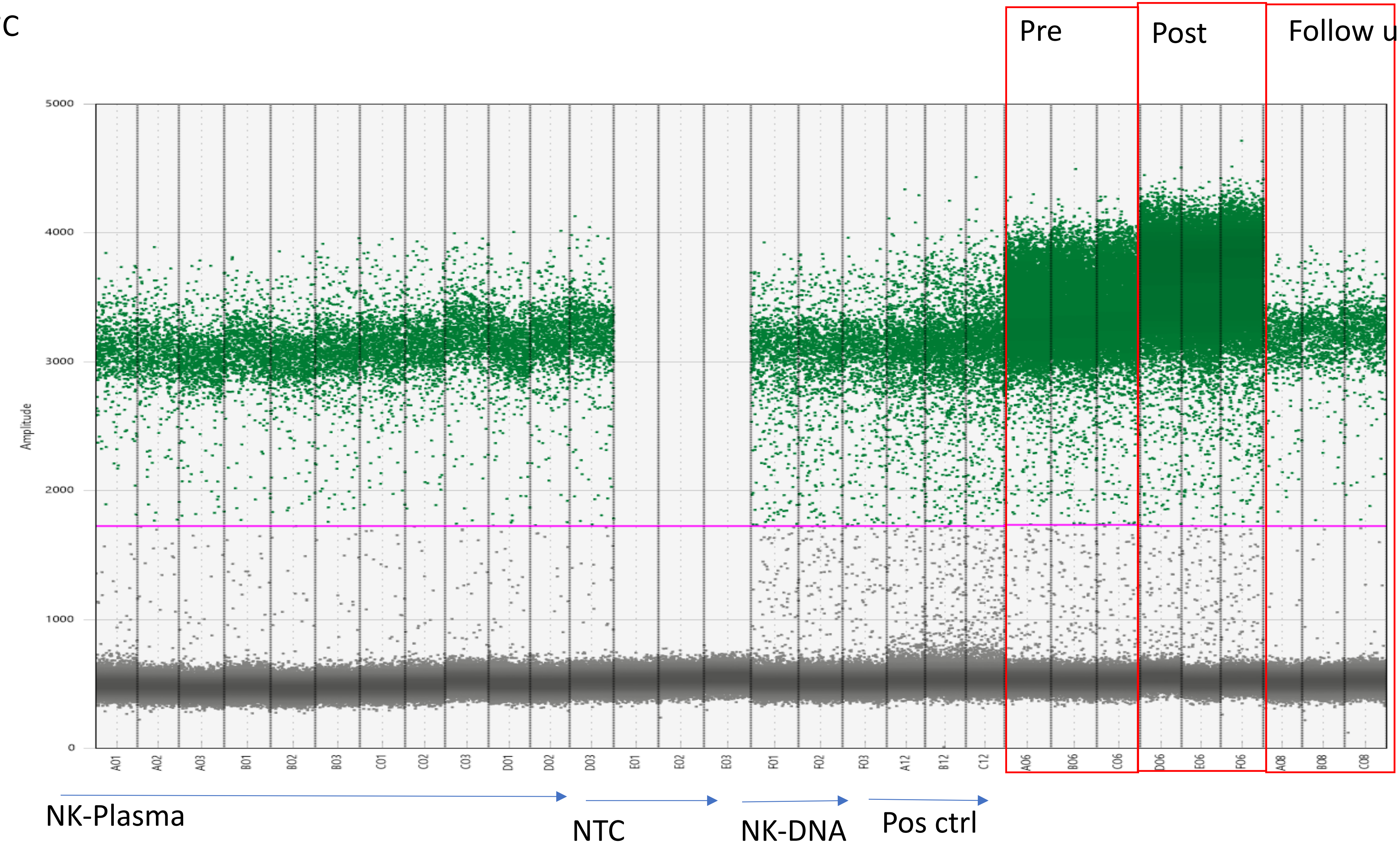

P27 - Final diluted

Gene targets : PIK3CA, p.H1047R,c.3140A>G and TP53, p.R273C, c.817C>T

Assays: 0.5X PIK3CA, p.H1047R mut (FAM) and 1X PIK3CA WT, p.H1047R (HEX)
1X TP53, p.R273C mut (FAM) and 1X dark probe for TP53, p.R273C

Annealing temp: 55°C
Protocol: Exp 23

0.5X PIK3CA, p.H1047R mut

1X TP53, p.R273C mut

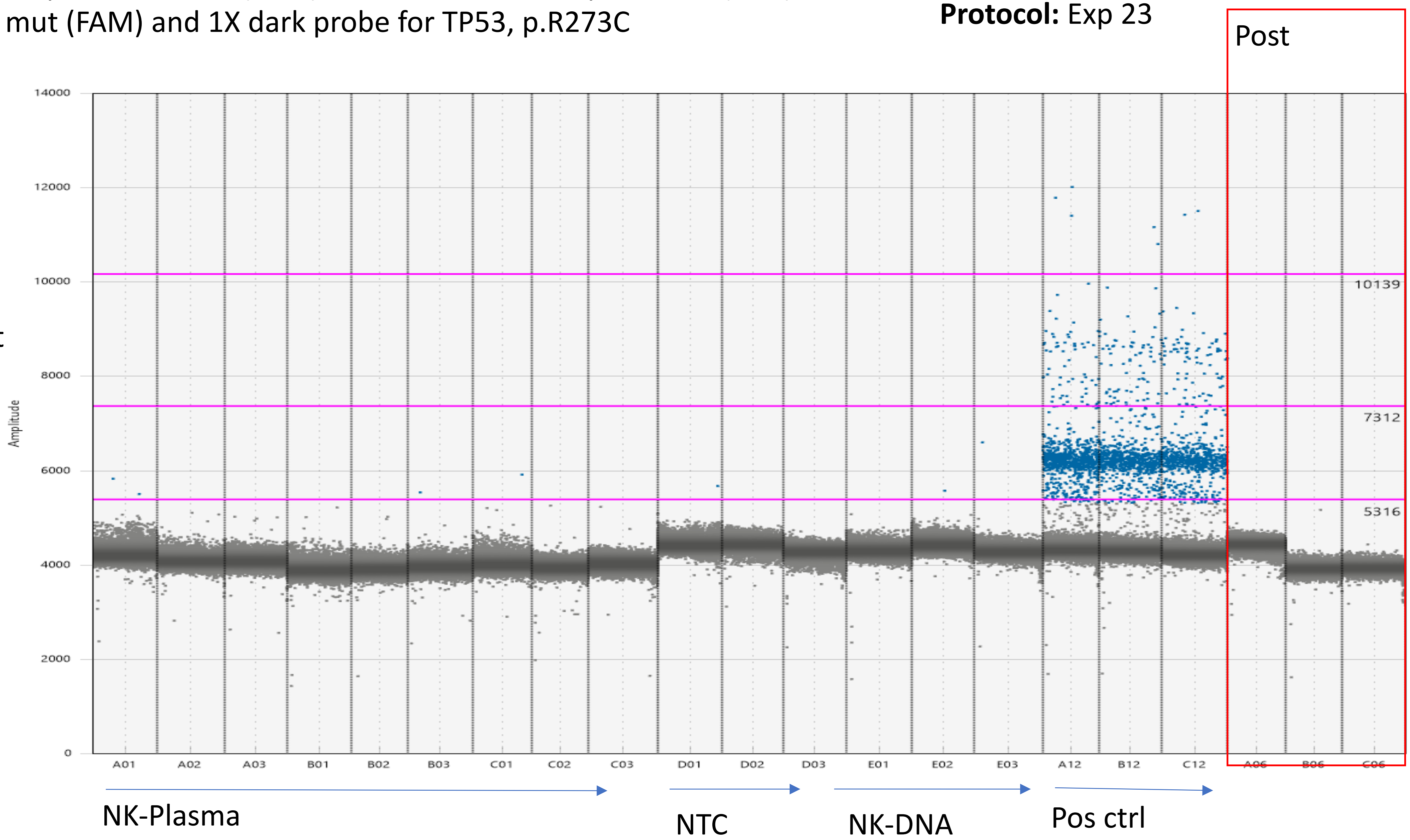

P27 - Final diluted -WT

Gene targets : PIK3CA, p.H1047R,c.3140A>G and TP53, p.R273C, c.817C>T

Assays: 1X PIK3CA WT, p.H1047R (HEX) and 1X dark probe for TP53, p.R273C

Annealing temp: 55°C

Protocol: Exp 23

0.5X PIK3CA WT, p.H1047R

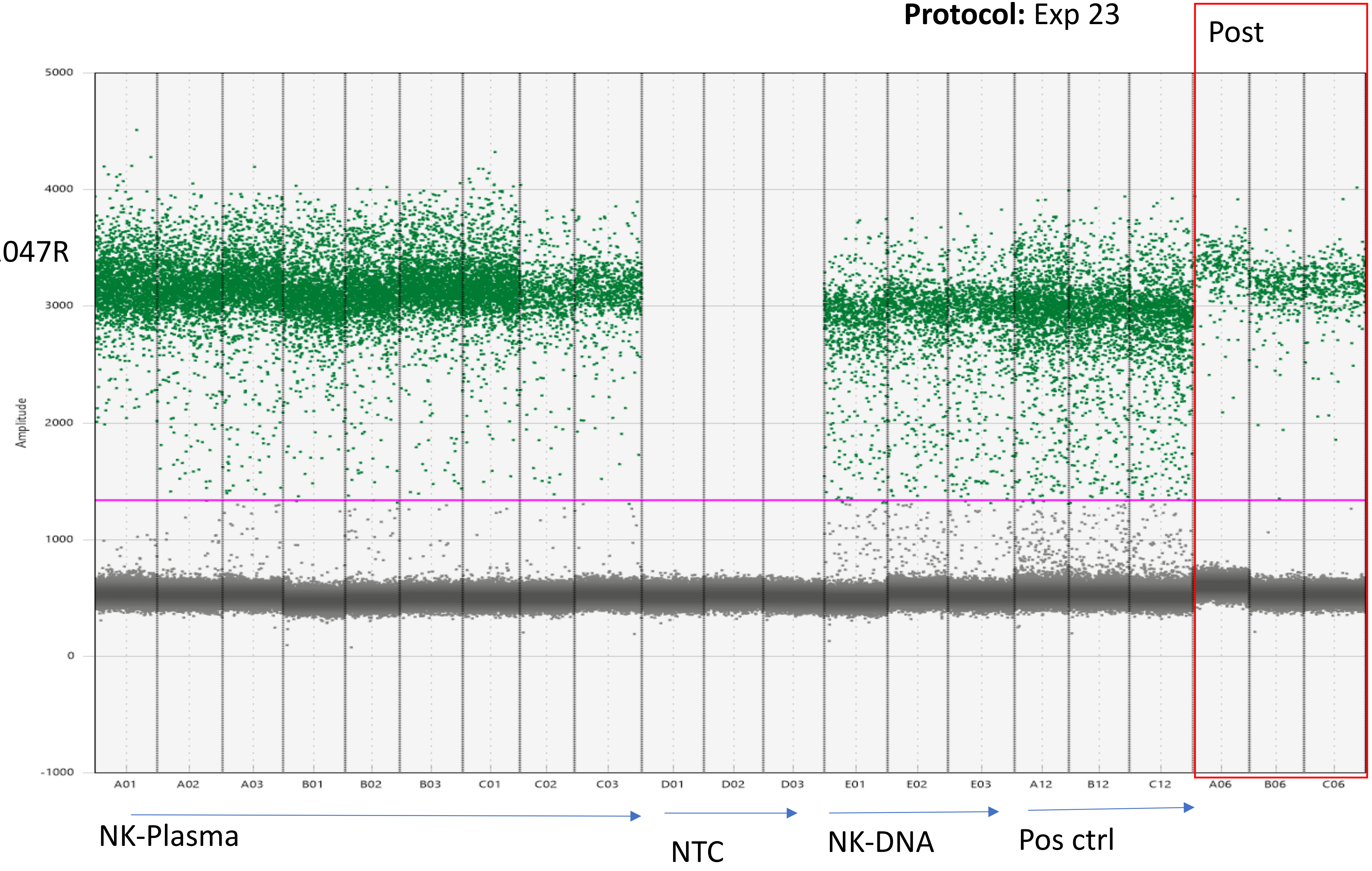

Positive – control

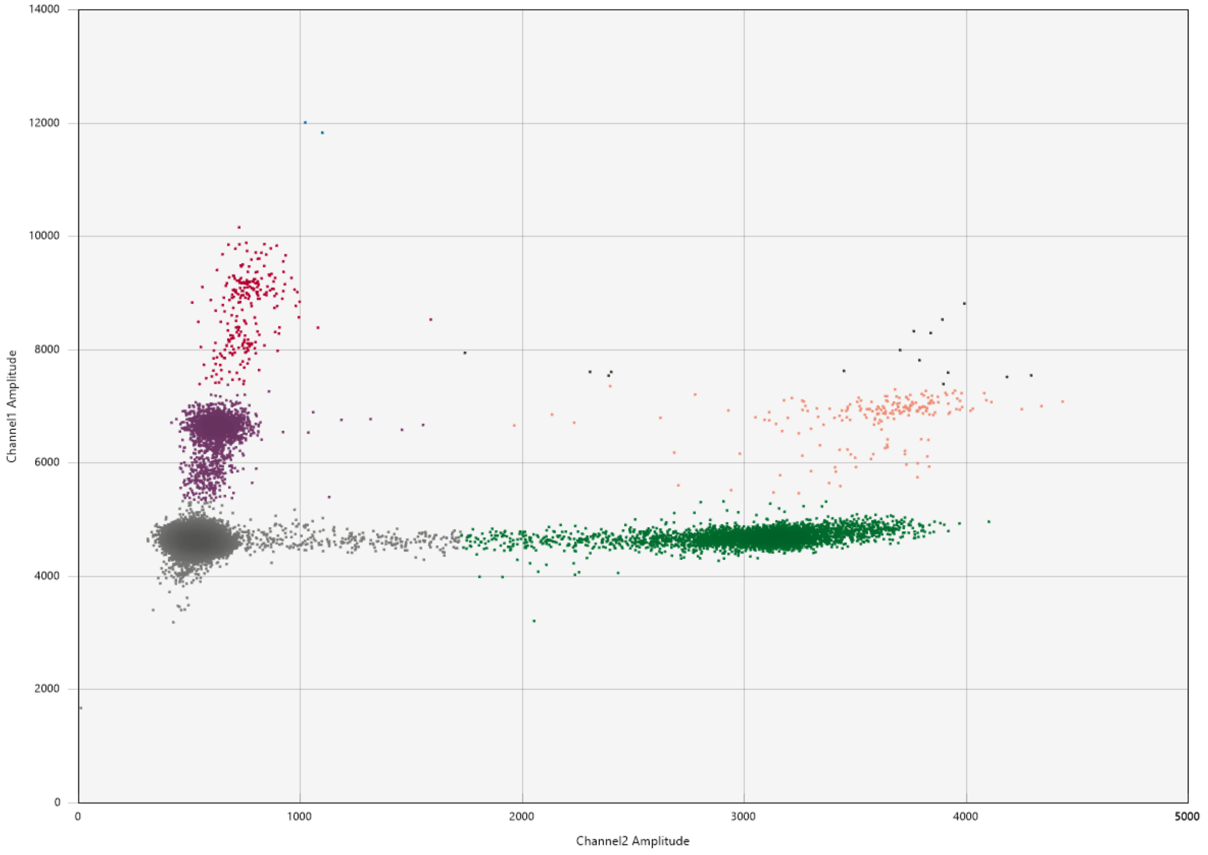

NK-Plasma

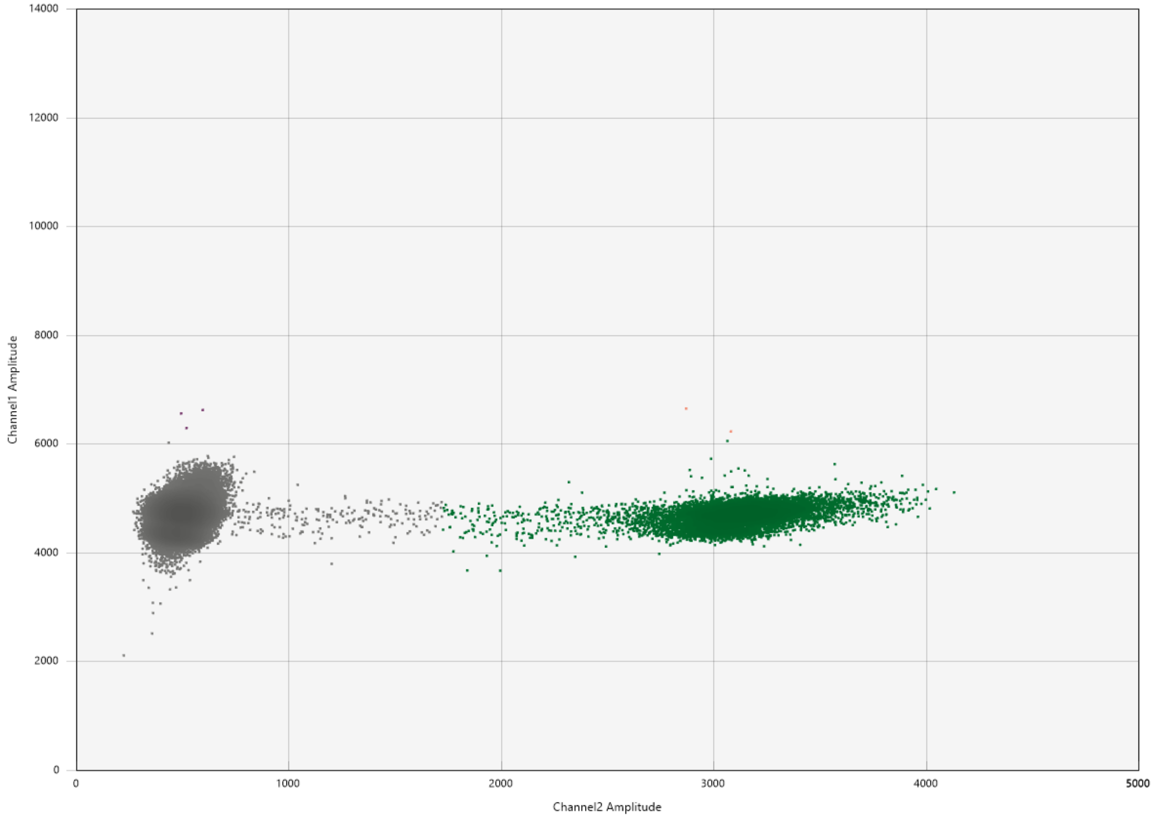

Pre-surgery

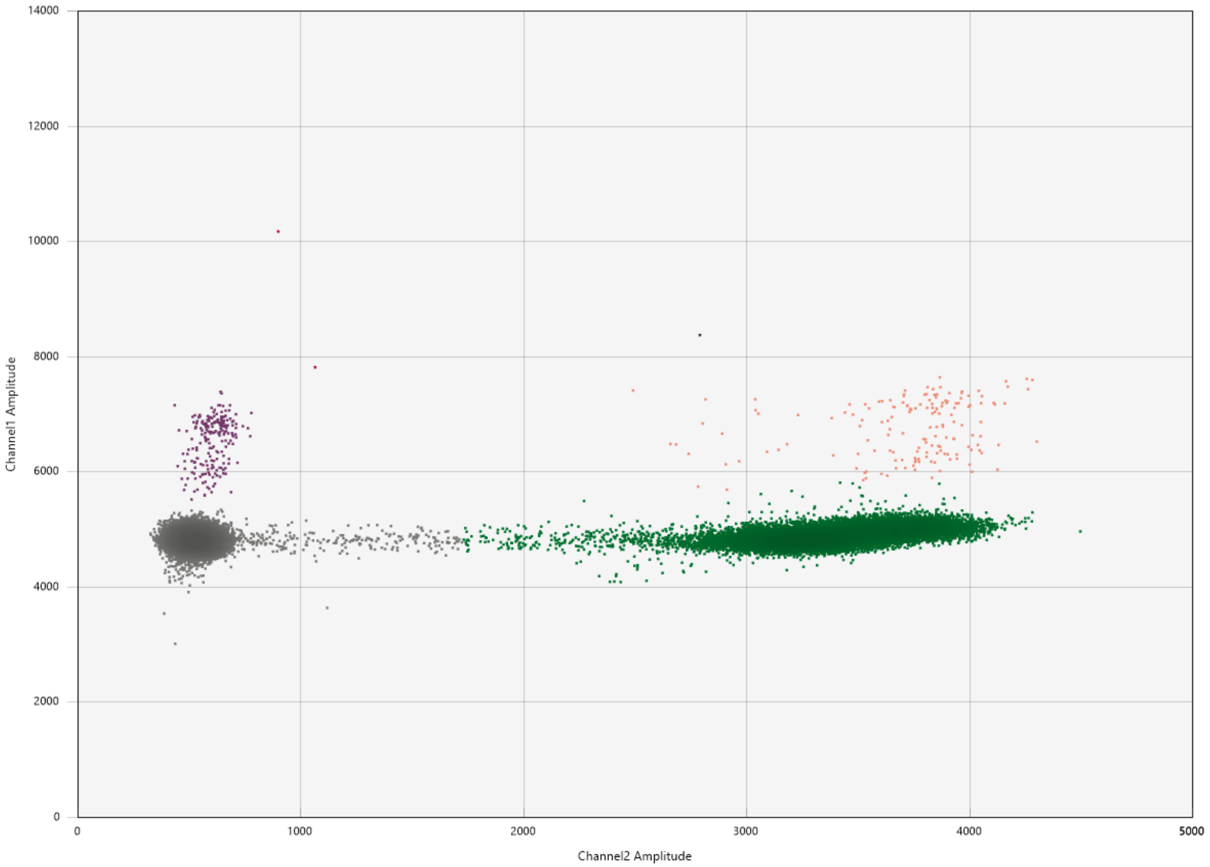

Follow-up 1

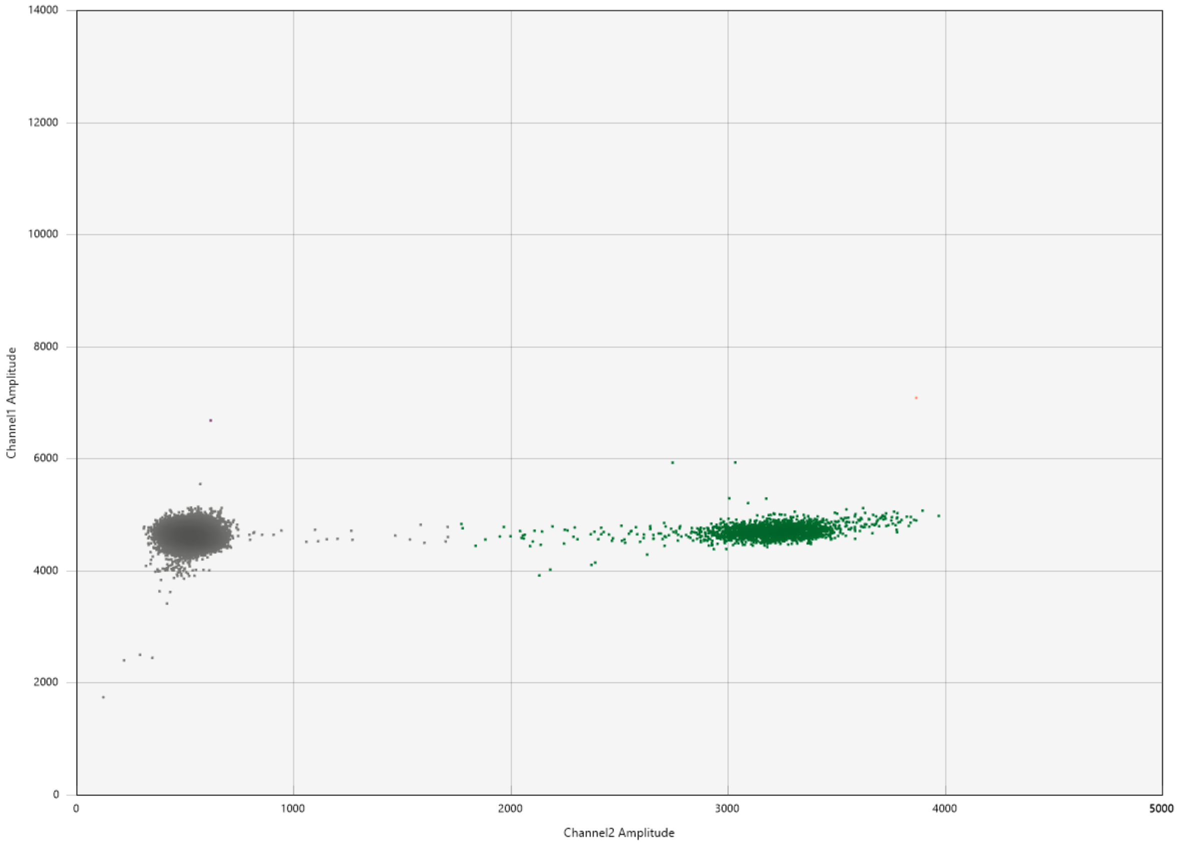

Post-surgery

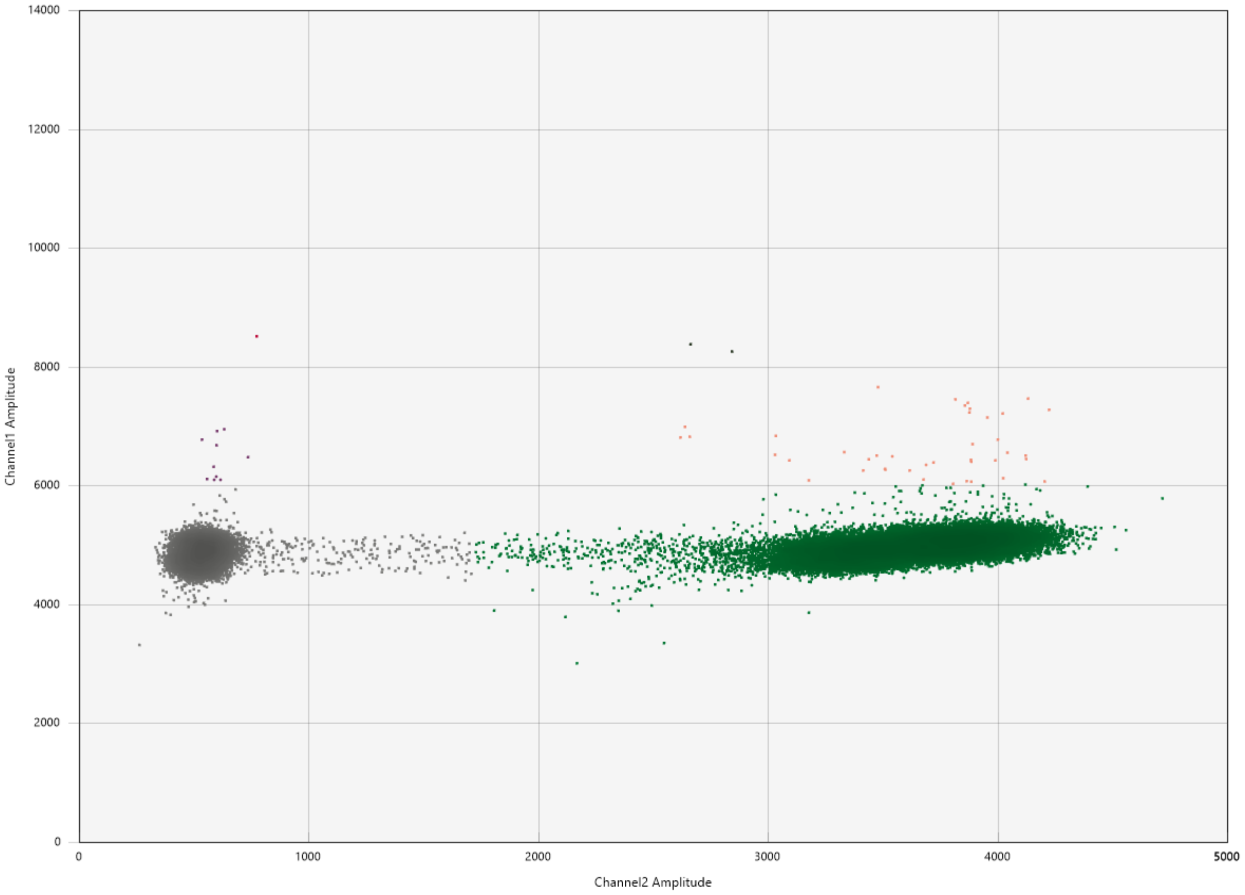

Post-surgery diluted

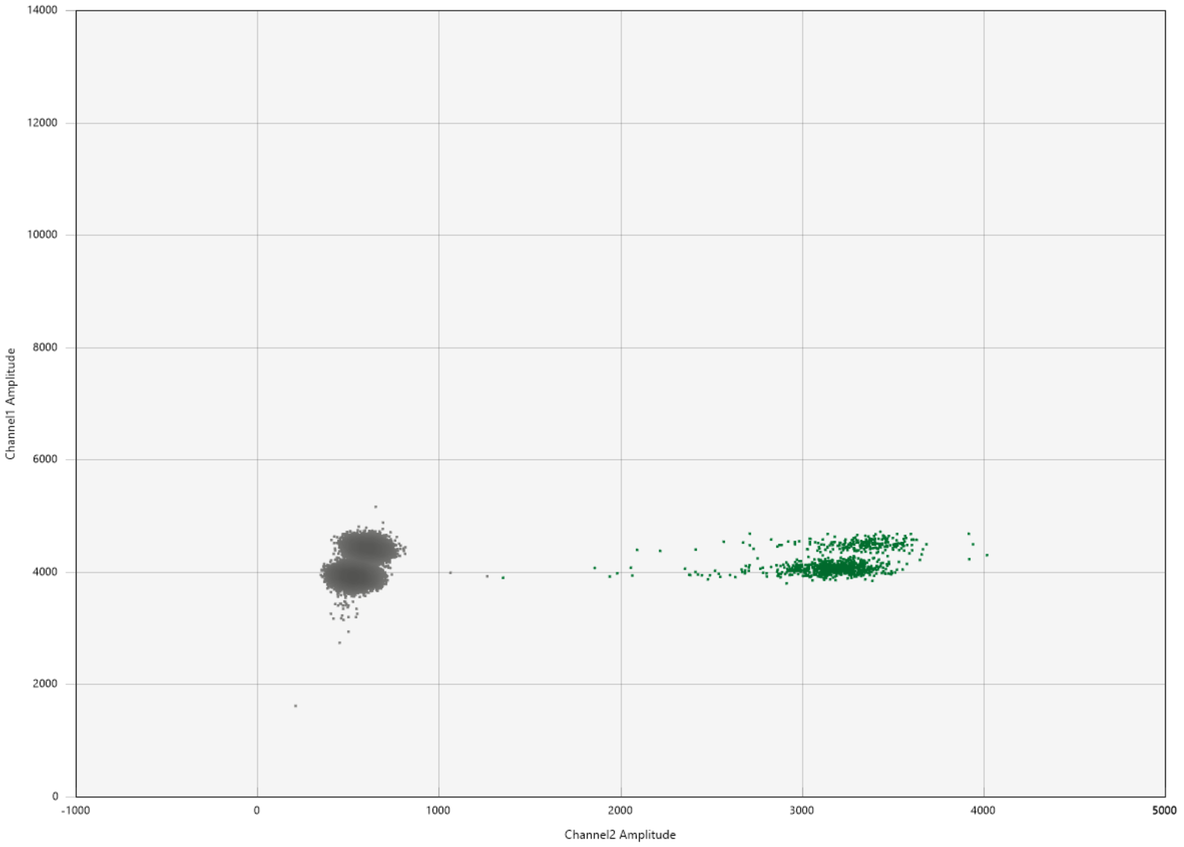

Concentration plots Pre, post and follow-up

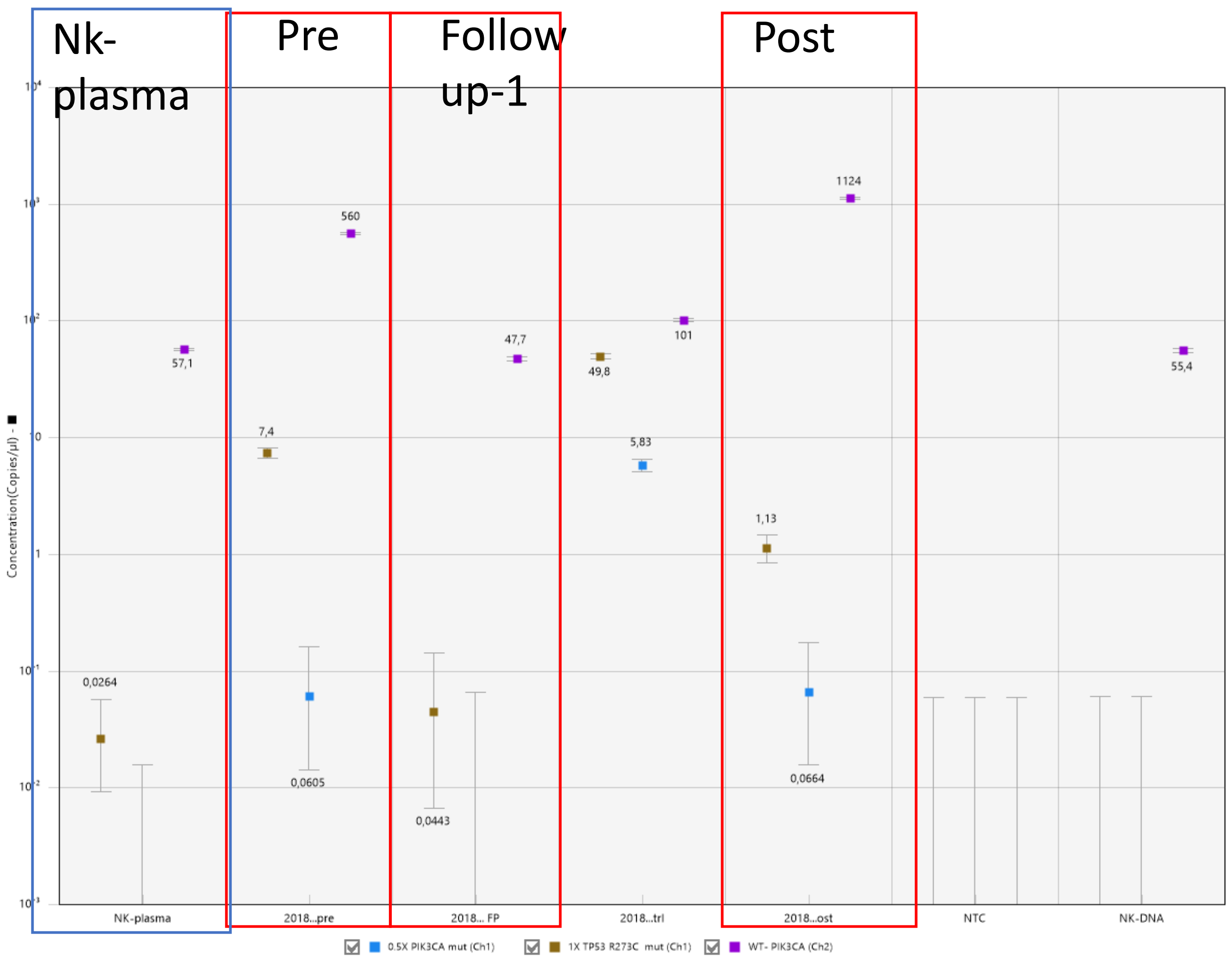

Post 10X dilution

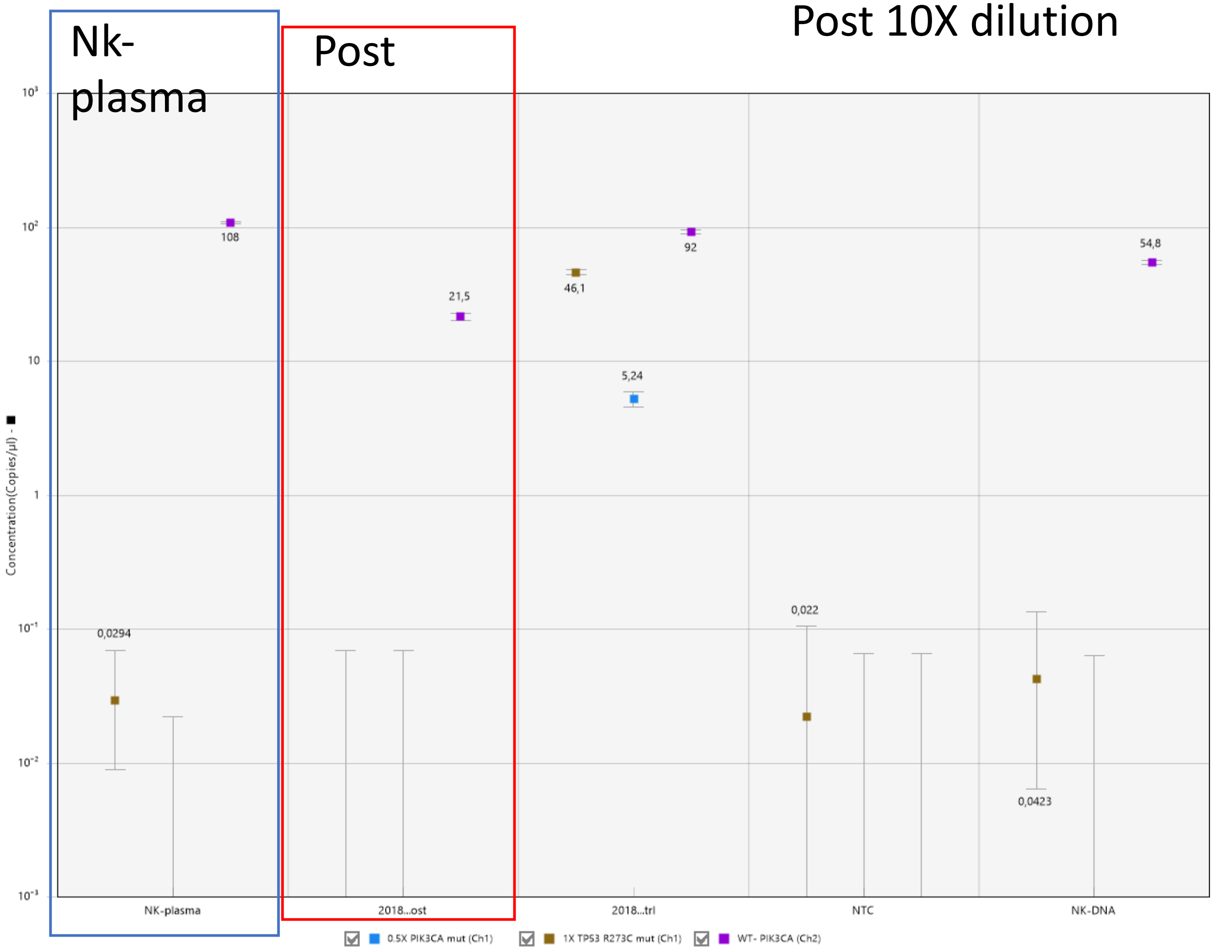

Calling – Pre-op- positive for –PIK3CA and TP53 R273C
Post-op- positive for PIK3CA and TP53 R273C
Follow-up- negative for PIK3CA and TP53 R273C

Calling – After dilution
Post-op- negative for PIK3CA and TP53 R273C

P43 - Final

Gene target : KRAS, p.G12D, c.35G>A

Assays: 1X KRAS, p.G12D mut (FAM) and 1X KRAS WT, p.G12D (HEX)

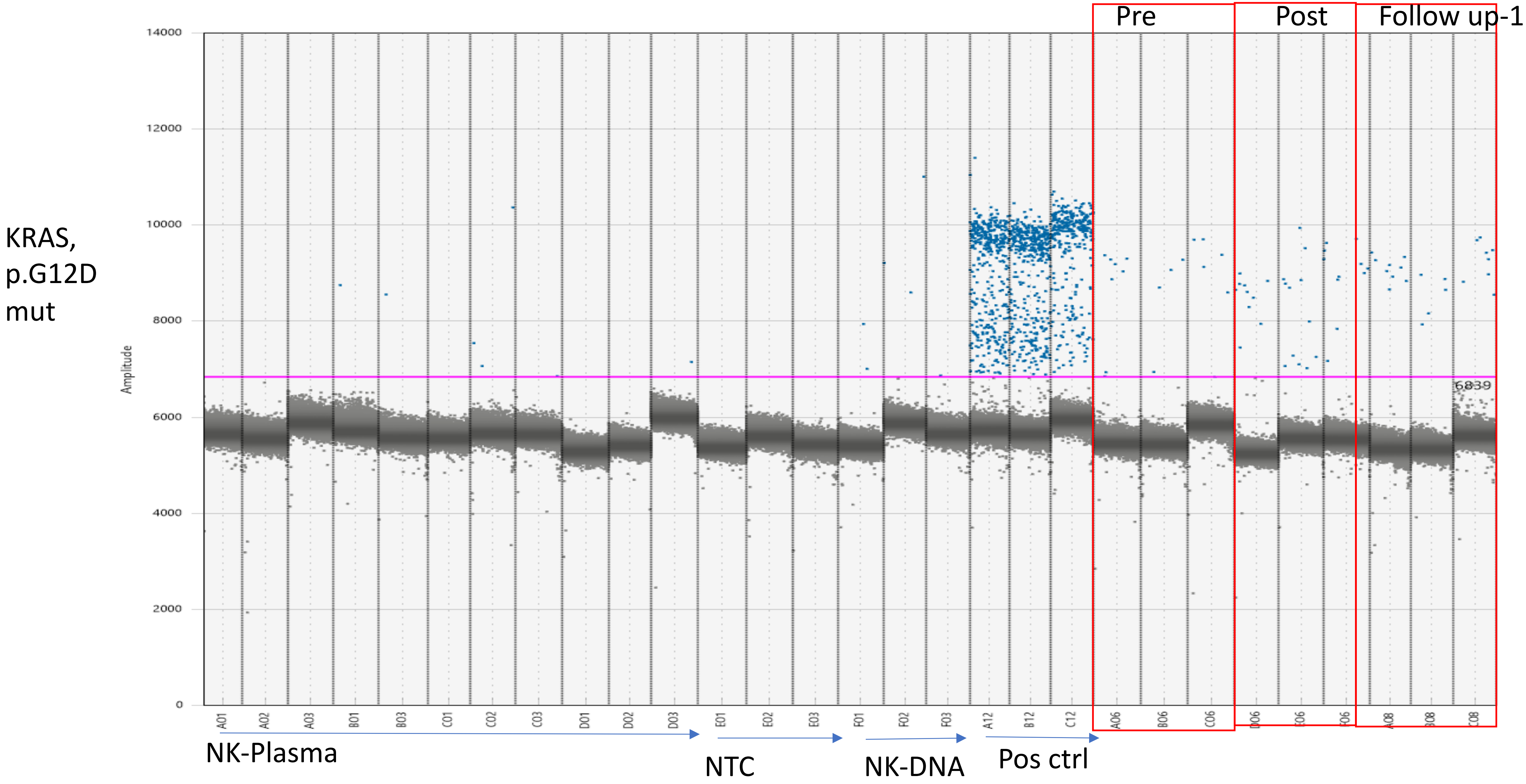

P43 - Final-WT

Gene target : KRAS, p.G12D, c.35G>A

Assay: 1X KRAS WT, p.G12D (HEX)

KRAS WT, p.G12D

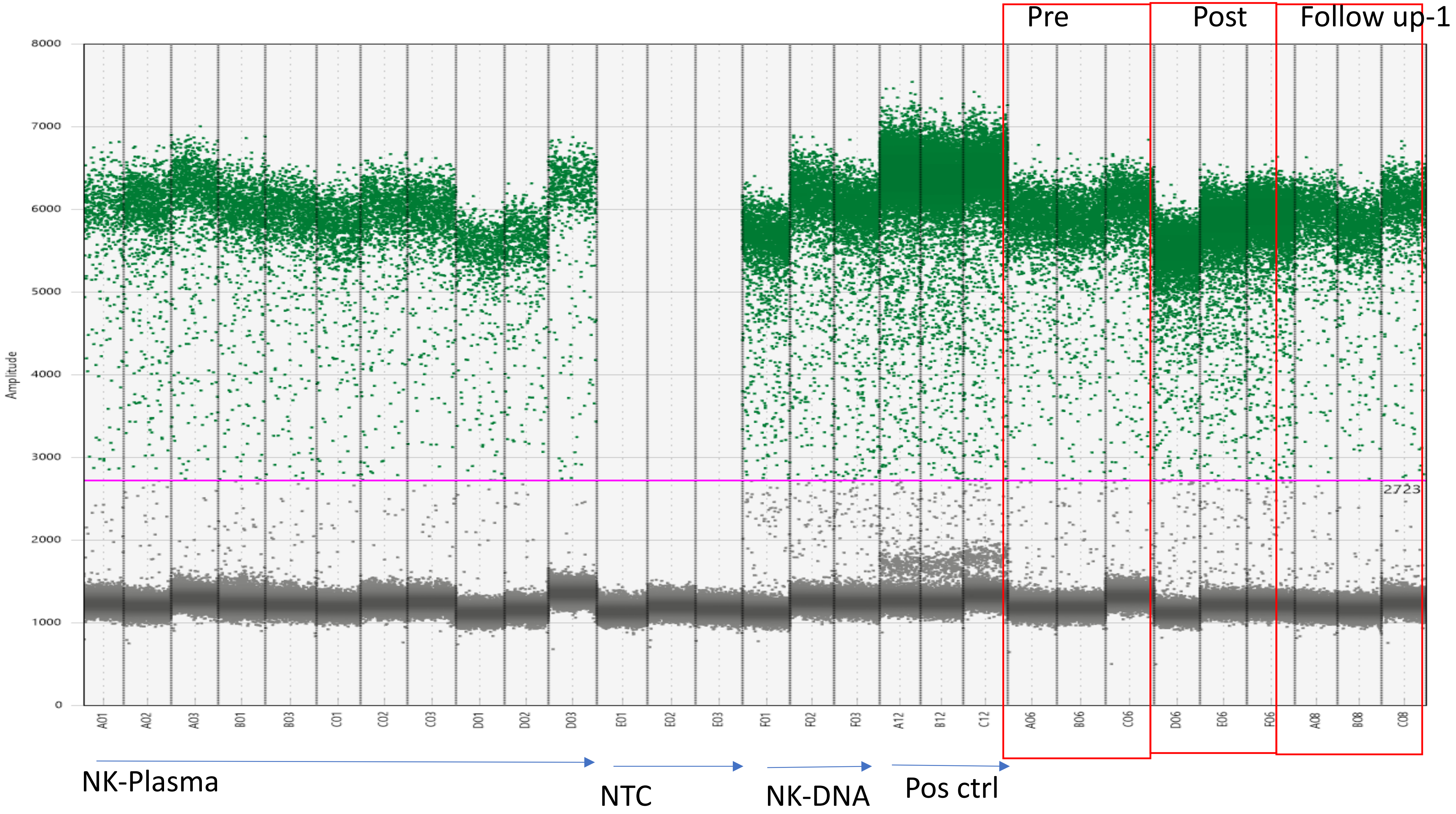

Positive – control

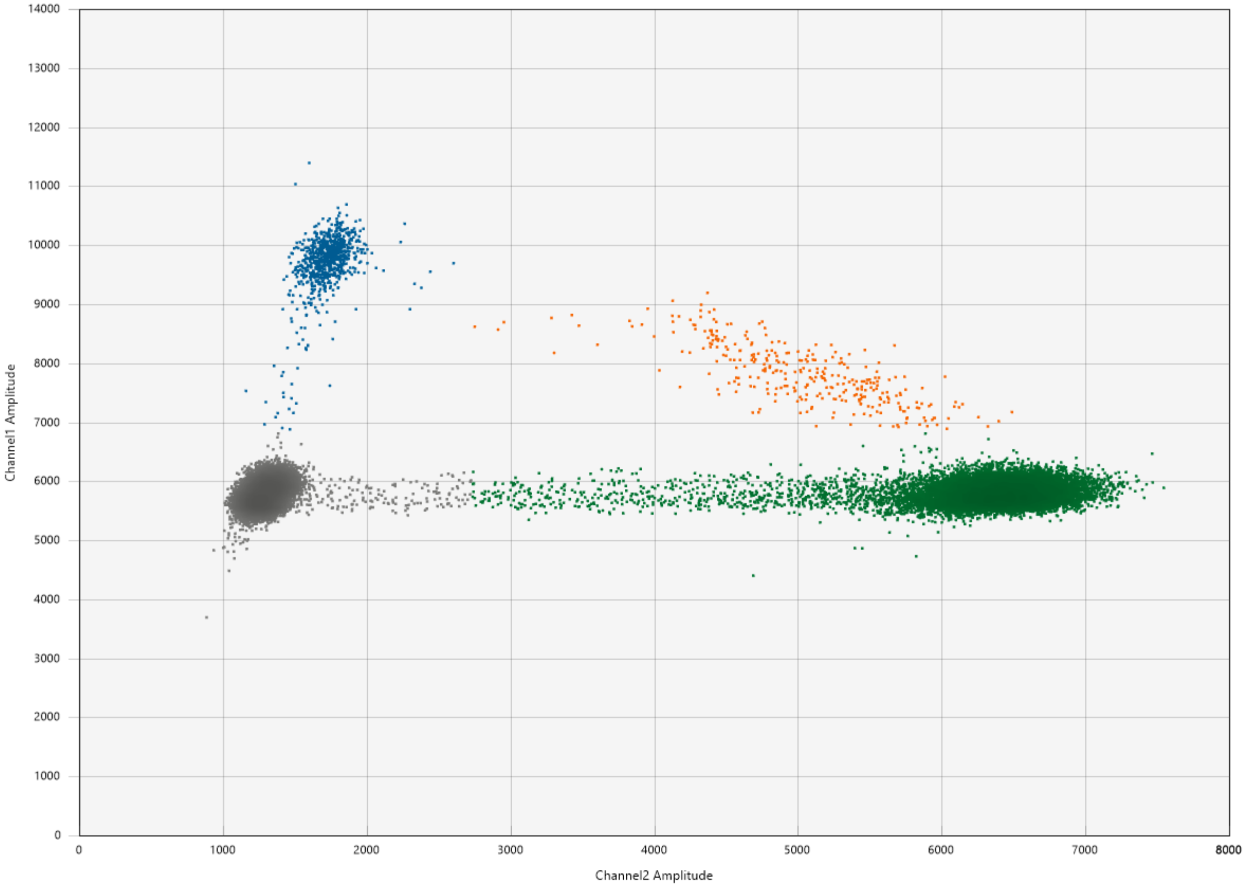

NK-Plasma

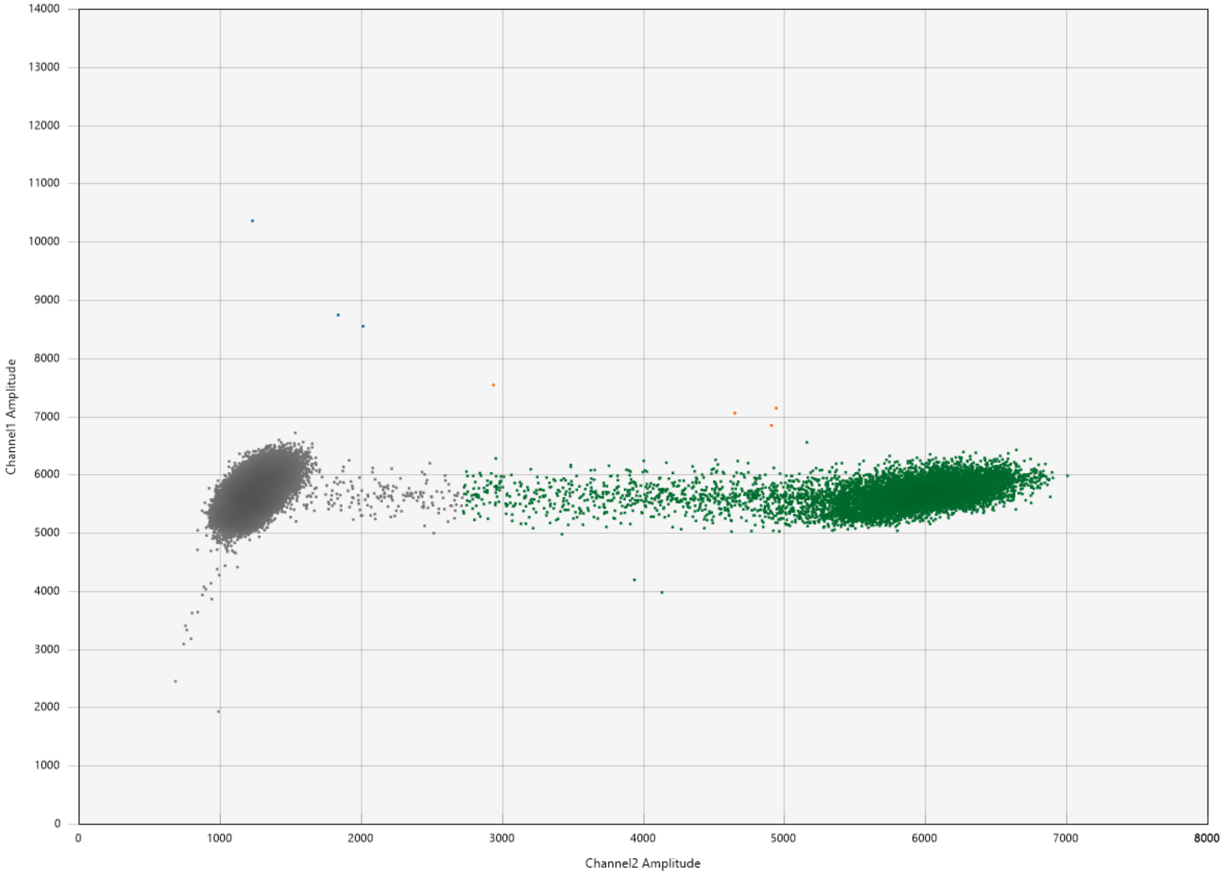

Pre-surgery

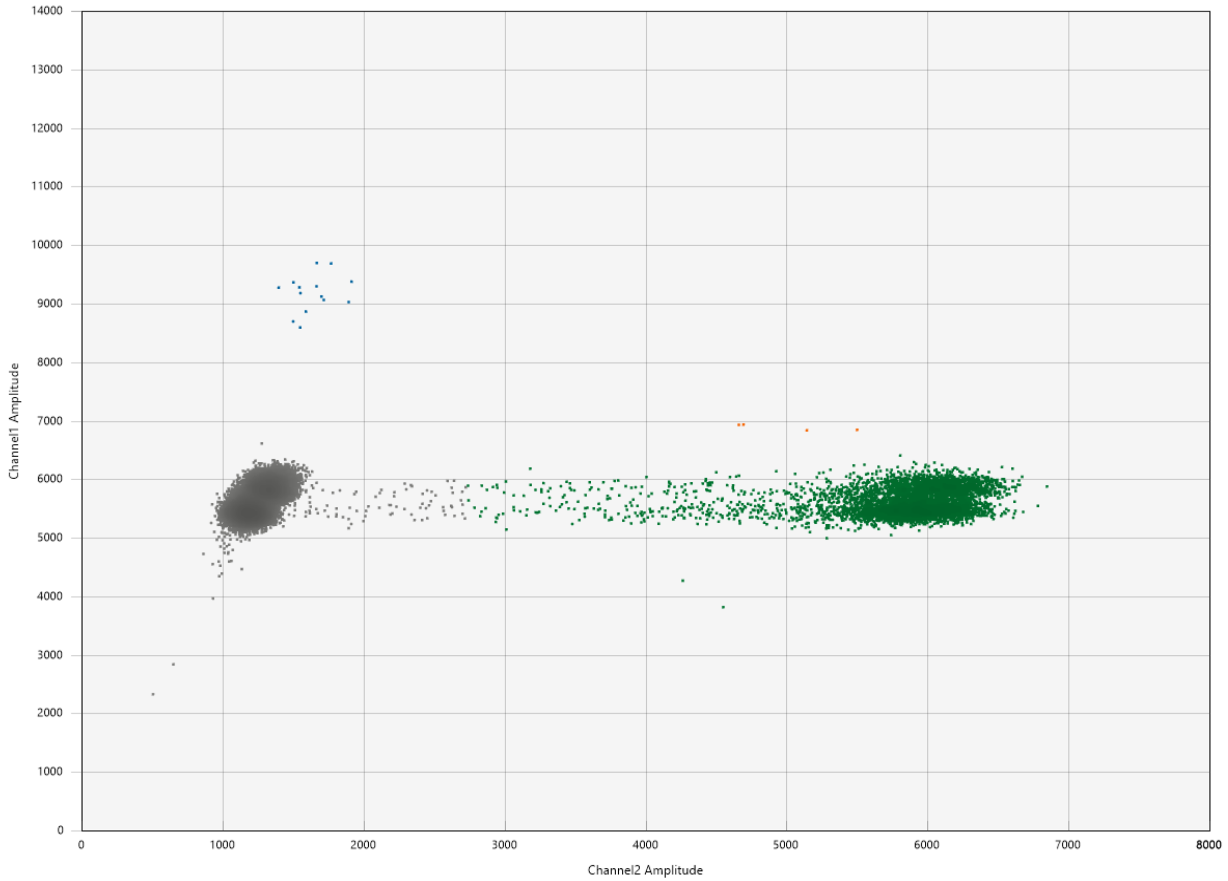

Post-surgery

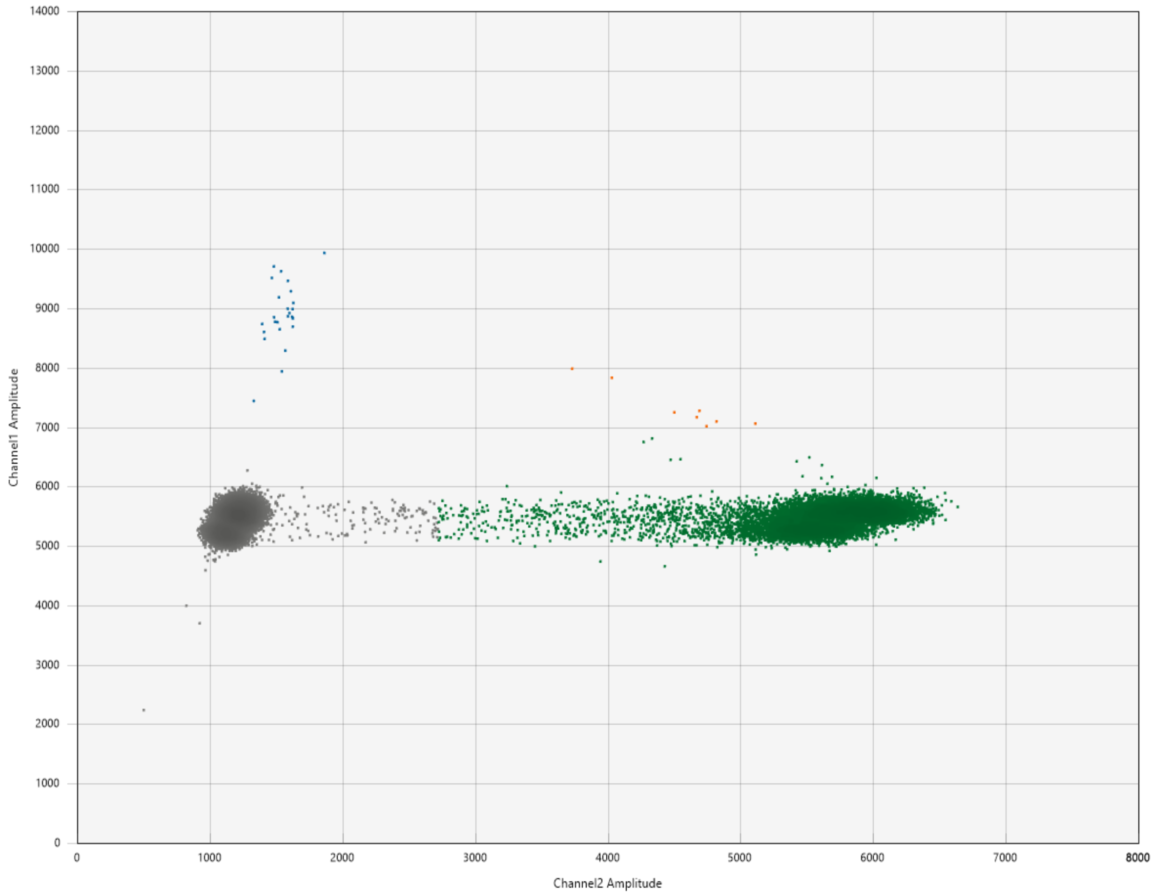

Follow-up 1

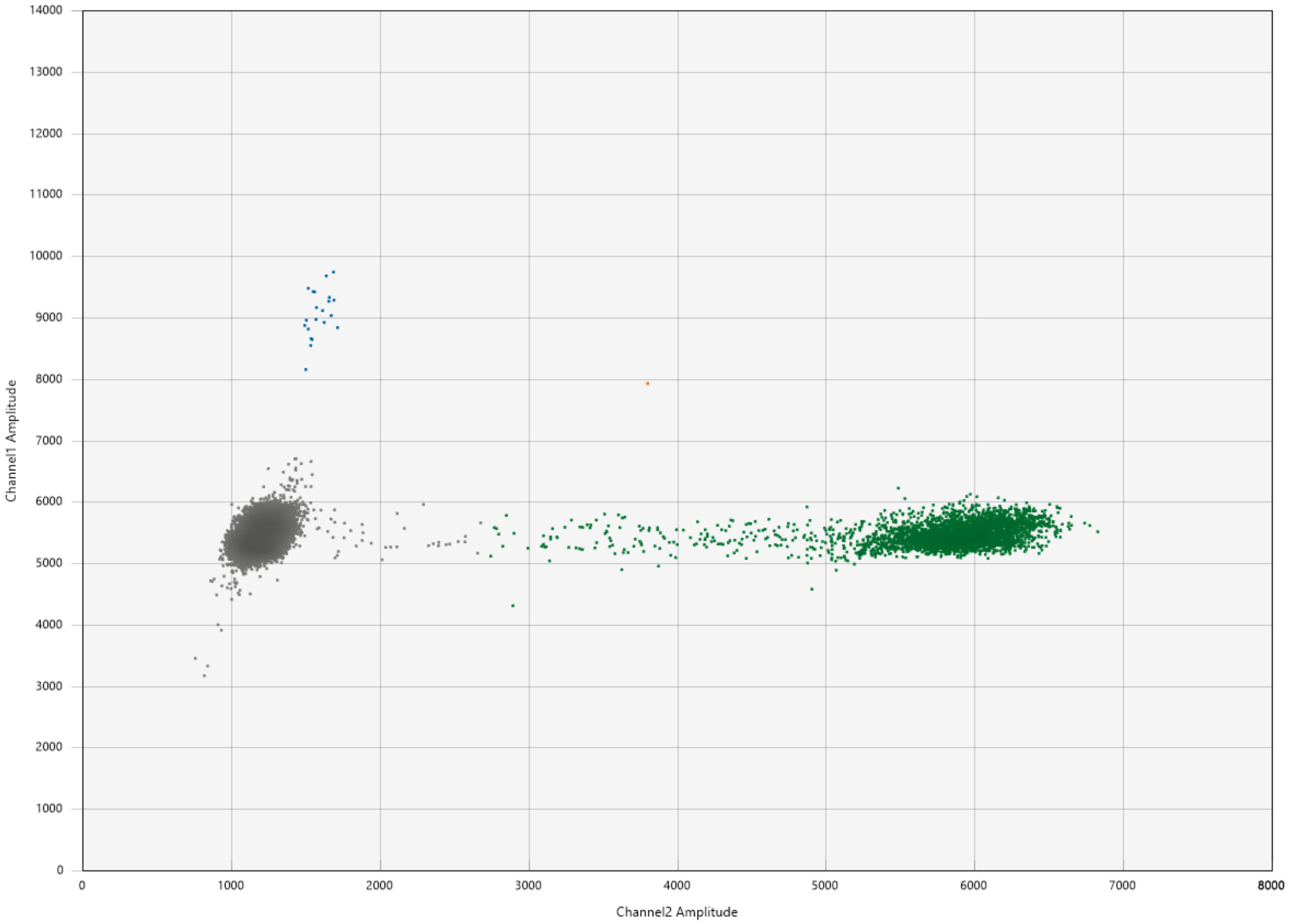

14000
13000
12000
11000
10000
9000
8000
7000
6000
5000
4000
3000
2000
1000
0
Channel1 Amplitude
0
1000
2000
3000
4000
5000
6000
7000
8000
Channel2 Amplitude

Concentration plots

Pre, post and follow-up

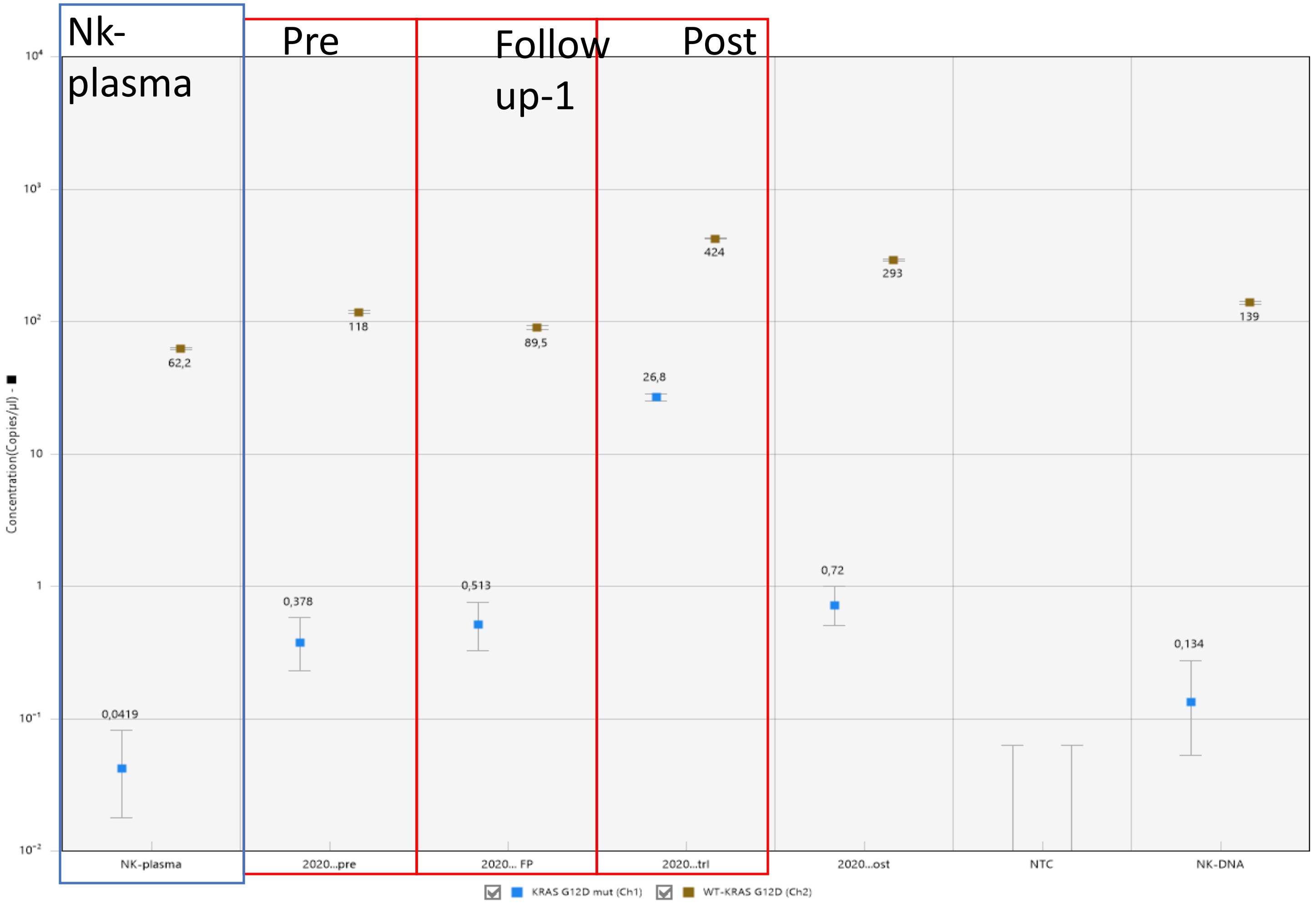

Calling – Pre-op- positive for –KRAS G12D
Post-op- positive for KRAS G12D
Follow-up- positive for KRAS G12D

P38 - Final

Gene target :TP53, p.R248W, c.742C>T

Assays: 1X TP53, p.R248W mut (FAM) and 1X TP53 WT, p.R248W (HEX)

1X TP53, p.R248W mut

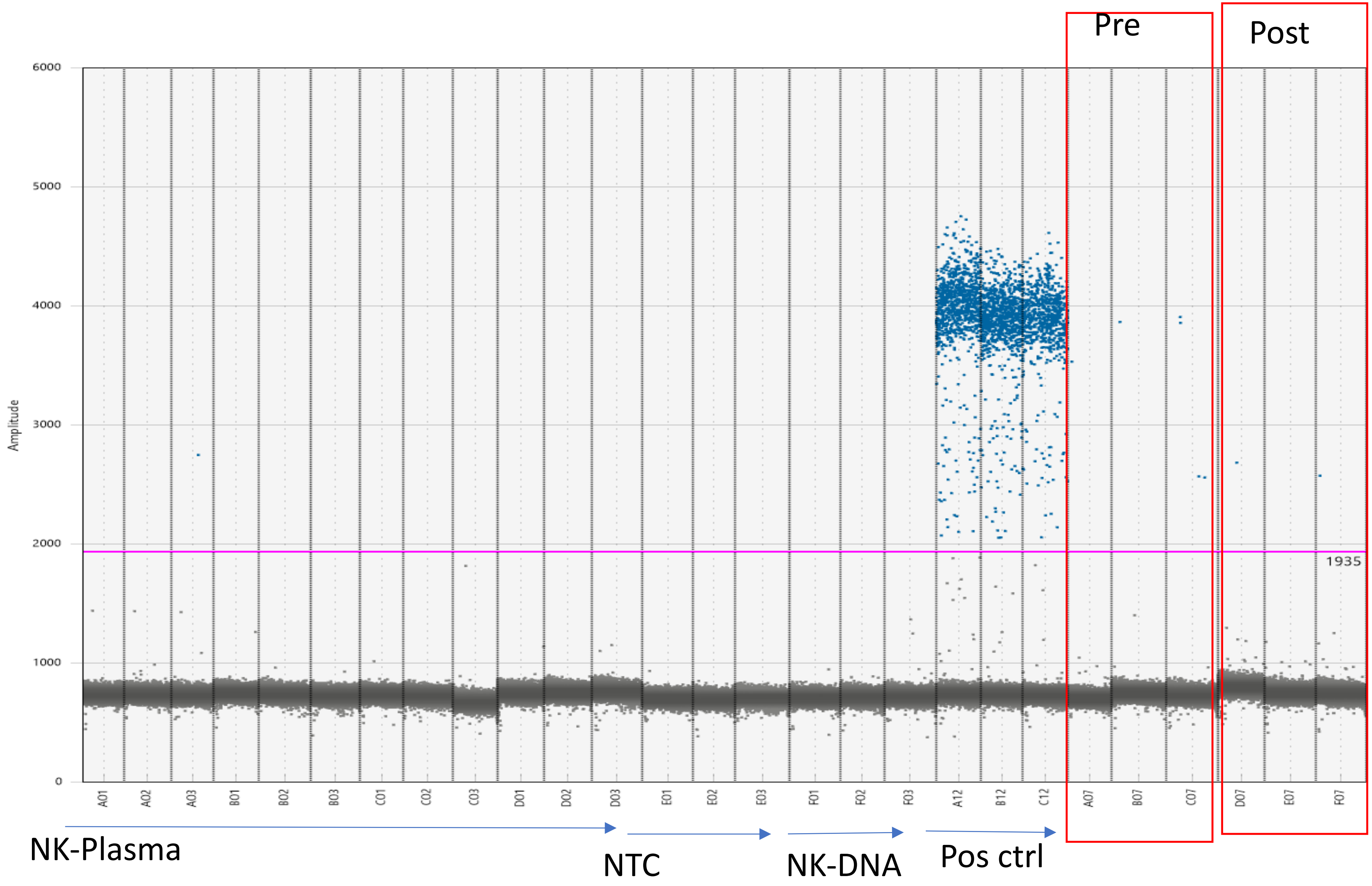

P38 -Final -WT

Gene target :TP53, p.R248W, c.742C>T

Assays: 1X TP53 WT, p.R248W (HEX)

1X TP53 WT, p.R248W

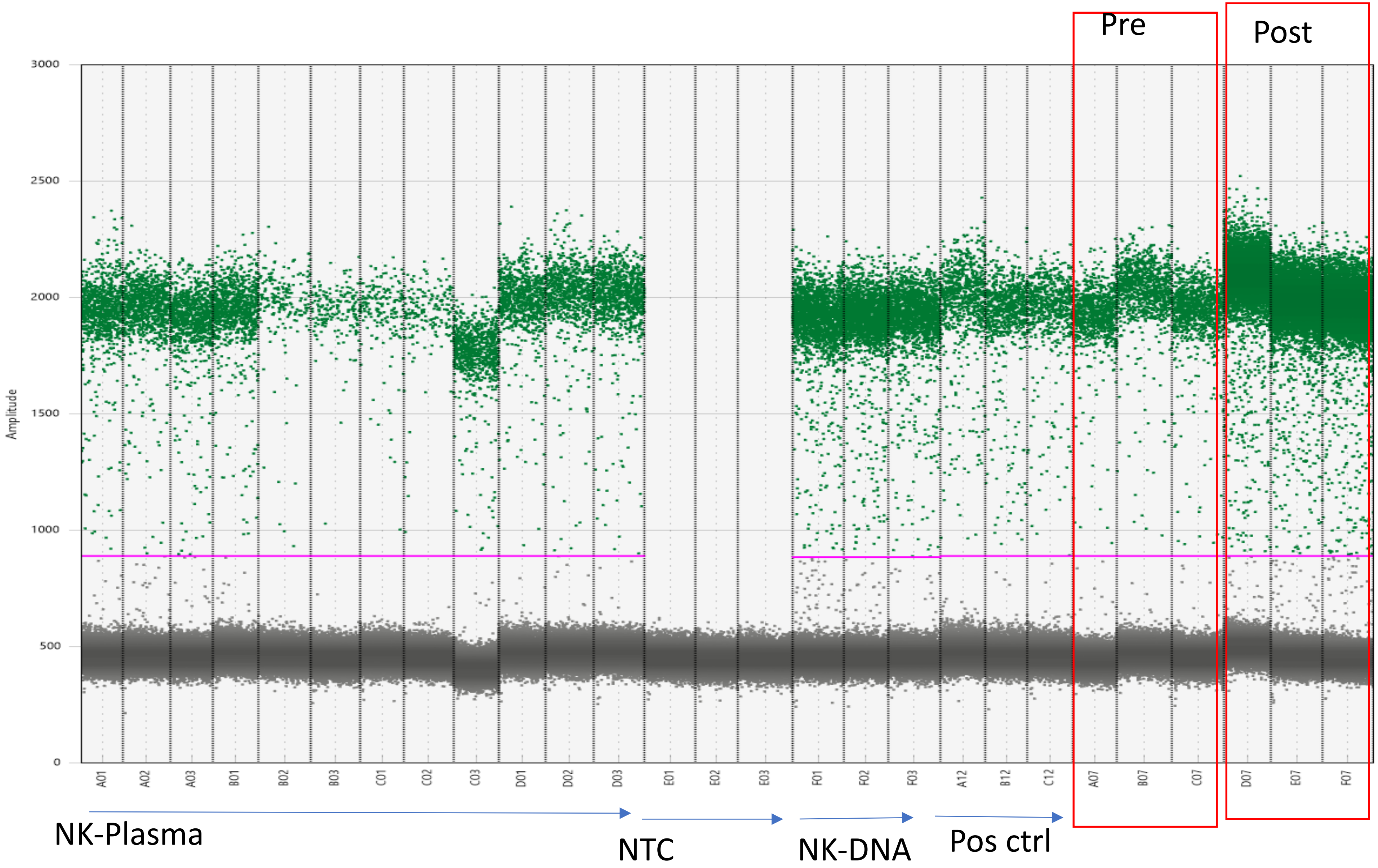

NK-plasma DNA

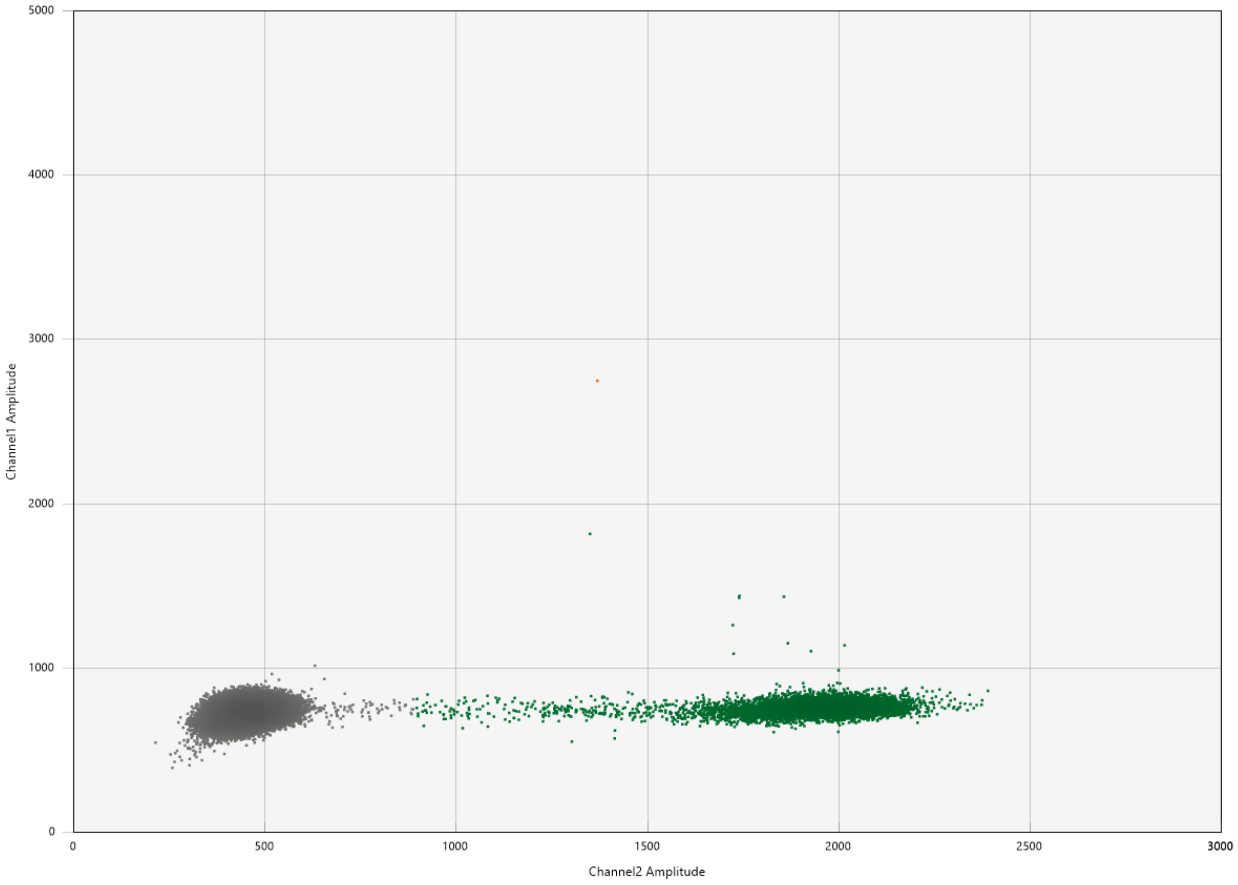

Positive control

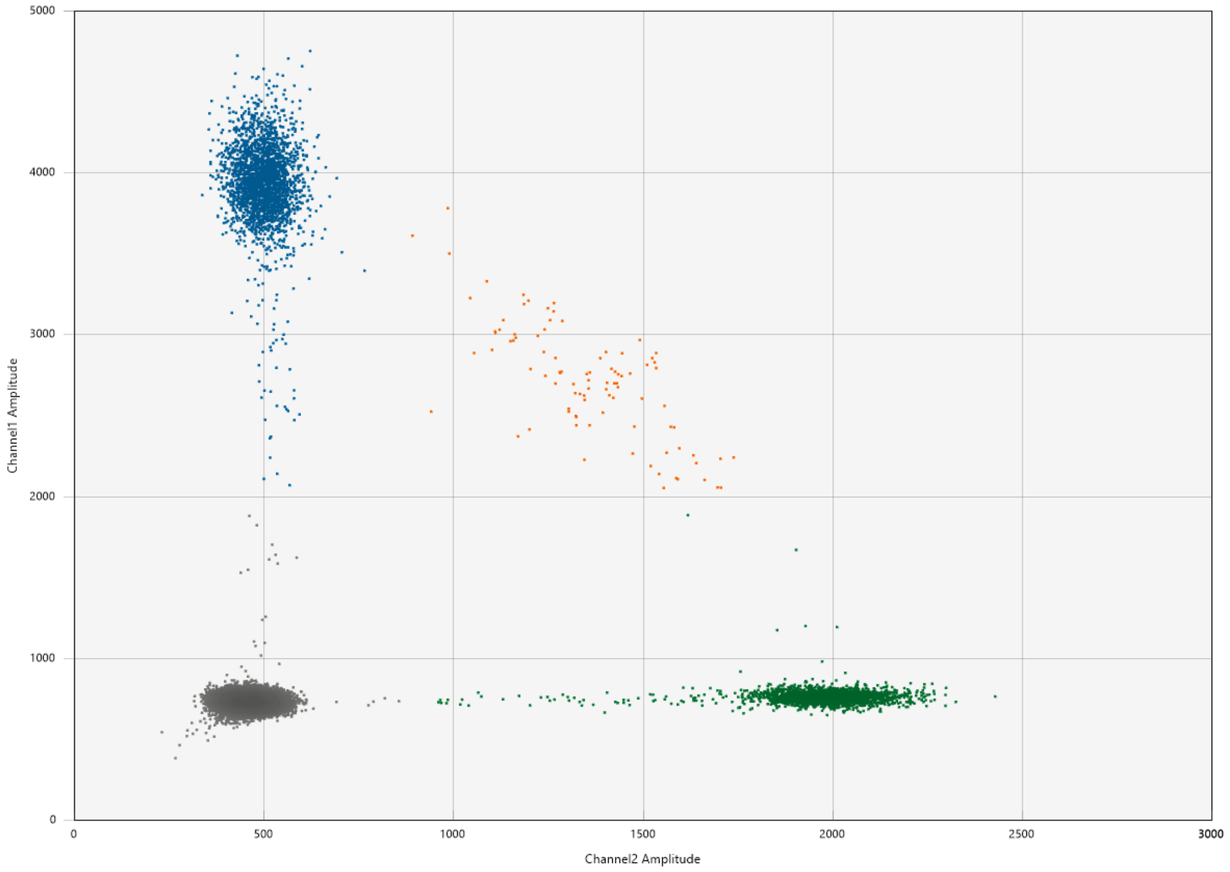

Pre-surgery

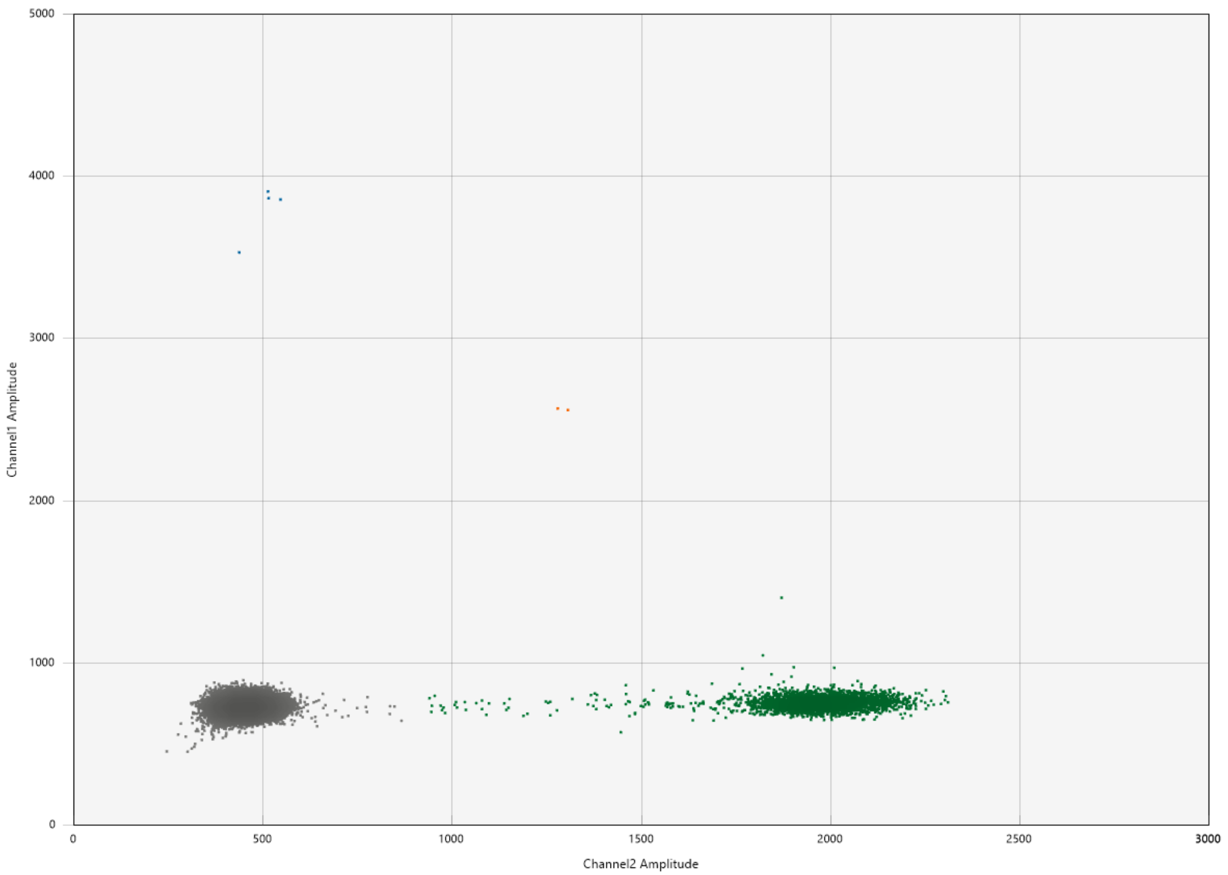

Post-surgery

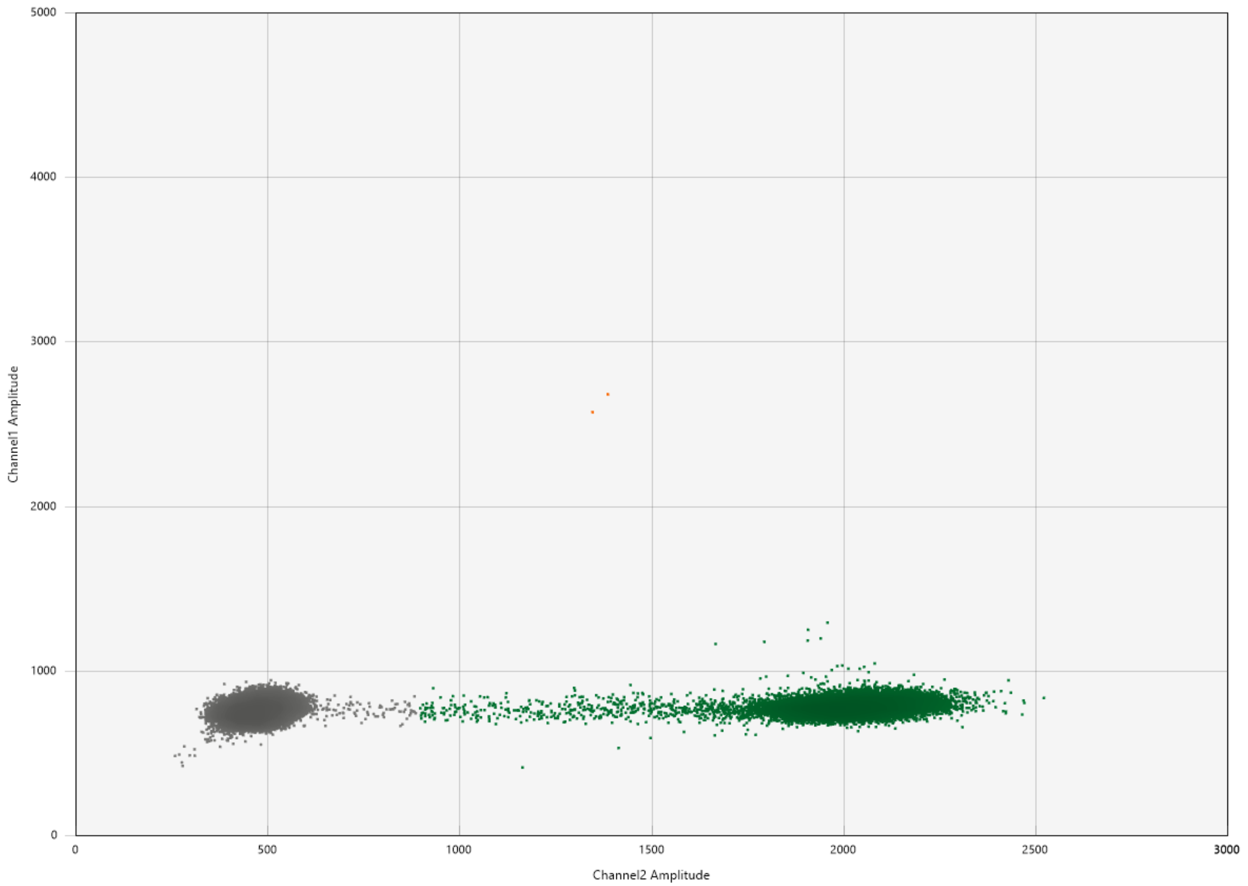

Concentration plots

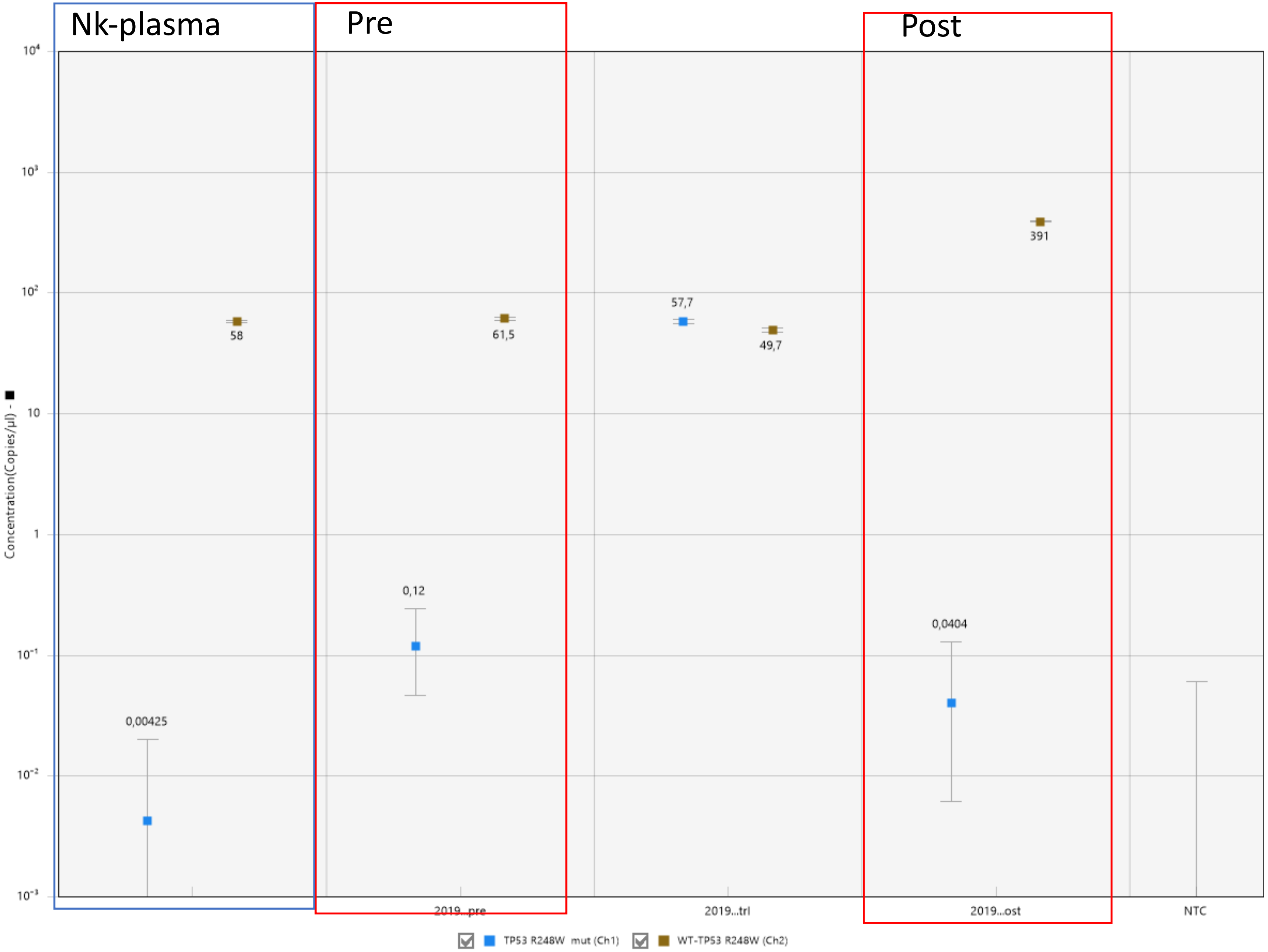

P42 - Final

Gene target : TP53,p.R158fs*12 , c.472del

Assays: 1X TP53 ,p.R158fs*12 mut (FAM) and 1X TP53 WT ,p.R158fs*12 (HEX)

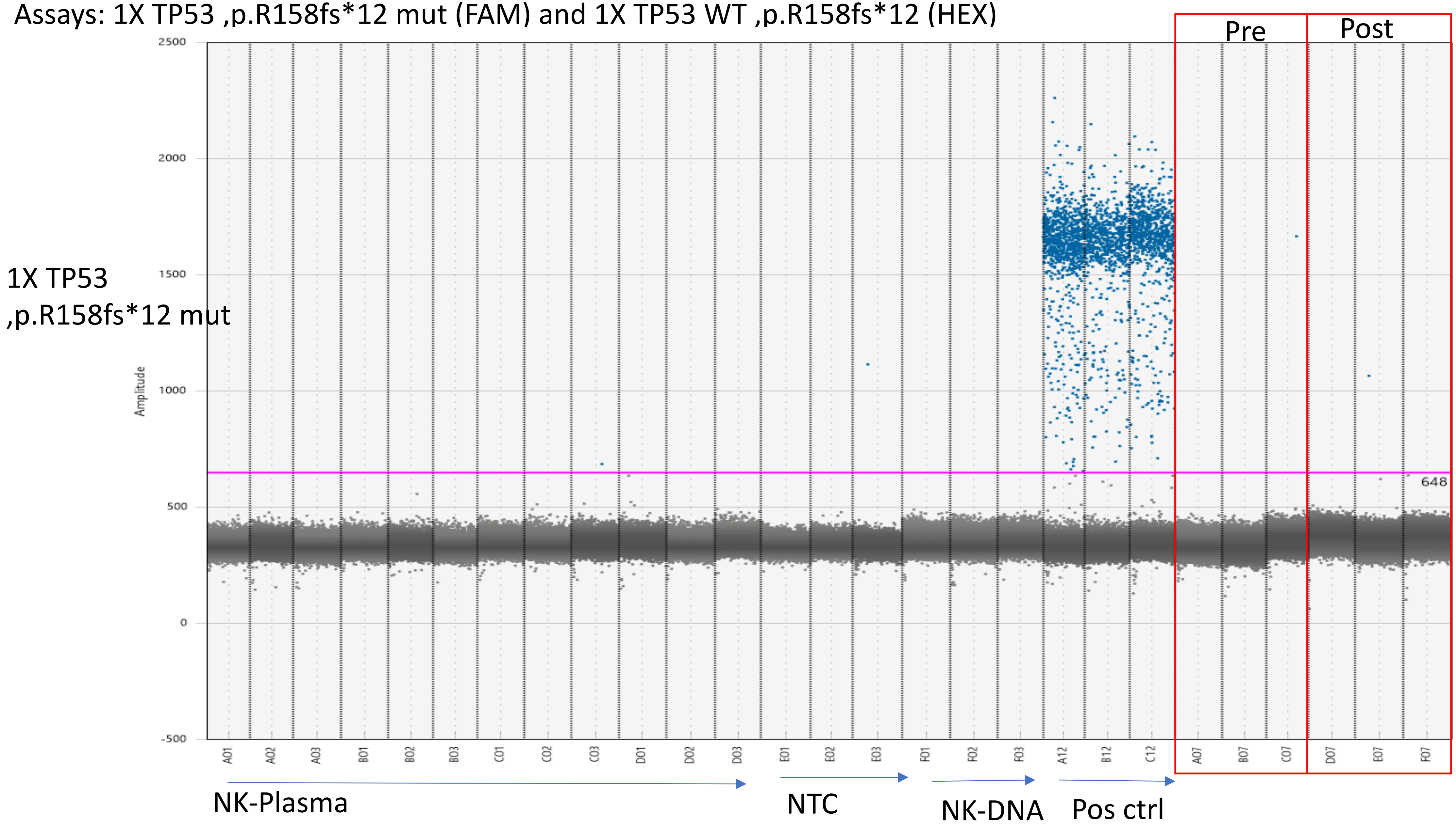

P42 - Final -WT

Gene target : TP53,p.R158fs*12 , c.472del

Assays: 1X TP53 WT ,p.R158fs*12 (HEX)

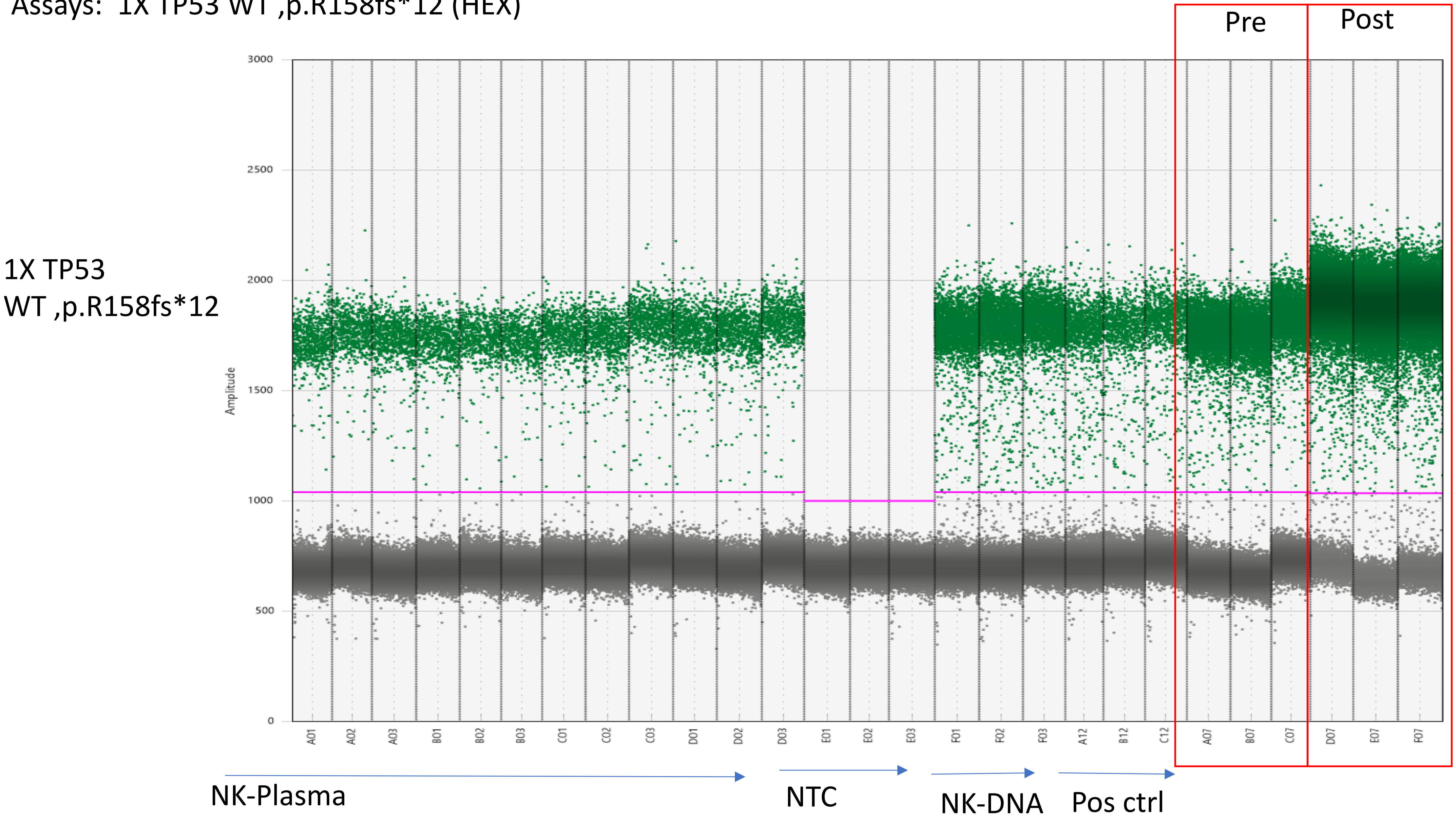

Positive – control

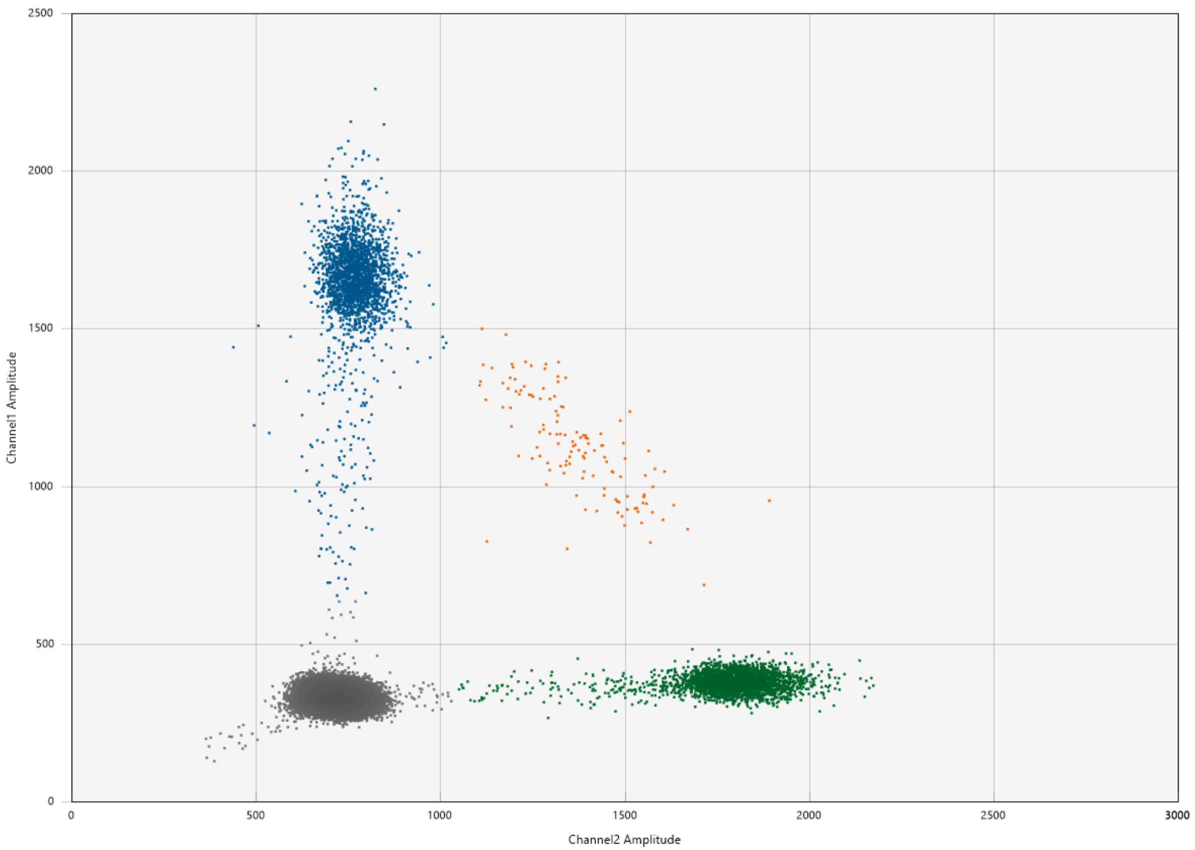

Pre-surgery

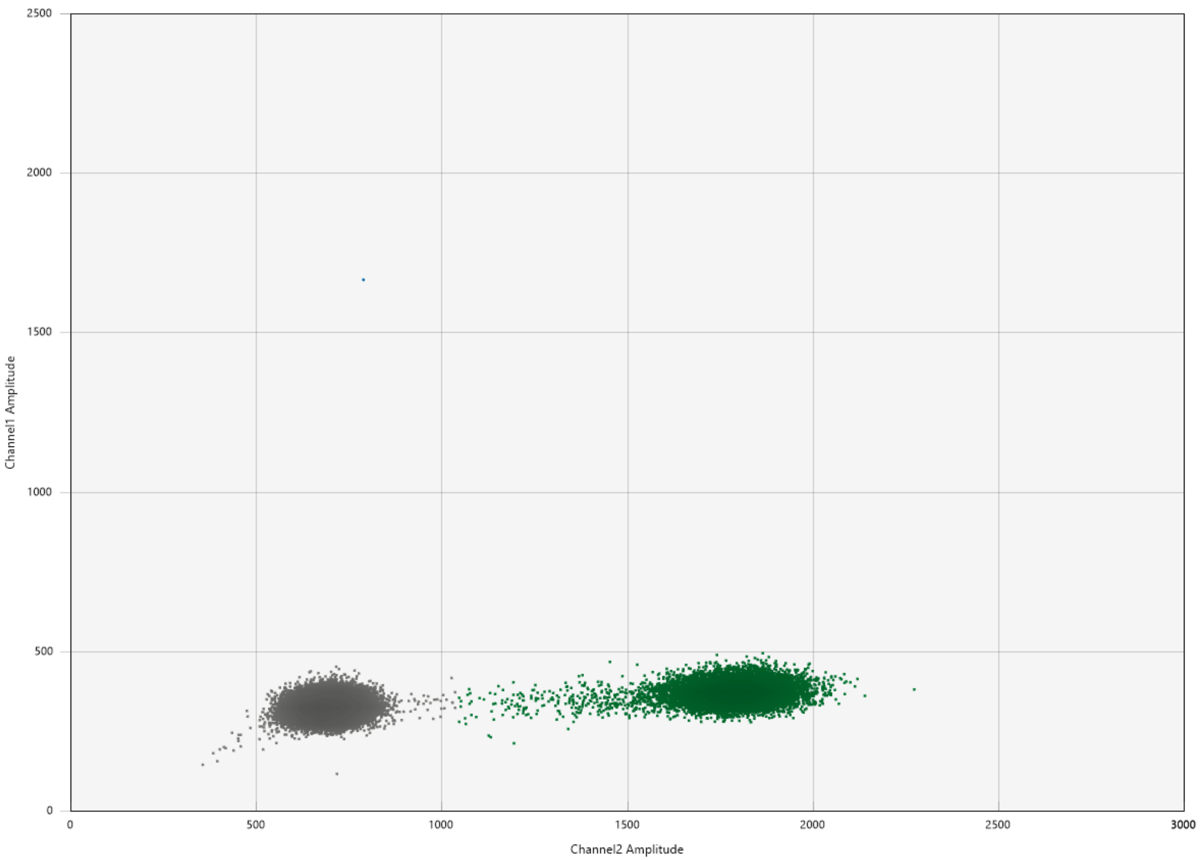

NK-Plasma

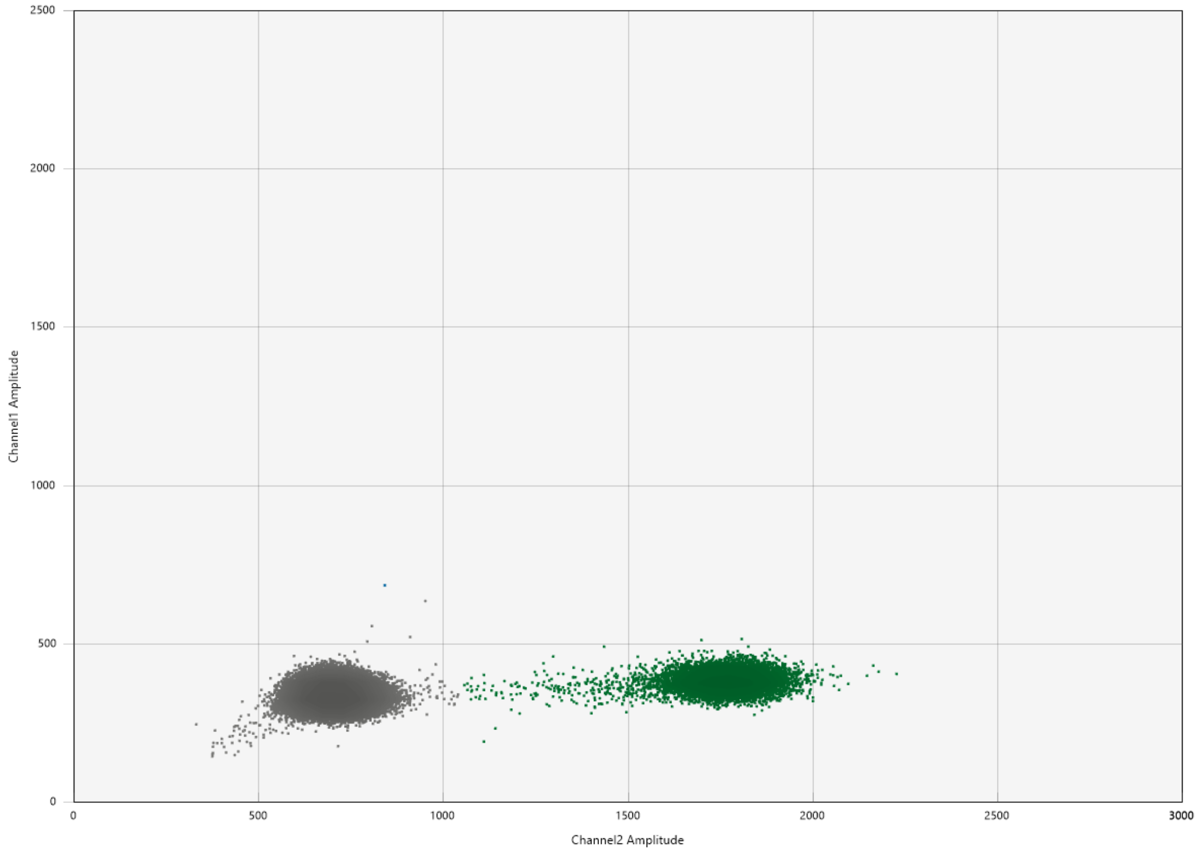

2500
2000
1500
1000
500
0
Channel1 Amplitude
0
500
1000
1500
2000
2500
3000
Channel2 Amplitude

Post-surgery

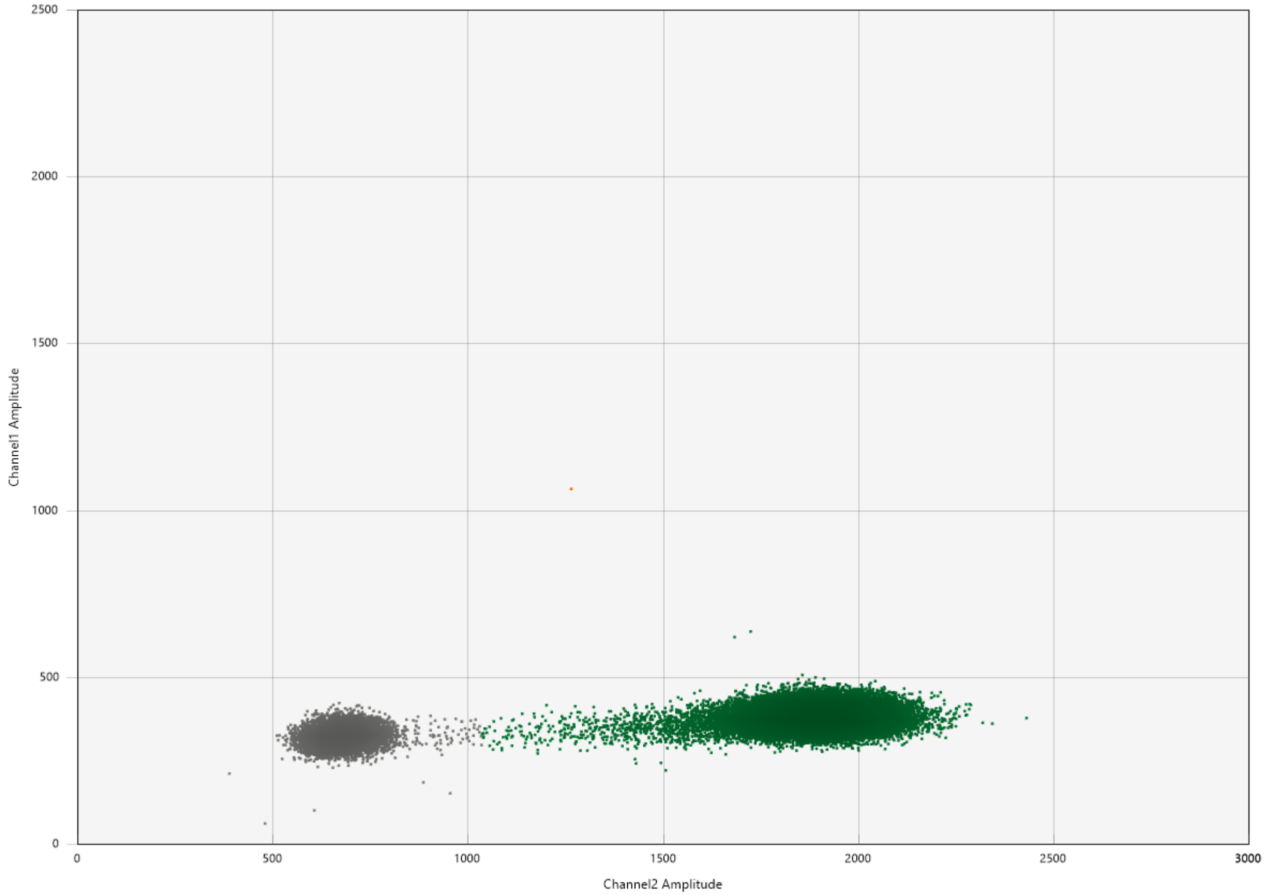

Post-surgery diluted

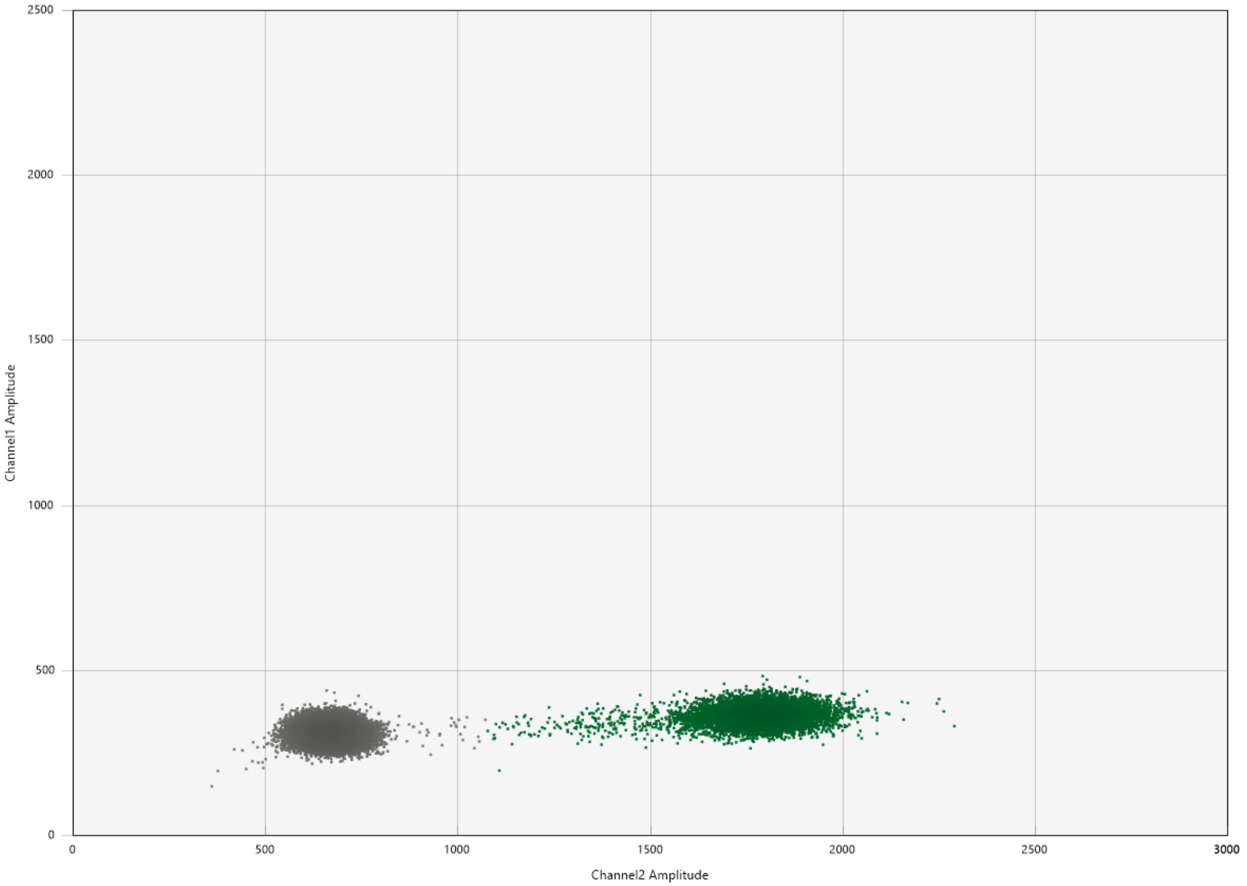

Concentration plots

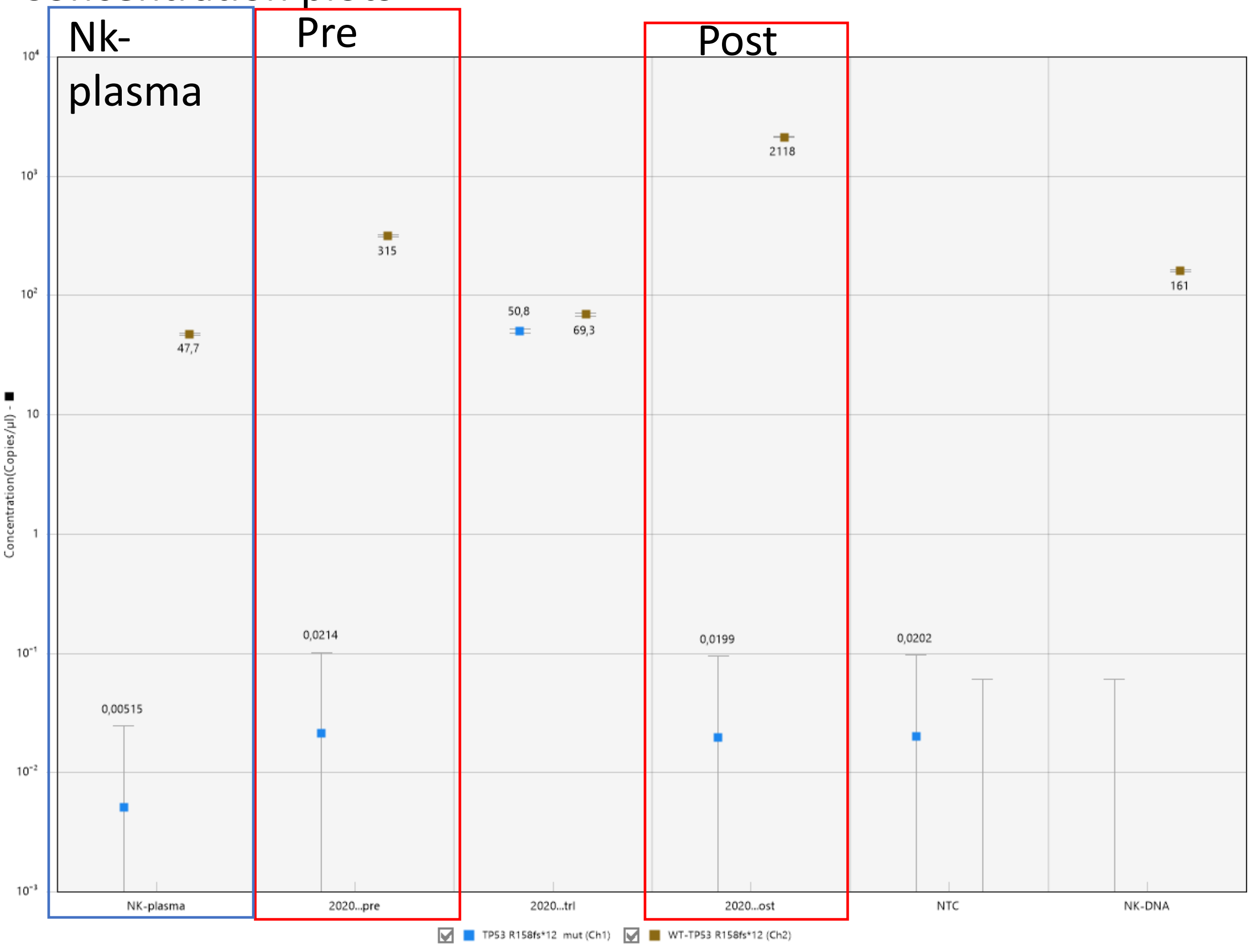

Post-surgery diluted

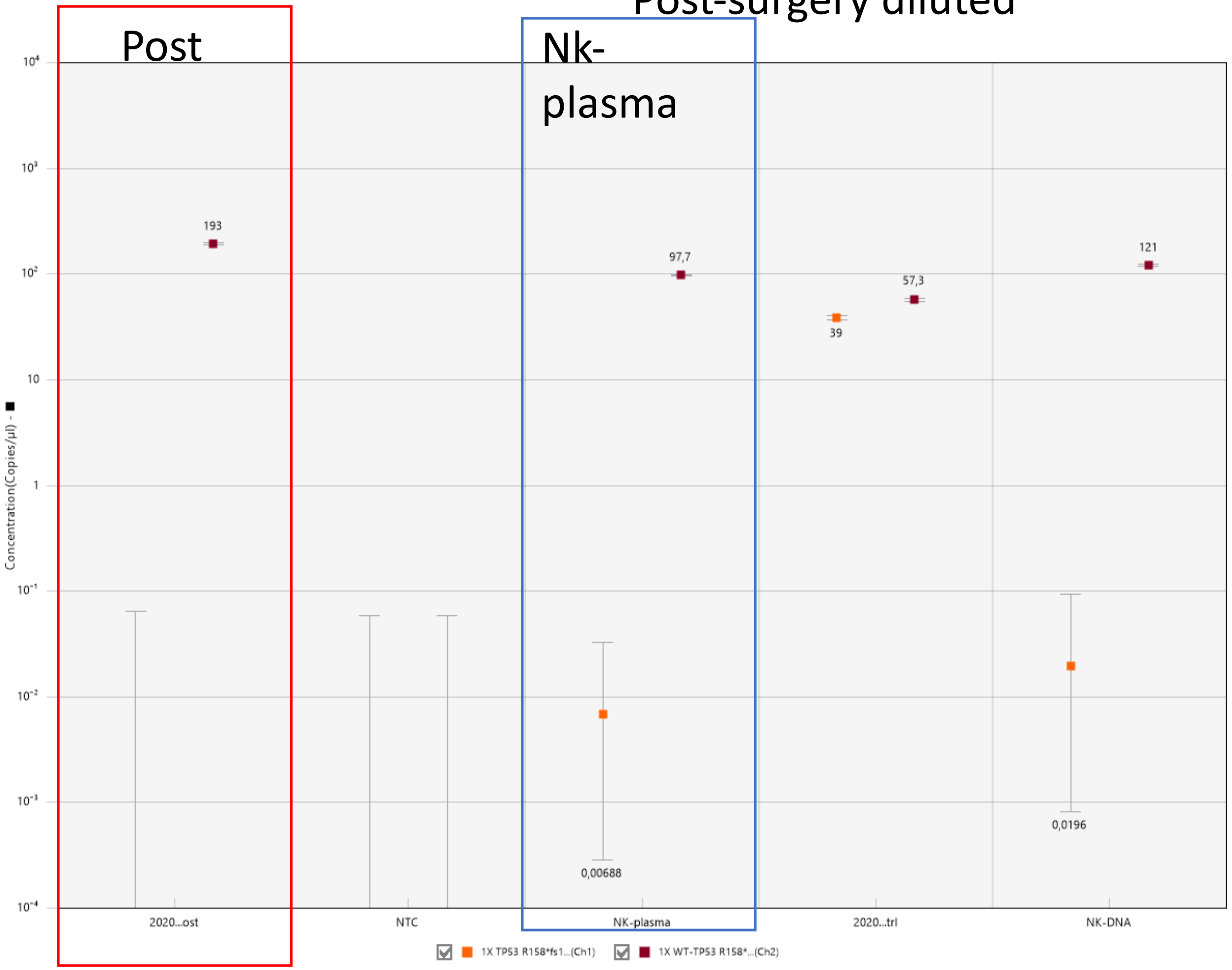

Pre, post

Calling – Pre-op- negative for –TP53 R158fs*- did not met droplet criteria and overlap

Post-op and diluted- negative for TP53 R158fs*

P32 -Final

Gene target : TP53 p.R248W, c.742C>T and TP53,p.?, c.673-2A>G

Assays: 1X TP53 p.R248W mut (FAM) and1X TP53 WT p.R248W (HEX)

1X TP53 p.R248W mut

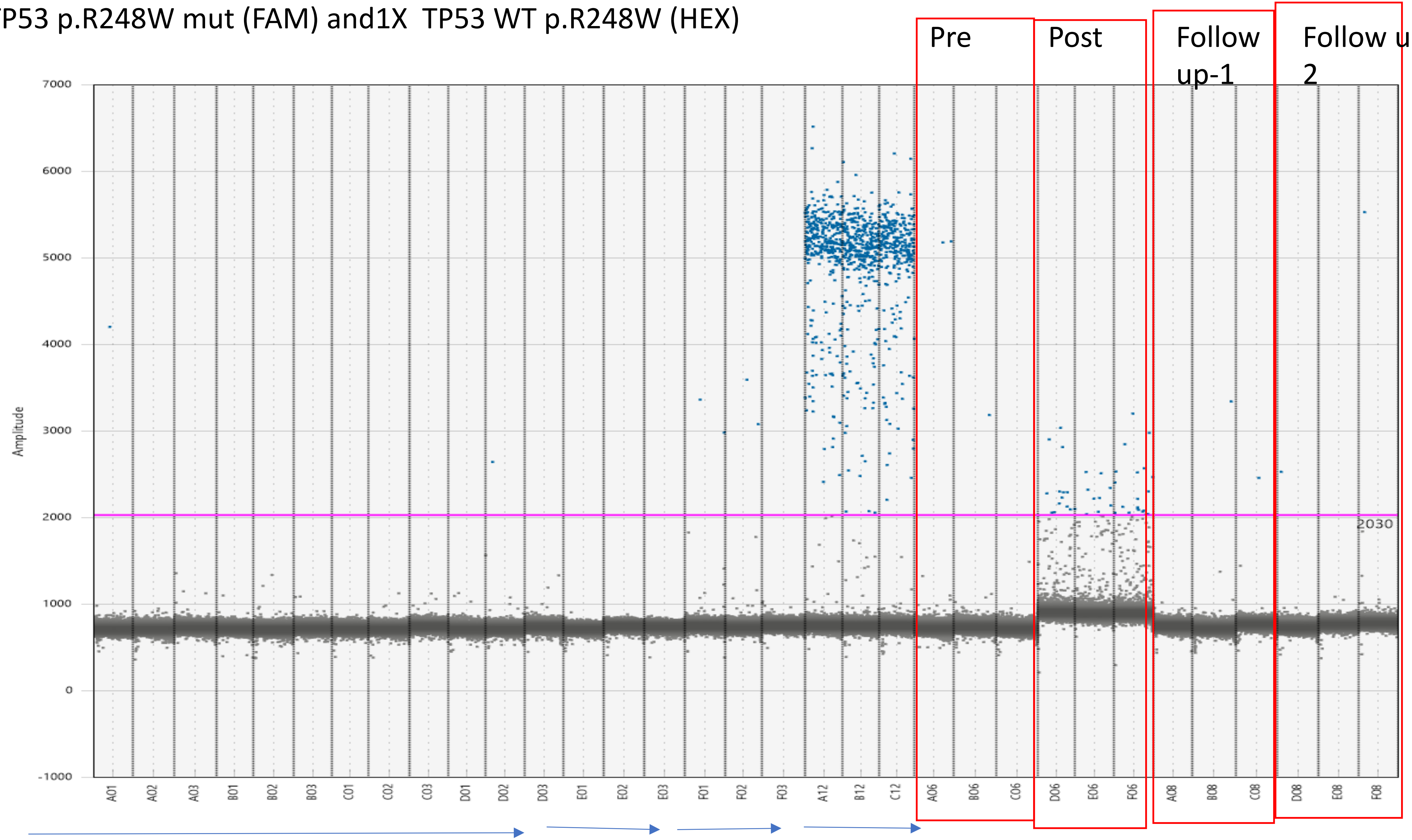

NK-Plasma NTC NK-DNA Pos ctrl

P32 - Final-WT

Assays: 1X TP53 WT p.R248W (HEX)

Gene target : TP53 p.R248W, c.742C>T and TP53,p.?, c.673-2A>G

1X TP53 p.R248W mut

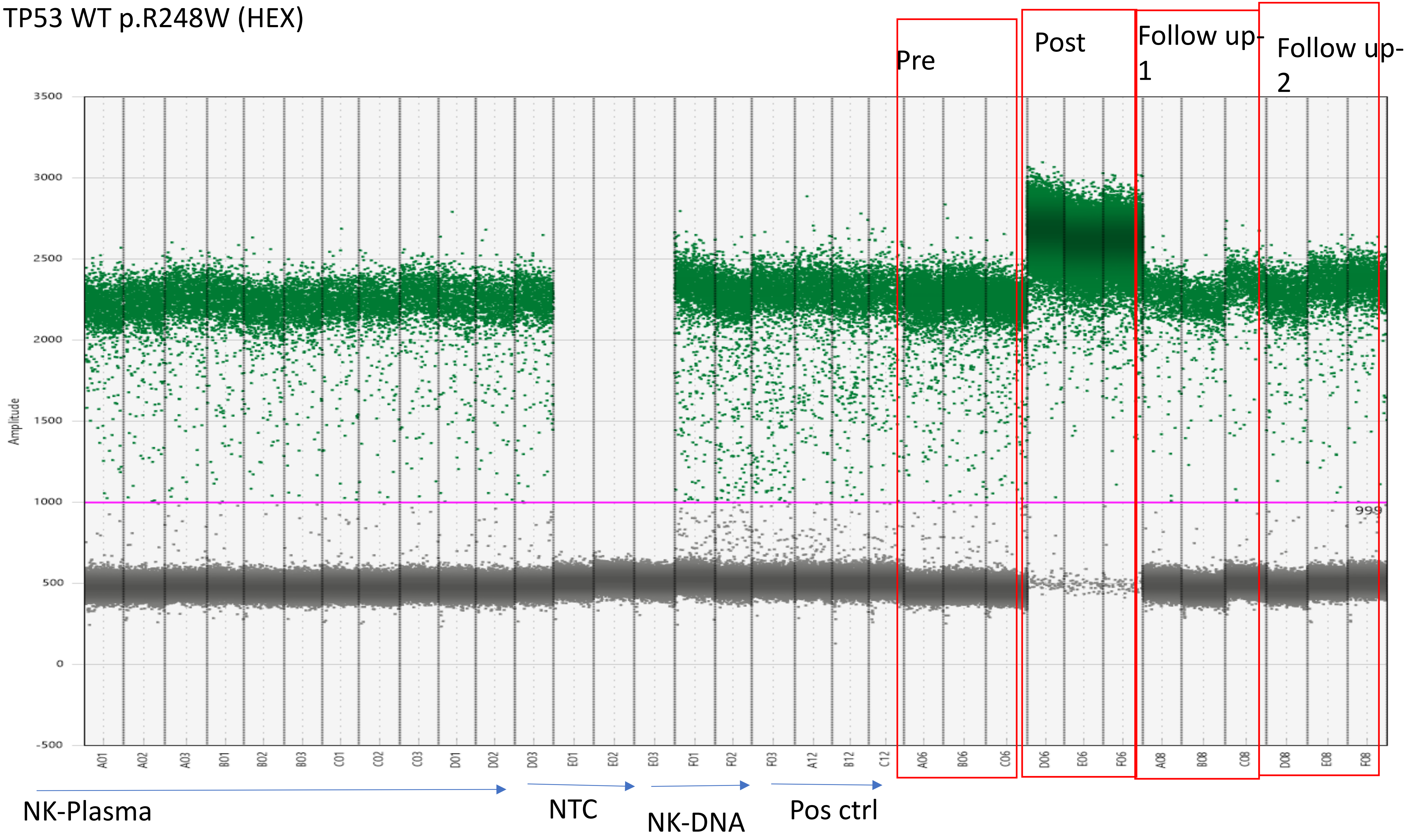

P32 -Final diluted

Gene target : TP53 p.R248W, c.742C>T and TP53,p.?, c.673-2A>G

Assays: 1X TP53 p.R248W mut (FAM) and1X TP53 WT p.R248W (HEX)

1X TP53 p.R248W mut

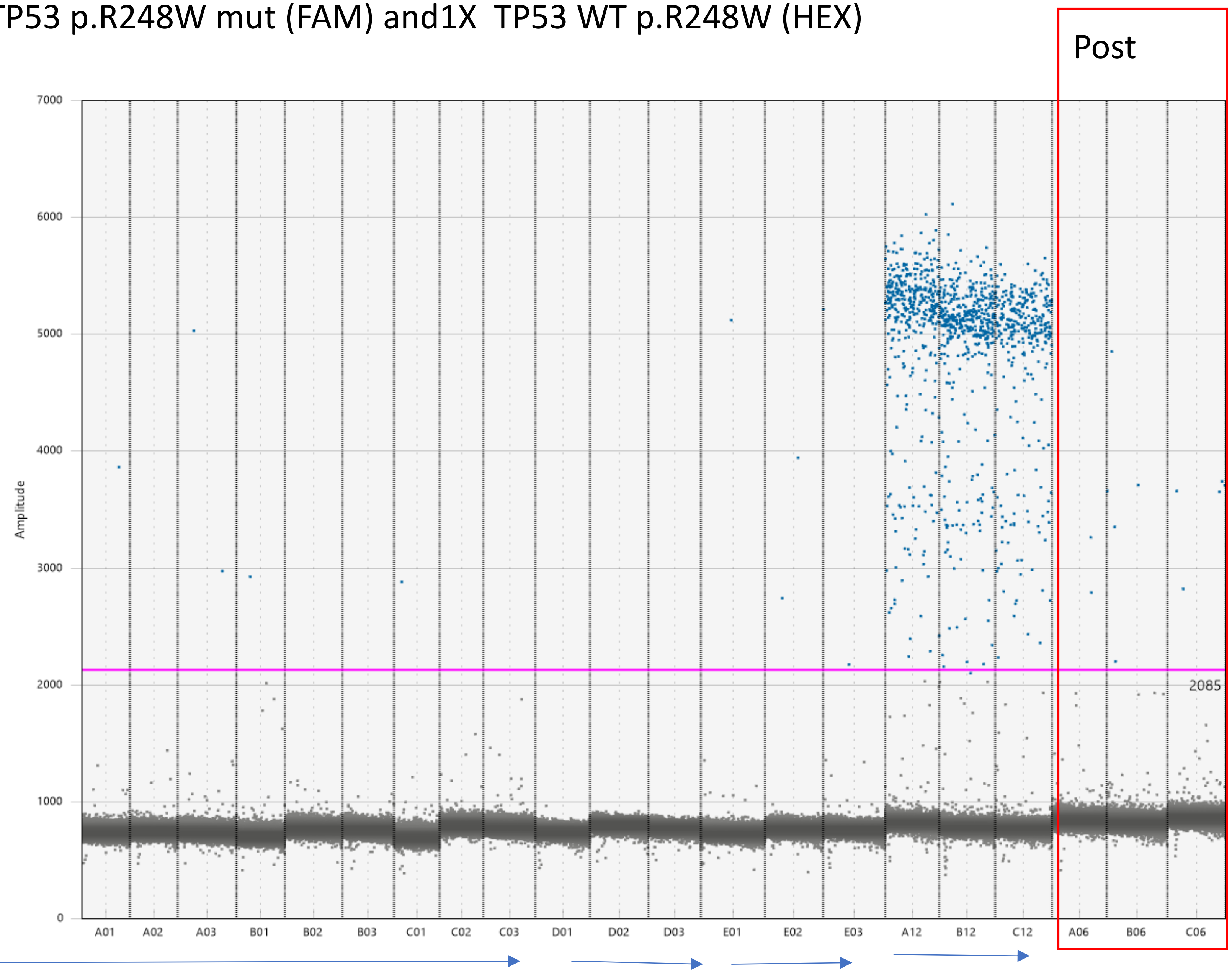

NK-Plasma

NTC

NK-DNA

Pos ctrl

P32 -Final-WT

Assays: 1X TP53 WT p.R248W (HEX)

Gene target : TP53 p.R248W, c.742C>T and TP53,p.?, c.673-2A>G

1X TP53 WT p.R248 W

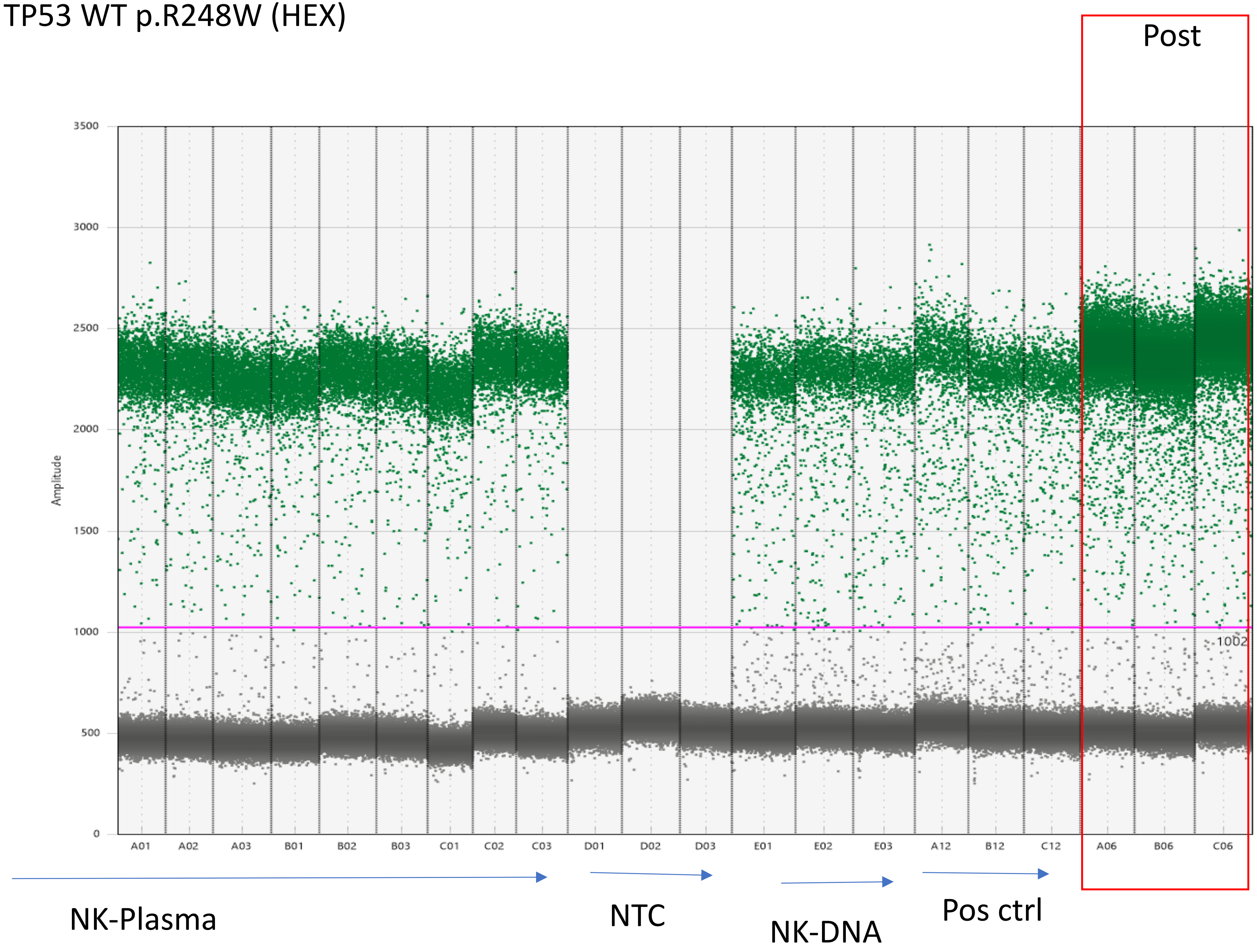

NK-plasma DNA

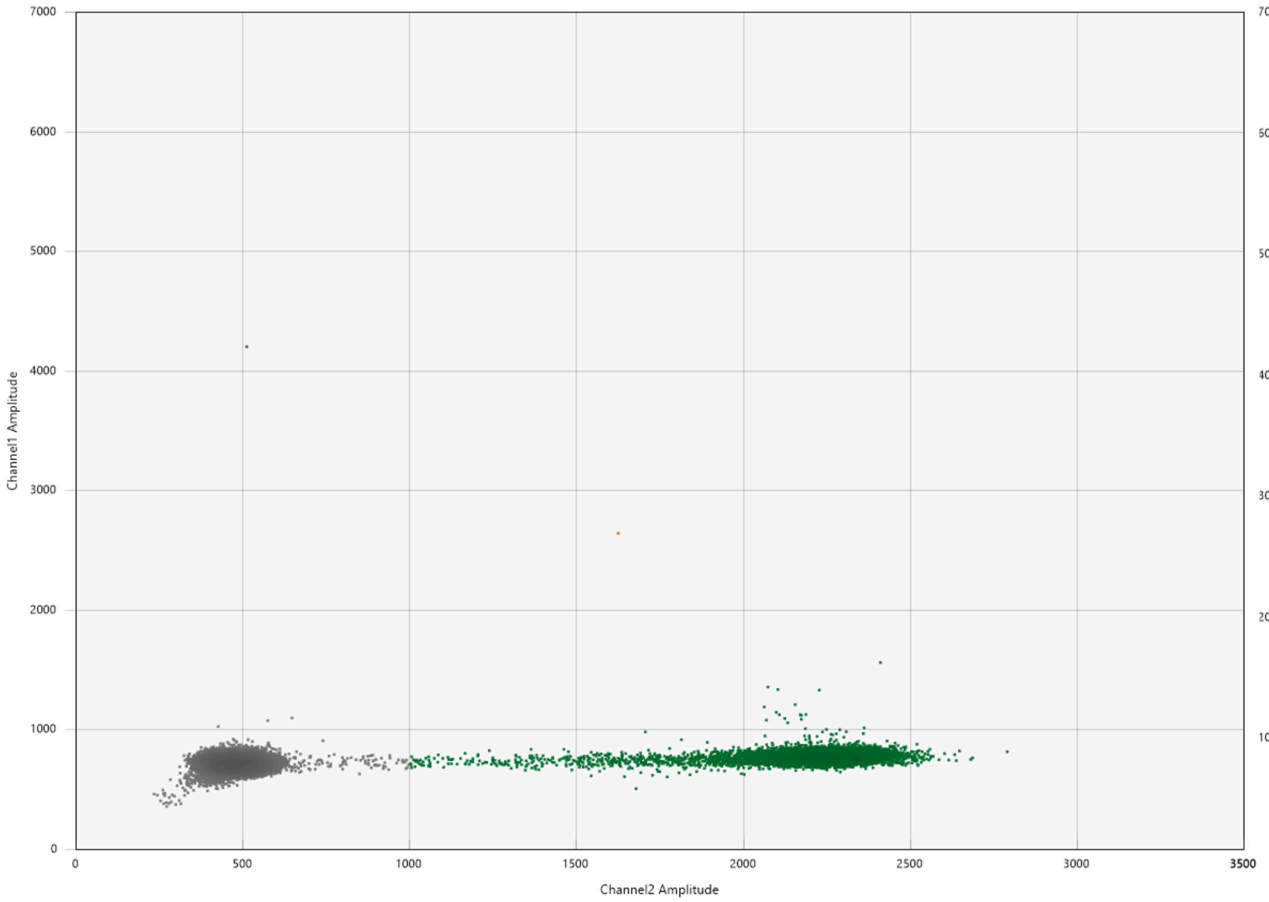

Positive control

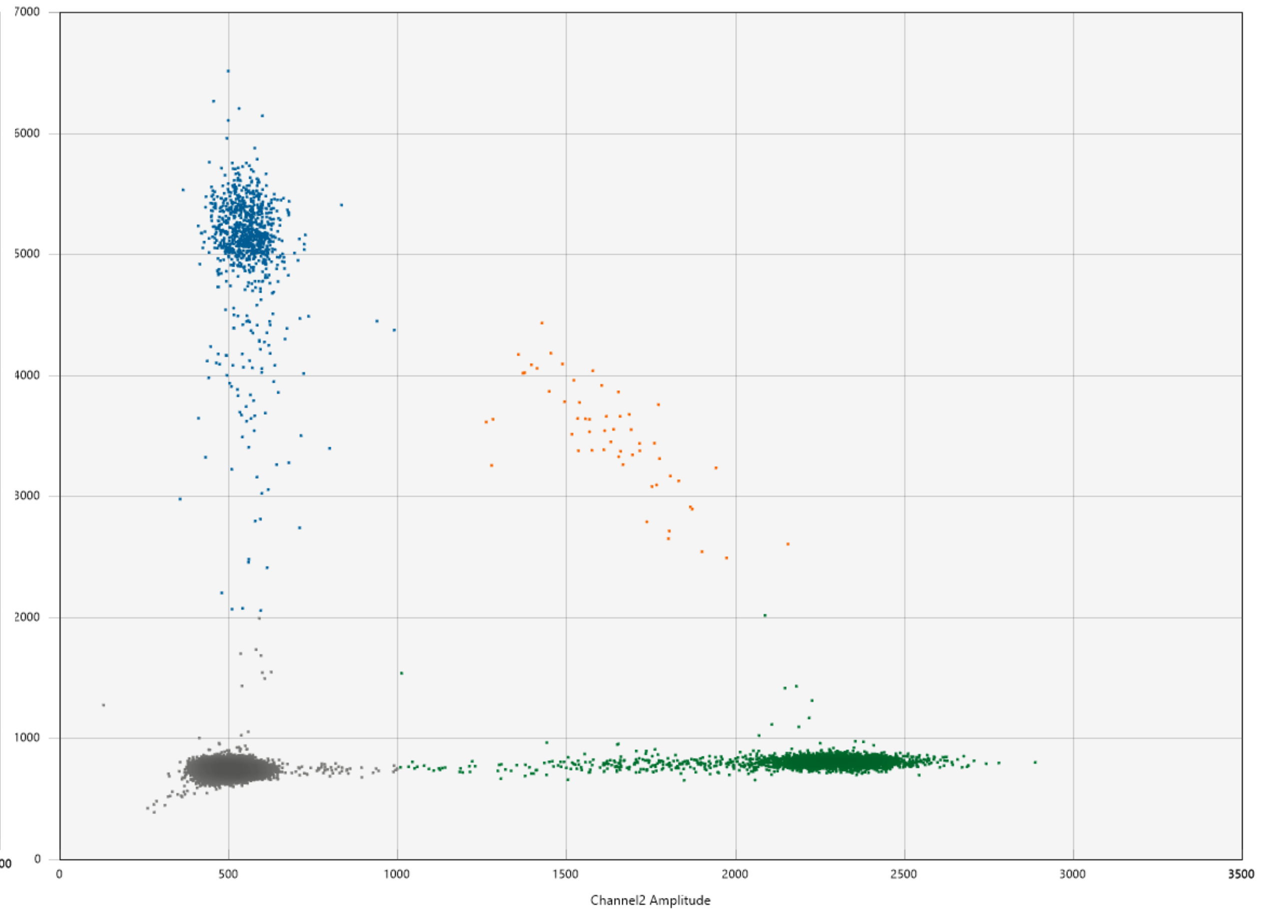

Pre-surgery

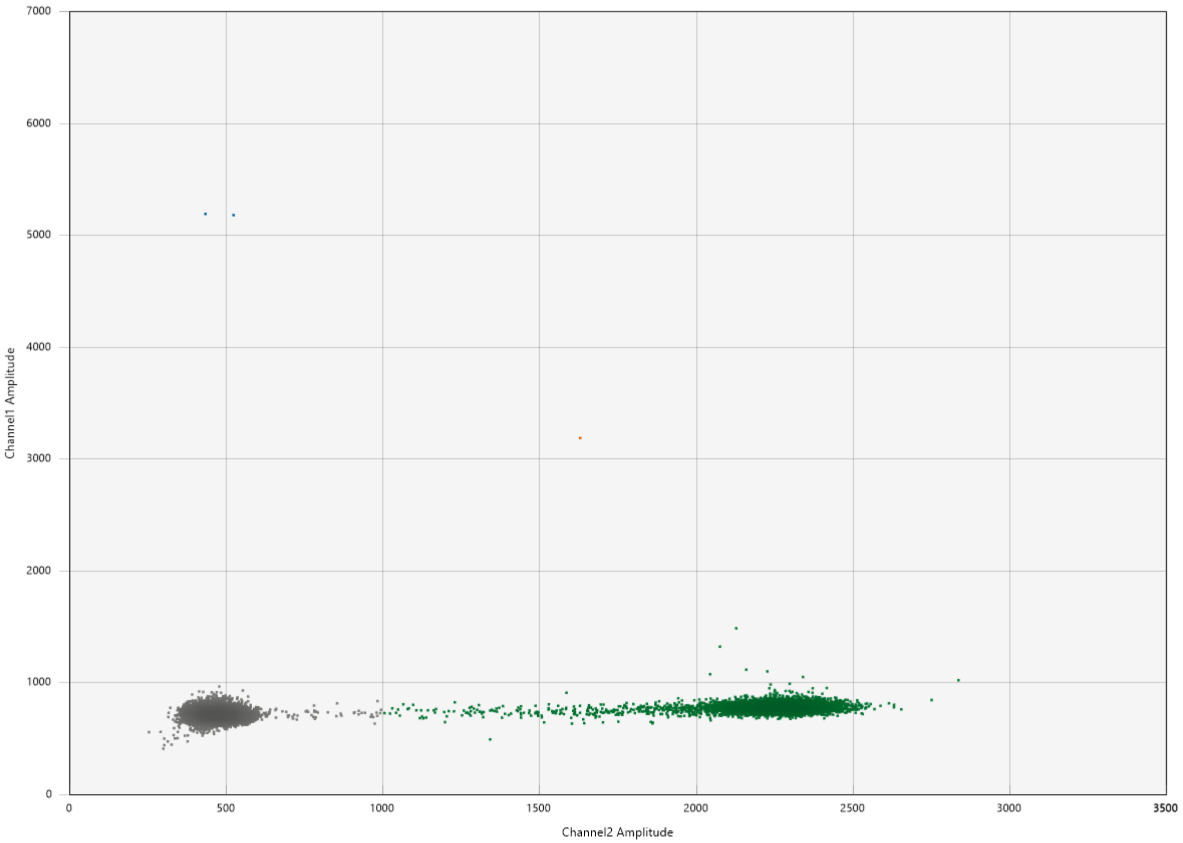

Post-surgery

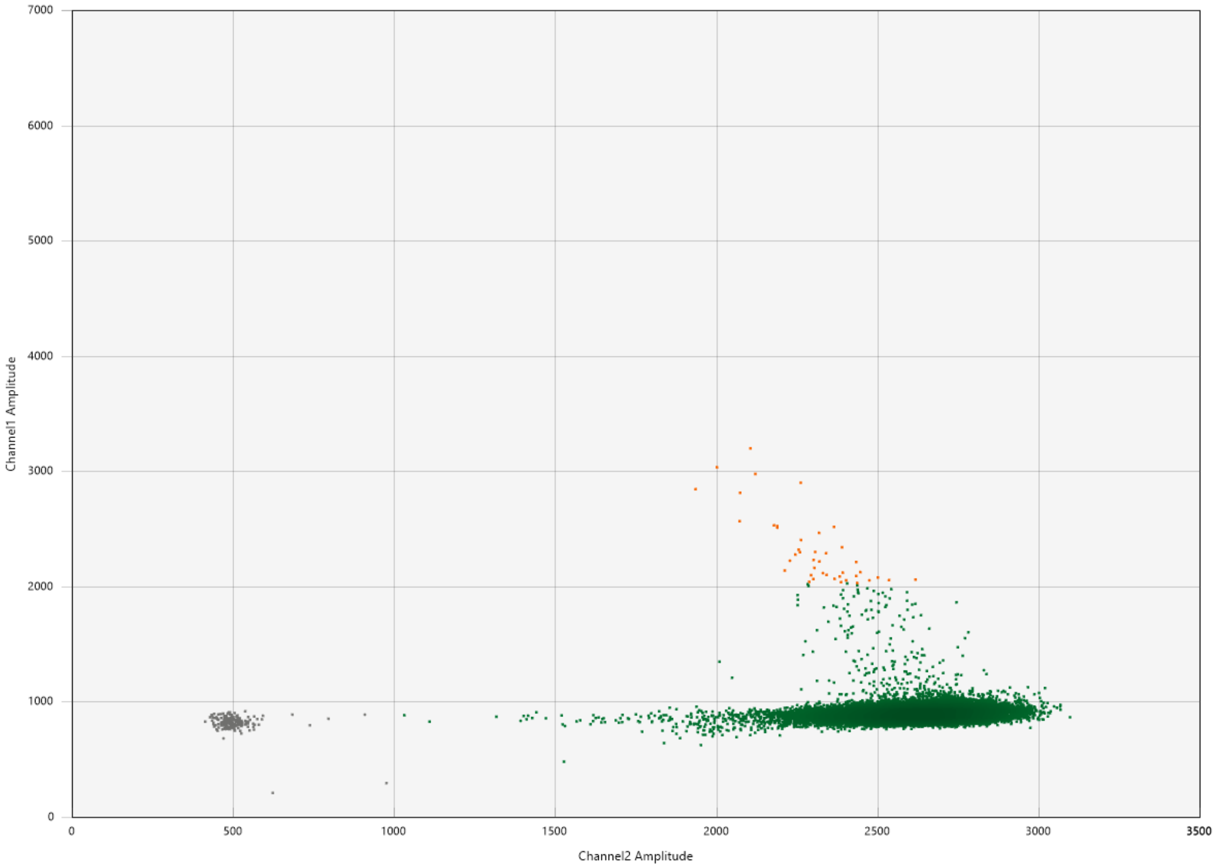

Post-surgery diluted

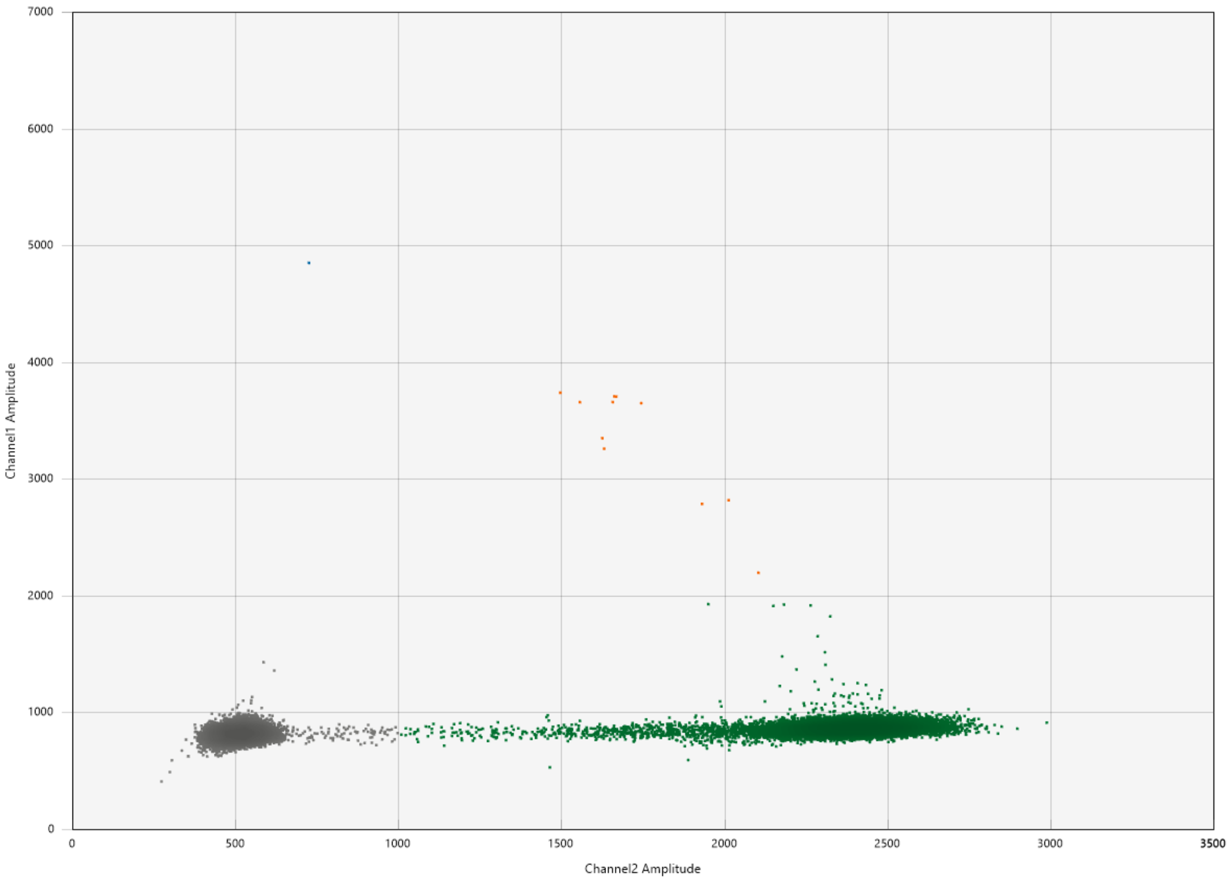

Follow-up 1

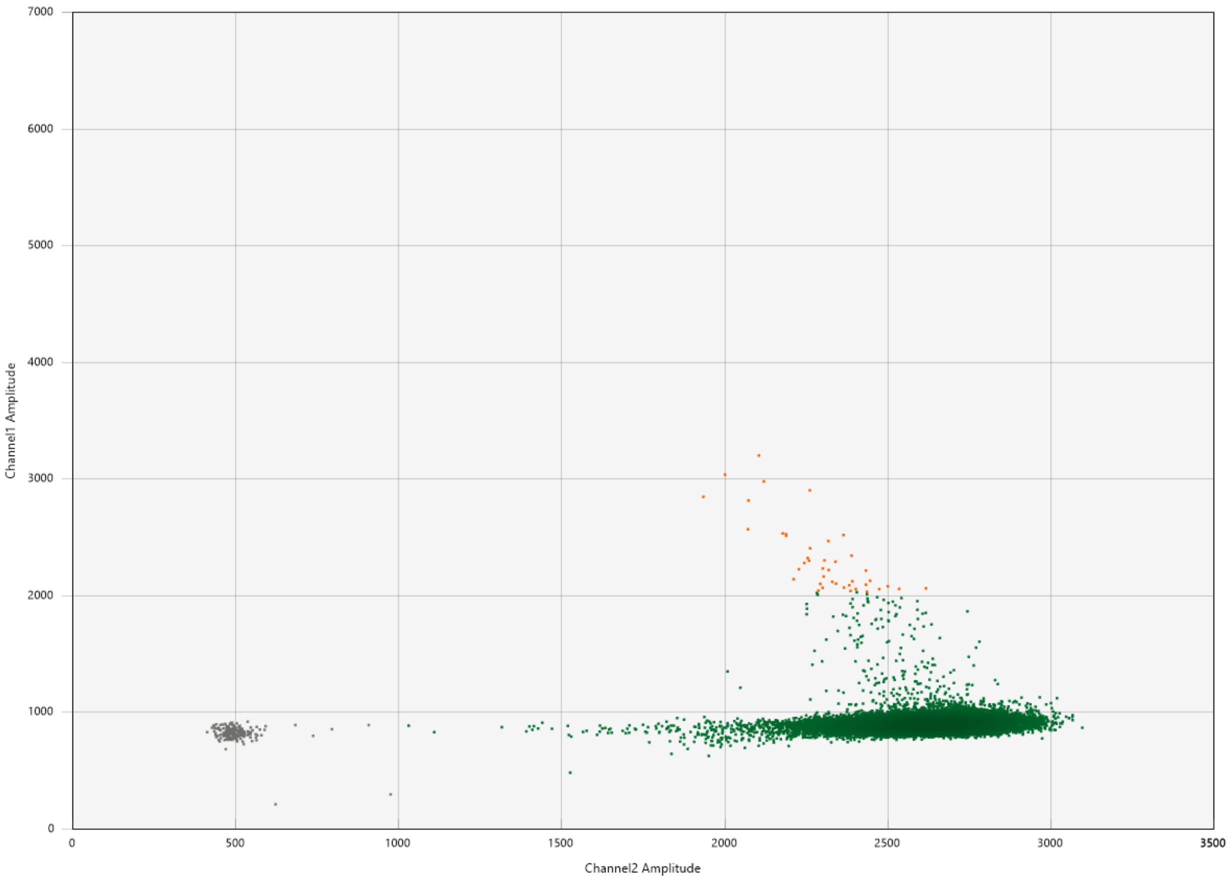

Follow-up 2

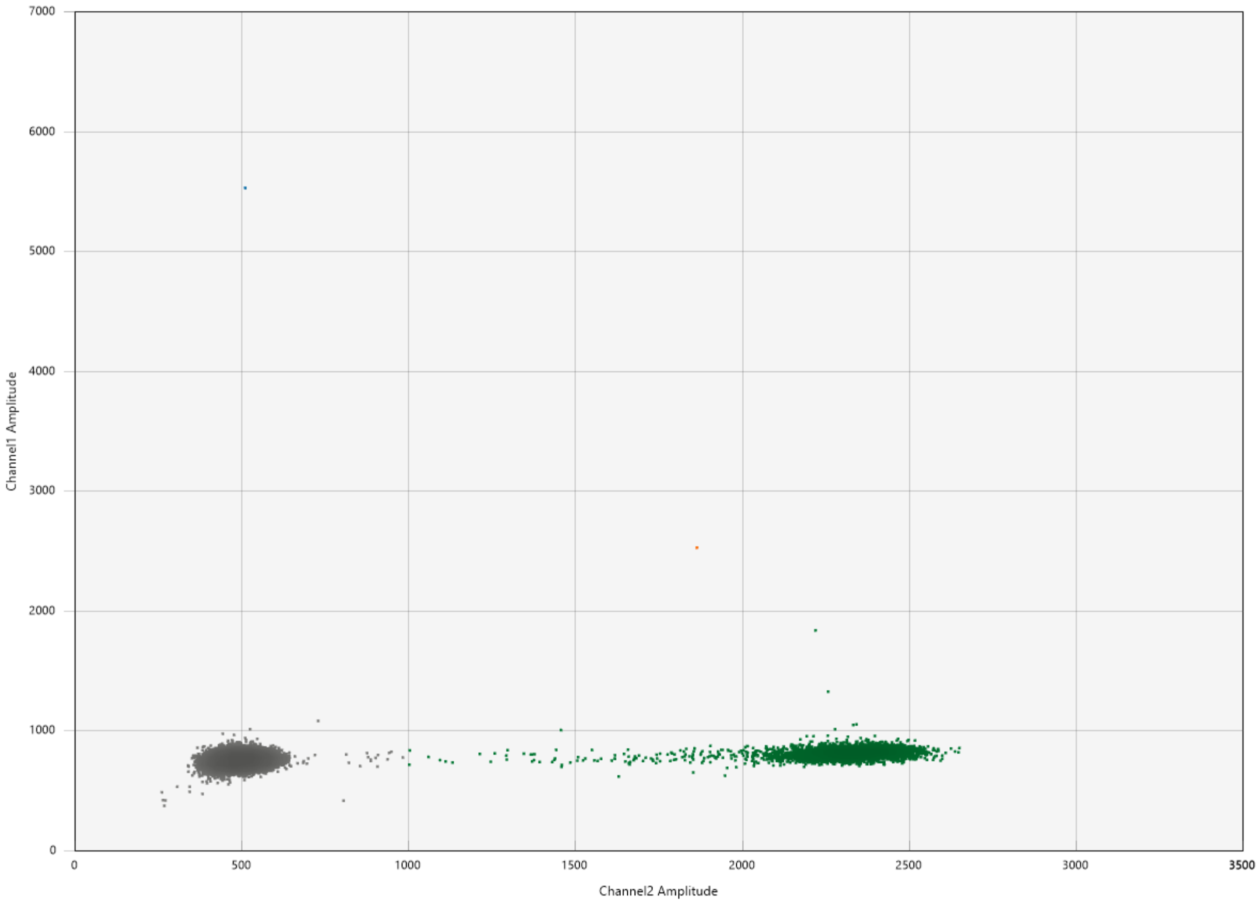

Concentration plots

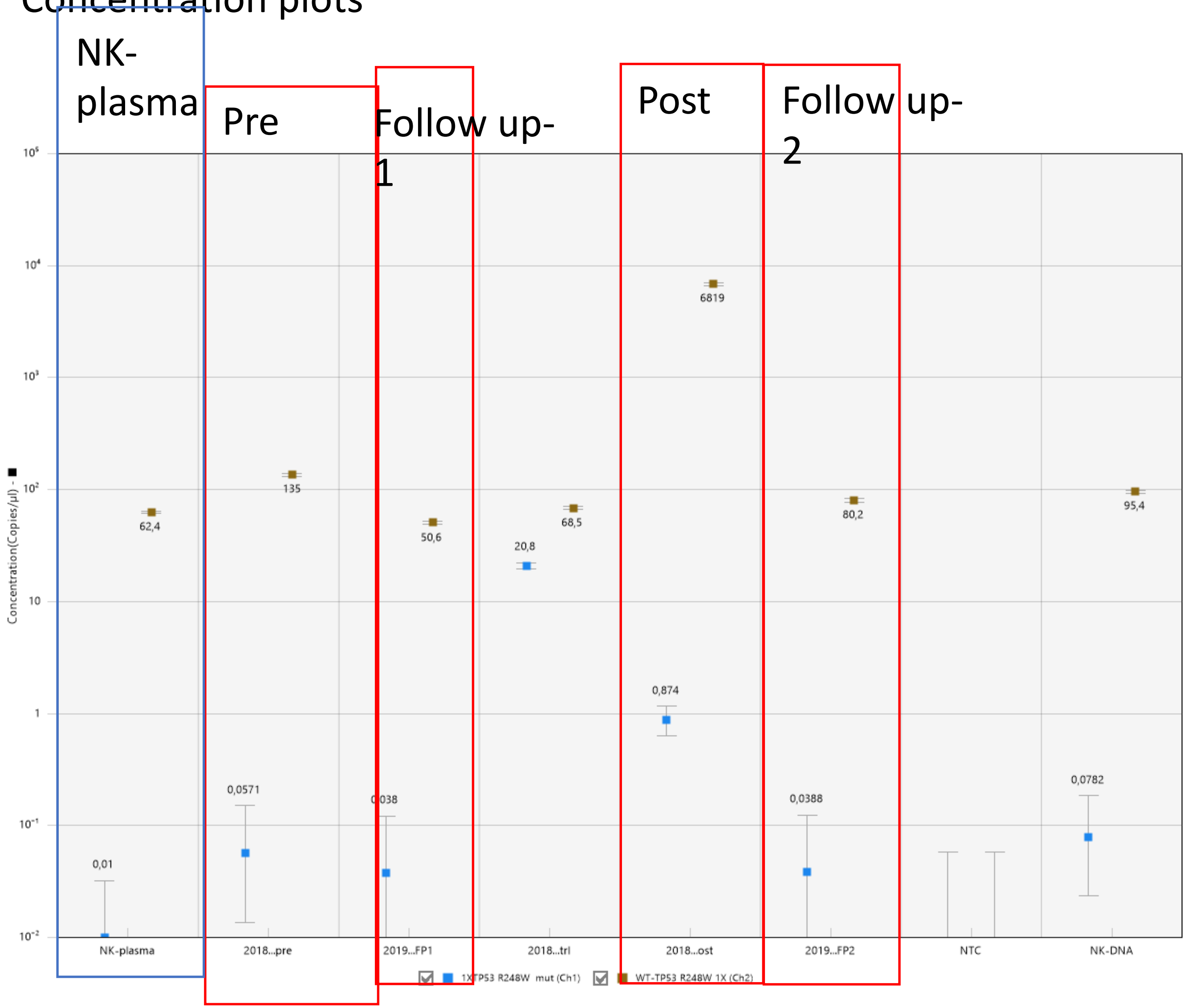

Diluted

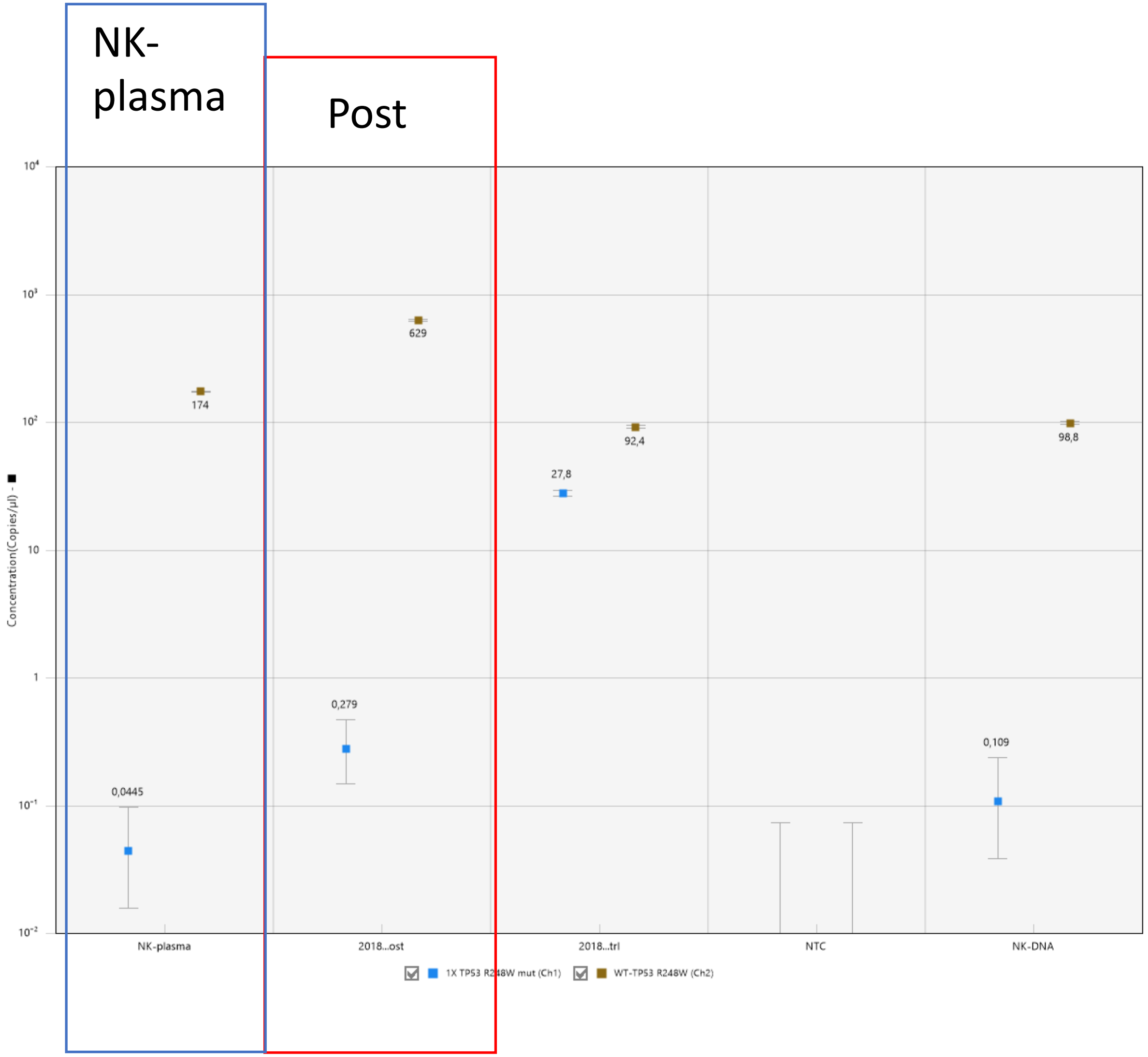

P44 -Final

Gene target : TP53,p.R273H, c.818G>A and KRAS,p.G13D, c.38G>A

Assays: 0.7X TP53,p.R273H mut (FAM) and 1X dark probe for TP53,p.R273H
0.3X KRAS p.G13D mut (FAM) and 1X KRAS WT p.G13D (HEX)

Pre

Post

0.7X TP53, p.R273H mut

0.3X KRAS p.G13D mut

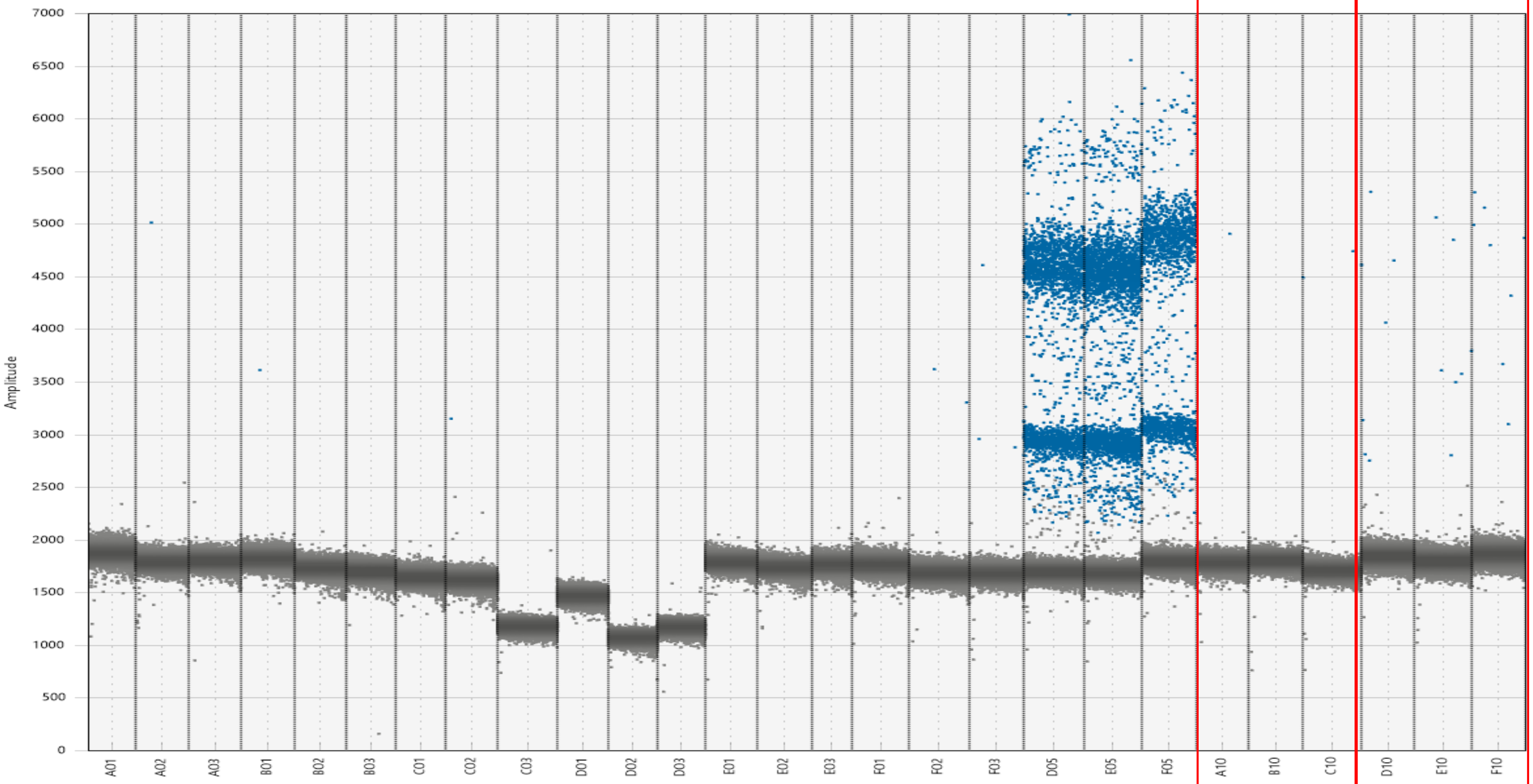

NK-Plasma

NTC

NK-DNA

Pos ctrl

P44 -Final -WT

Assays: 1X dark probe for TP53,p.R273H and 1X KRAS WT p.G13D (HEX)

0.3X KRAS WT p.G13D

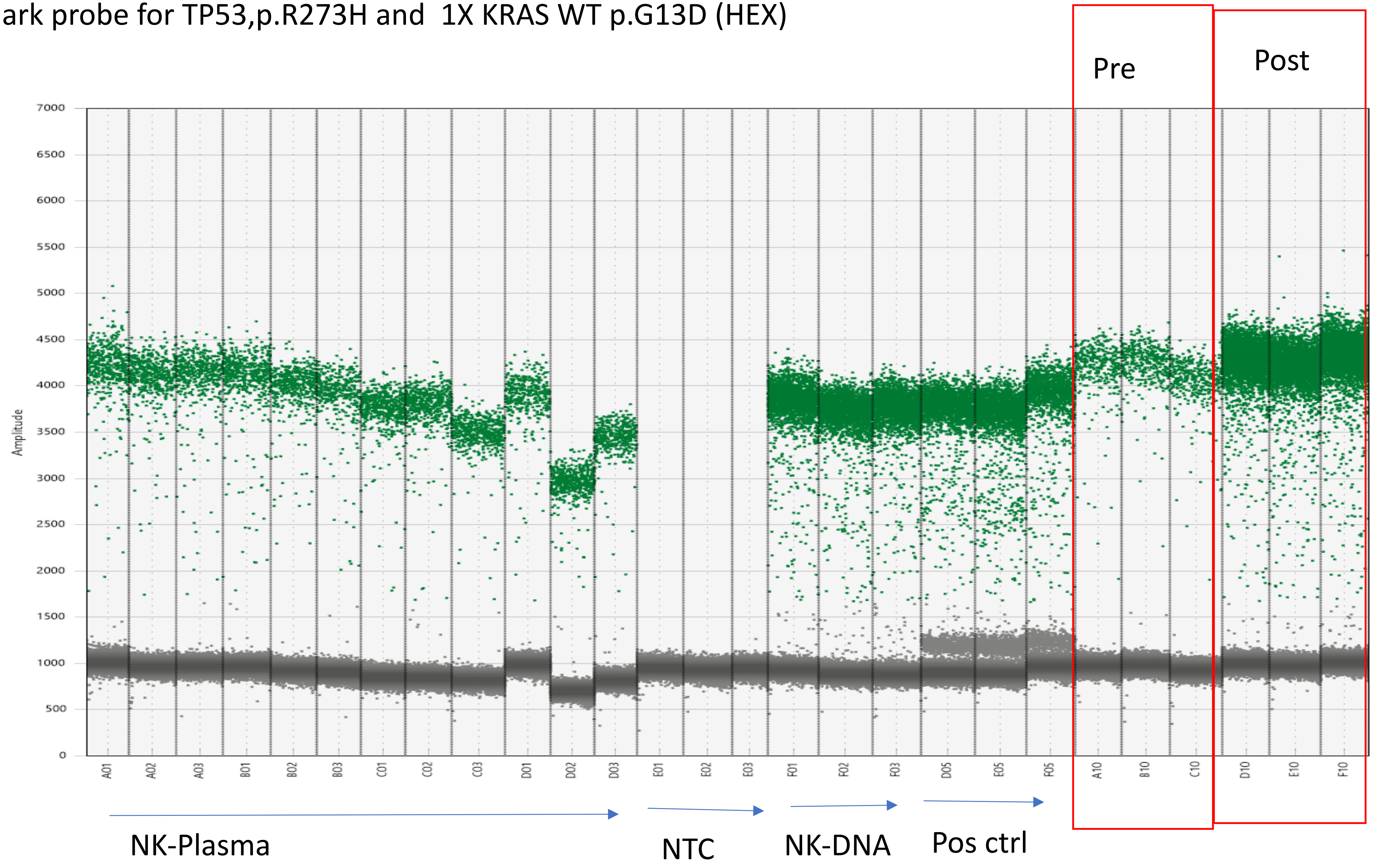

Positive control

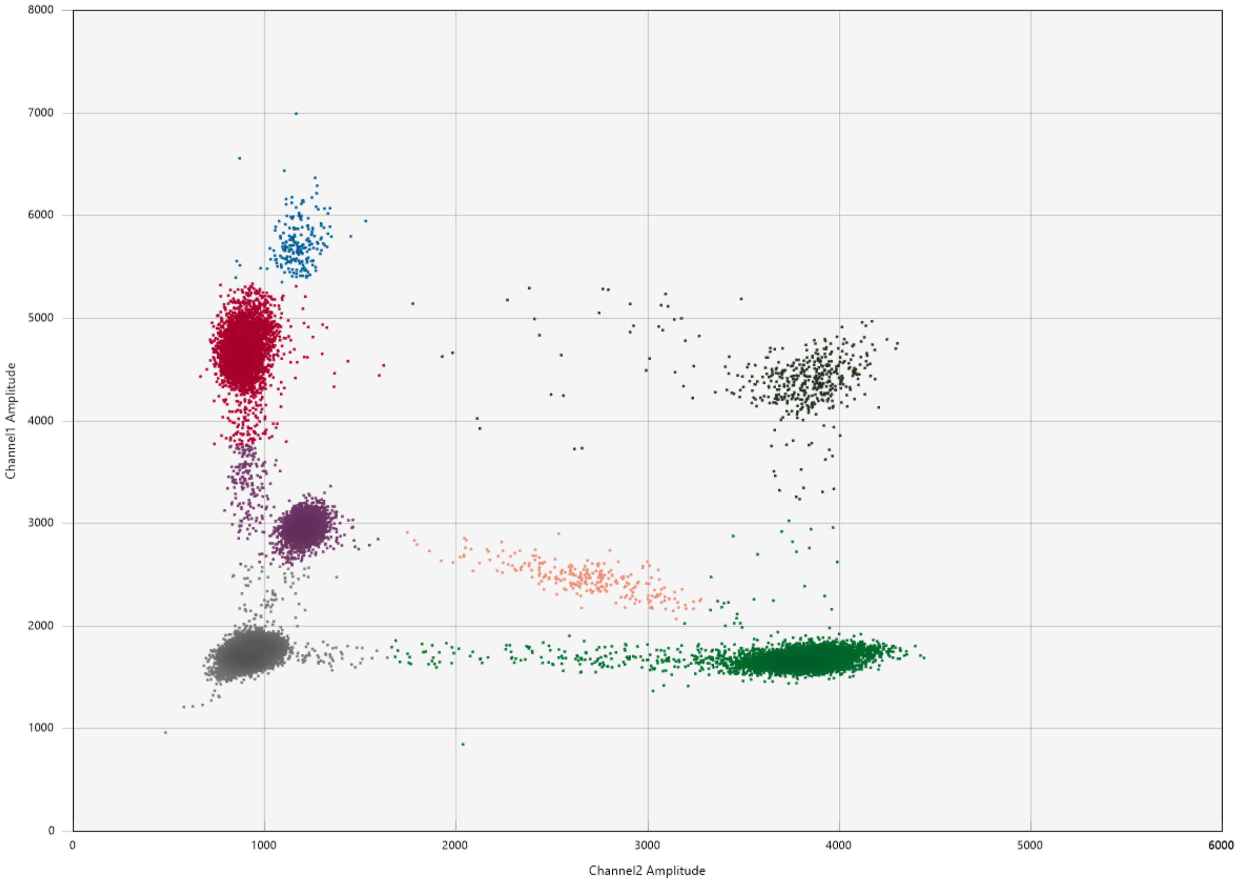

NK-plasma DNA

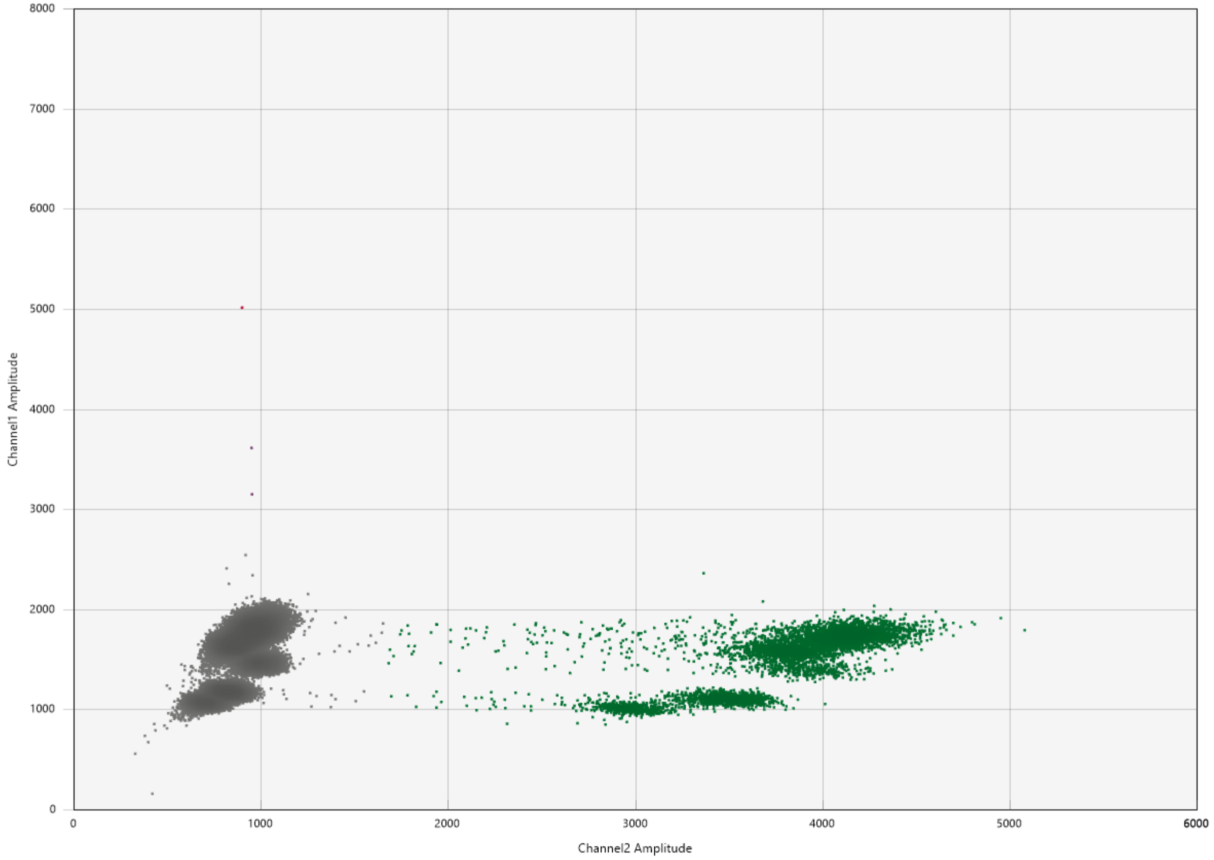

Pre-surgery

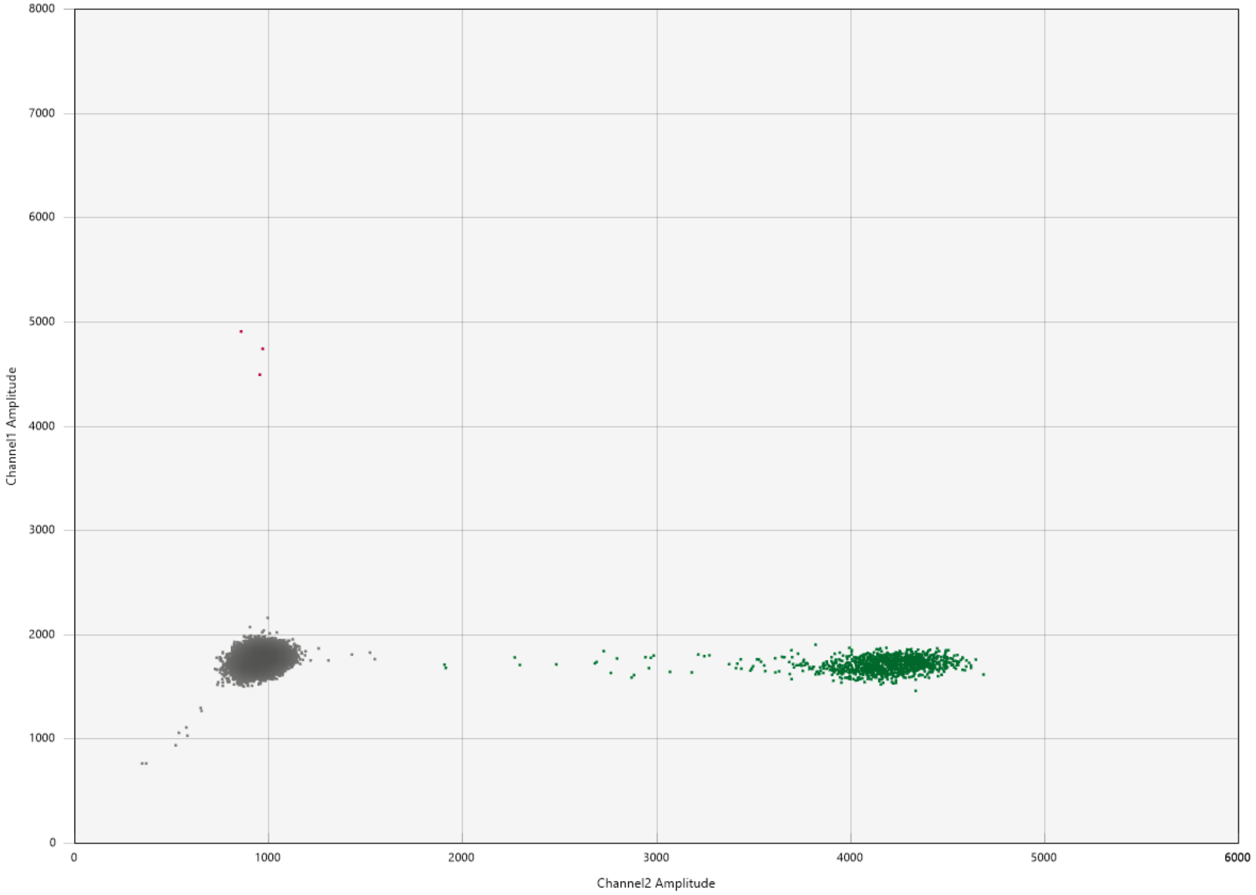

Post-surgery

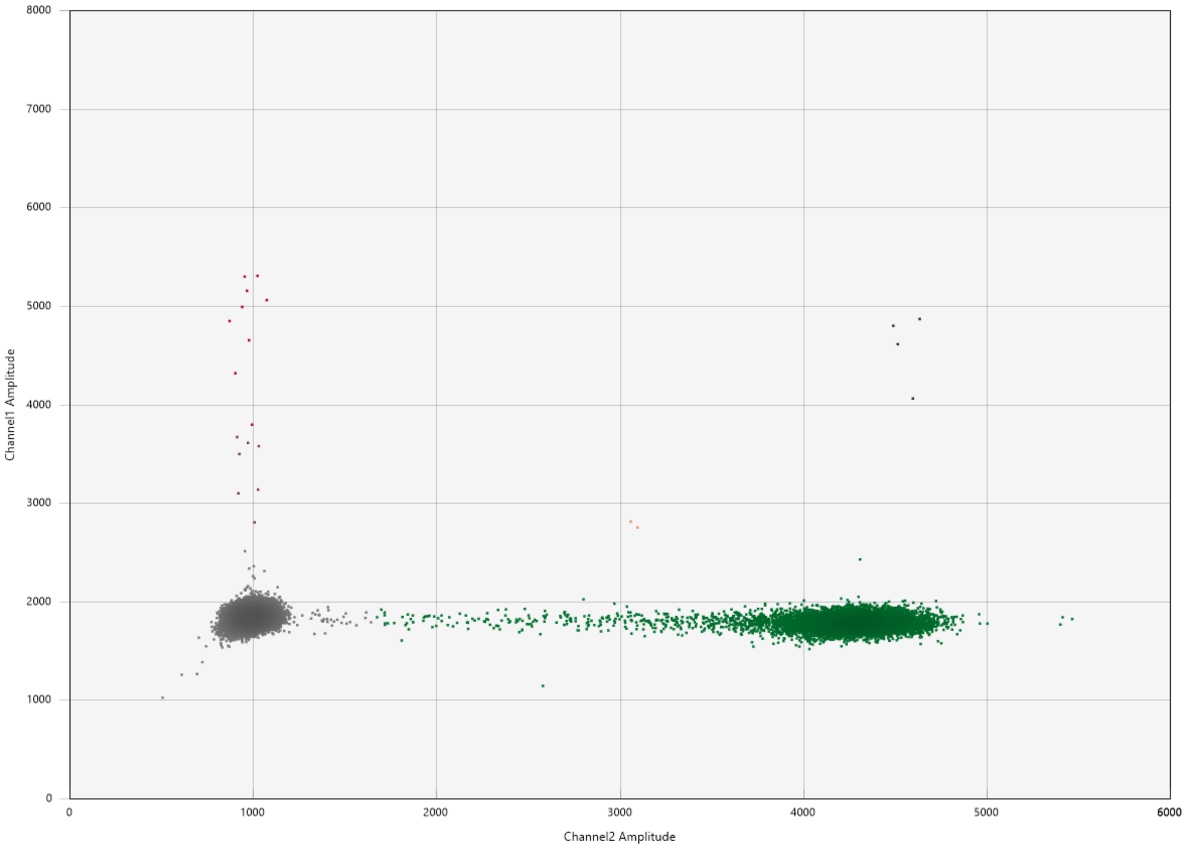

Concentration plots

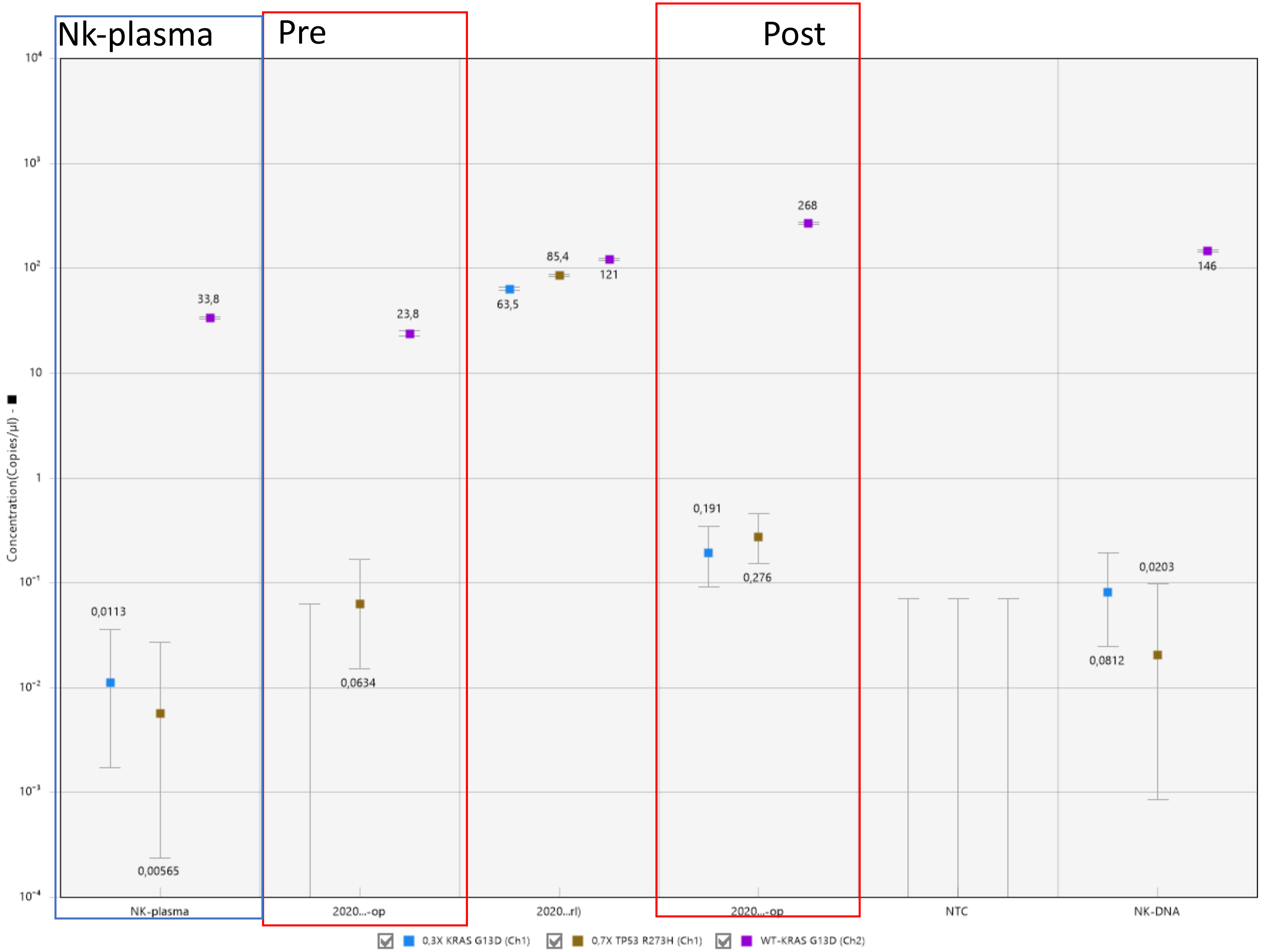

P23 -Final

Gene target : TP53,p.R273H, c.818G>A and KRAS,p.G13D, c.38G>A

Assays: 0.7X TP53,p.R273H mut (FAM) and 1Xdark probe for TP53,p.R273H
0.3X KRAS p.G13D mut (FAM) and1X KRAS WT p.G13D (HEX)

Pre

Post

0.7X TP53,p.
R273H mut

0.3X KRAS
p.G13D mut

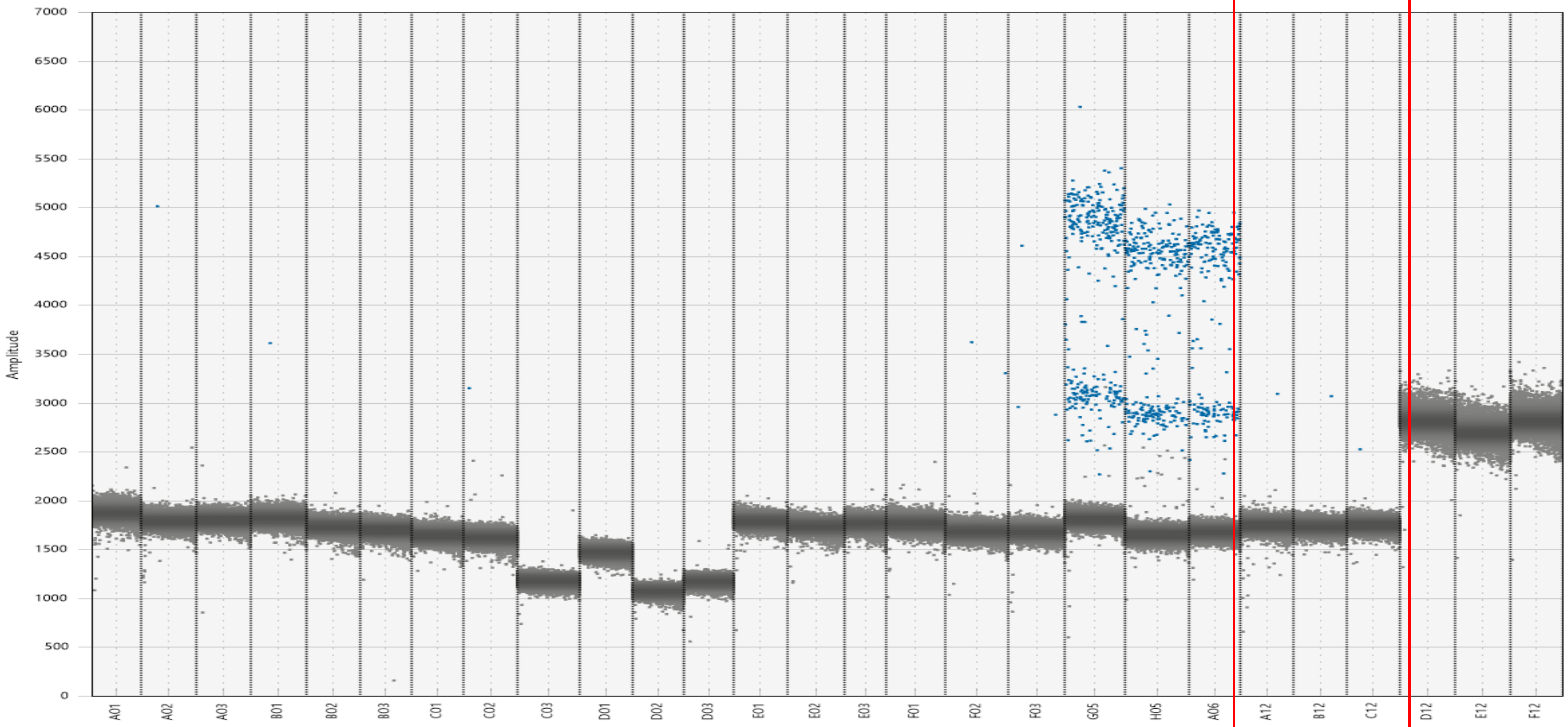

NK-Plasma

NTC

NK-DNA

Pos ctrl

P23 -Final -WT

Gene target : TP53,p.R273H, c.818G>A and KRAS,p.G13D, c.38G>A

Assays: 1Xdark probe for TP53,p.R273H
1X KRAS WT p.G13D (HEX)

0.3X KRAS
WT p.G13D

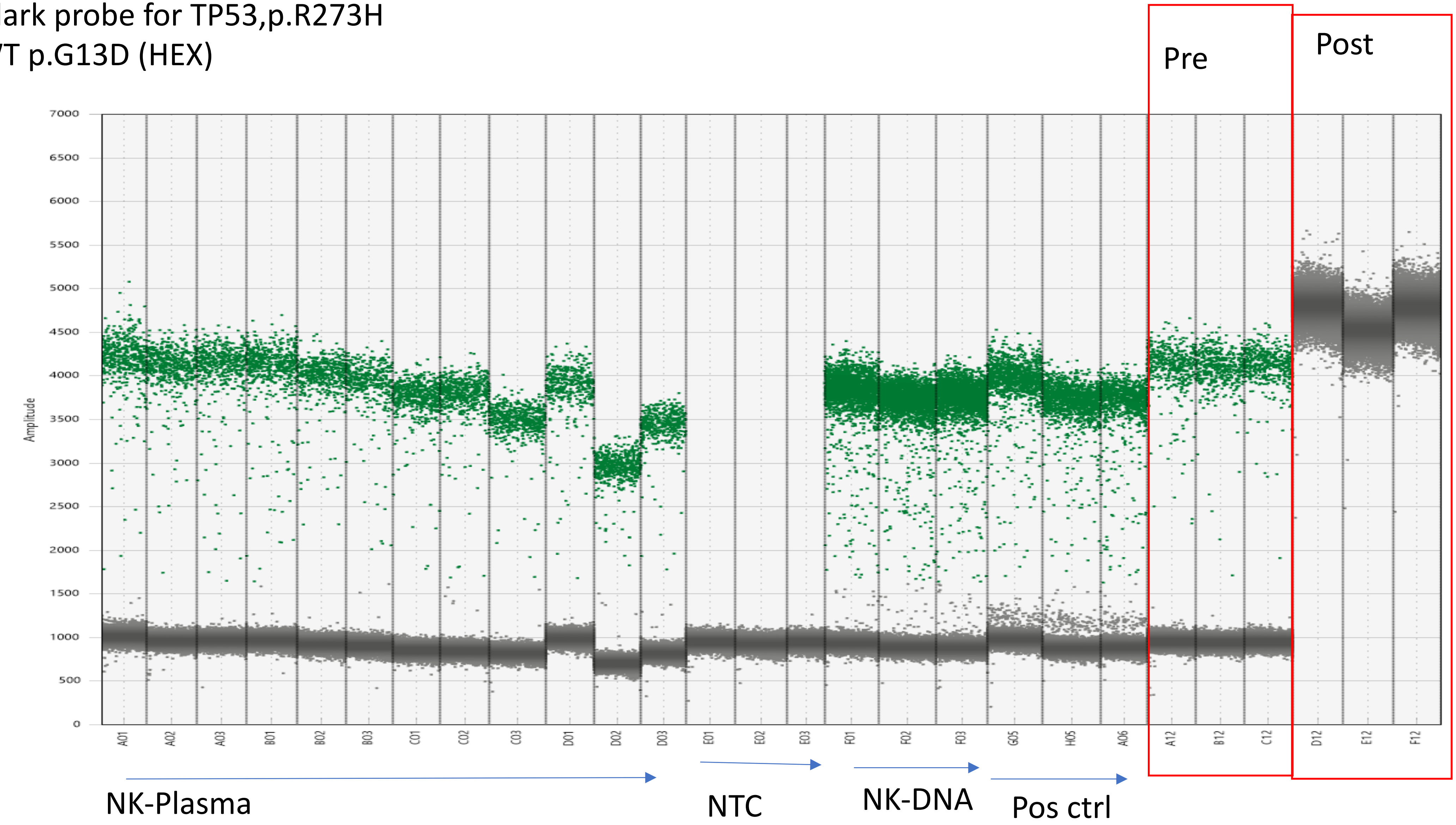

P23 - Final

Gene target : TP53,p.R273H, c.818G>A and KRAS,p.G13D, c.38G>A

Assays: 0.7X TP53,p.R273H mut (FAM) and 1Xdark probe for TP53,p.R273H
0.3X KRAS p.G13D mut (FAM) and1X KRAS WT p.G13D (HEX)

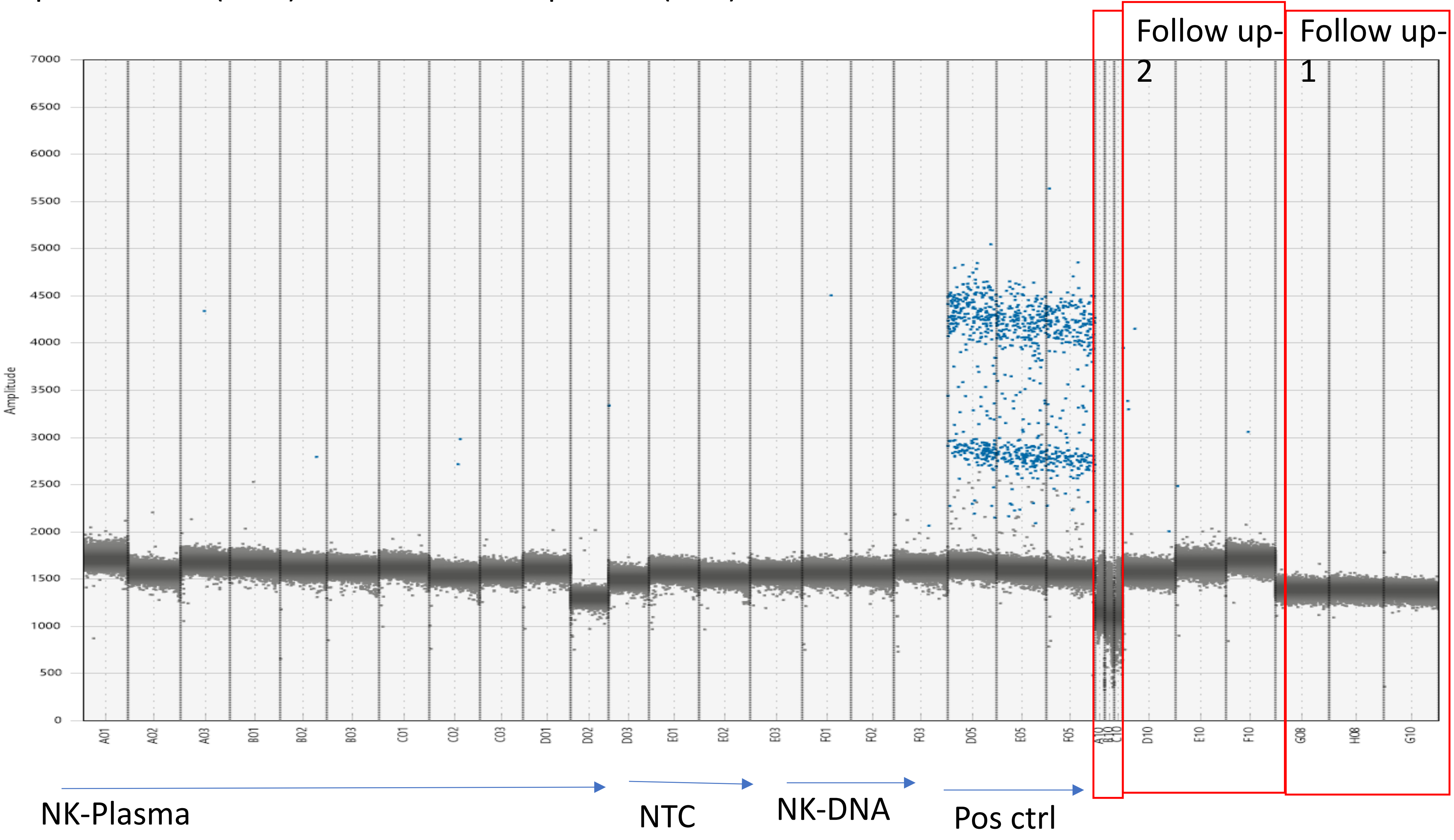

P23 -Final-WT

Gene target : TP53,p.R273H, c.818G>A and KRAS,p.G13D, c.38G>A

Assays: 1Xdark probe for TP53,p.R273H
1X KRAS WT p.G13D (HEX)

0.3X KRAS WT p.G13D

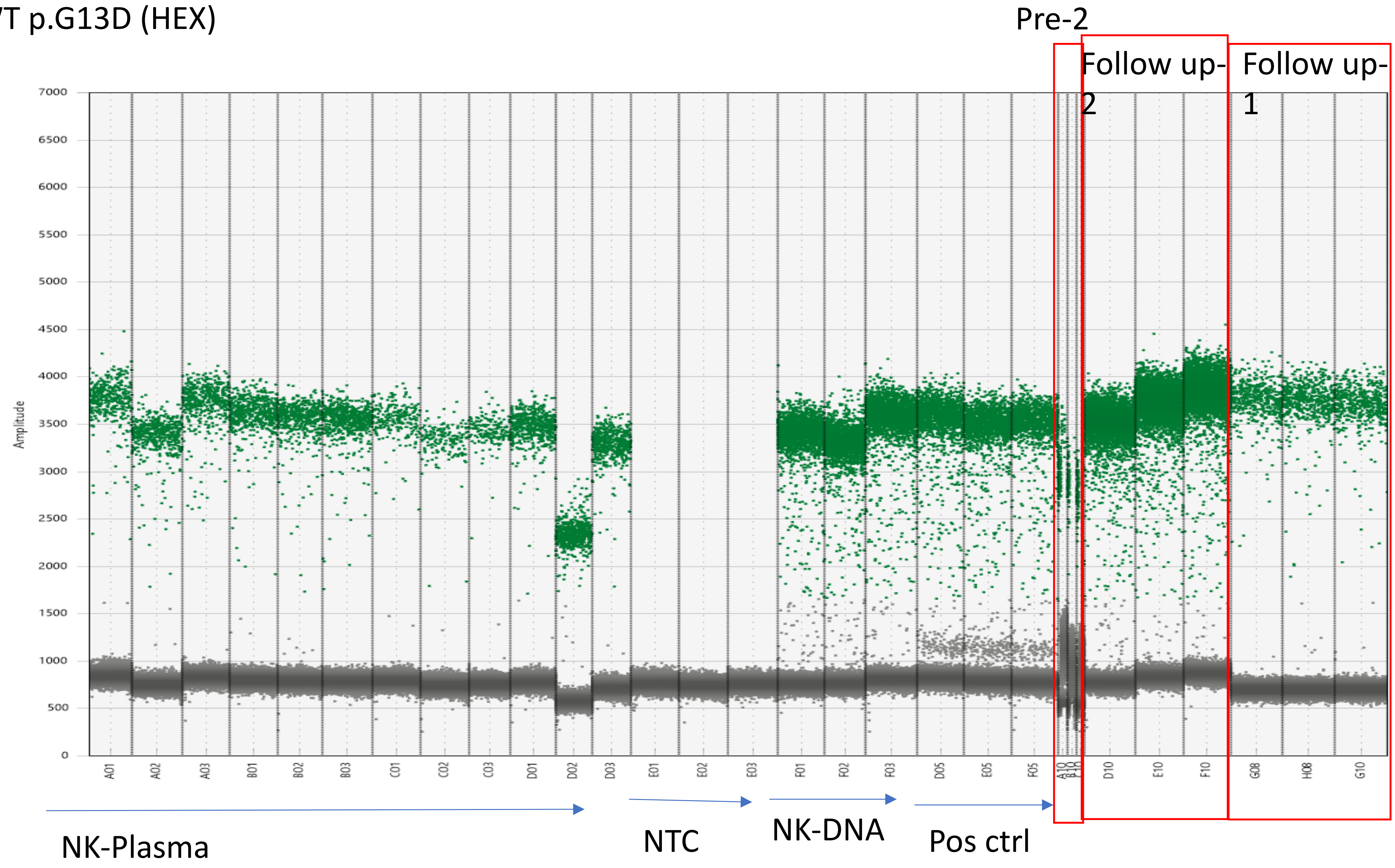

P23 -diluted

Gene target : TP53,p.R273H, c.818G>A and KRAS,p.G13D, c.38G>A

Assays: 0.7X TP53,p.R273H mut (FAM) and 1Xdark probe for TP53,p.R273H
0.3X KRAS p.G13D mut (FAM) and1X KRAS WT p.G13D (HEX)

0.7X TP53,p. R273H mut

0.3X KRAS p.G13D mut

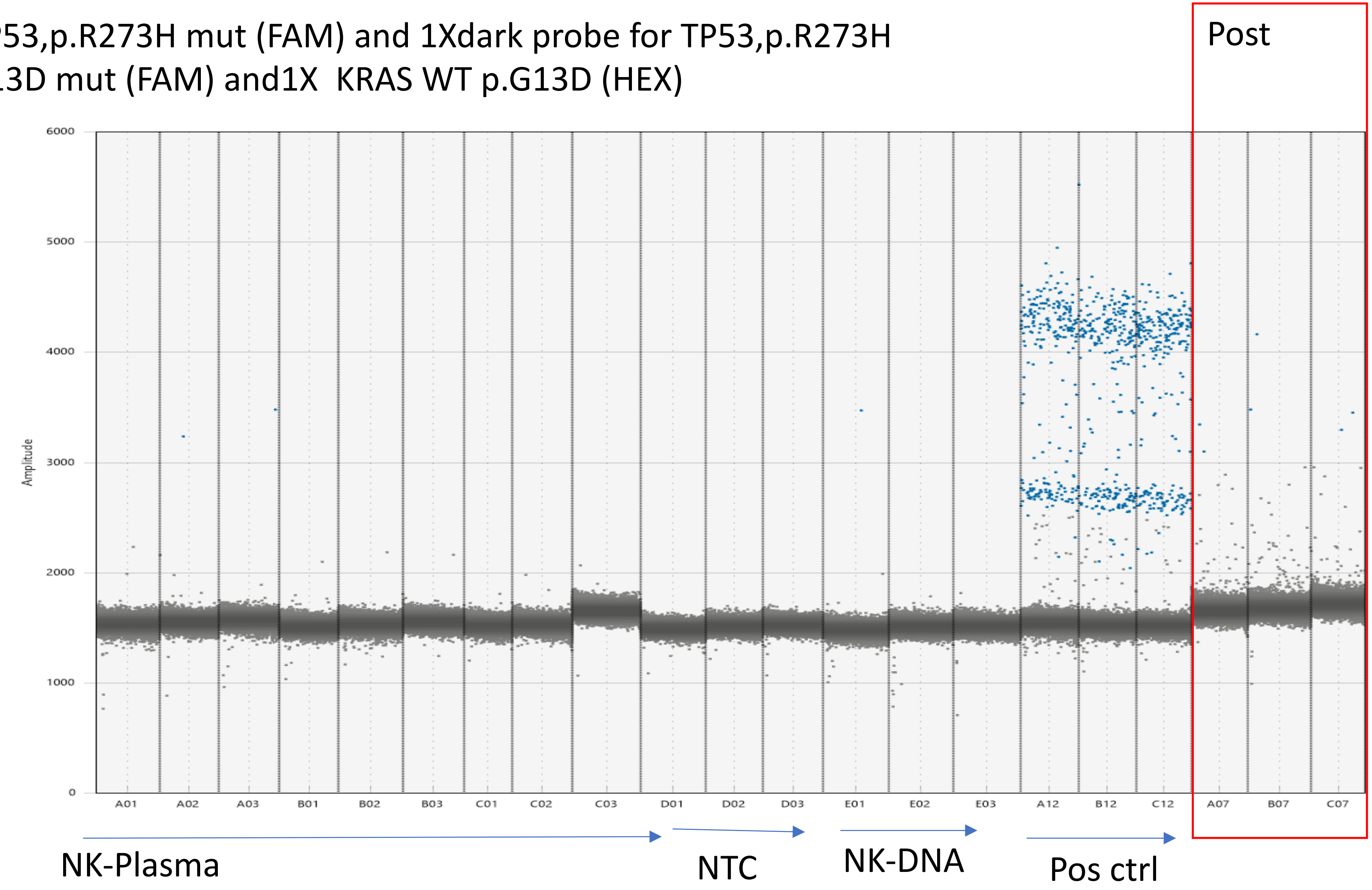

P23 -Final

Gene target : TP53,p.R273H, c.818G>A and KRAS,p.G13D, c.38G>A

Assays: 1Xdark probe for TP53,p.R273H
1X KRAS WT p.G13D (HEX)

0.3X KRAS
WT p.G13D

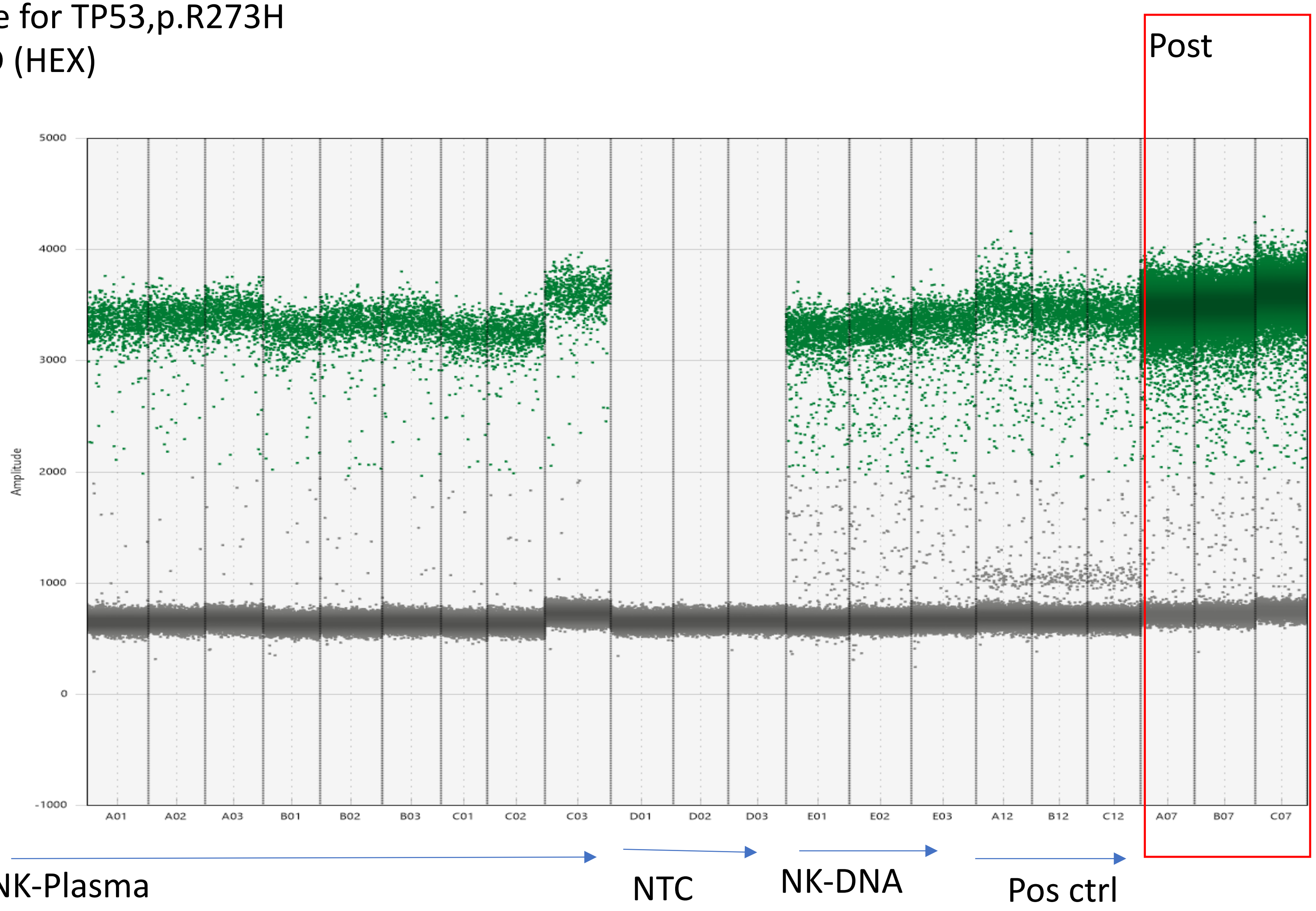

Positive control

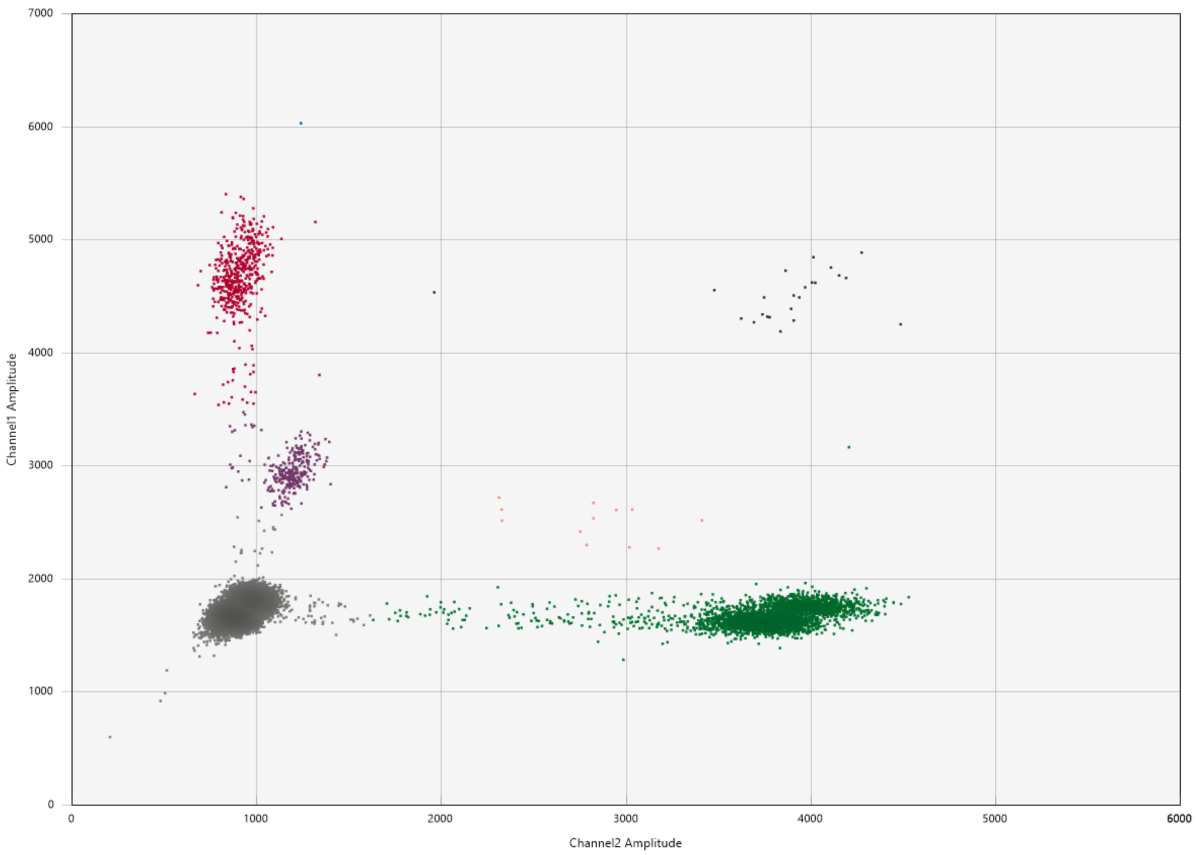

Nk-Plasma

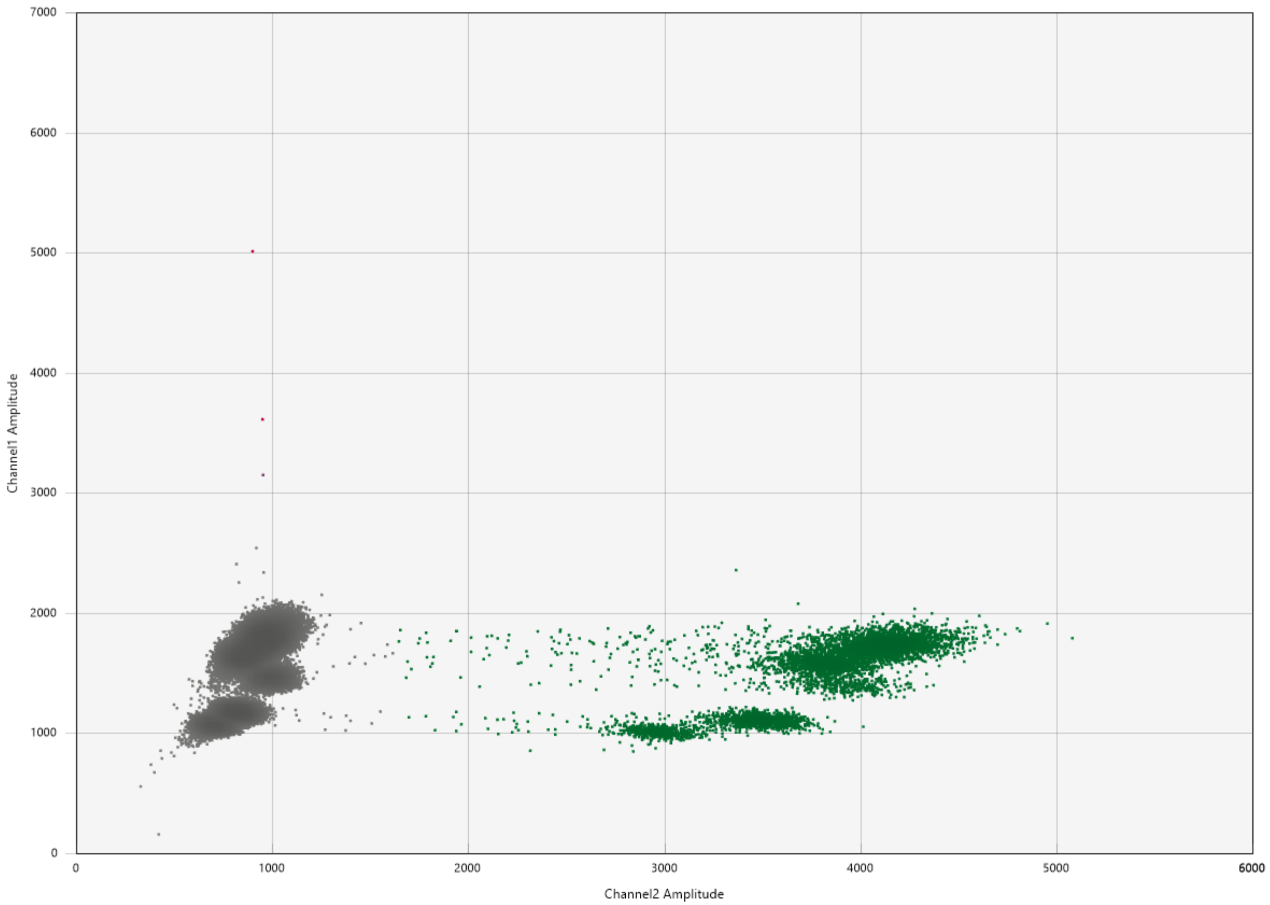

Pre-surgery

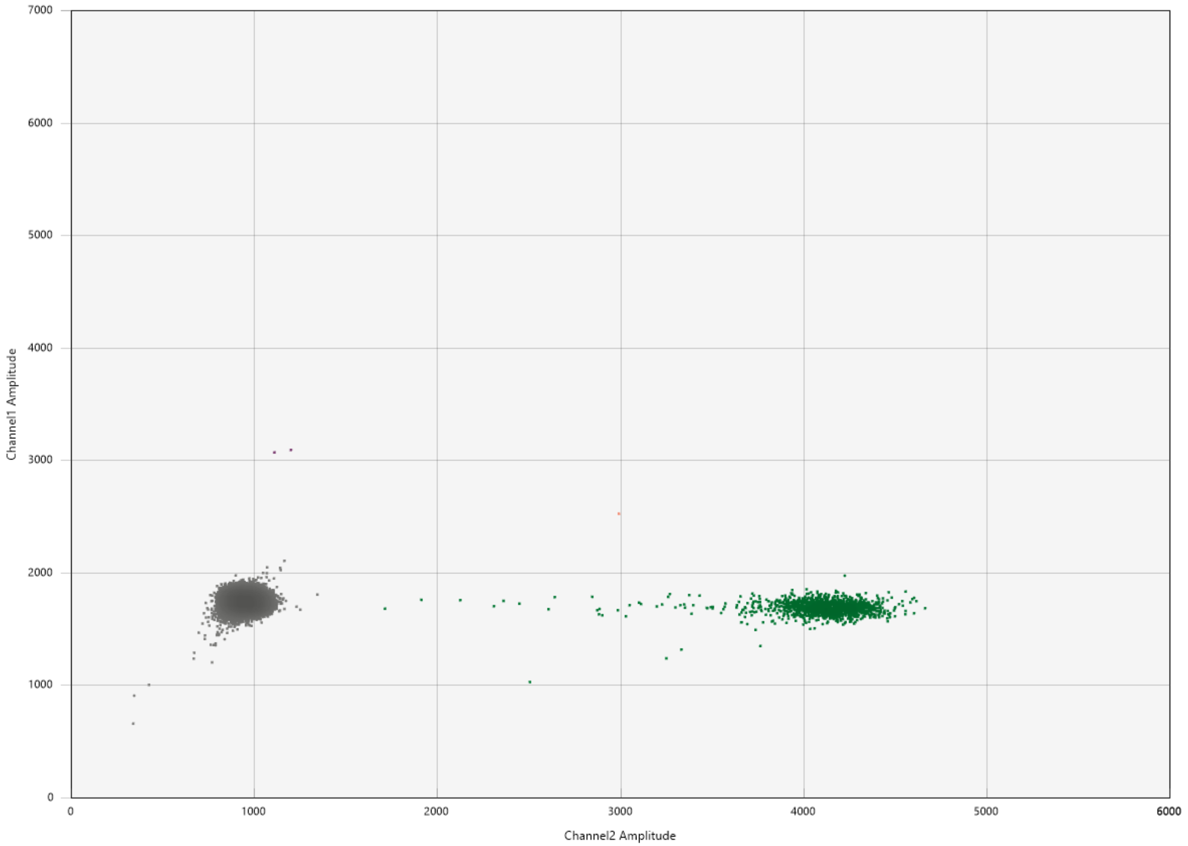

Pre-surgery-2

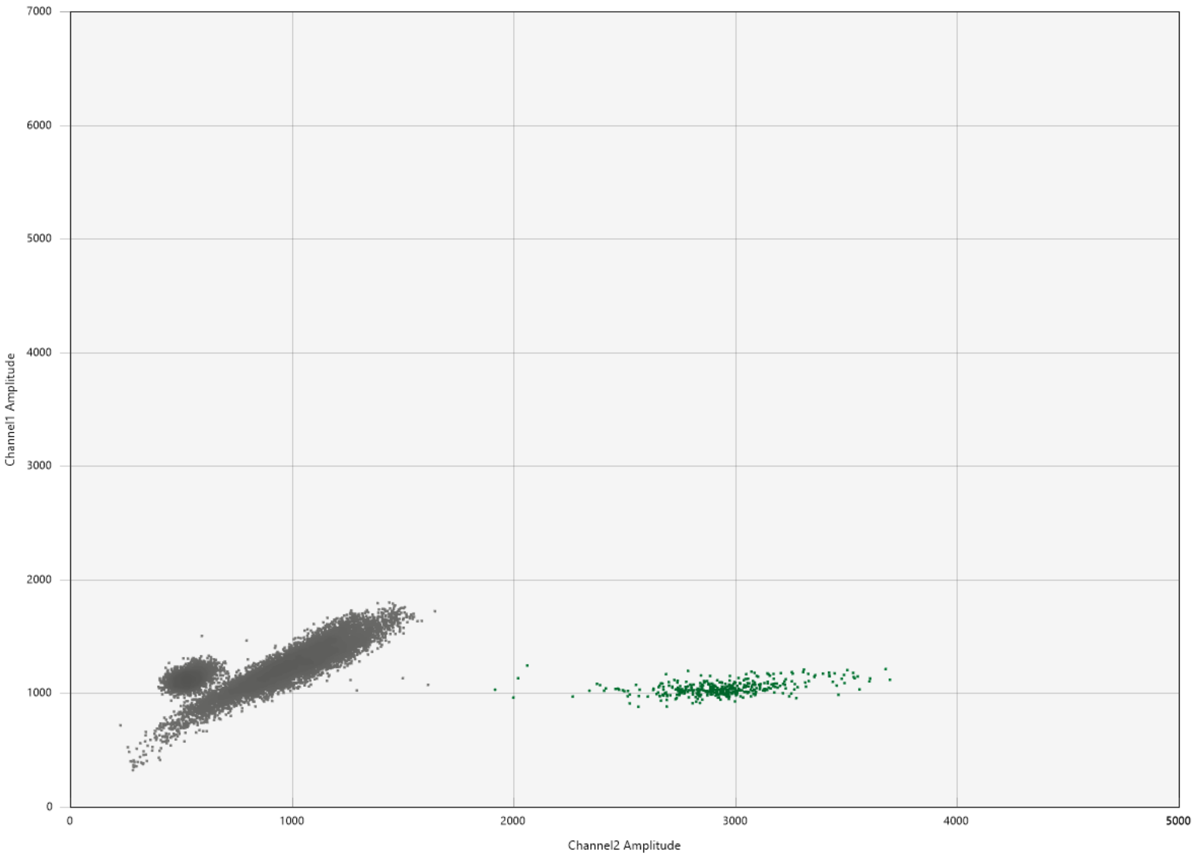

Post-surgery*

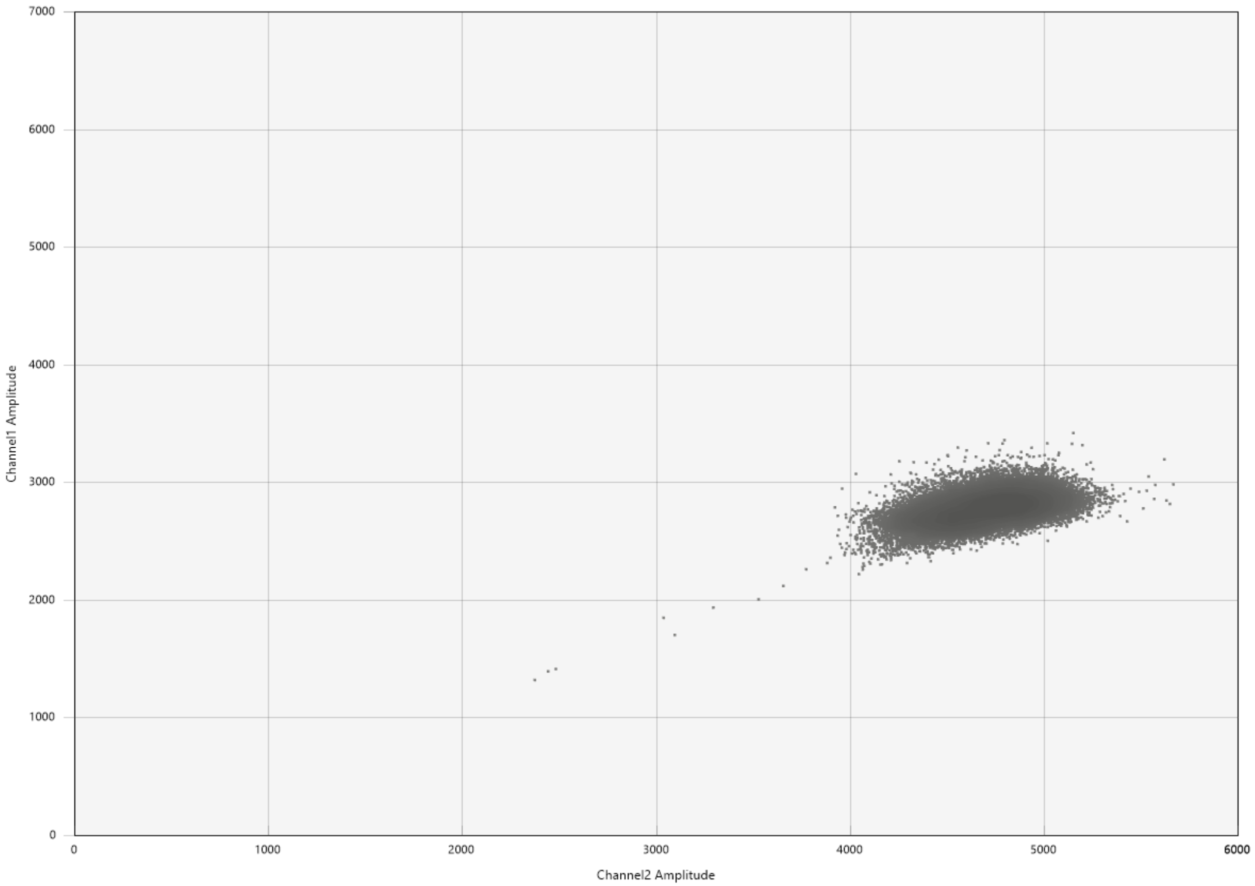

Post-surgery*- diluted

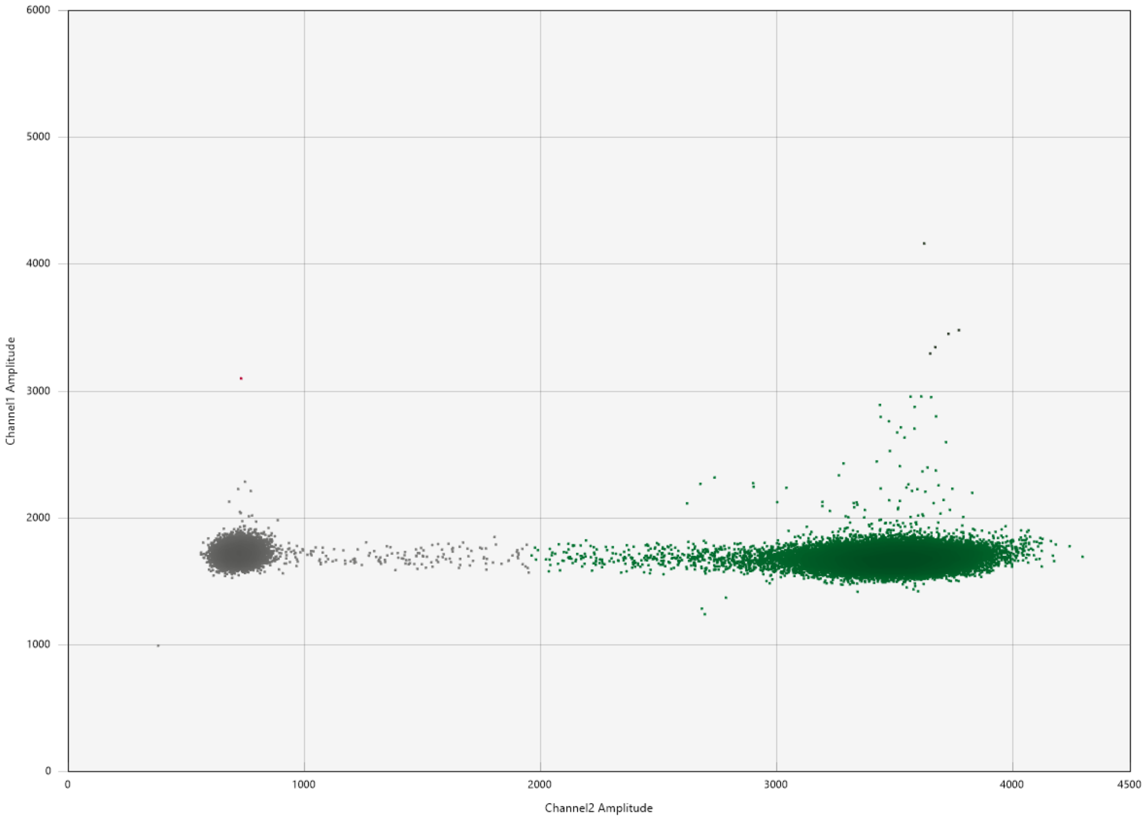

Follow- up-1

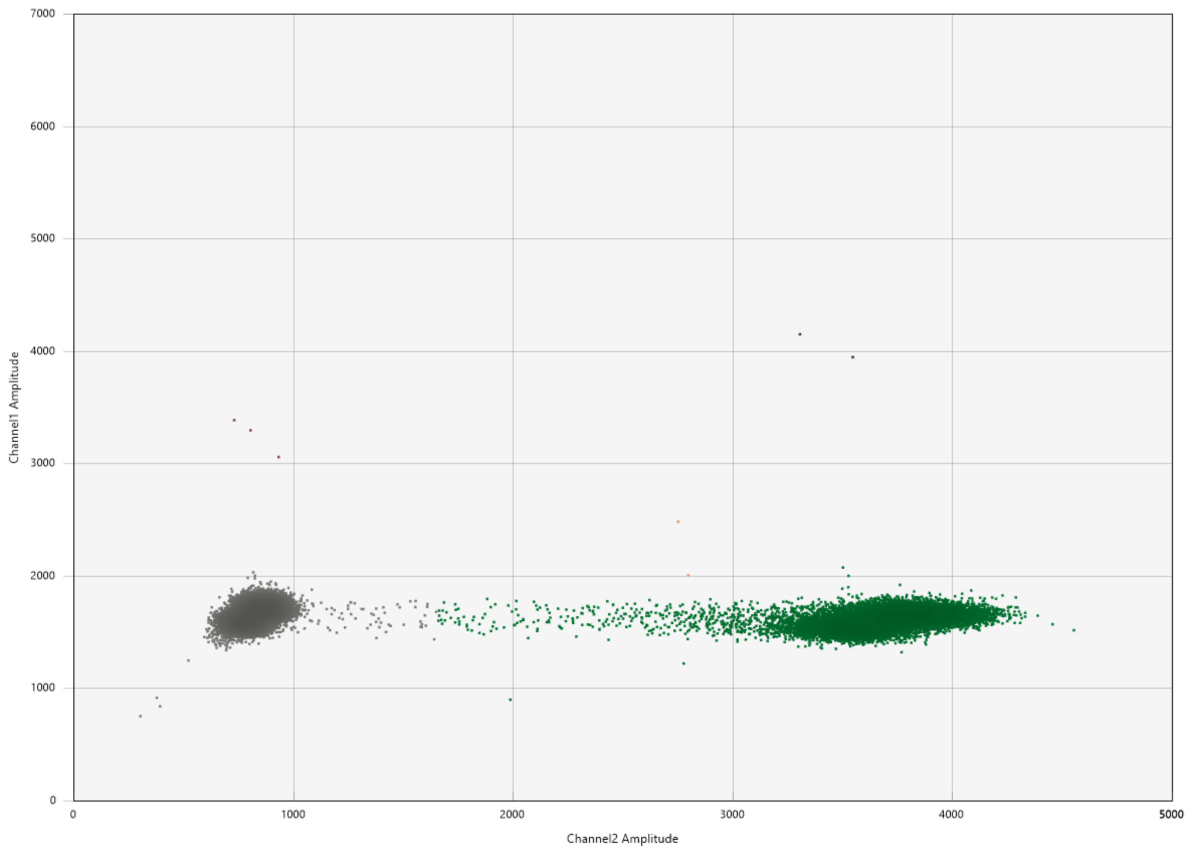

Follow-up-2

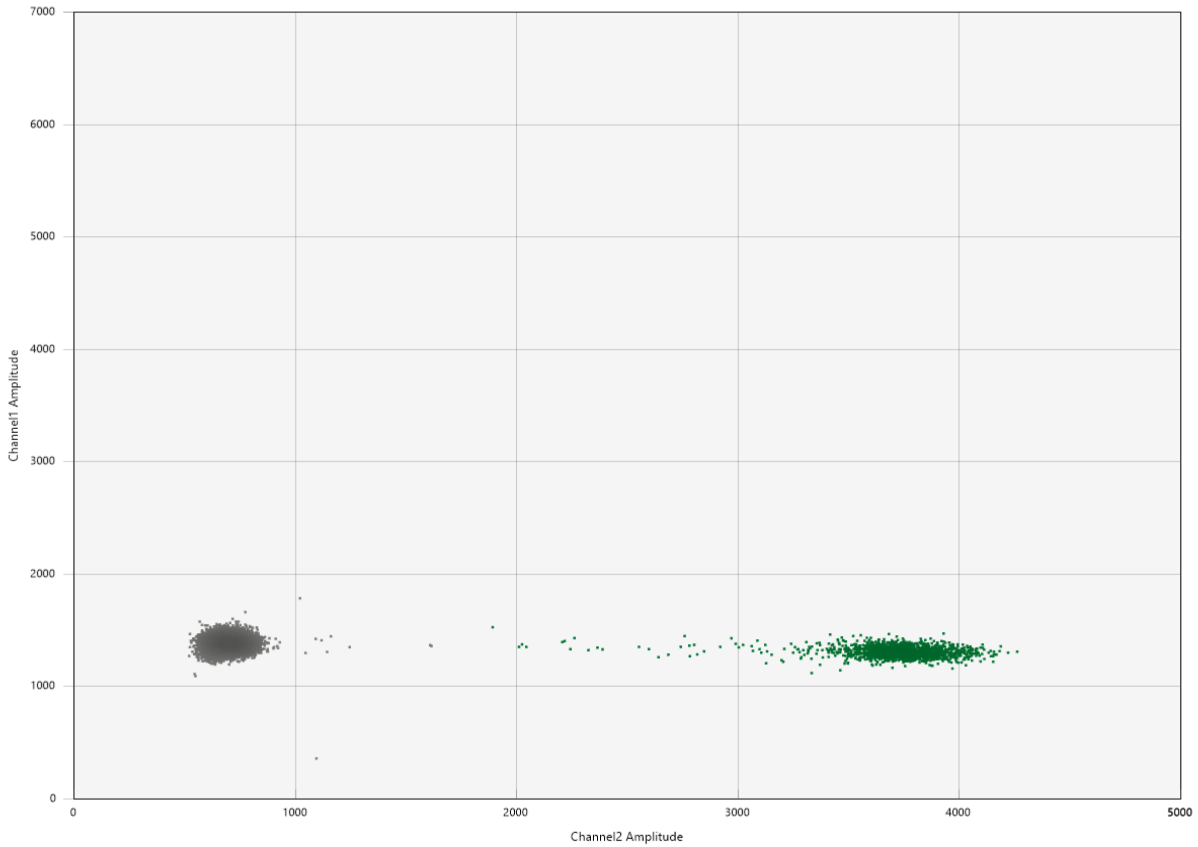

Concentration plots

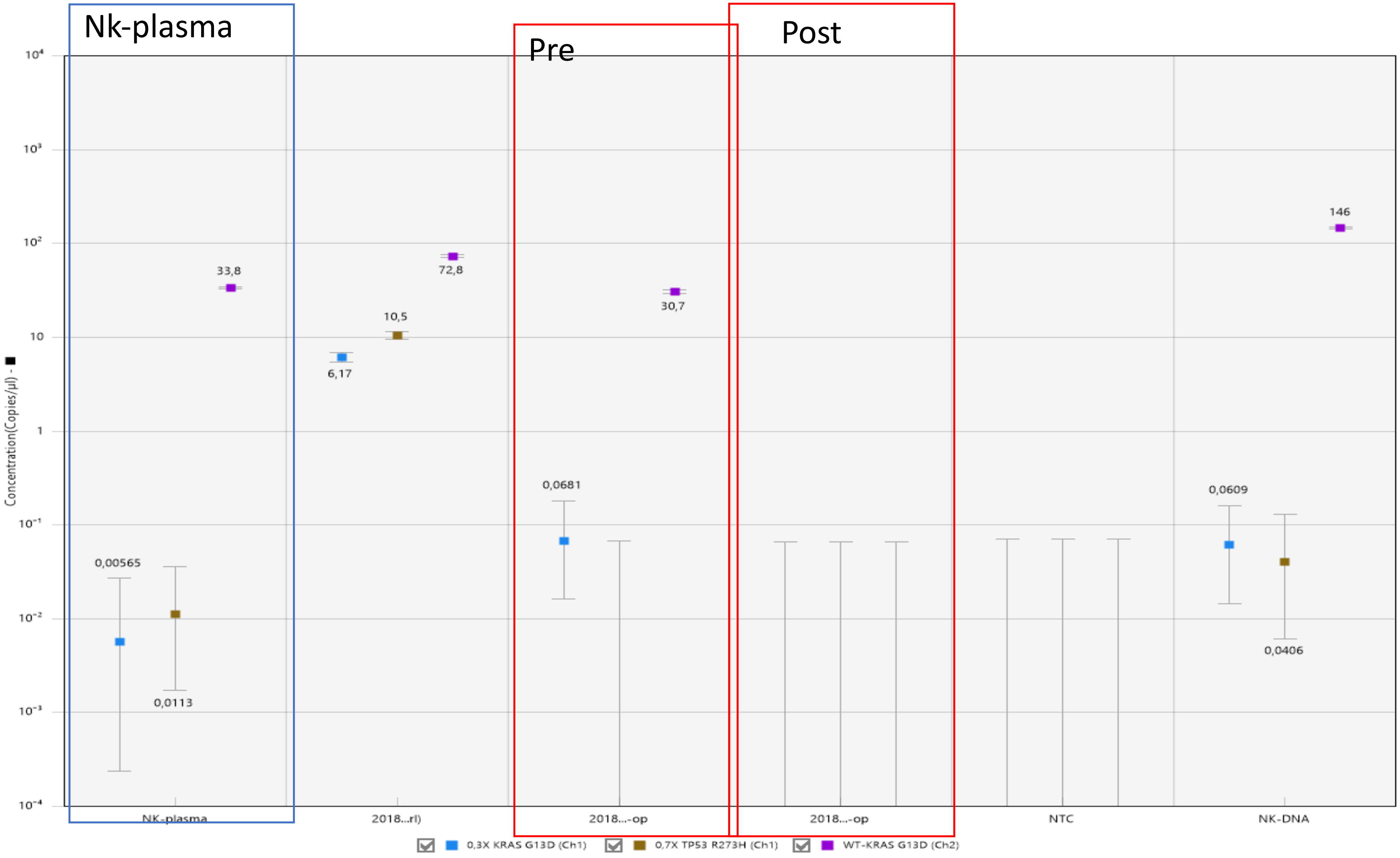

Post, Follow-up 1 and 2

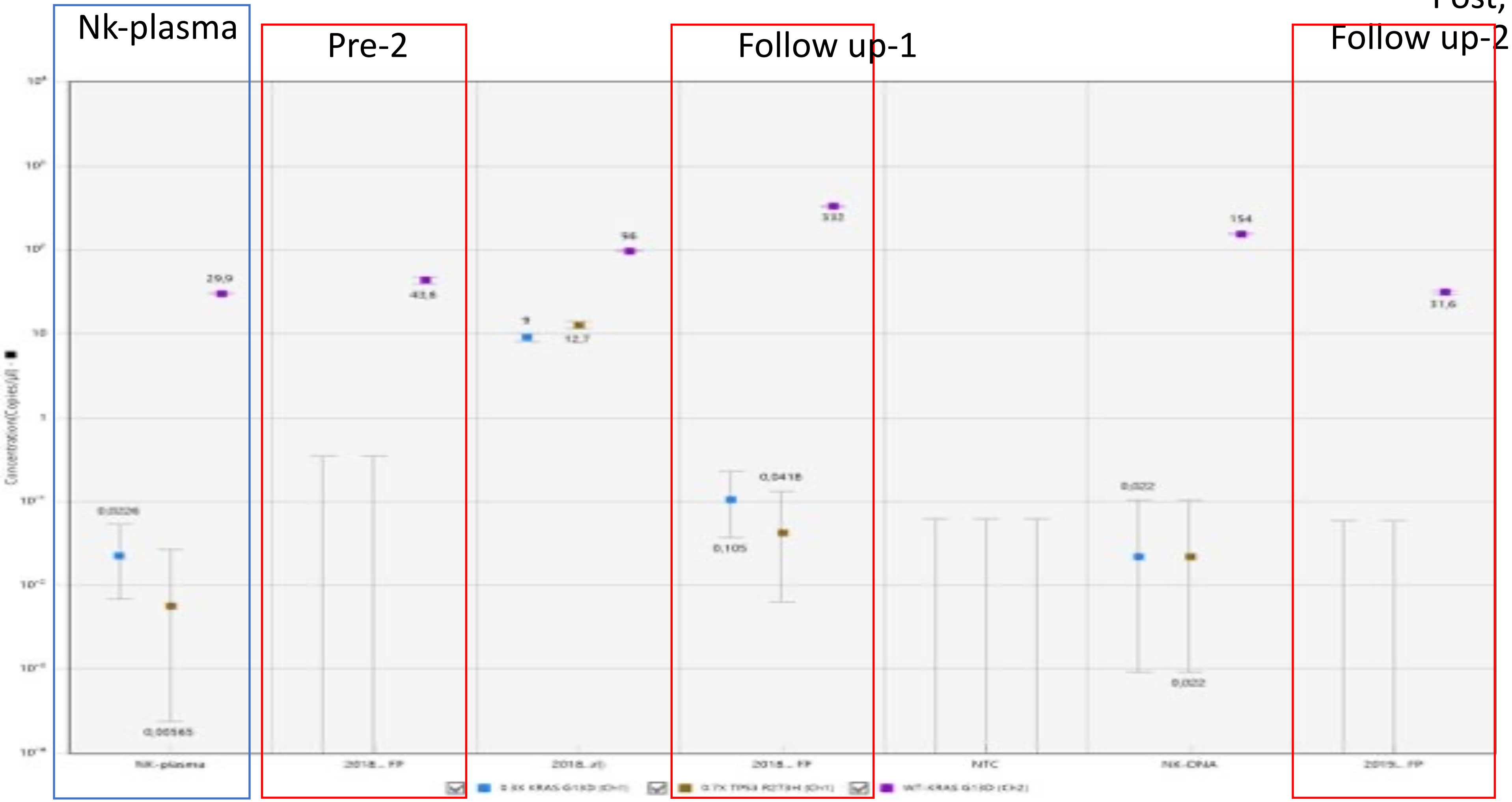

Post- surgery - diluted

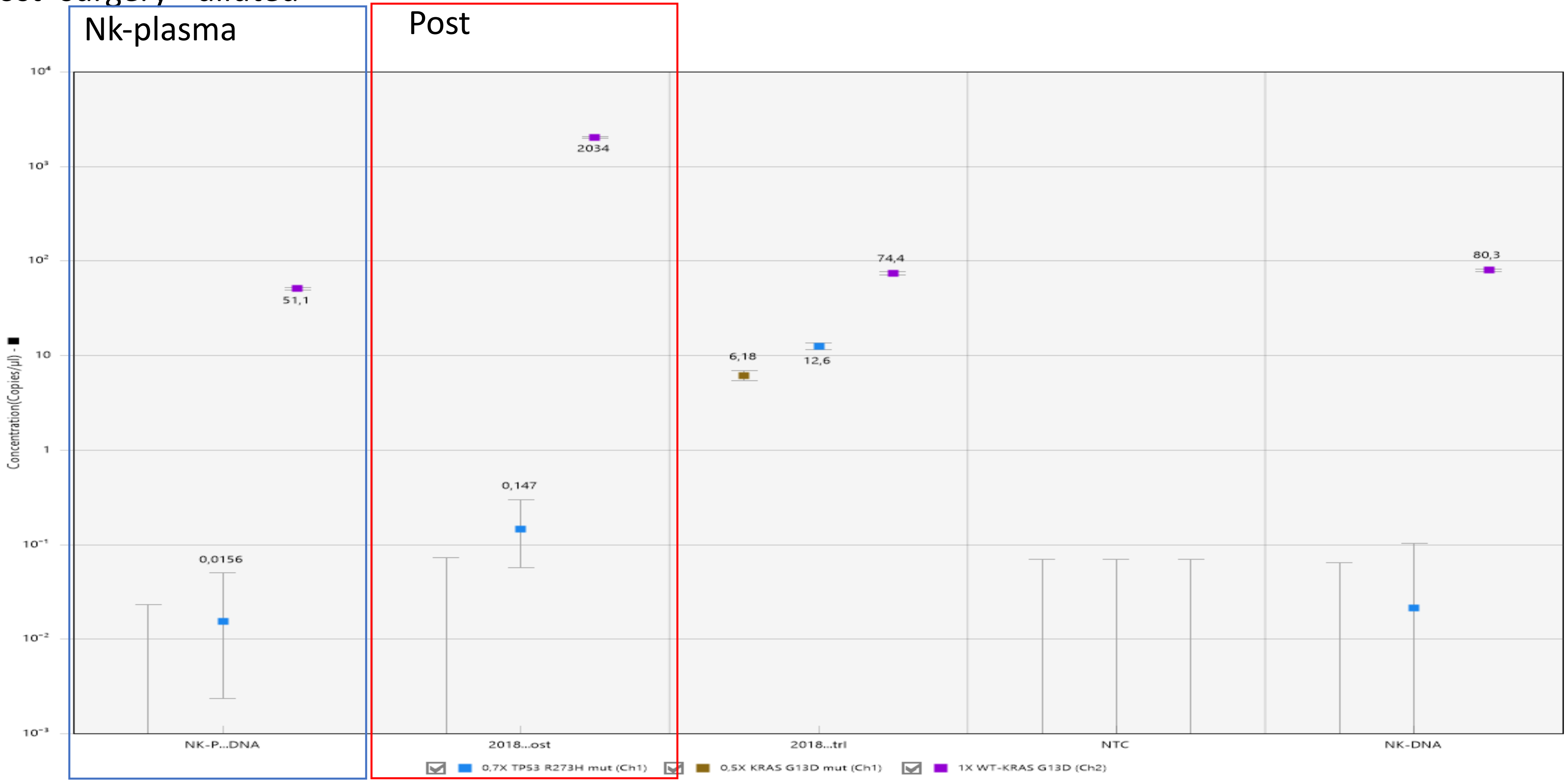

P12 - Final

Gene target : APC, p. R823*, c.2467C>T and TP53,c.720_766del

Assays: 0.5X TP53,c.720_766del mut (FAM)
1X APC, p. R823* mut (FAM) and 1X APC WT, p. R823* (HEX)

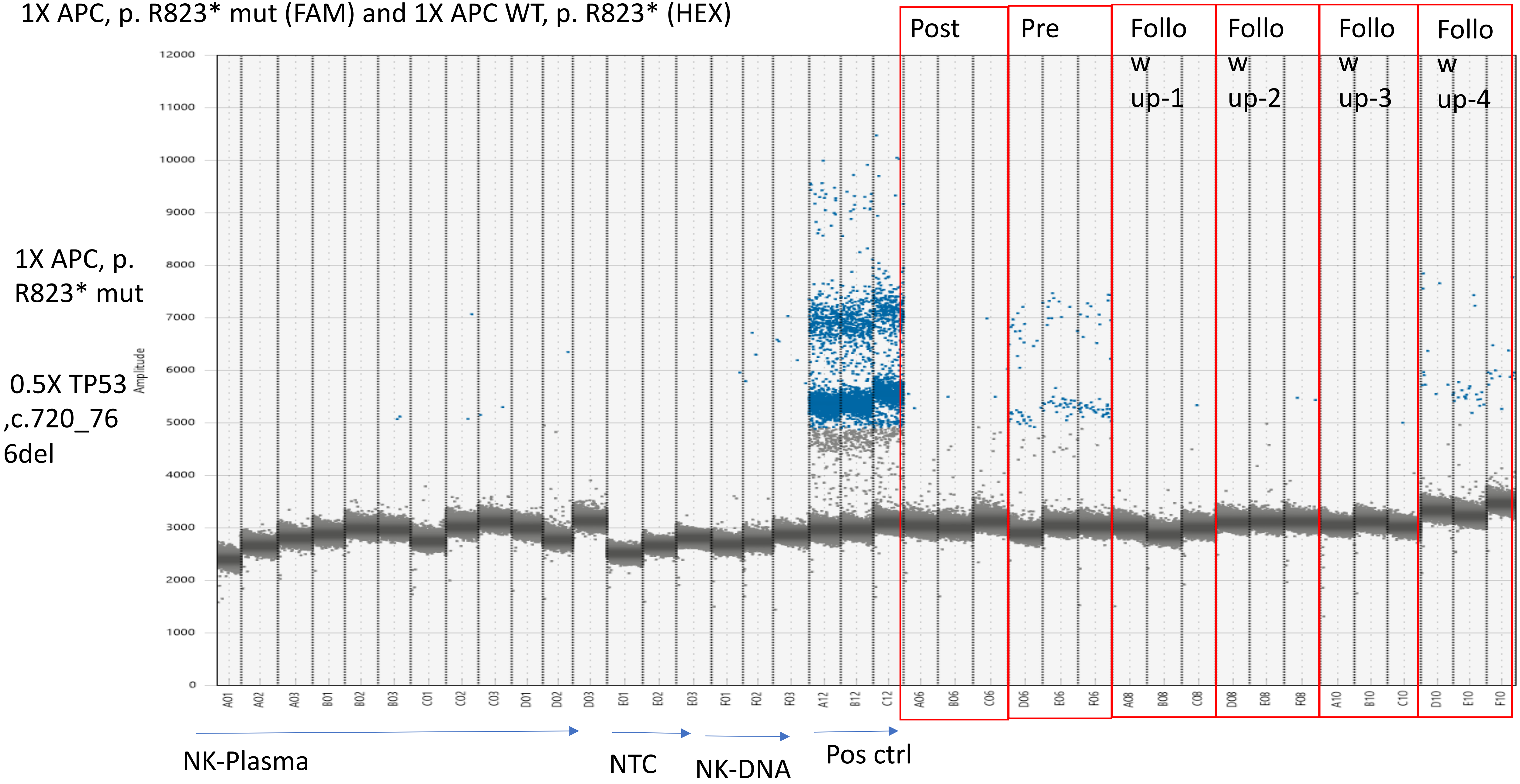

P12 – Final WT

Gene target : APC, p. R823*, c.2467C>T and TP53,c.720_766del

Assays: 1X APC WT, p. R823* (HEX)

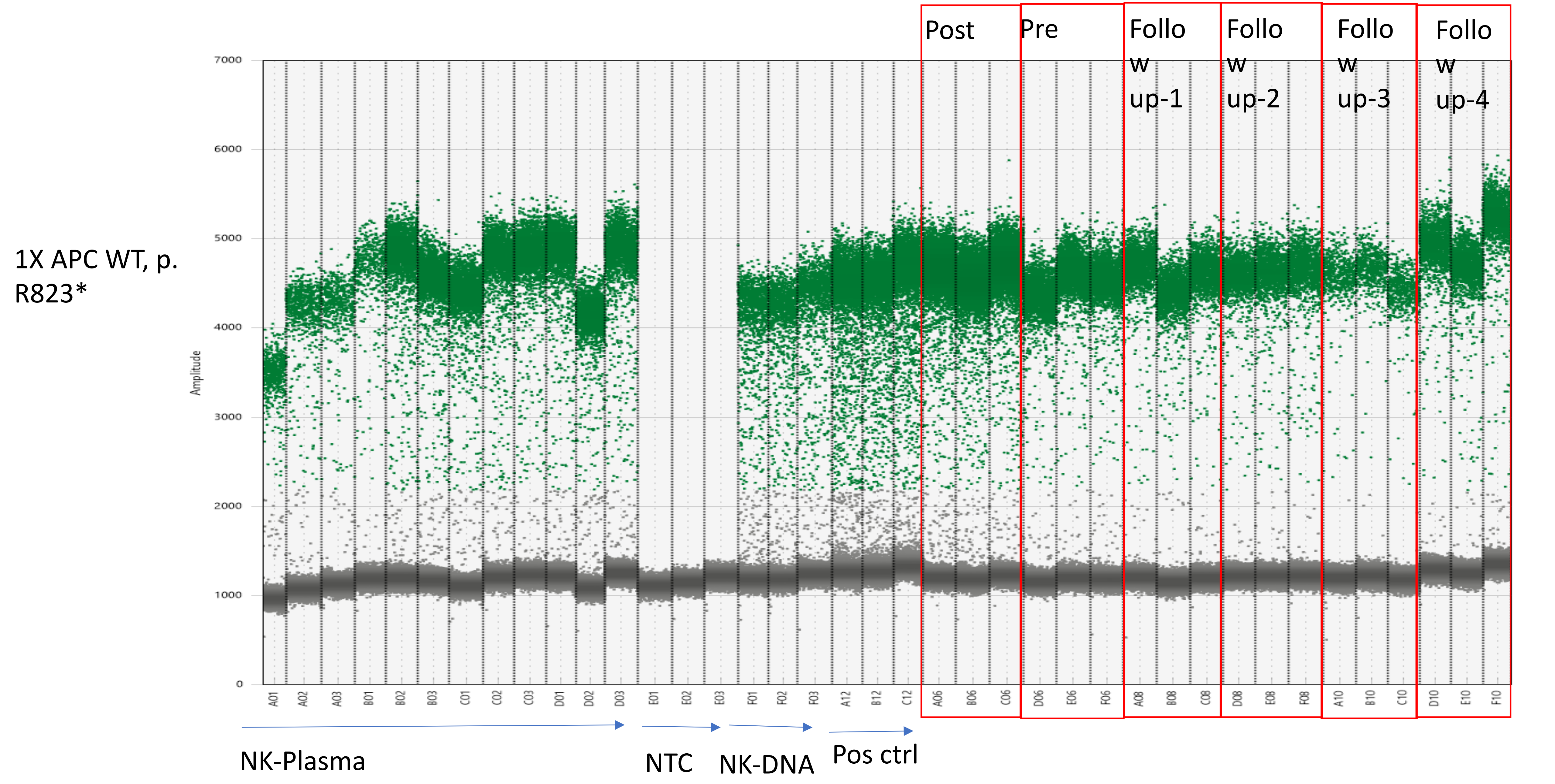

Positive control

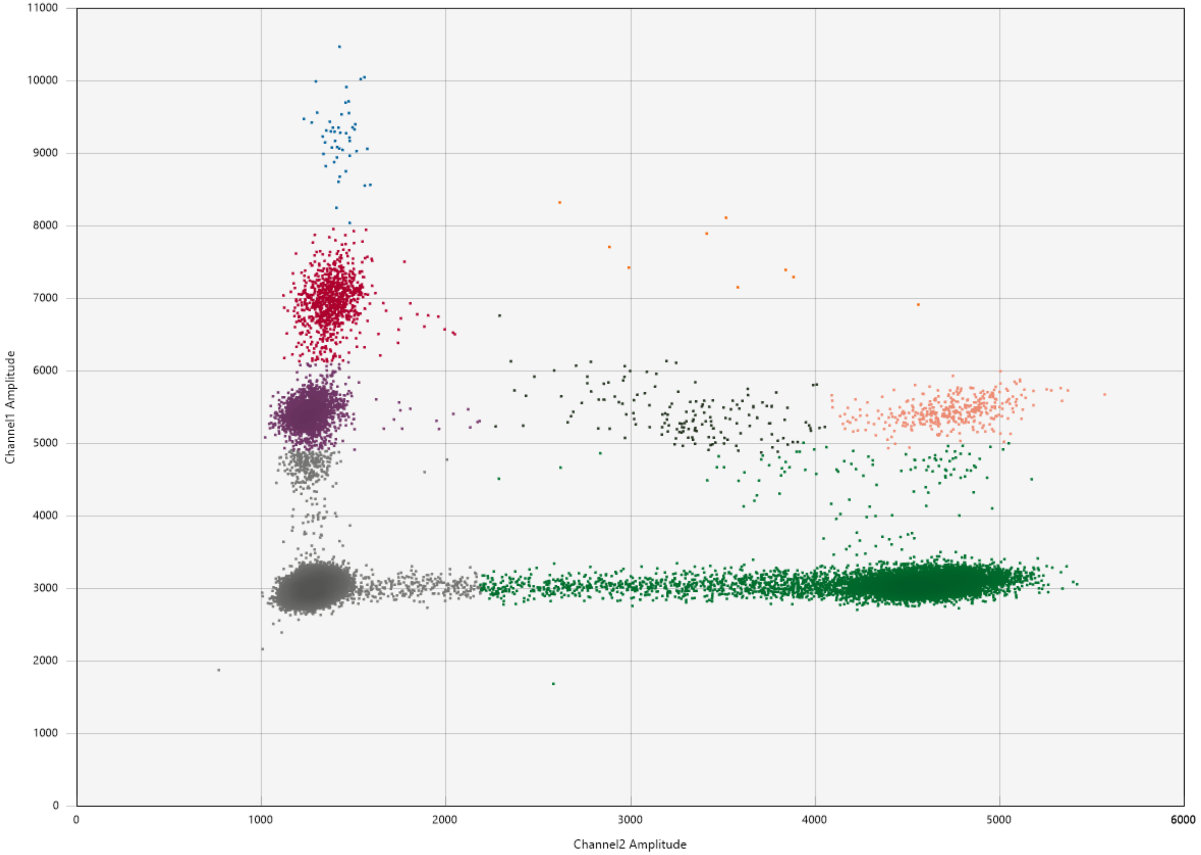

NK-Plasma

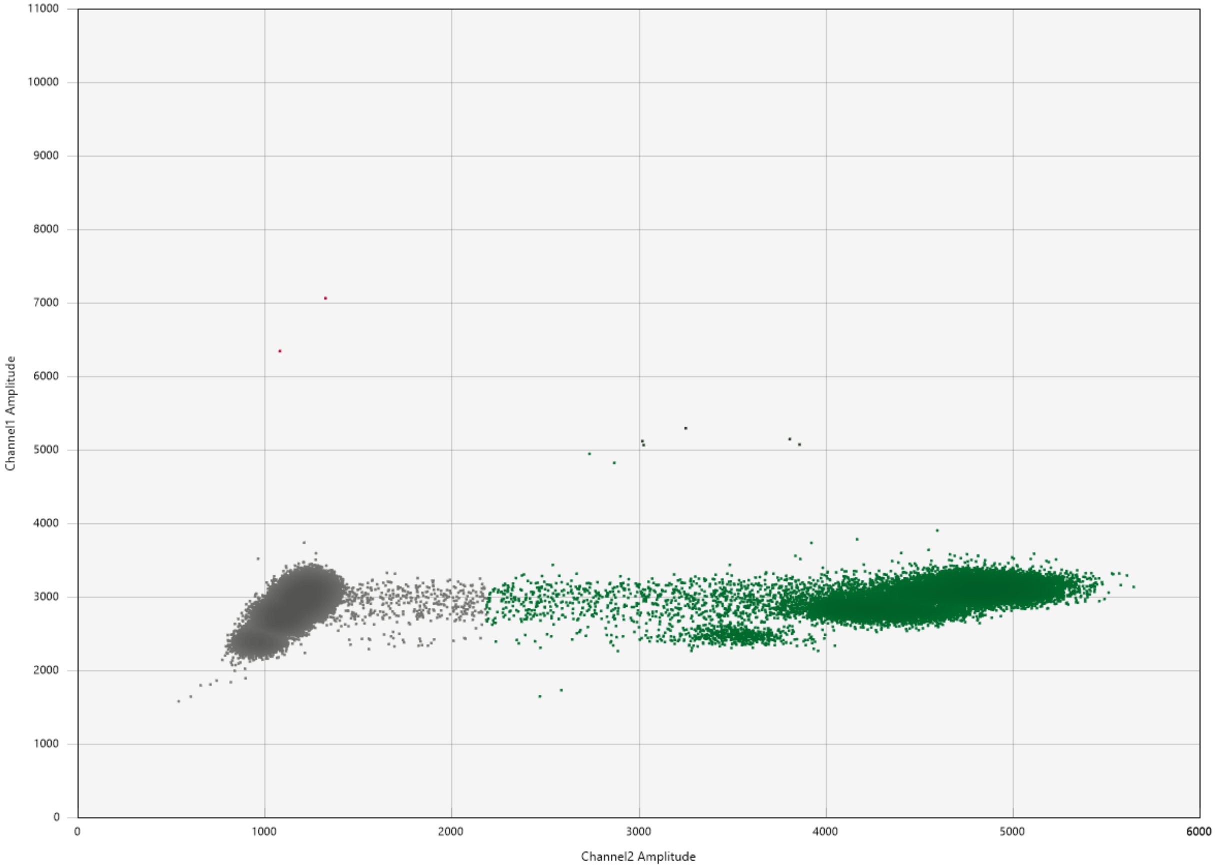

Pre-surgery

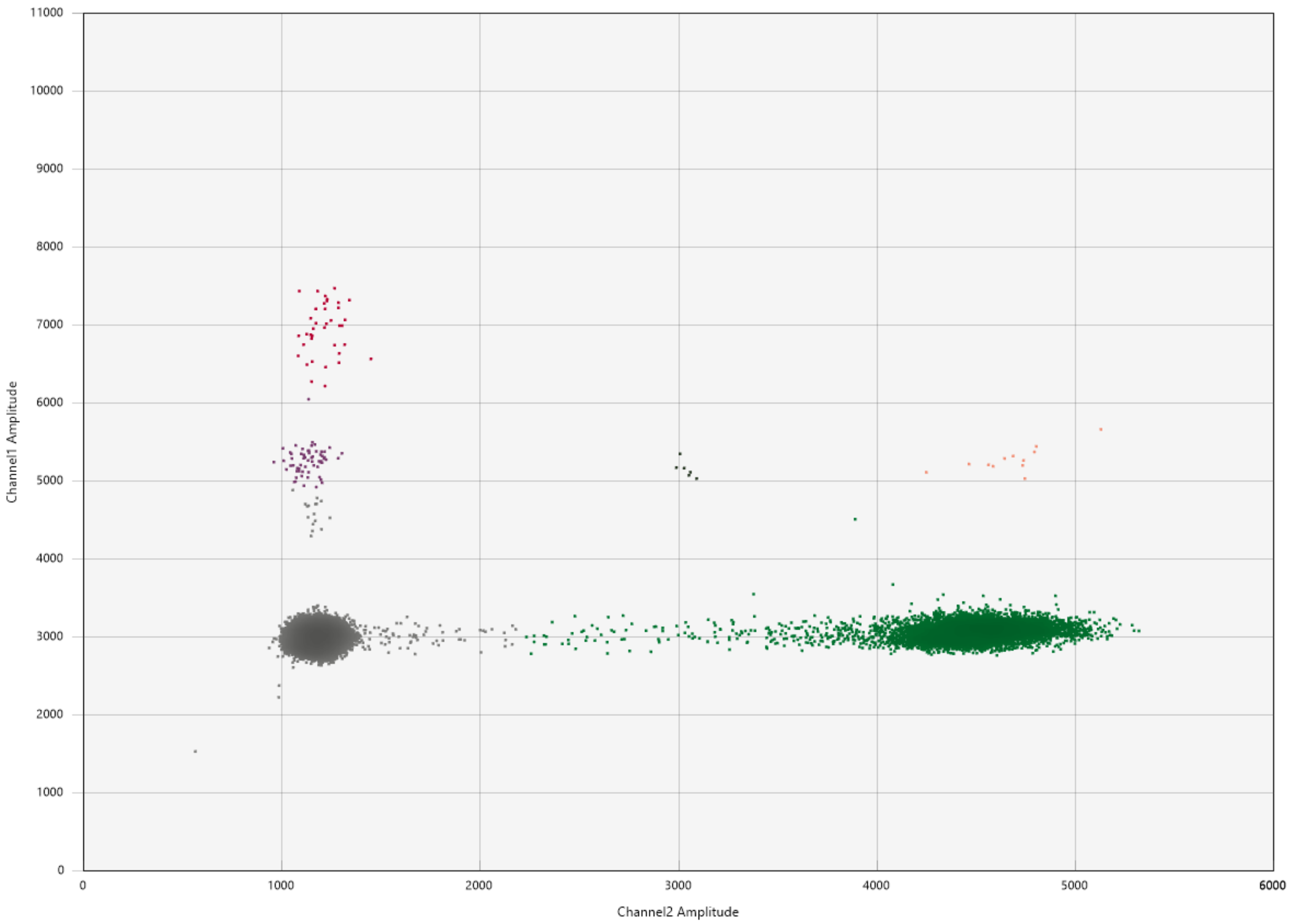

Follow up during chemo-1

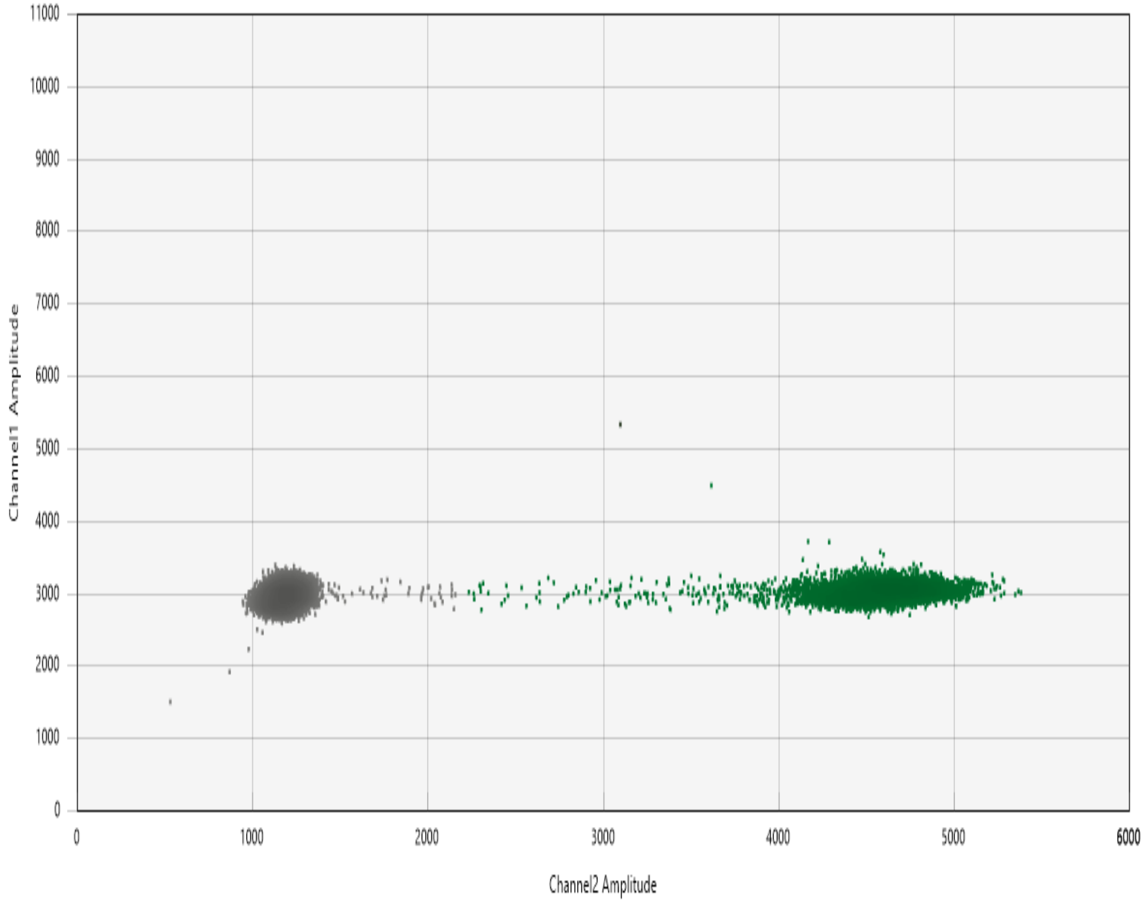

Follow up during chemo-2

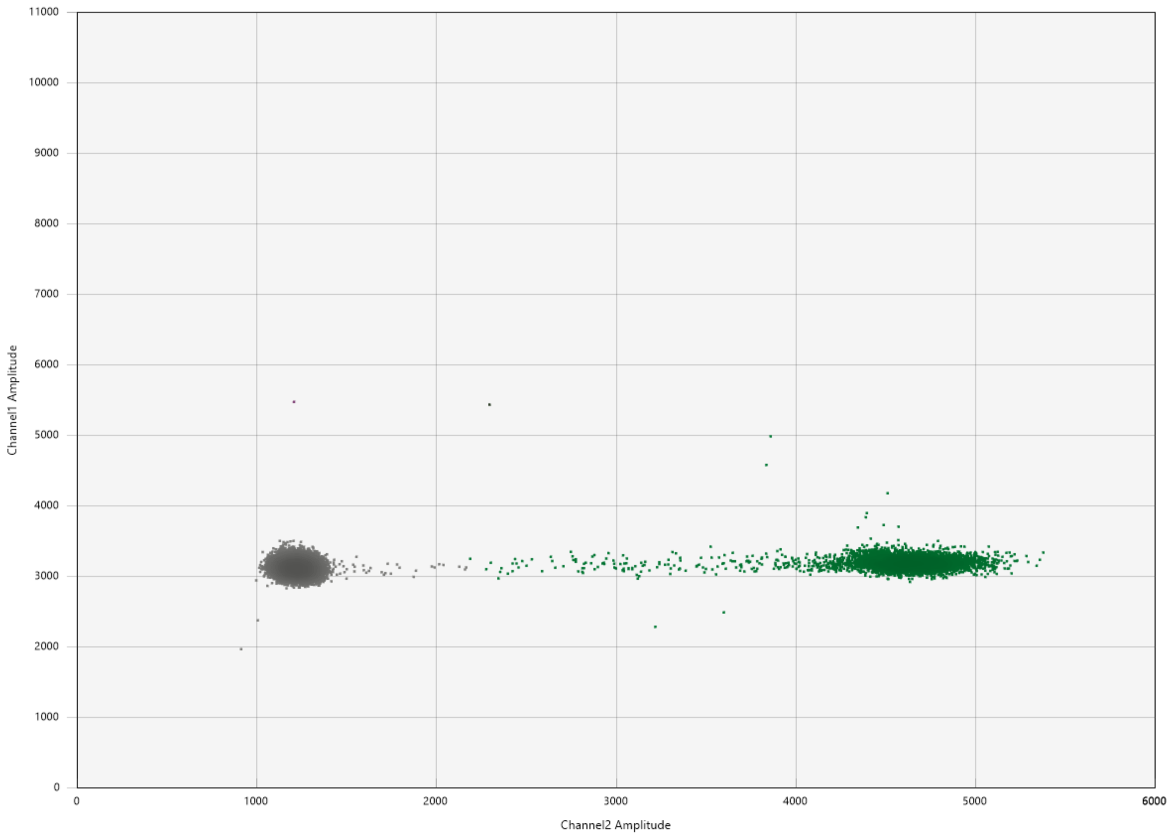

Follow up during chemo-3

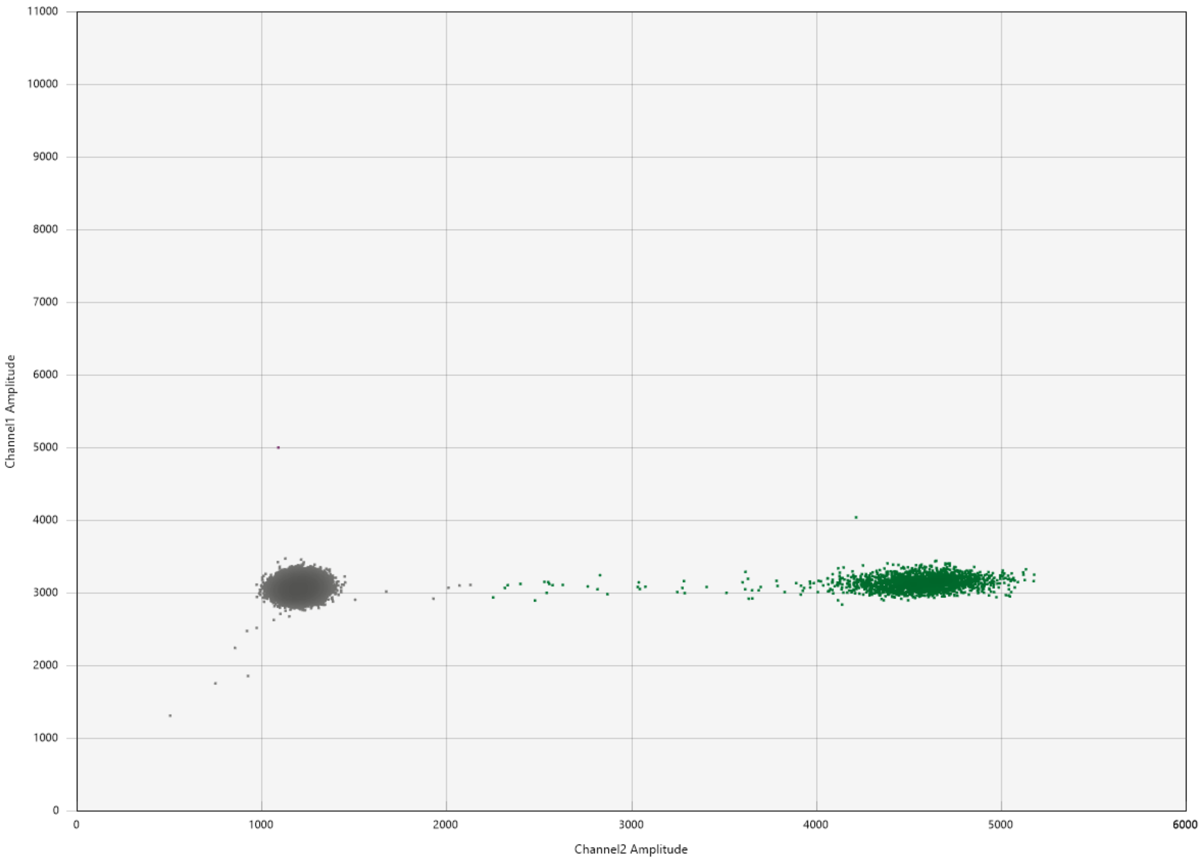

Follow up during chemo-4

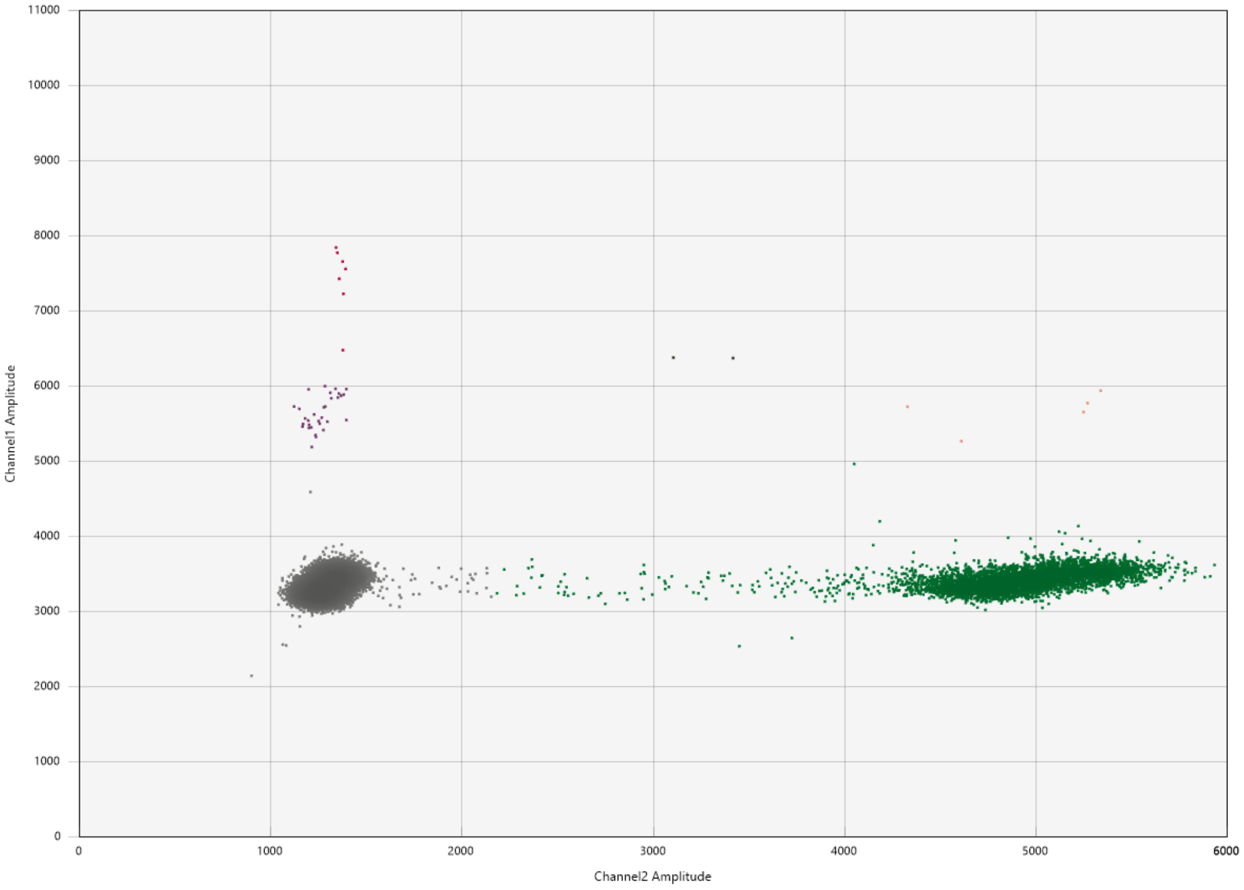

Post-surgery

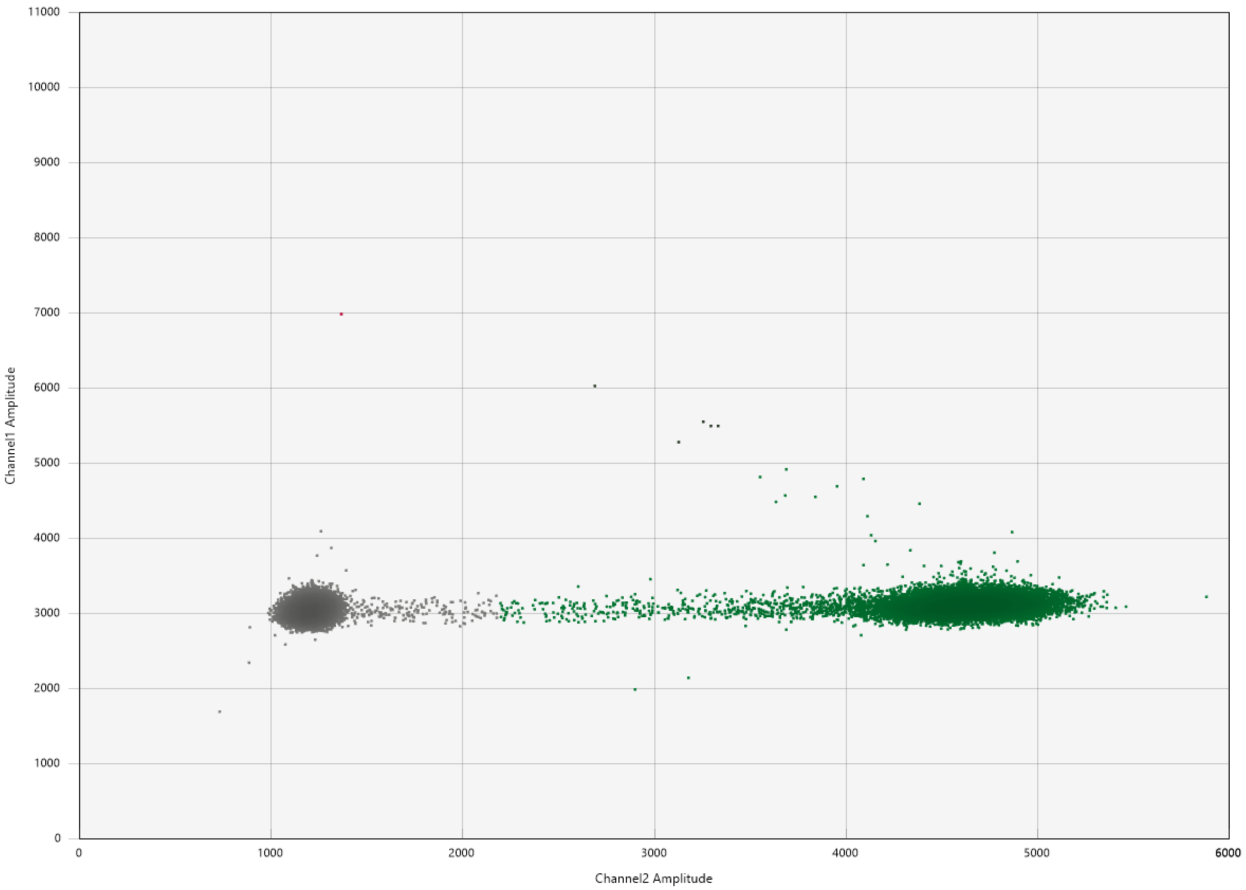

Concentration plots

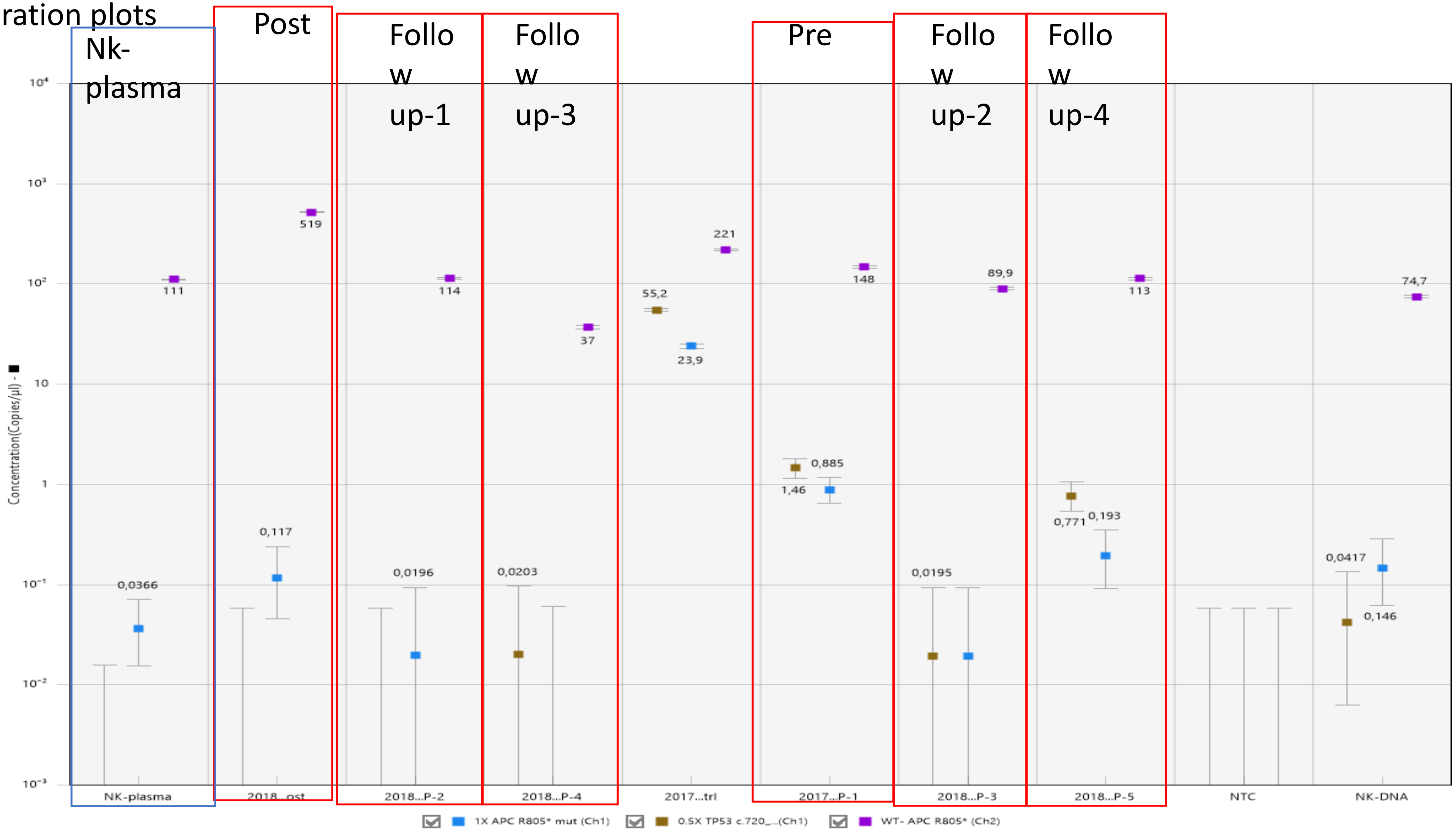

P41 - Final

Gene targets : RB1, p.R358*, c.1072C>T and TP53,p.R273C, c.817C>T

Assays: 0.5X TP53,p.R273C mut (FAM) and 1X dark probe for TP53,p.R273C

1X RB1, p.R358* mut (FAM) and 1X RB1 WT, p.R358* (HEX)

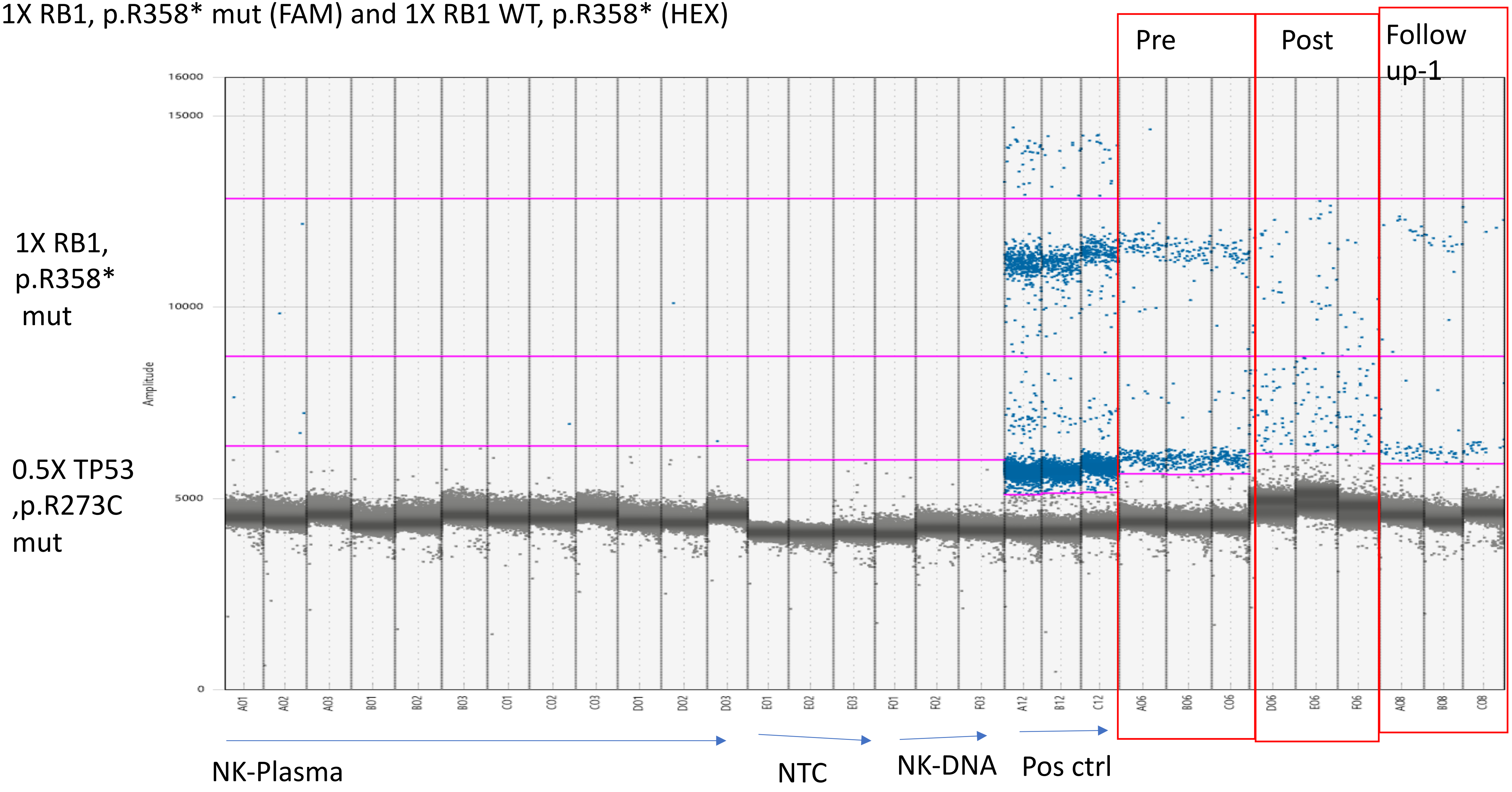

P41 - Final WT

Gene targets : RB1, p.R358*, c.1072C>T and TP53,p.R273C, c.817C>T

Assays: 1X dark probe for TP53,p.R273C and 1X RB1 WT, p.R358* (HEX)

1X RB1 WT, p.R358*

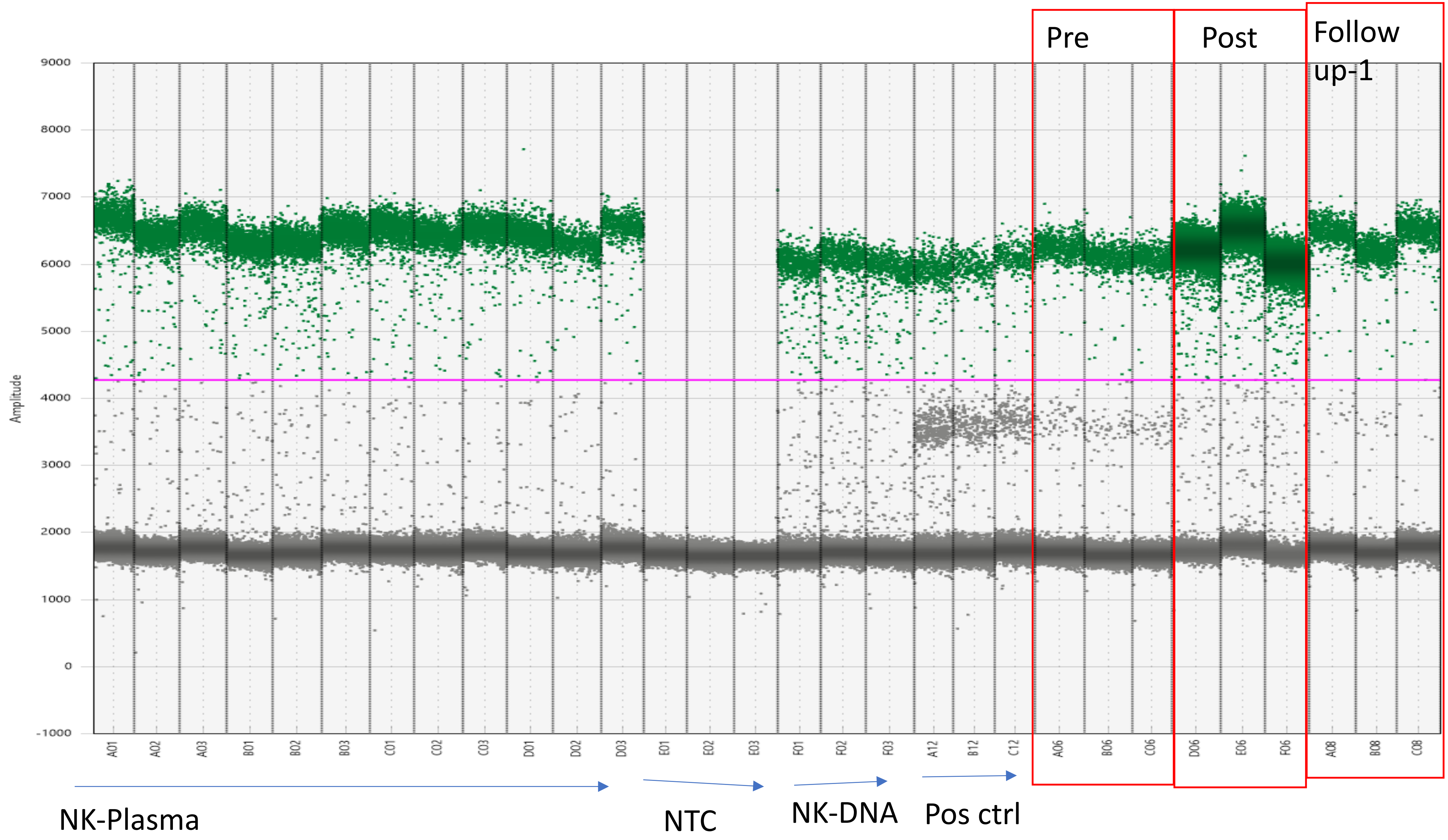

P41 - Final dil

Gene targets : RB1, p.R358*, c.1072C>T and TP53,p.R273C, c.817C>T

Assays: 0.5X TP53,p.R273C mut (FAM) and 1X dark probe for TP53,p.R273C

1X RB1, p.R358* mut (FAM) and 1X RB1 WT, p.R358* (HEX)

1X RB1, p.R358* mut

0.5X TP53 ,p.R273C mut

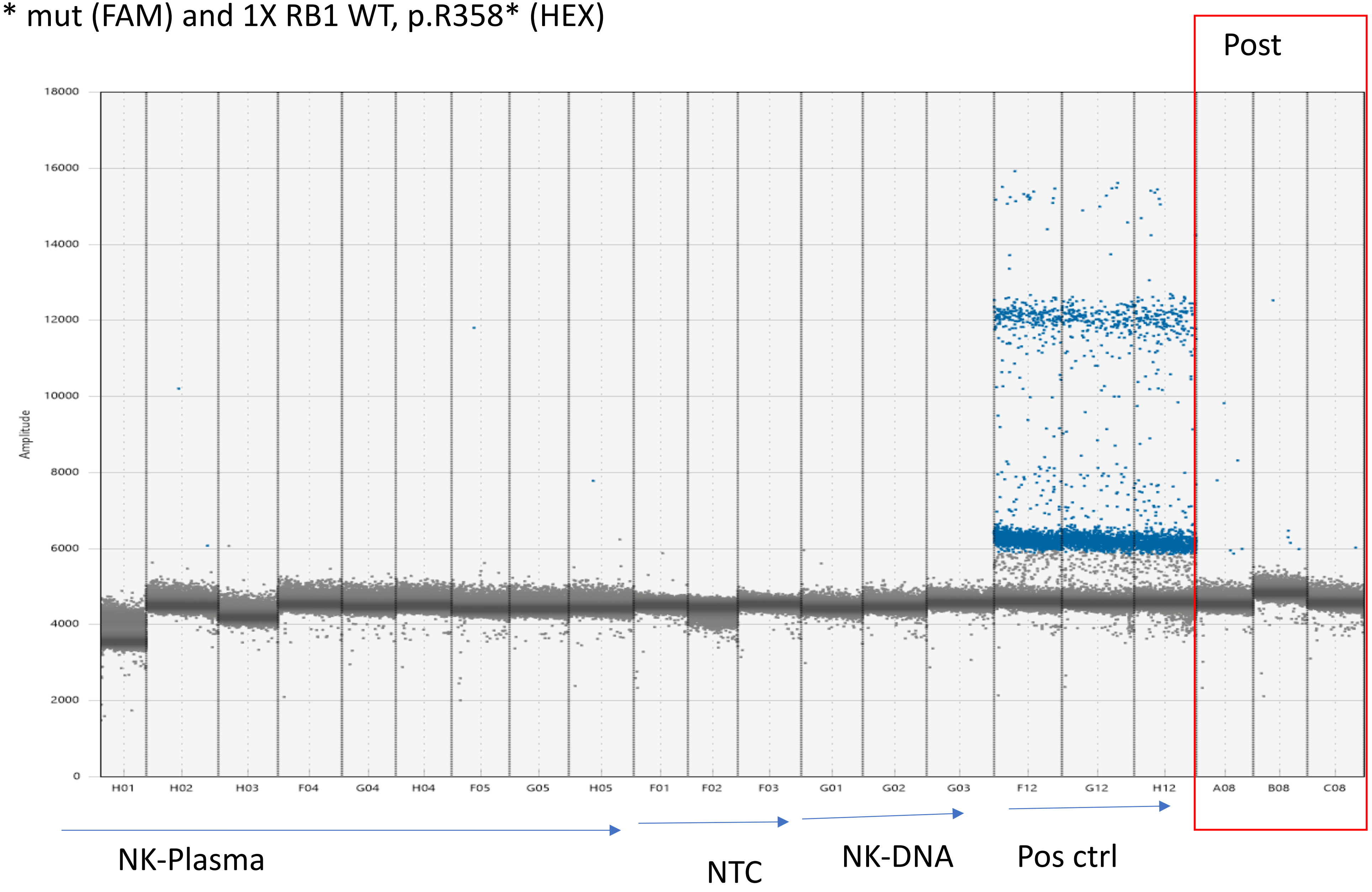

P41 - Final dil -WT

Gene targets : RB1, p.R358*, c.1072C>T and TP53,p.R273C, c.817C>T

Assays: 1X dark probe for TP53,p.R273C and 1X RB1 WT, p.R358* (HEX)

1X RB1 WT, p.R358*

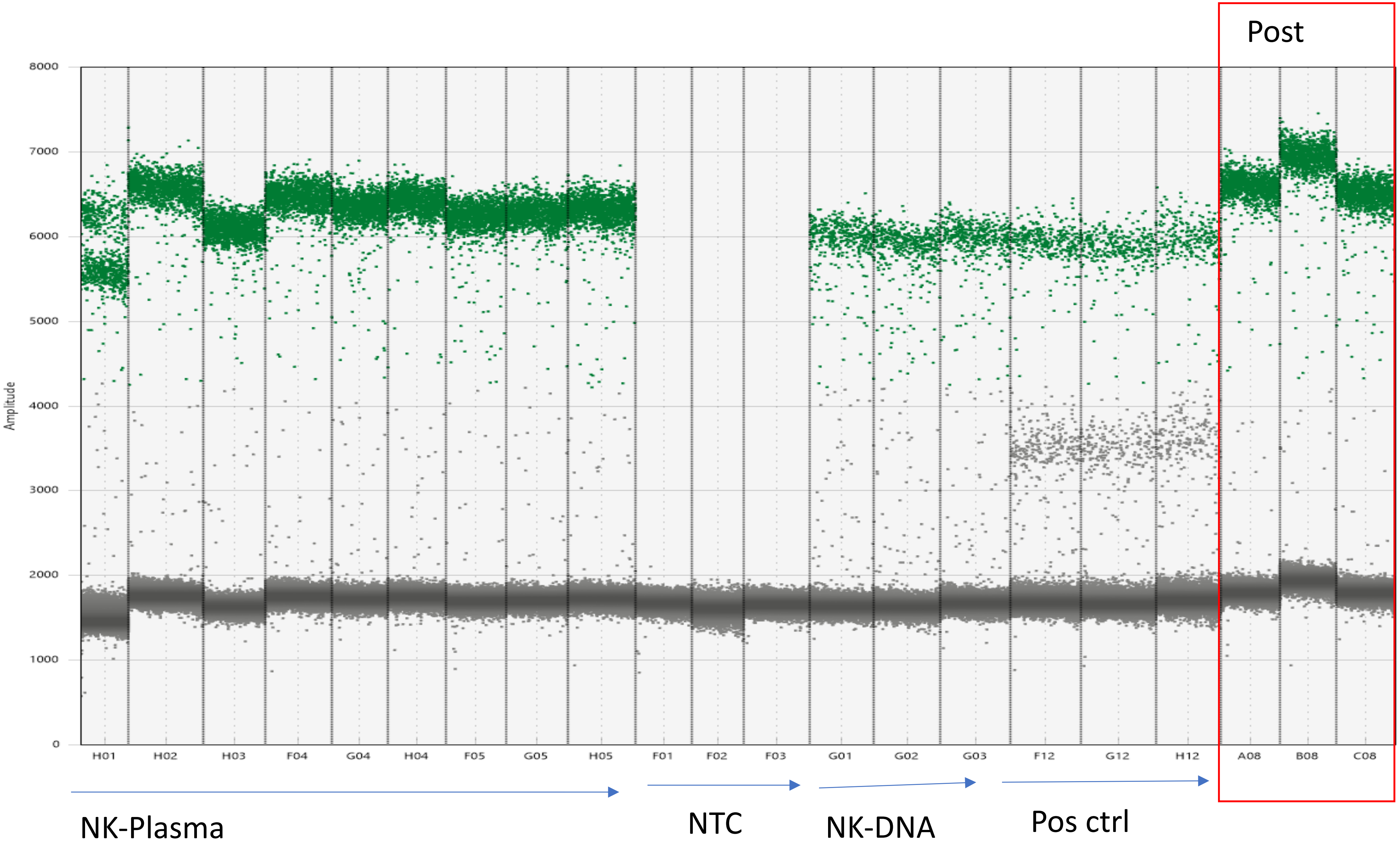

Positive control

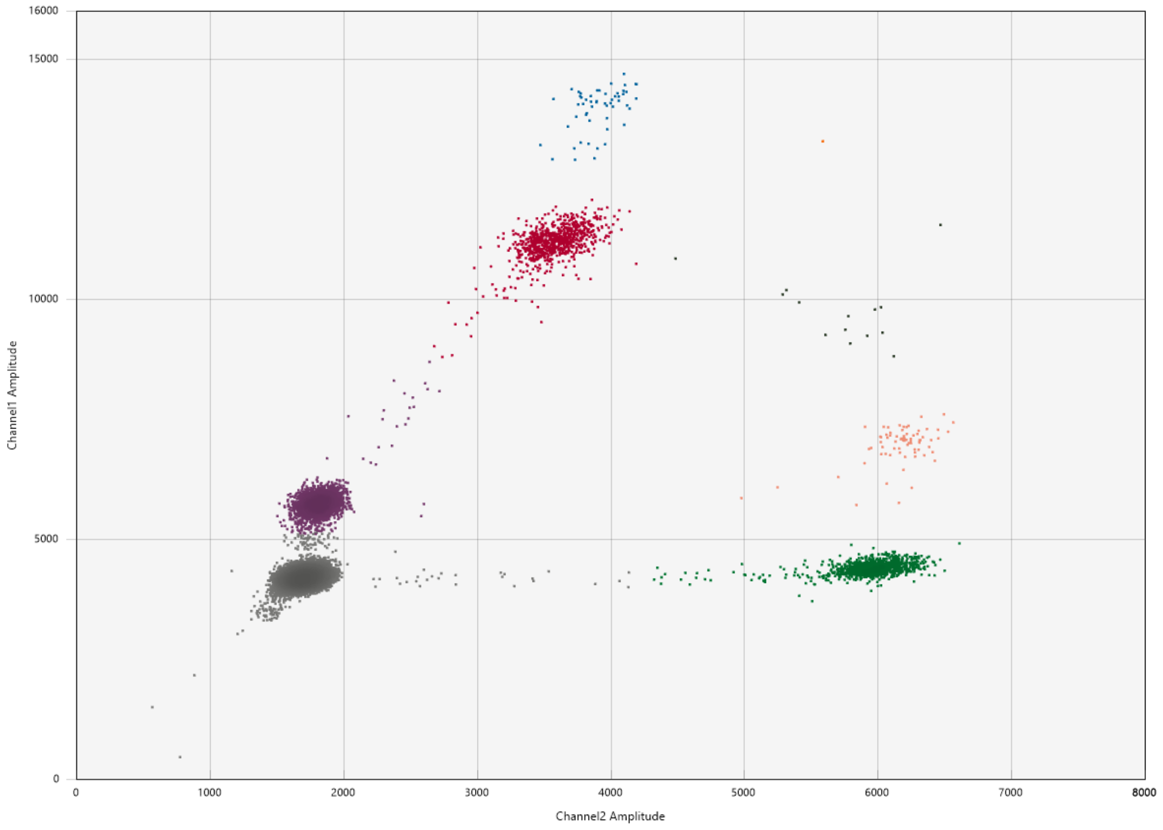

NK-Plasma

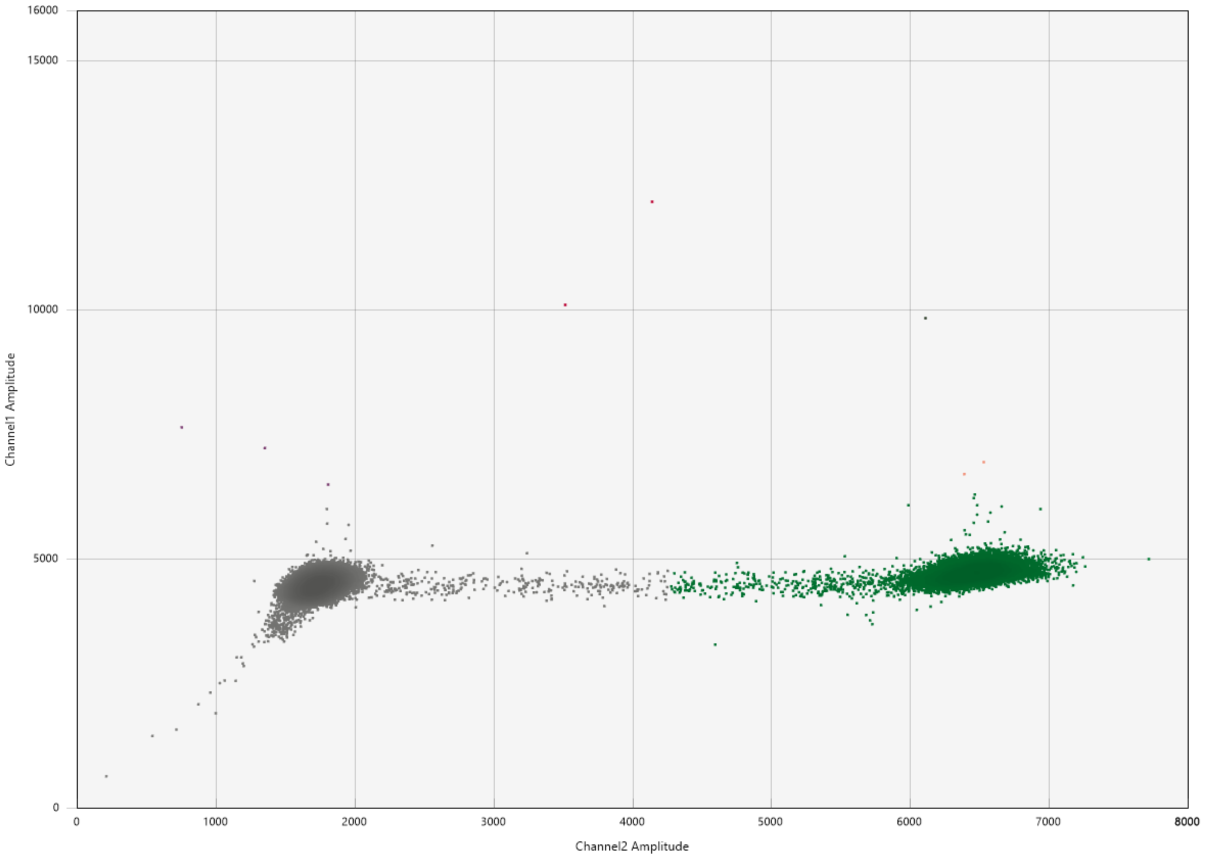

Pre-surgery

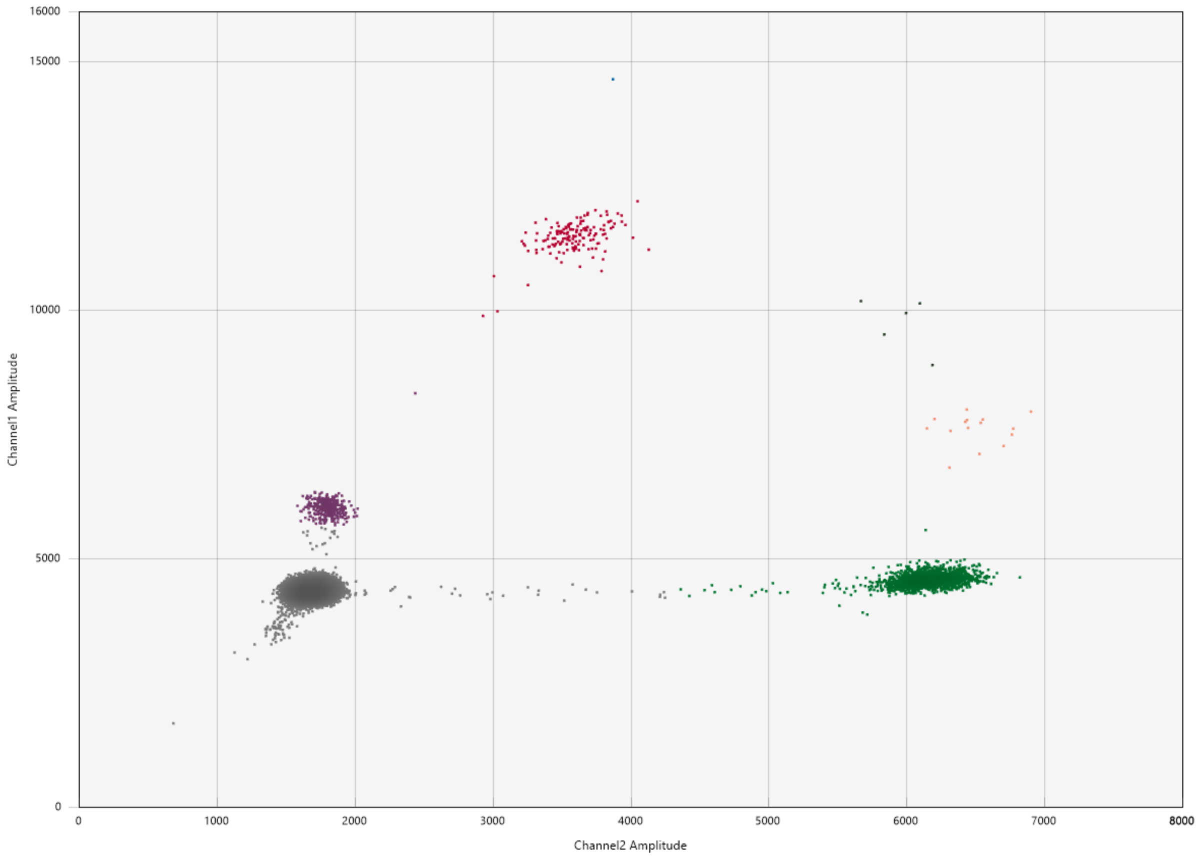

Follow-up 1

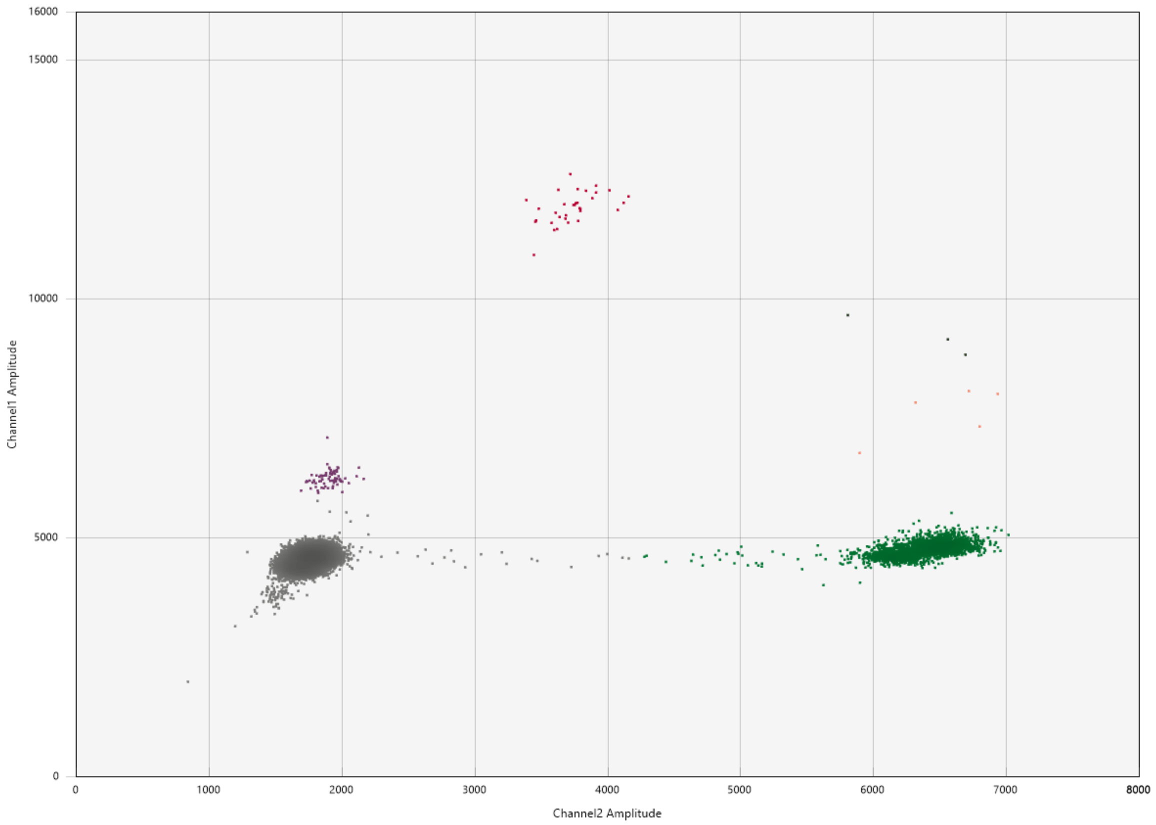

Post-surgery

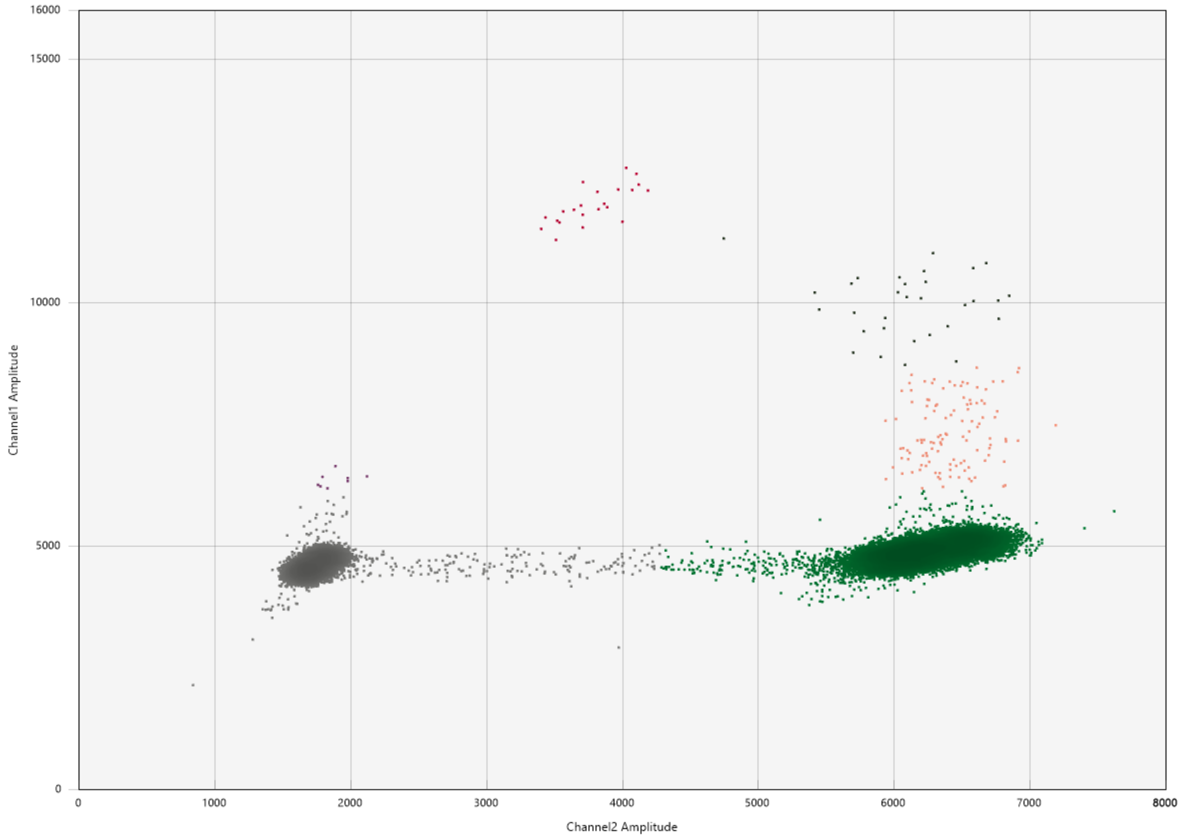

Post-surgery diluted

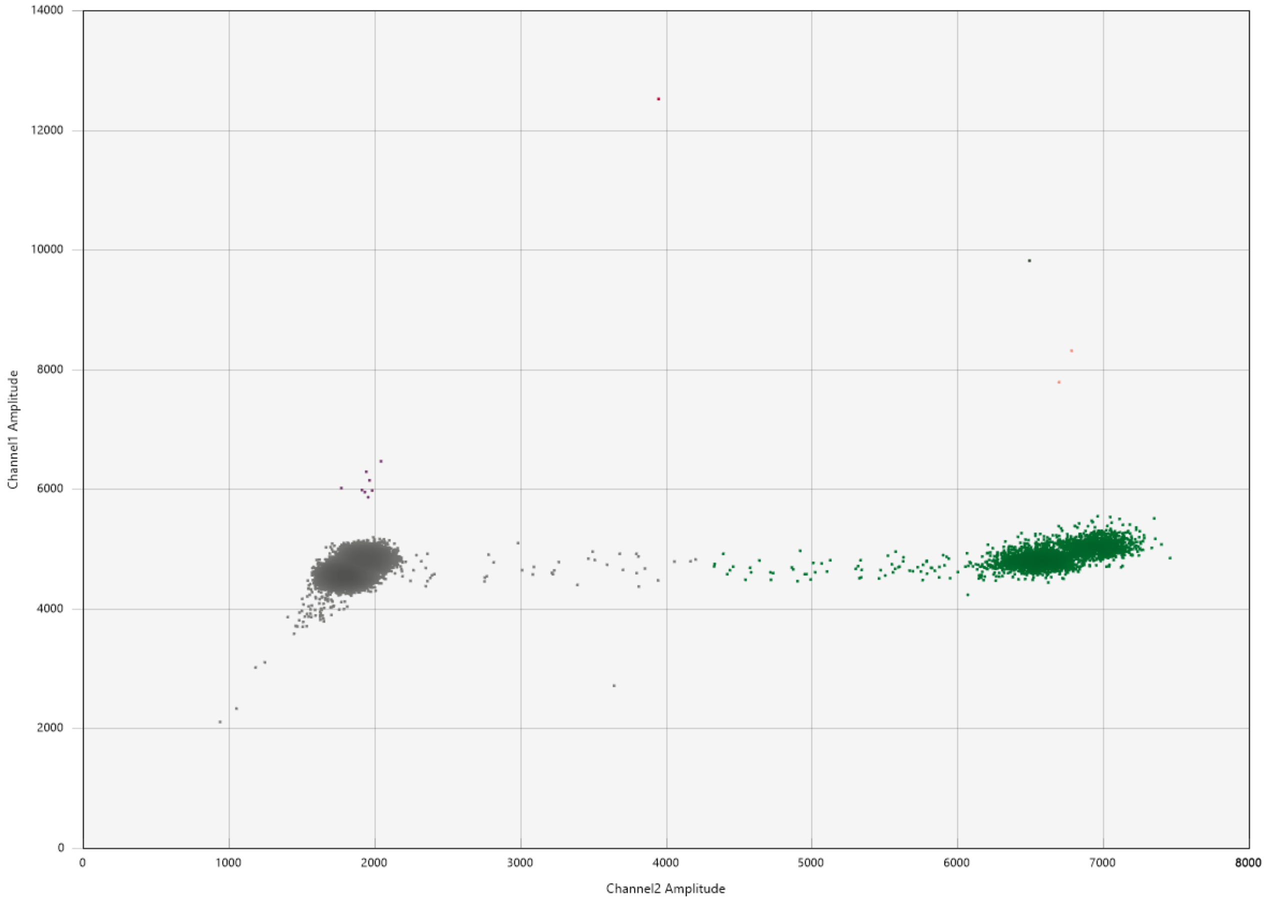

Concentration plots

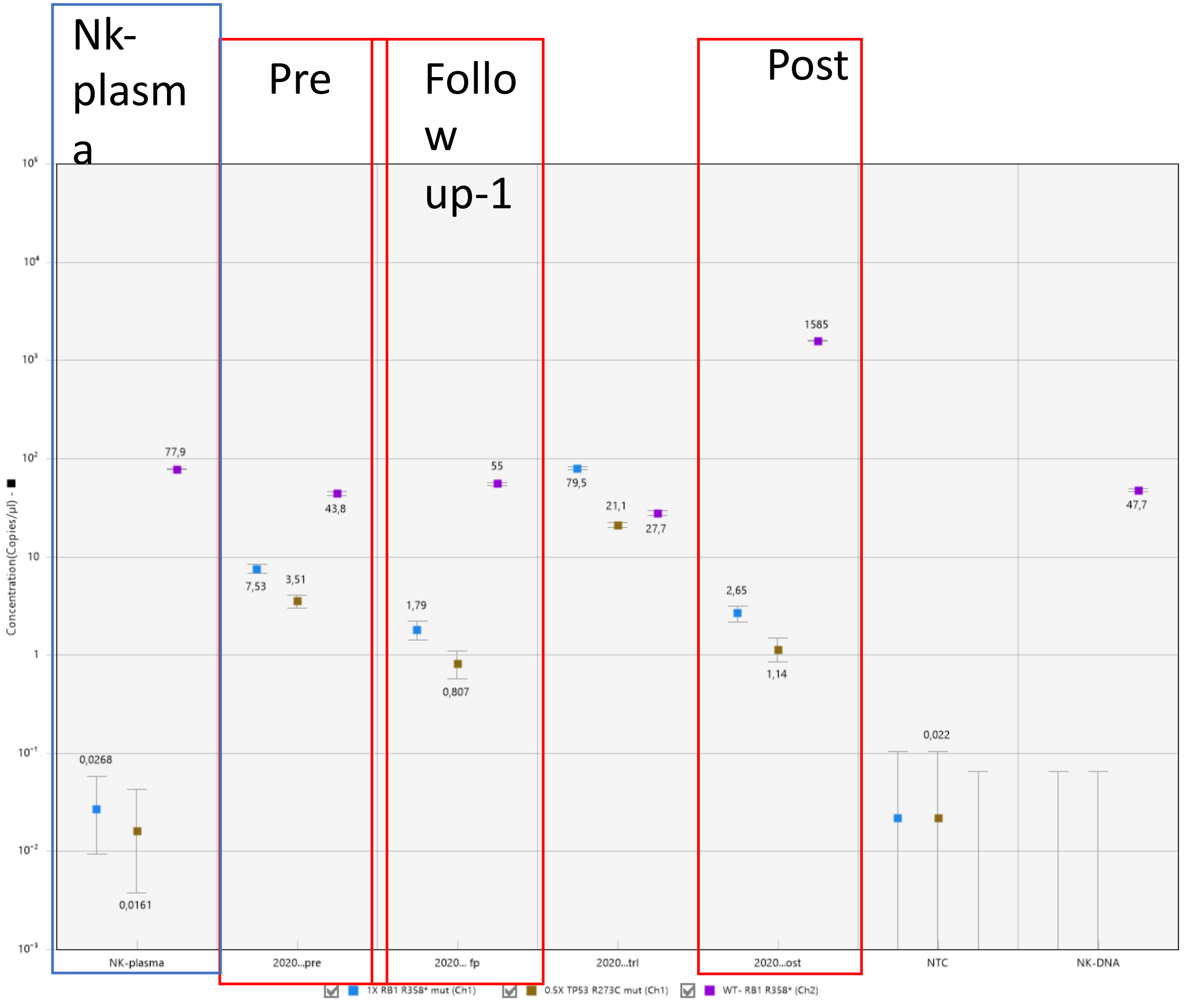

Post-surgery diluted

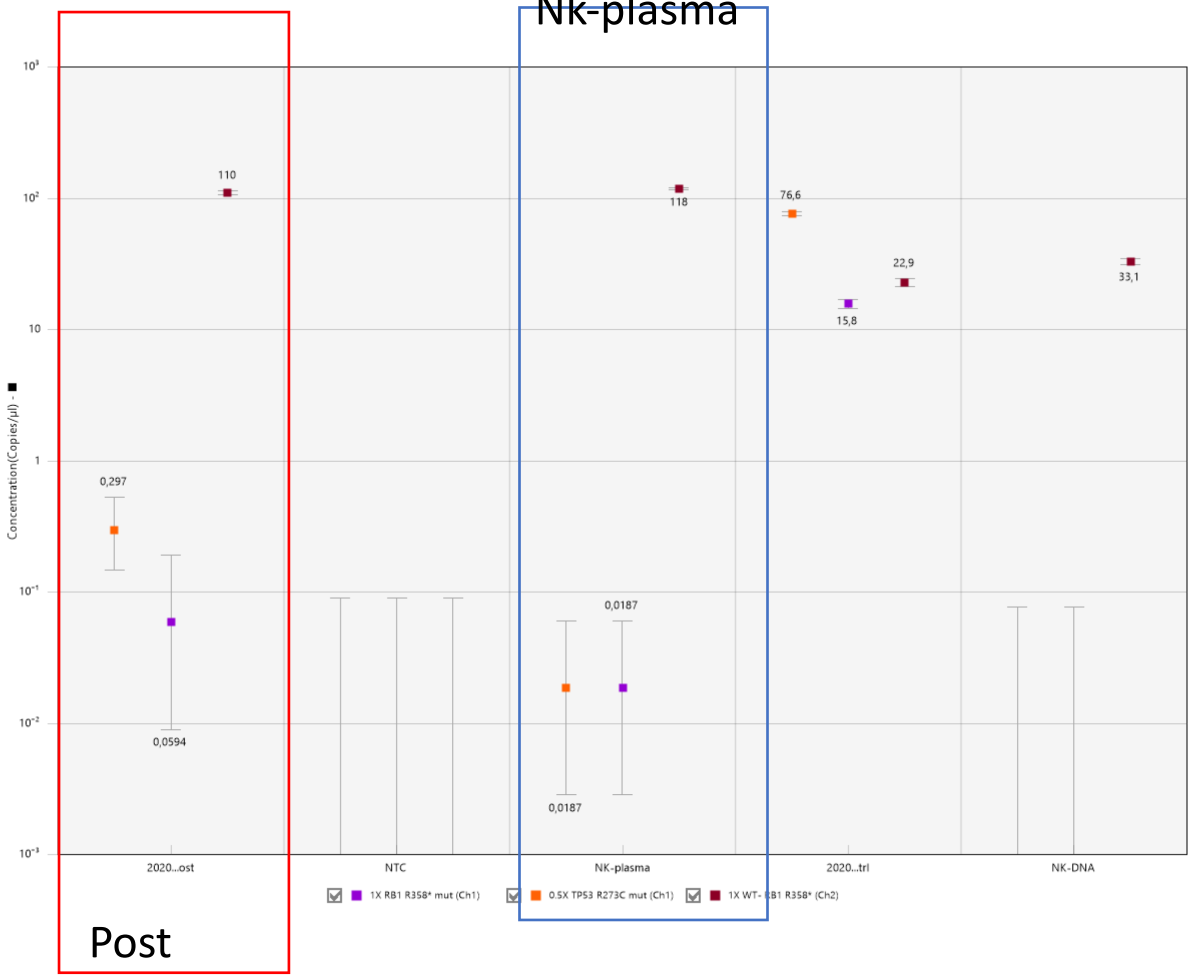

P25 - Final

Assays: 1X TP53,p.R196*mut (FAM) and 1X TP53 WT,p.R196* (HEX)

Gene target :TP53,p.R196*,c.586C>T

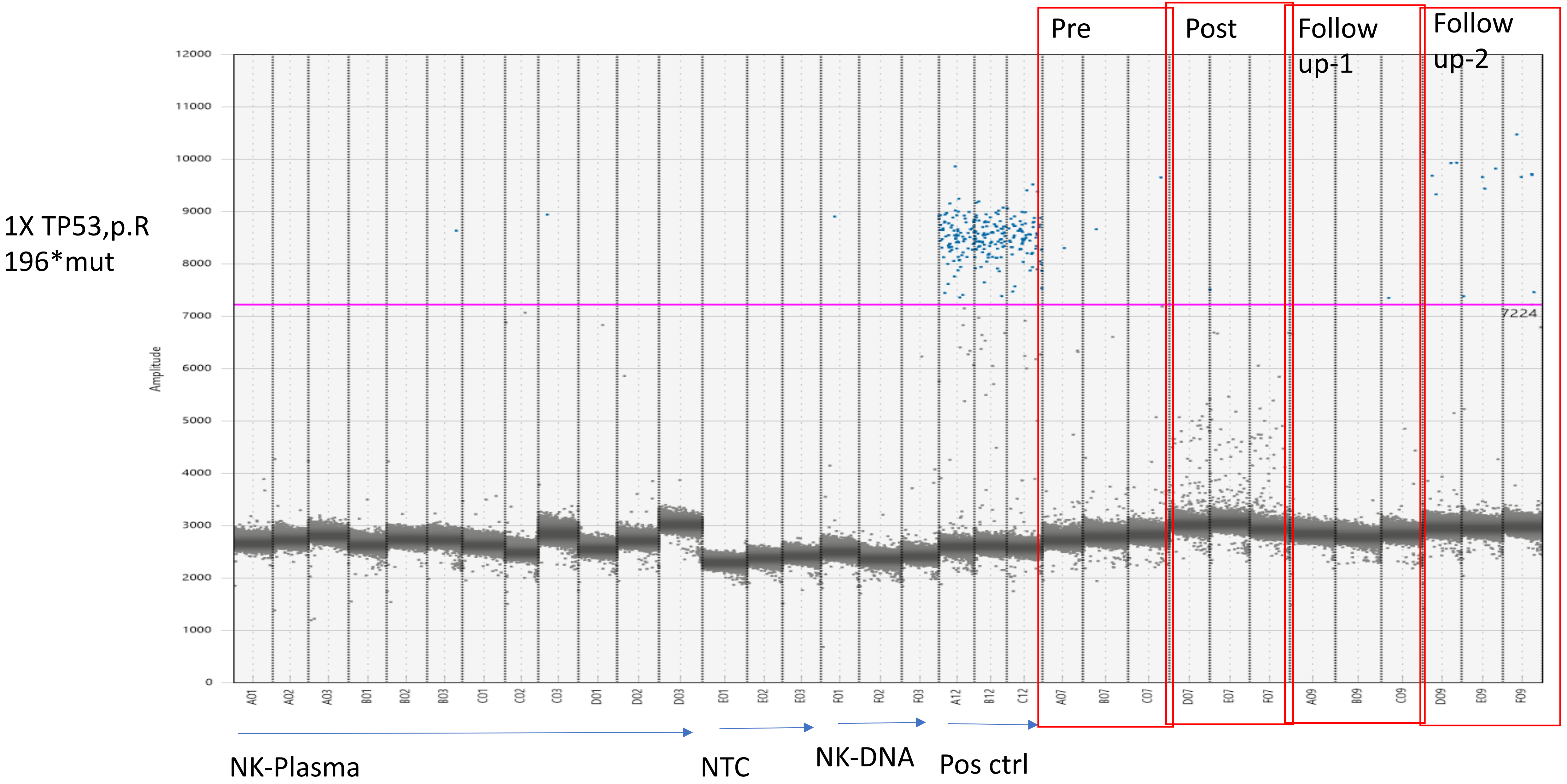

P25 - Final-WT

Assays: 1X TP53 WT,p.R196* (HEX)

Gene target :TP53,p.R196*,c.586C>T

1X TP53 WT ,p.R196*

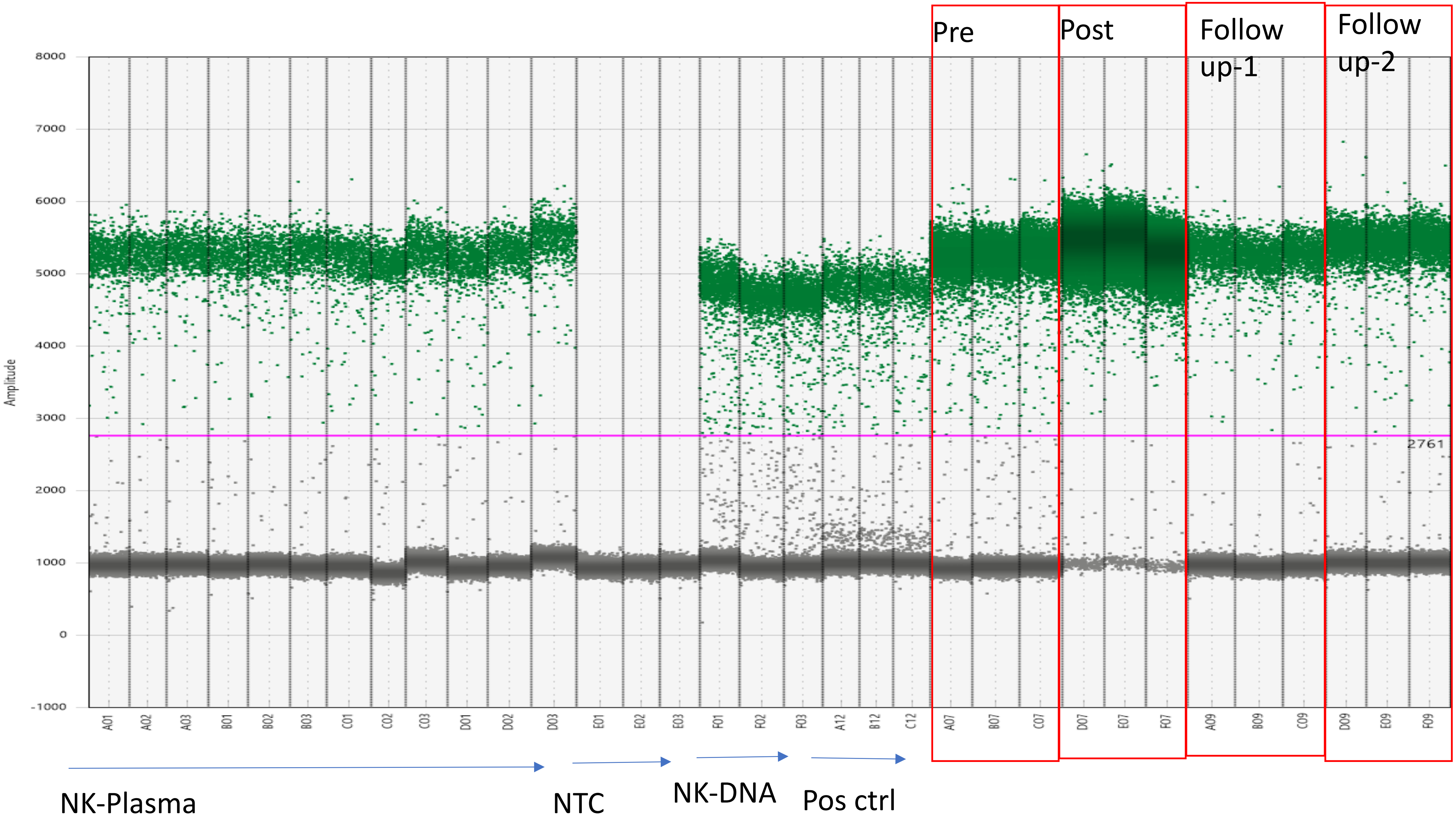

P25 - Final- diluted

Assays: 1X TP53,p.R196*mut (FAM) and 1X TP53 WT,p.R196* (HEX)

Gene target :TP53,p.R196*,c.586C>T

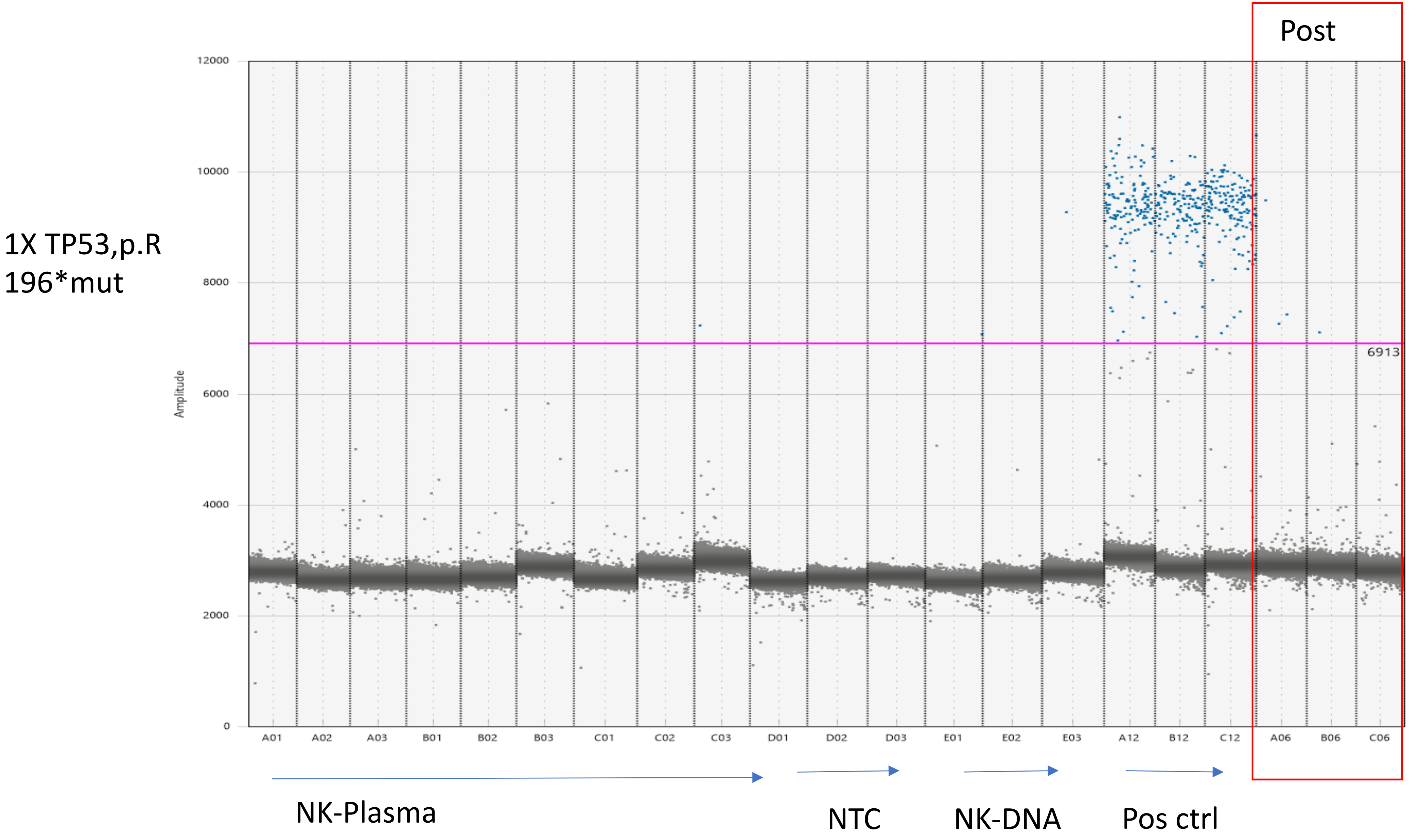

P25 - Final diluted

Assays: 1X TP53 WT,p.R196* (HEX)

Gene target :TP53,p.R196*,c.586C>T

1X TP53 WT,p.R196*

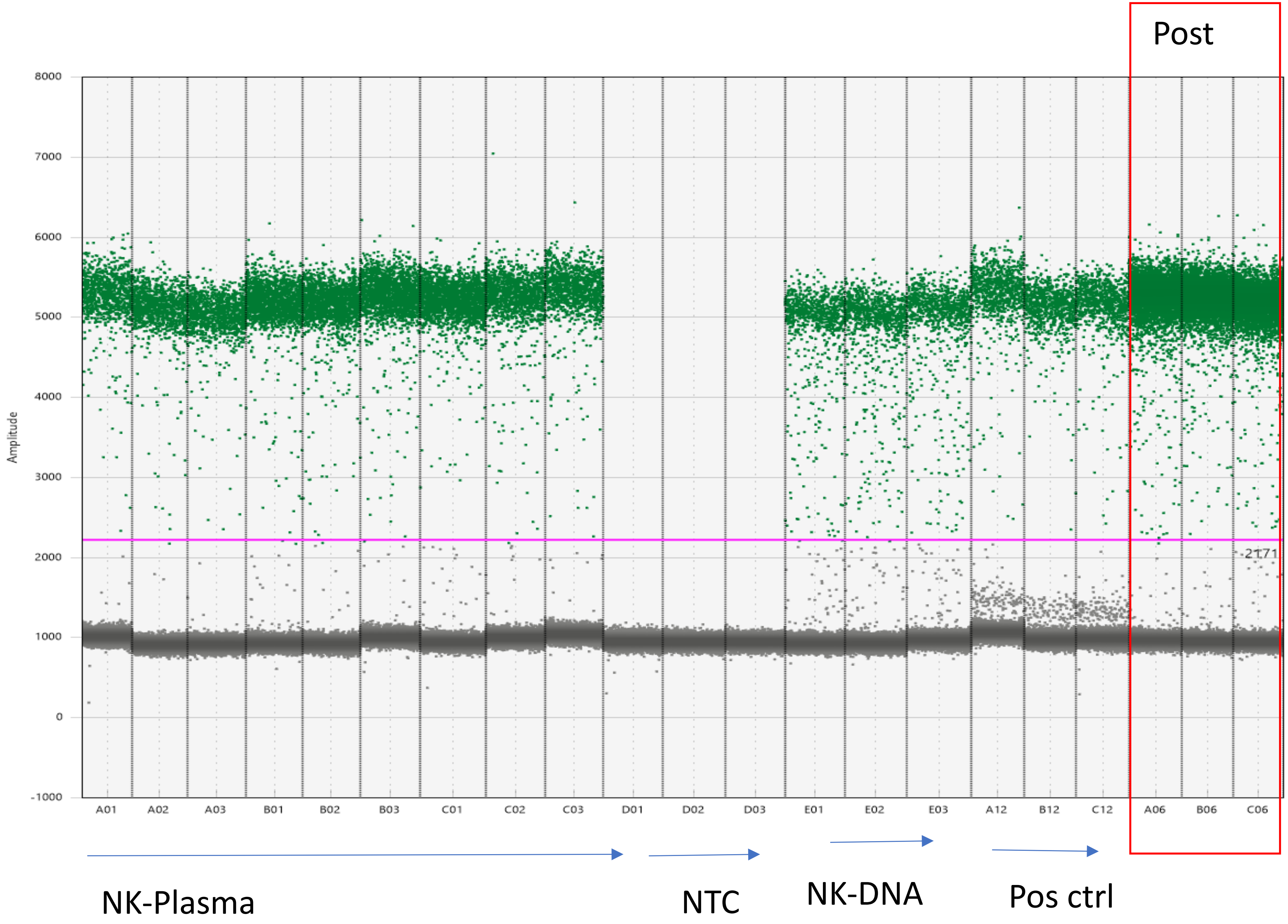

Positive control

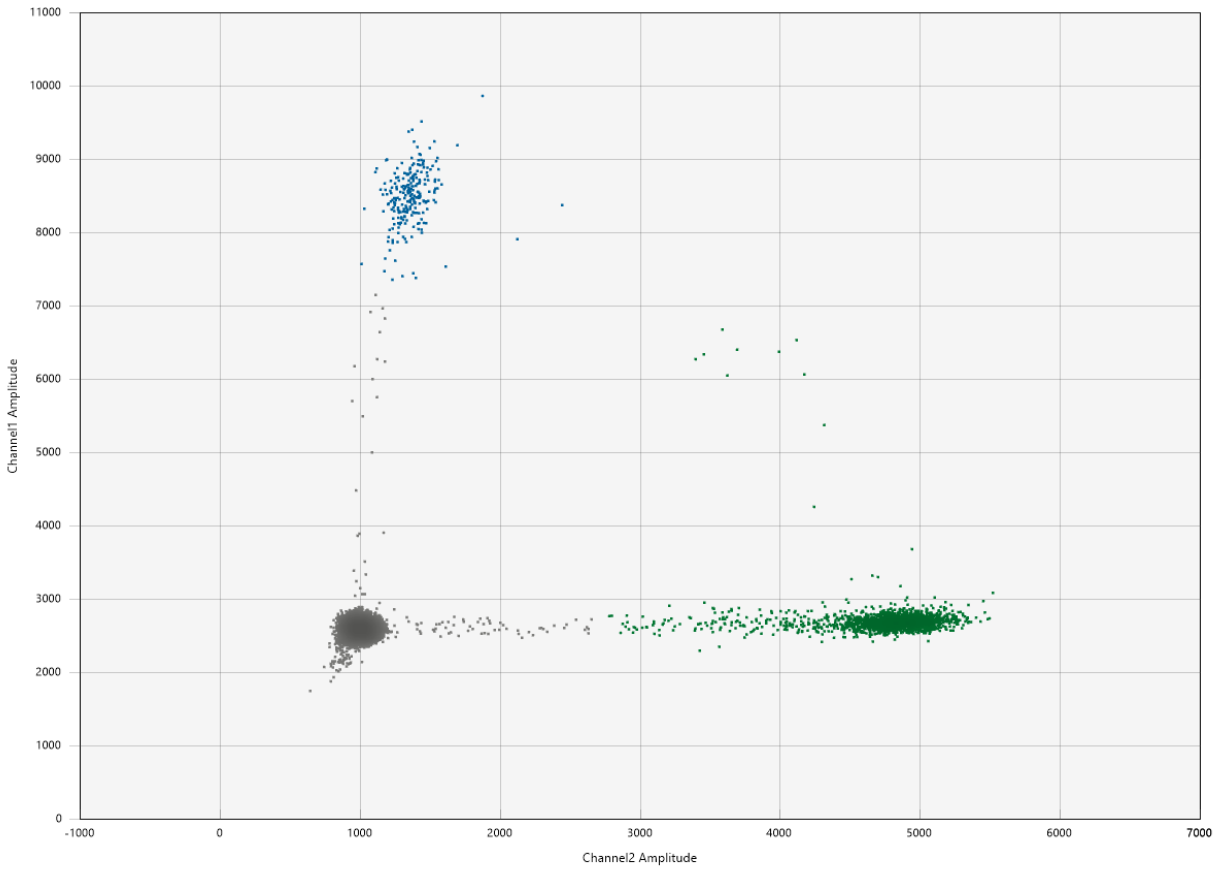

NK-plasma cfDNA

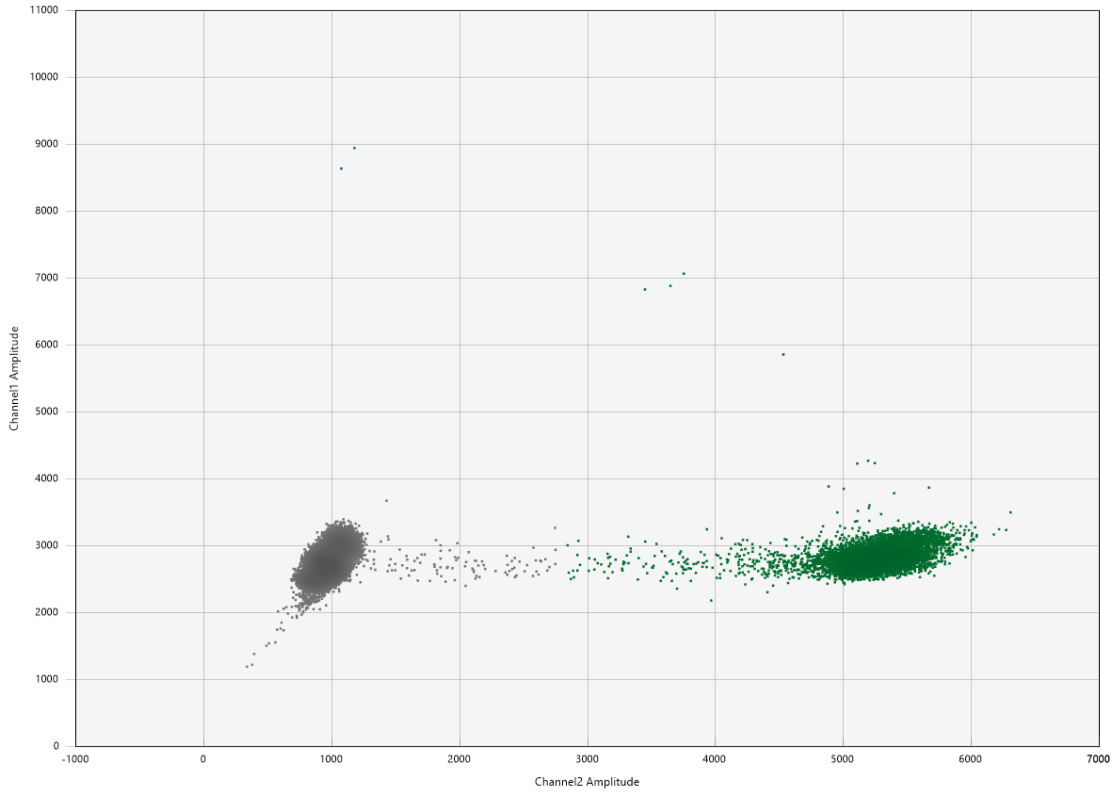

Pre-surgery

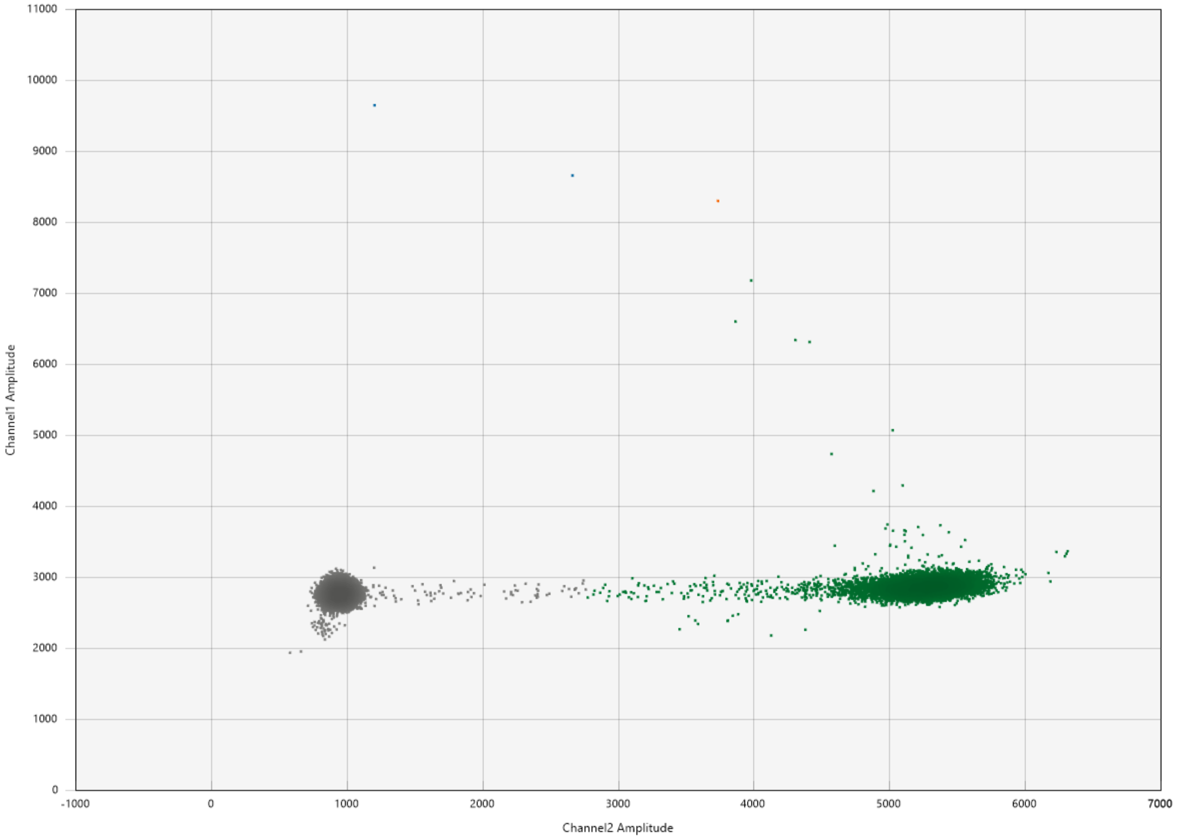

Post-surgery

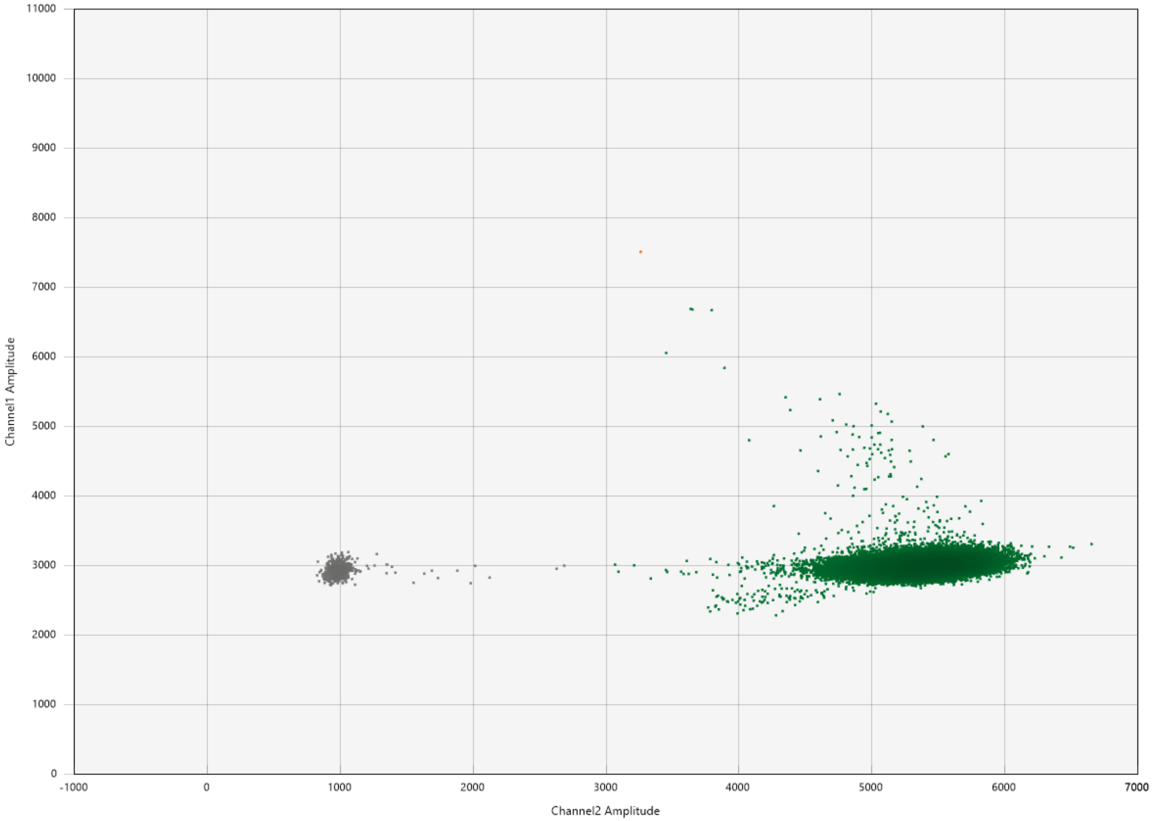

Post-surgery - diluted

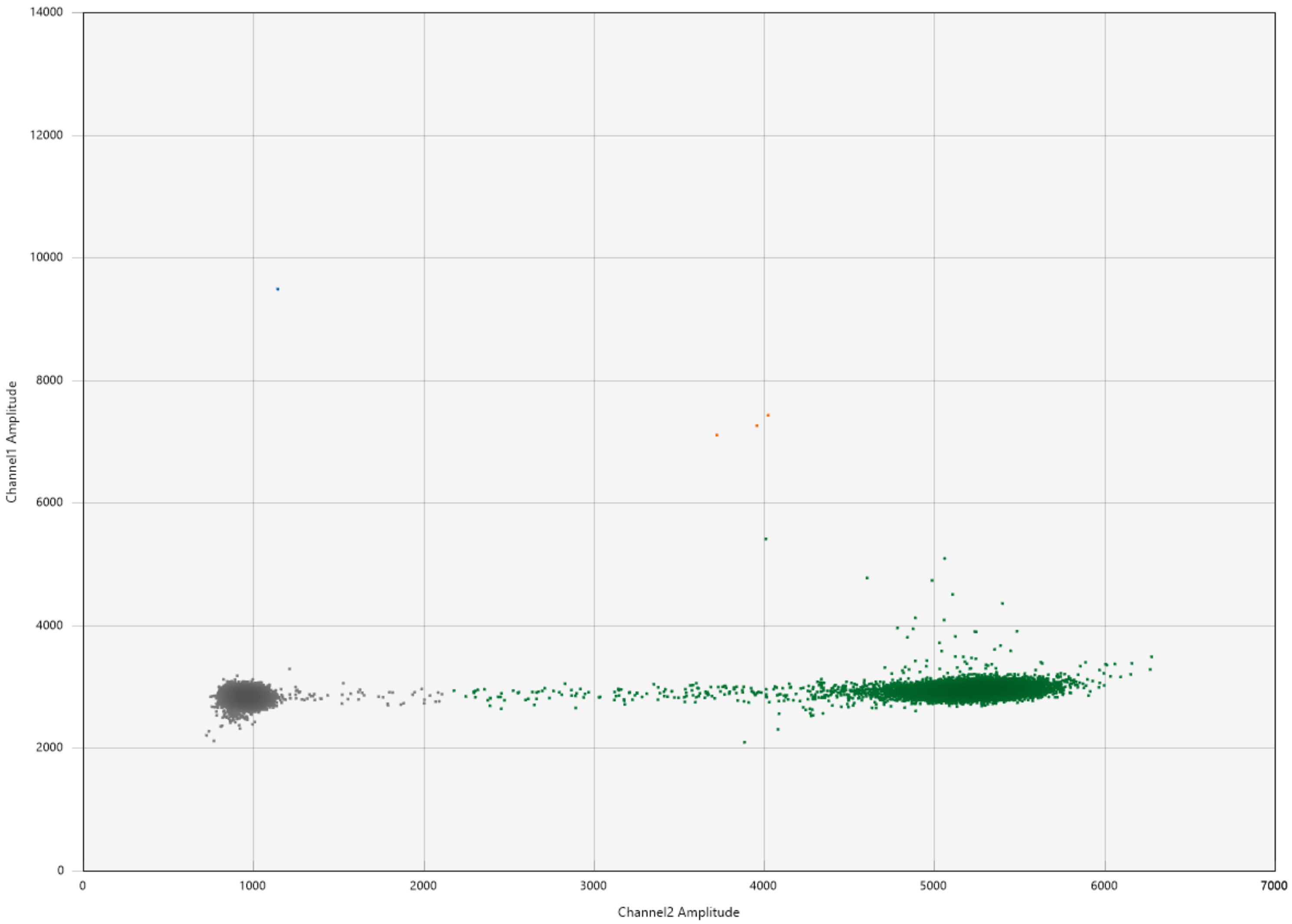

Follow up 1

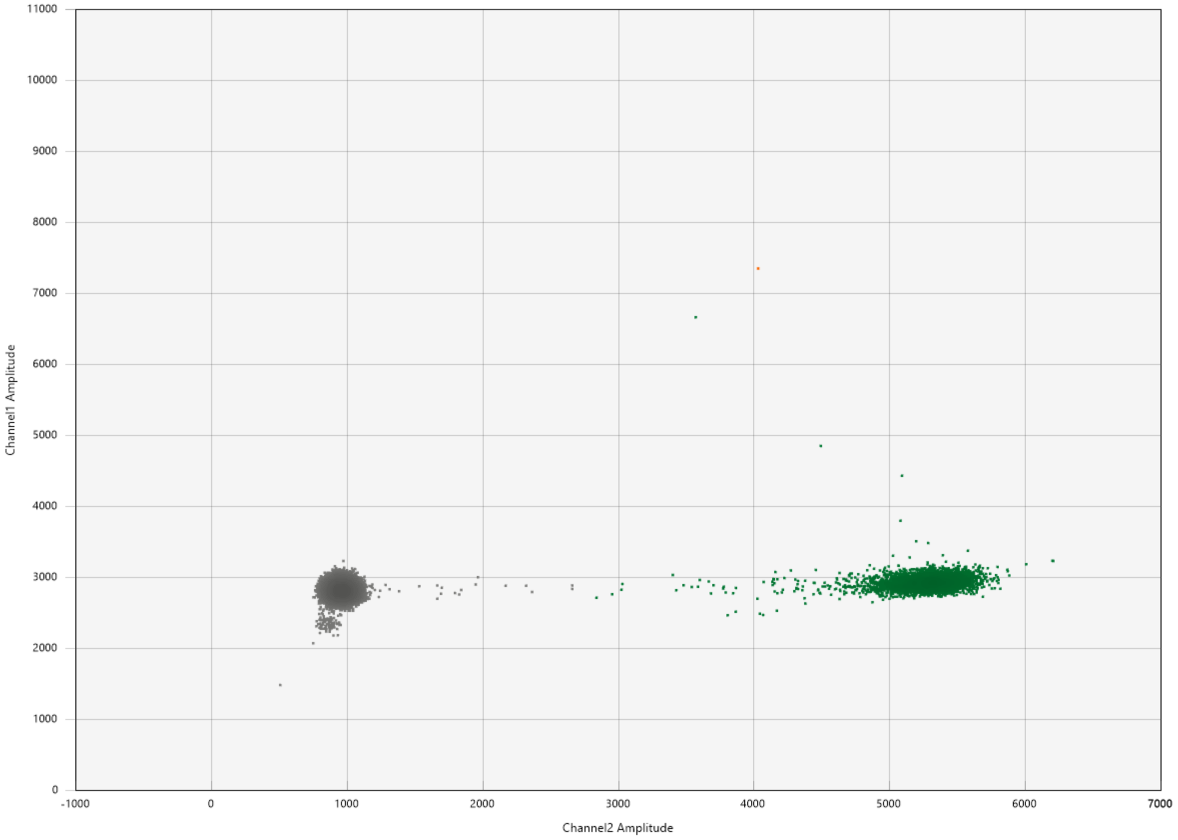

Follow up 2

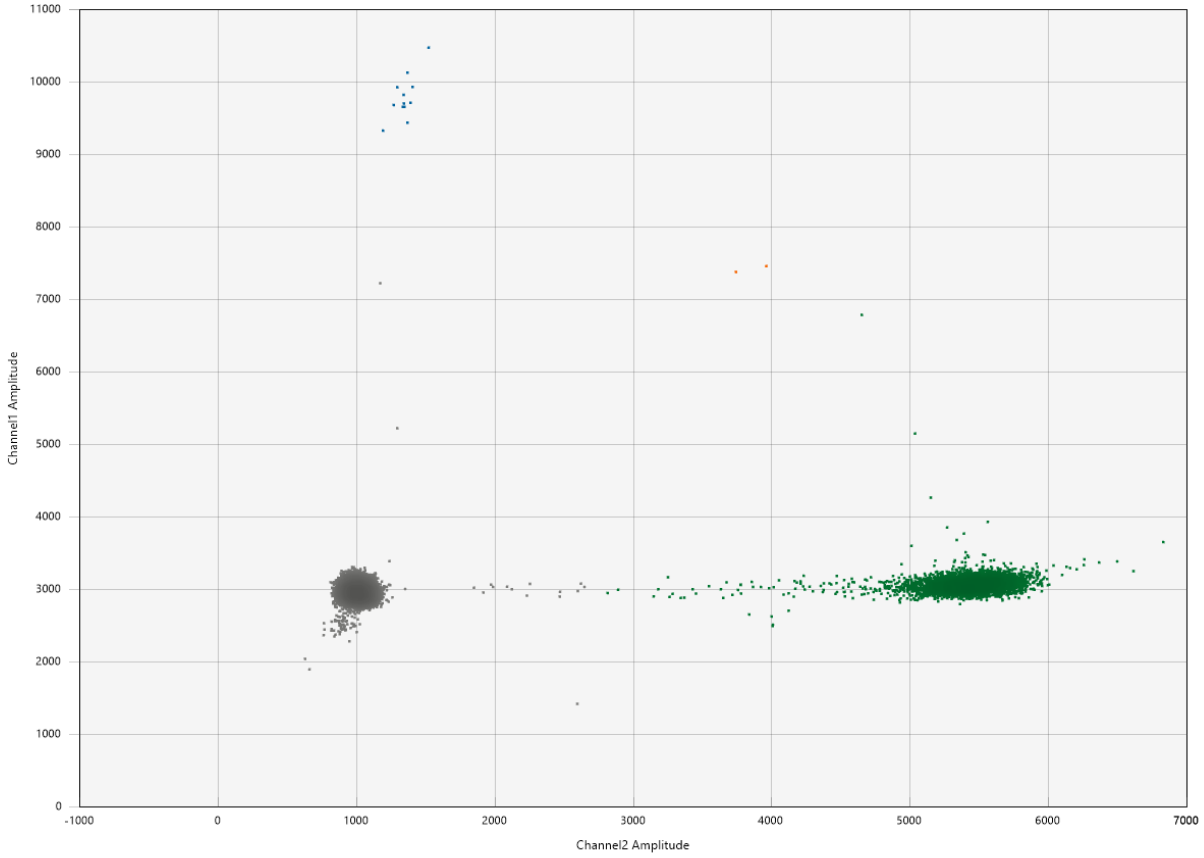

Concentration plots Pre ,Post-surgery and Follow-up1 and 2

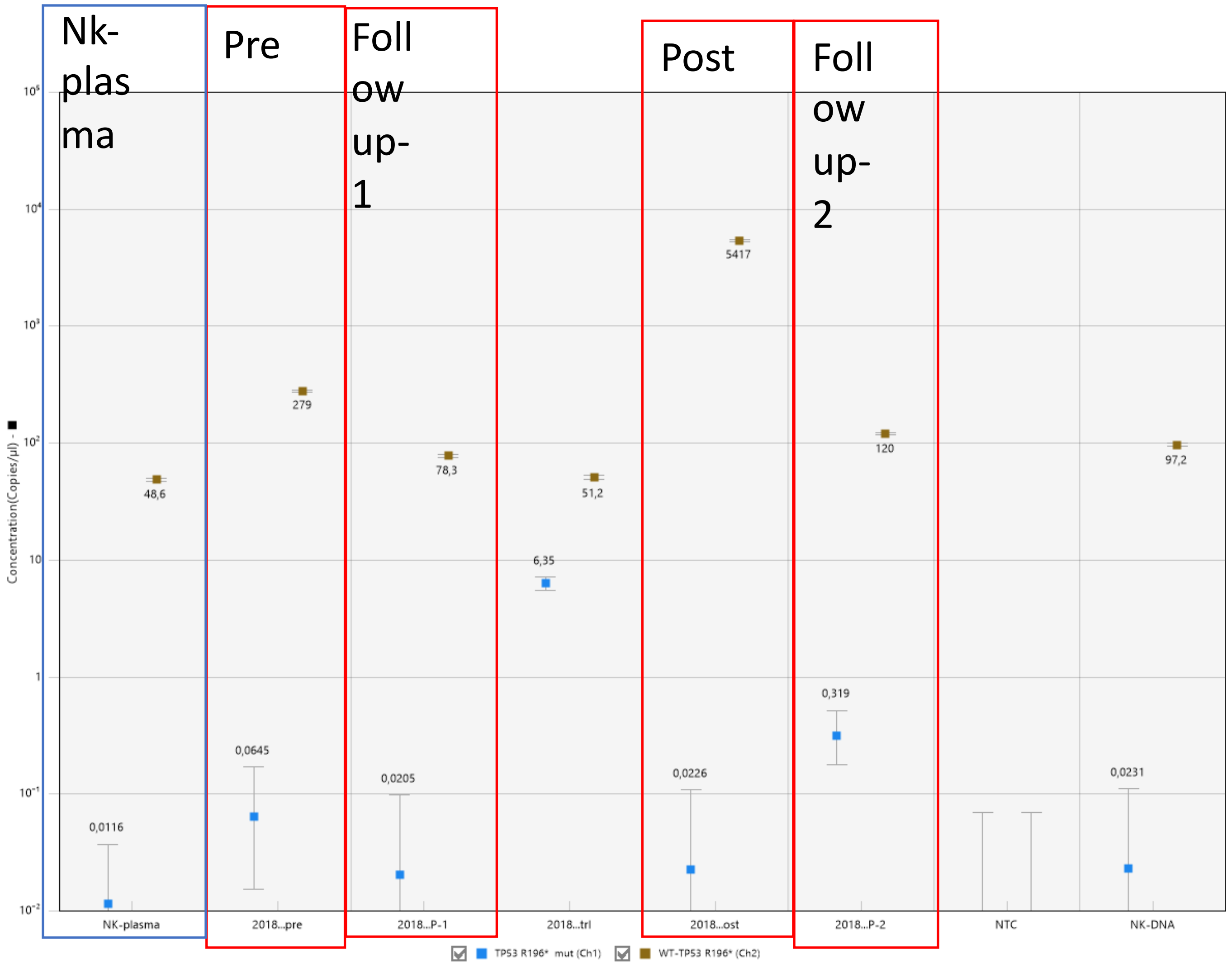

Post-dilution

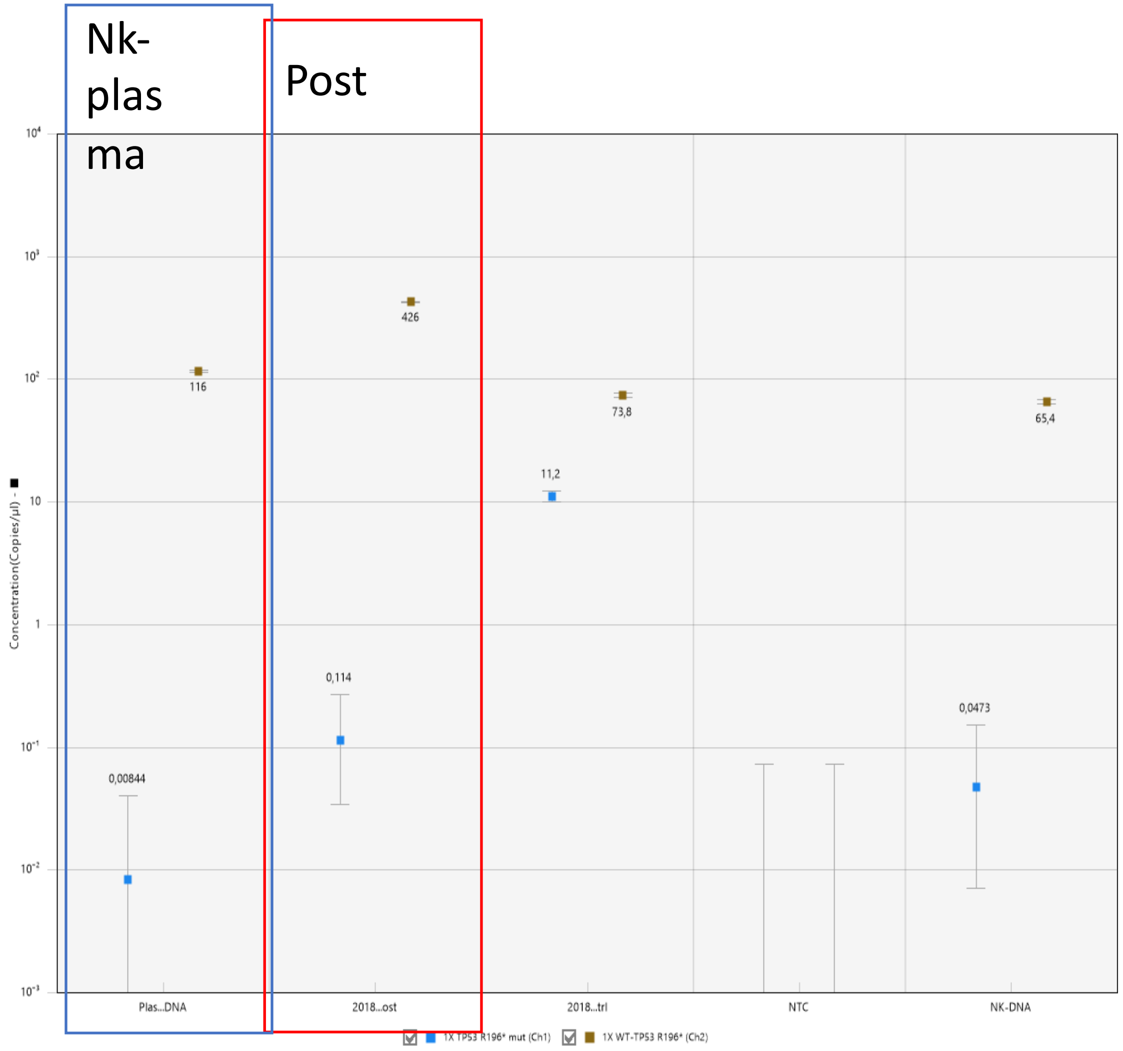

P15 - Final

Gene targets : TP53,p.R248Q, c.743G>A and ERBB2,p.S310Y, c.929C>A

Assays: 1X TP53,p.R248Q mut (FAM) and 1X TP53 WT,p.R248Q (HEX)
0.5X ERBB2,p.S310Y mut (FAM) and 1X dark probe for ERBB2,p.S310Y,

1X TP53,p.R248 Q mut

0.5X ERBB2,p.S310 Y mut

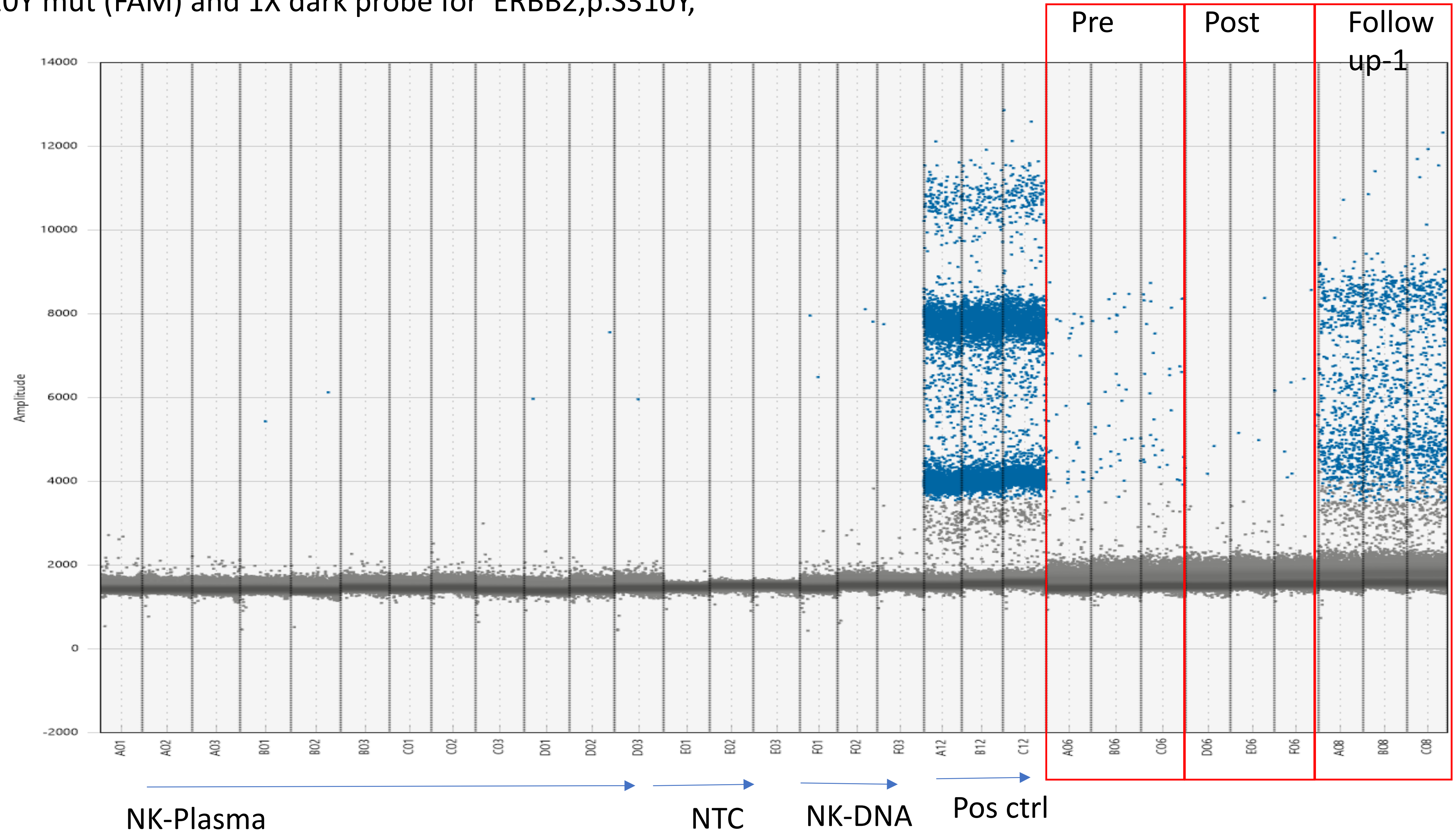

P15 - Final-WT

Gene targets : TP53,p.R248Q, c.743G>A and ERBB2,p.S310Y, c.929C>A

Assays:1X TP53 WT,p.R248Q (HEX) and 1X dark probe for ERBB2,p.S310Y,

1X TP53 WT,p.R248Q mut

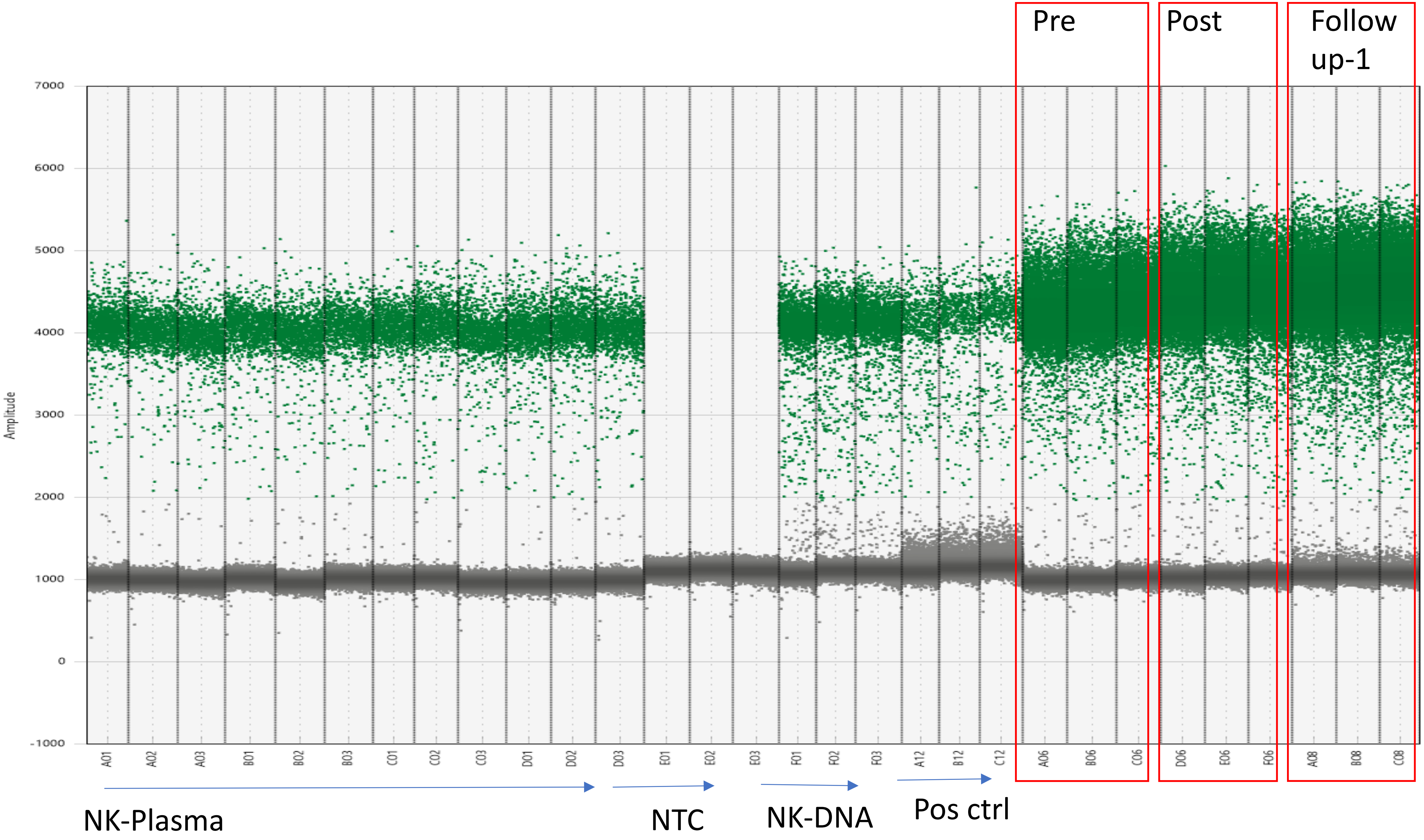

P15 Diluted

Gene targets : TP53,p.R248Q, c.743G>A and ERBB2,p.S310Y, c.929C>A

1X TP53,p.R248 Q mut

0.5X ERBB2,p.S310 Y mut

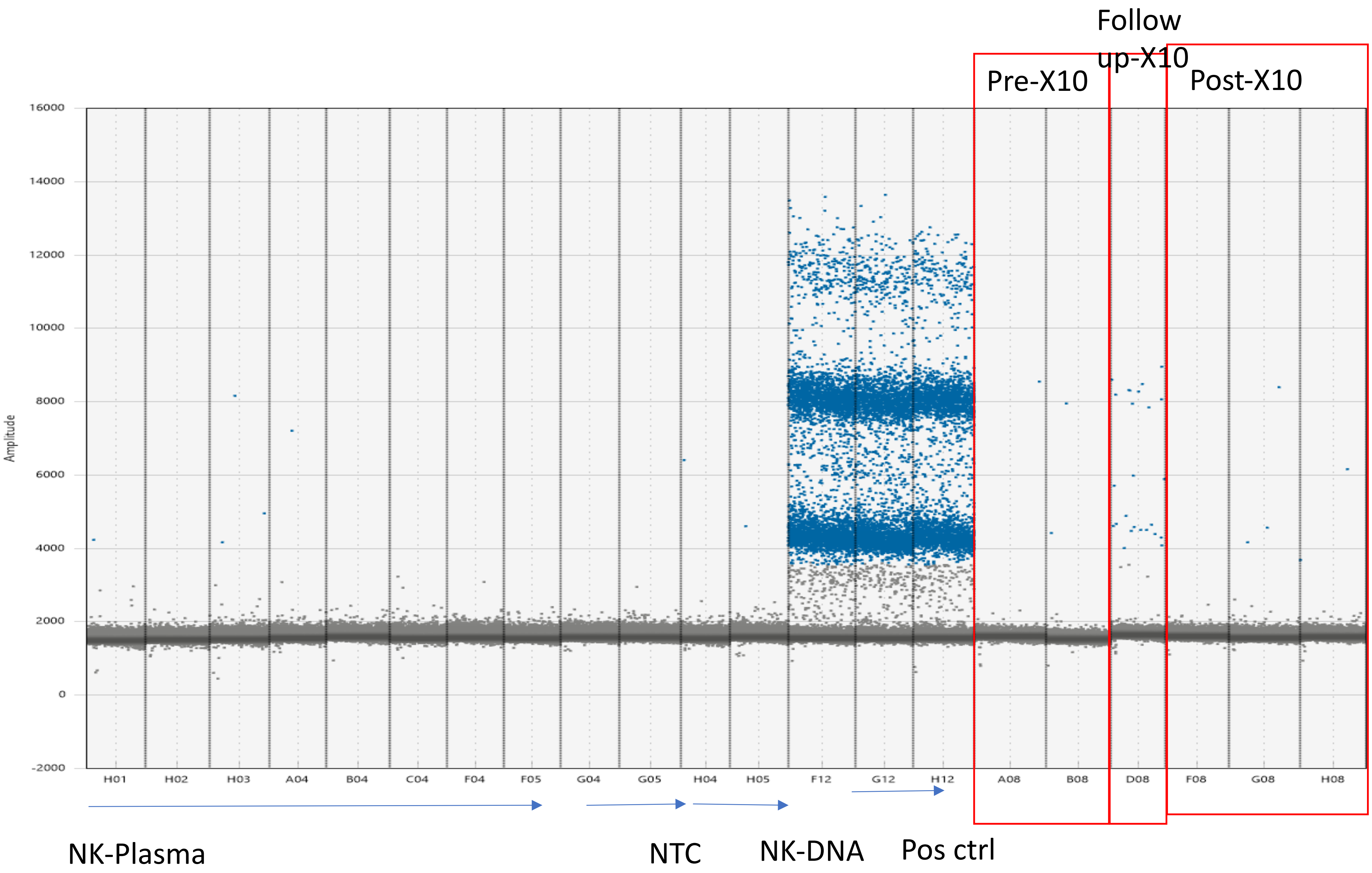

P15 - Final diluted

Gene targets : TP53,p.R248Q, c.743G>A and ERBB2,p.S310Y, c.929C>A

Assays: 1X TP53 WT,p.R248Q (HEX) and 1X dark probe for ERBB2,p.S310Y

1X TP53 WT,p.R248Q

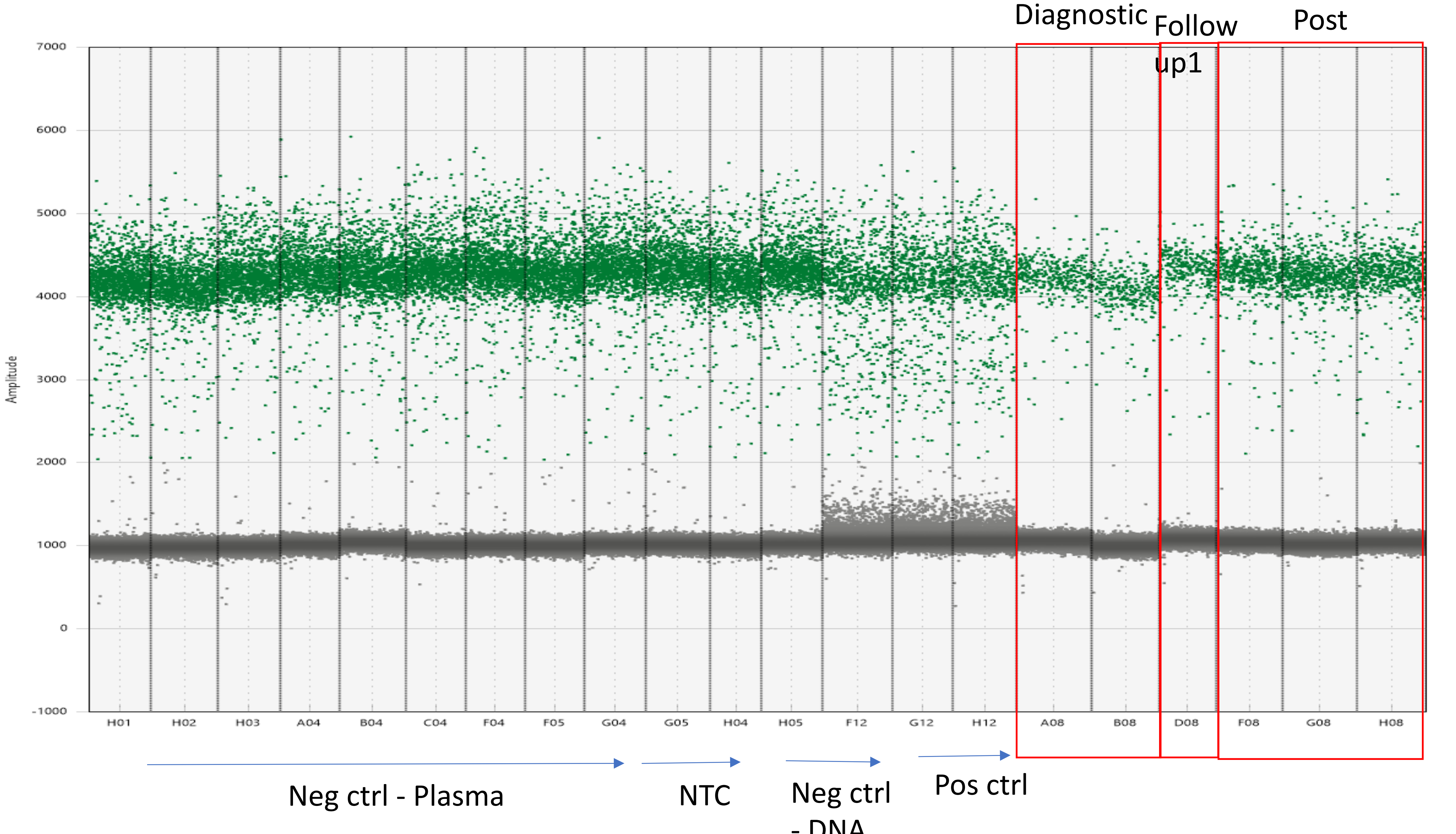

Positive control

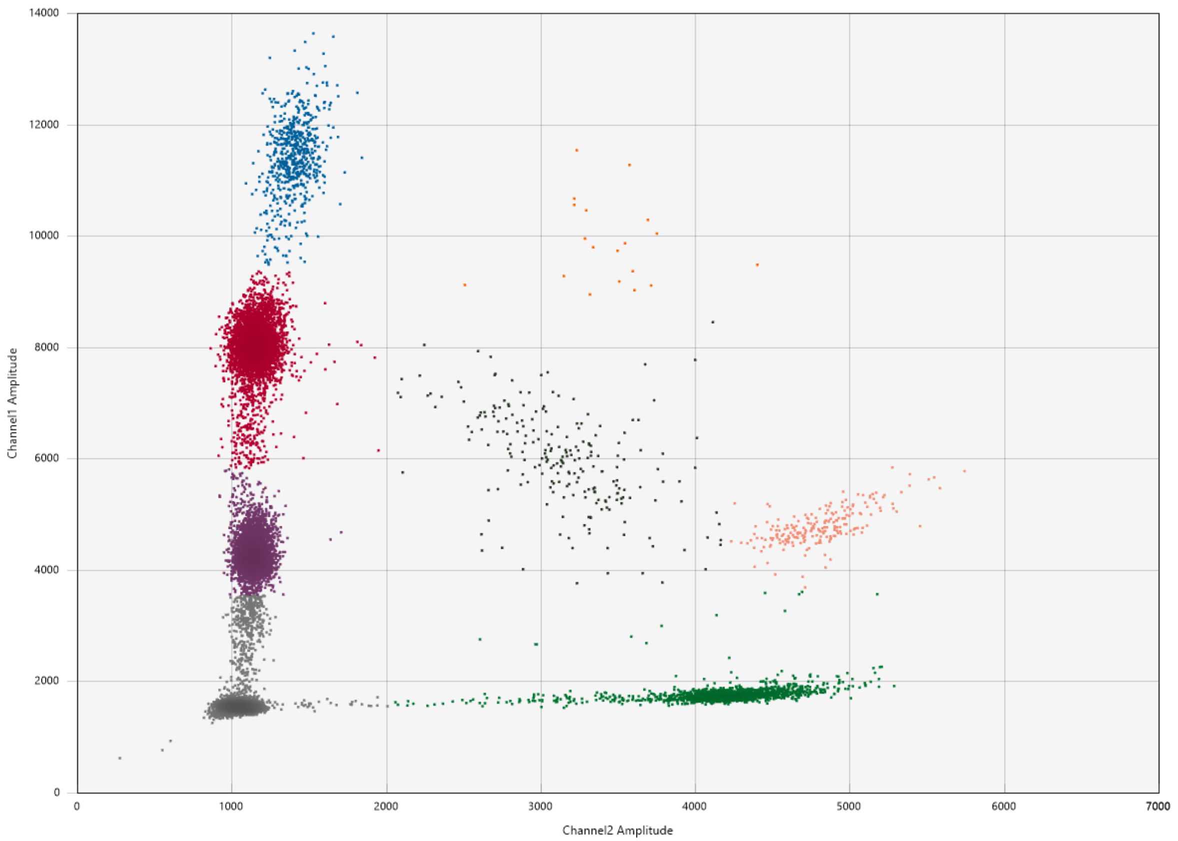

NK-plasma

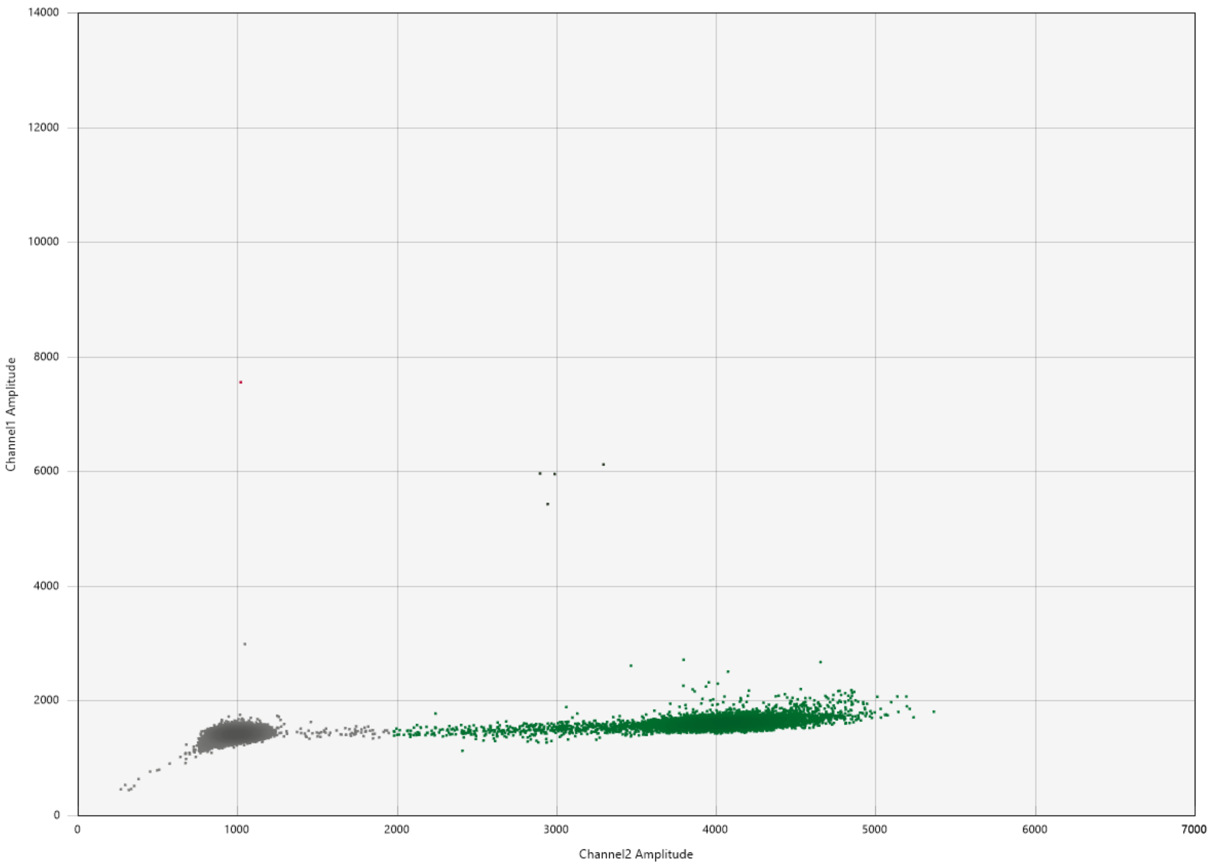

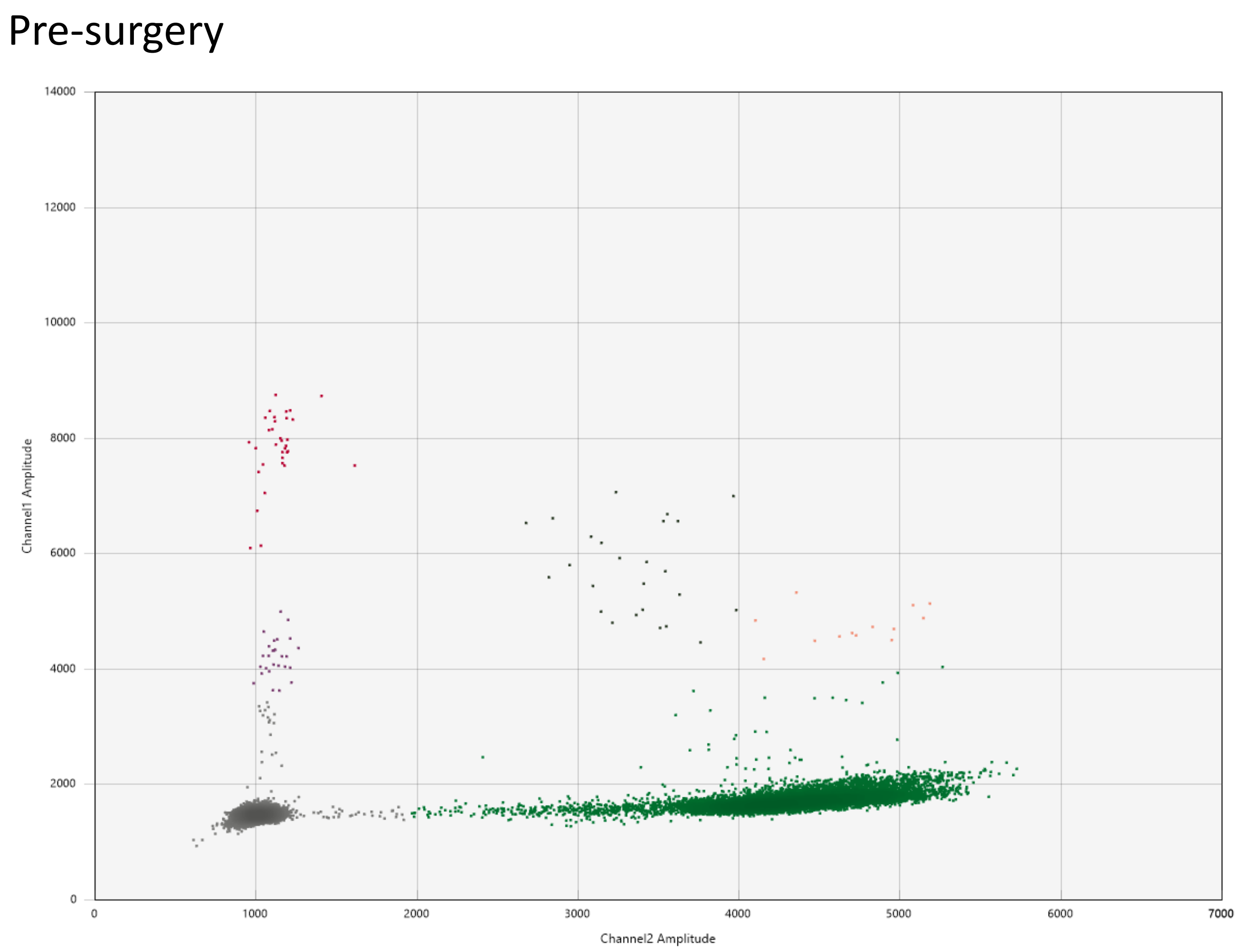
Pre-surgery
Channel1 Amplitude
Channel2 Amplitude
0
2000
4000
6000
8000
10000
12000
14000
0
1000
2000
3000
4000
5000
6000
7000

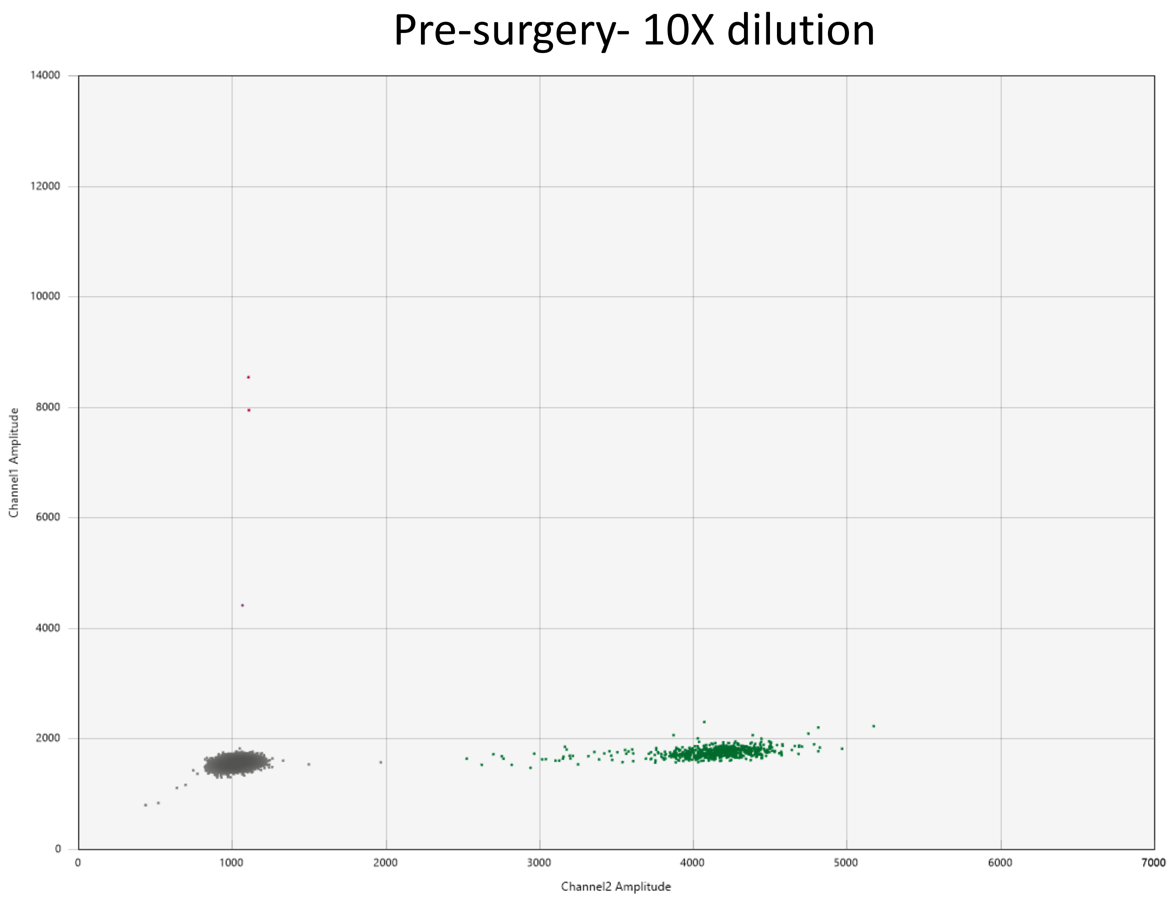
Pre-surgery- 10X dilution
Channel1 Amplitude
Channel2 Amplitude
0
2000
4000
6000
8000
10000
12000
14000
0
1000
2000
3000
4000
5000
6000
7000

Post-surgery

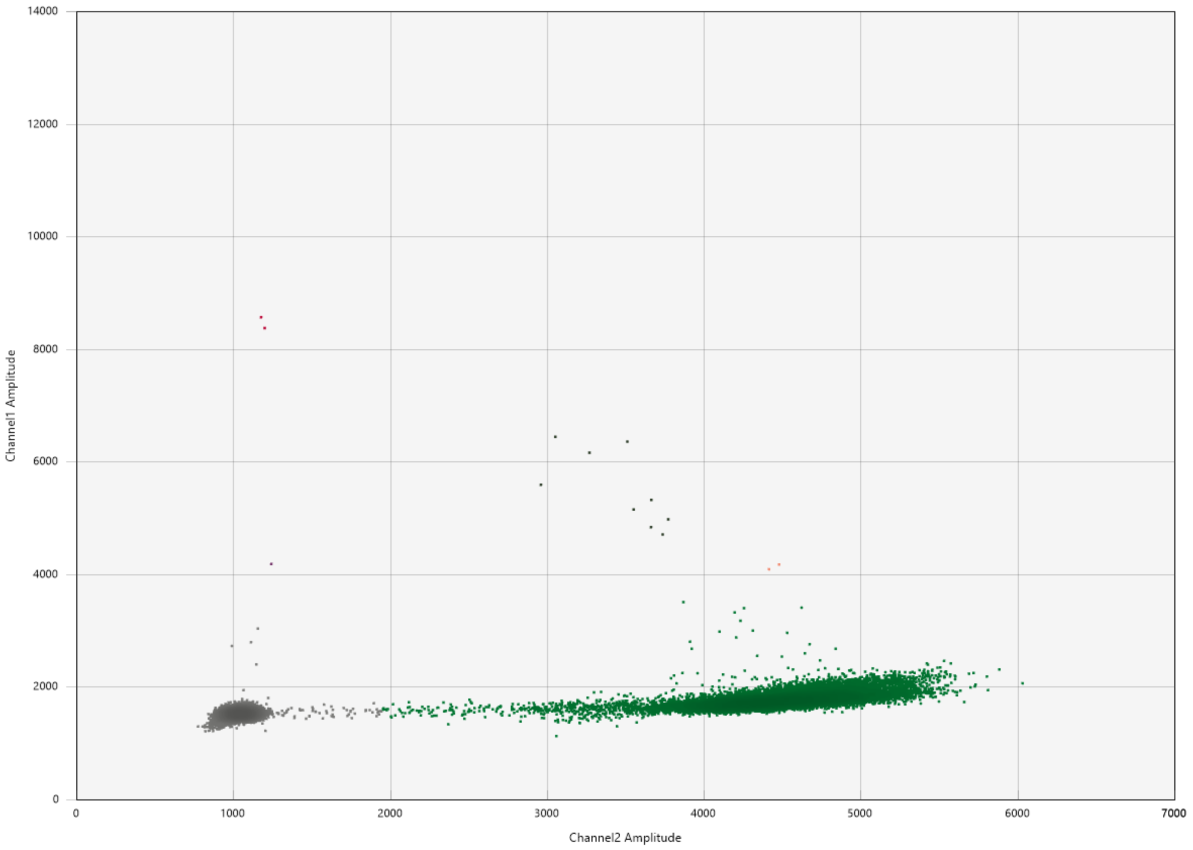

Post-surgery-10X dilution

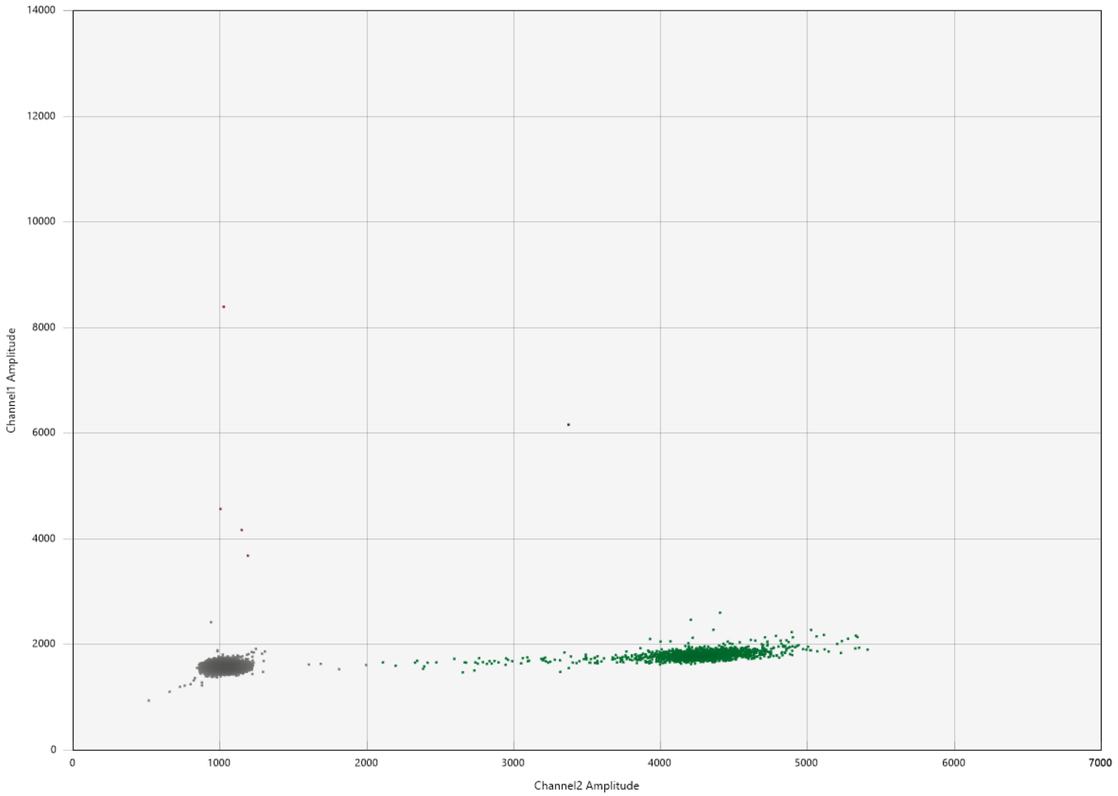

follow-up 1

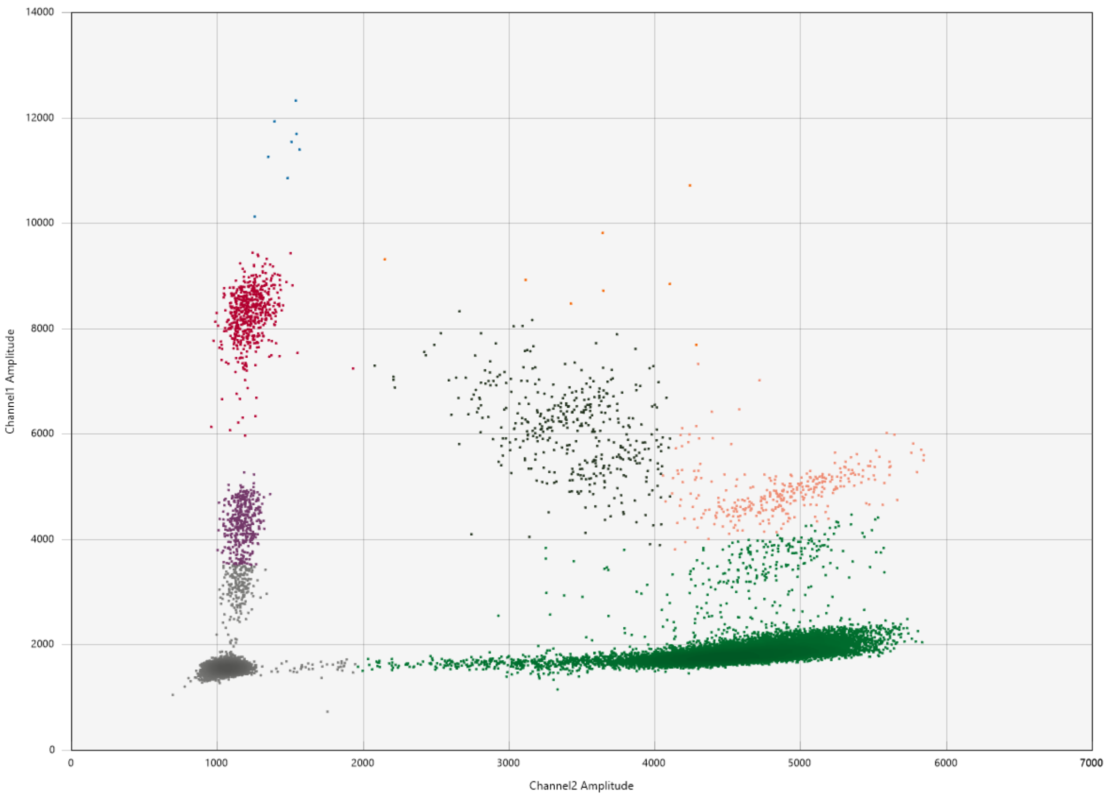

follow-up 1- 10 X dilution

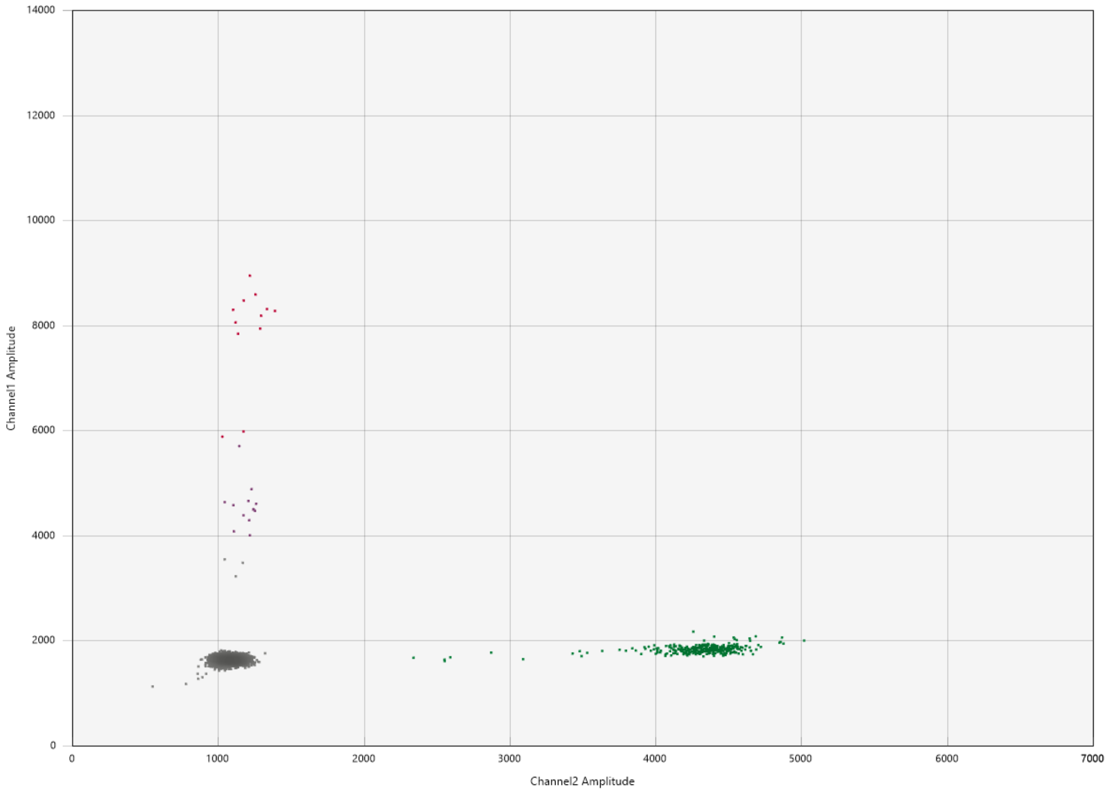

Concentration plots Pre ,Post-surgery and Follow-up1 Diluted-Pre ,Post-surgery and Follow-up1

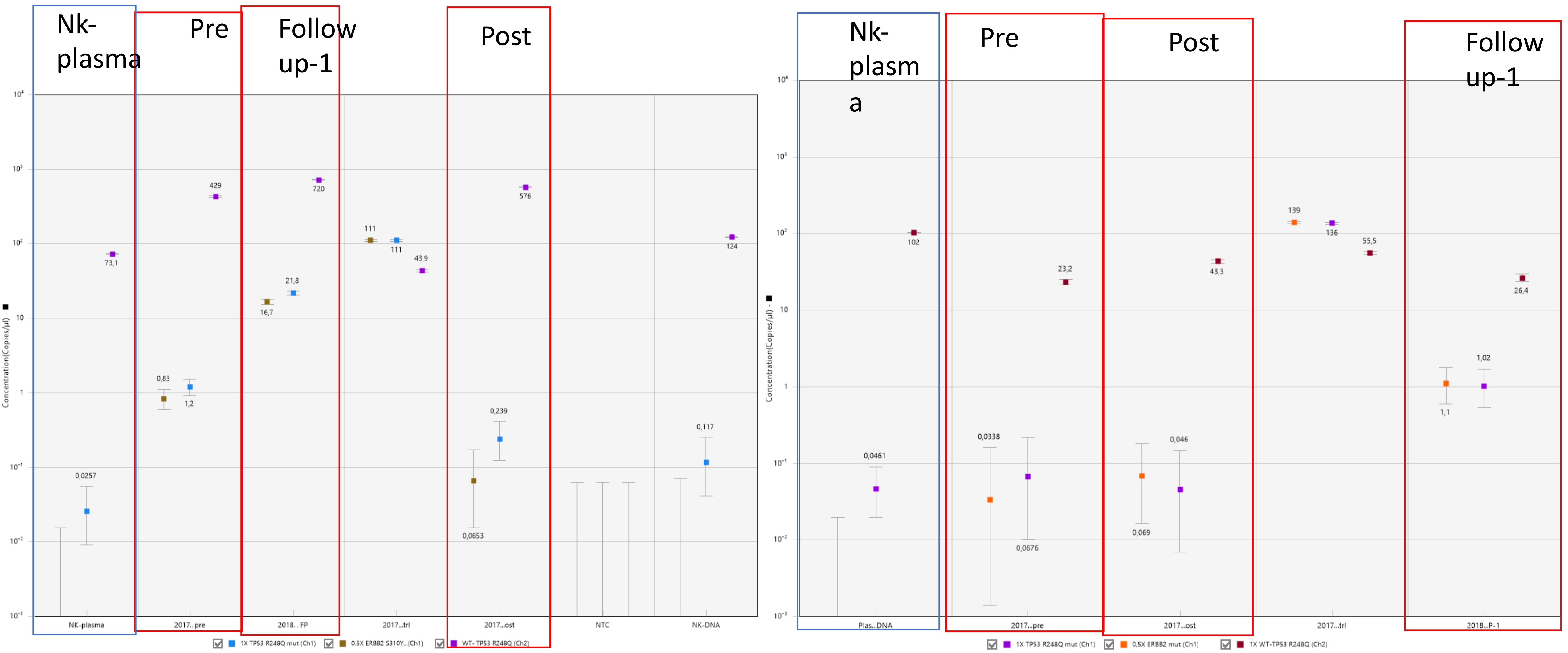

Supplement: Supplementary file 1 [file cancers-15-01160-s001.zip › Figure S6.pdf]
