# Supplementary material for: Sensitive Detection of Cell-Free Tumour DNA Using Optimised Targeted Sequencing Can Predict Prognosis in Gastro-Oesophageal Cancer
Source: Cancers (Basel). 2023 Feb 11;15(4):1160. doi: 10.3390/cancers15041160 (PMC9954085; doi:10.3390/cancers15041160)
Supplement: Supplementary file 1 [file cancers-15-01160-s001.zip › FigureS1.pdf]

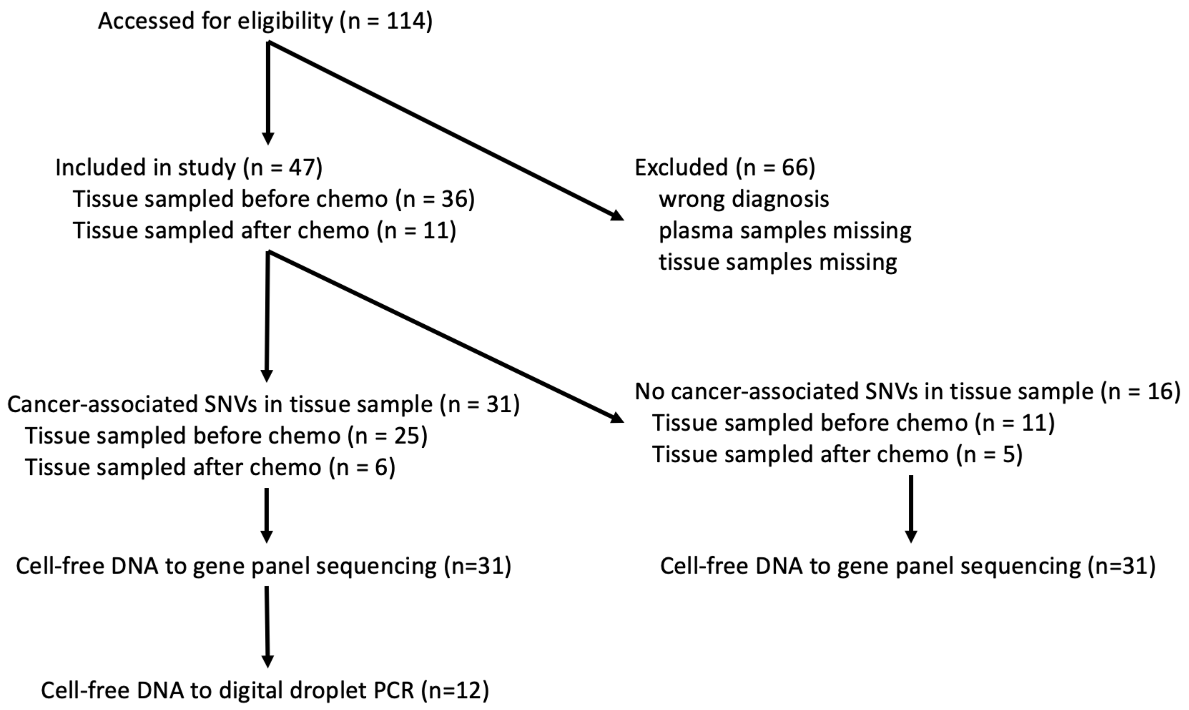

Figure S1 – REMARK diagram of patient inclusion

Out of the 47 participants, 31 had cancer-associated SNVs in tissue. Out of those, 12 were selected for additional ddPCR (all of those 12 had tissue sampled before chemotherapy).

chemo; chemotherapy

REMARK; REporting recommendations for tumour MARKer prognostic studies
