# Supplementary material for: Sensitive Detection of Cell-Free Tumour DNA Using Optimised Targeted Sequencing Can Predict Prognosis in Gastro-Oesophageal Cancer
Source: Cancers (Basel). 2023 Feb 11;15(4):1160. doi: 10.3390/cancers15041160 (PMC9954085; doi:10.3390/cancers15041160)
Supplement: Supplementary file 1 [file cancers-15-01160-s001.zip › FigureS2.pdf]

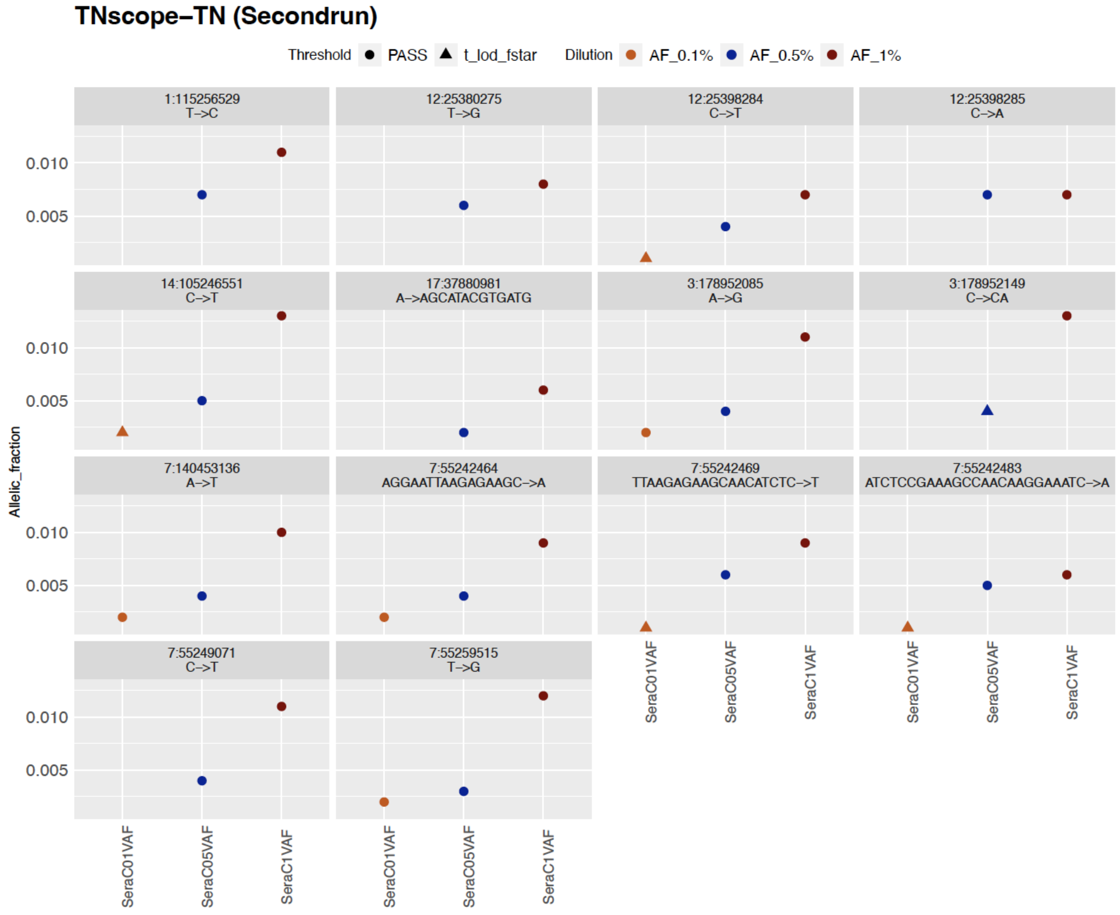

Figure S2 – Scatter plot of technical validation of the GIcfDNA gene panel on cell free DNA from reference samples. Scatterplot of detection of 14 reference variants with TNScope (Senteion) analysis of cfDNA sequencing aiming at 40M readpairs on SeraCare samples with VAF 0.1% (denoted SeraC01), 0.5% (denoted SeraC05) and 1% (SeraC1) run with plasma from blood donor as normal control. The default min_tumor_lod score was used. We could detect 4 variants at VAF 0.1%, 13 at VAF 0.5% and all 14 at 1% VAF.

VAF; variant allele frequency.
