# Supplementary material for: Sensitive Detection of Cell-Free Tumour DNA Using Optimised Targeted Sequencing Can Predict Prognosis in Gastro-Oesophageal Cancer
Source: Cancers (Basel). 2023 Feb 11;15(4):1160. doi: 10.3390/cancers15041160 (PMC9954085; doi:10.3390/cancers15041160)
Supplement: Supplementary file 1 [file cancers-15-01160-s001.zip › FigureS4.pdf]

A

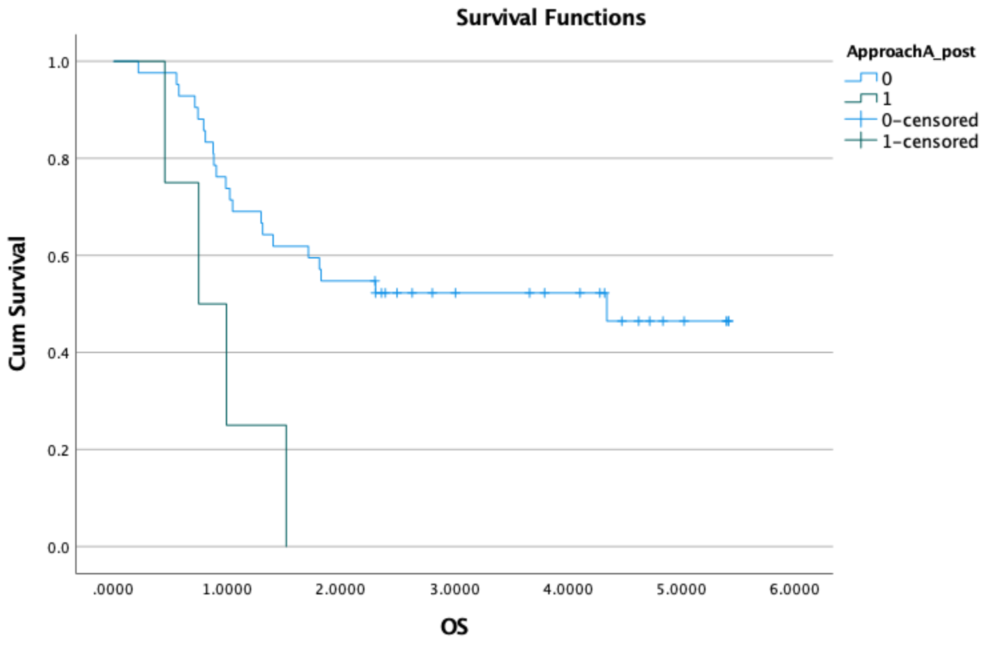

B

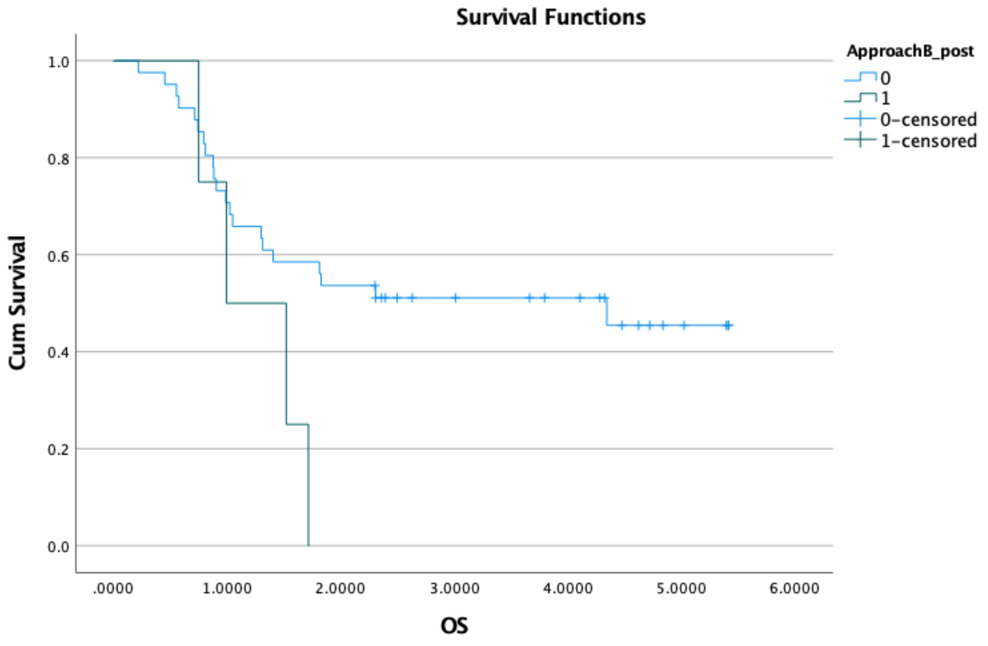

Figure S4
Kaplan-Meier curves of overall survival, in correlation to detectable ctDNA (1, green line) and no detectable ctDNA (0, blue line) in the plasma sampled after surgery (post-surgical).
(a) Kaplan-Meire curve of overall survival based on ctDNA detection using approach A (denominated according to Figure 2) for the post-surgical sample. Log rank p=0.05**.**
(b) Kaplan-Meire curve of overall survival based on ctDNA detection using approach B (denominated according to Figure 2) for the post-surgical sample. Log rank p=0.072**.**
