# Supplementary material for: Sensitive Detection of Cell-Free Tumour DNA Using Optimised Targeted Sequencing Can Predict Prognosis in Gastro-Oesophageal Cancer
Source: Cancers (Basel). 2023 Feb 11;15(4):1160. doi: 10.3390/cancers15041160 (PMC9954085; doi:10.3390/cancers15041160)
Supplement: Supplementary file 1 [file cancers-15-01160-s001.zip › FigureS5.pdf]

Figure S5 - Longitudinal graphs of ctDNA variants detected by ddPCR
ddPCR results for each participant, in relation to surgery and chemotherapy. The VAFs of the ctDNA variants are defined by the left axis. The hGe/ml is defined by the scale on the right Y axis. The scales differ between figures.
On the Y axis, levels of detected pathogenic variants targeted by the ddPCR assay, as well as the total amount of cell-free DNA (hGe/ml). The hGE/ml is defined by the scale on the right Y axis. On the X axis, time from diagnosis. For P44, the *KRAS* variant was determined negative since the error bars overlapped with the negative controls.
ctDNA; cell-free tumour DNA, ddPCR; digital droplet PCR, VAF; variant allele frequency.
Doc; Docetaxel, Oxa; Oxaliplatin, Flu; Fluorouracil, Iri; irinotecan, Flv; 5-fluorouracil/leucovorin.

P12

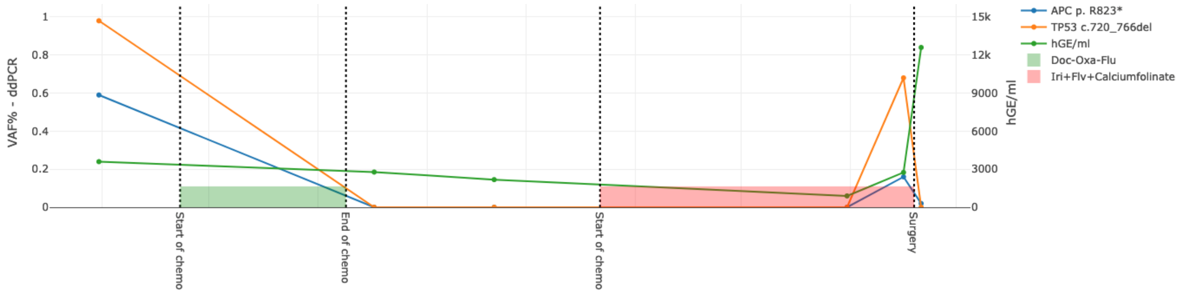

P15

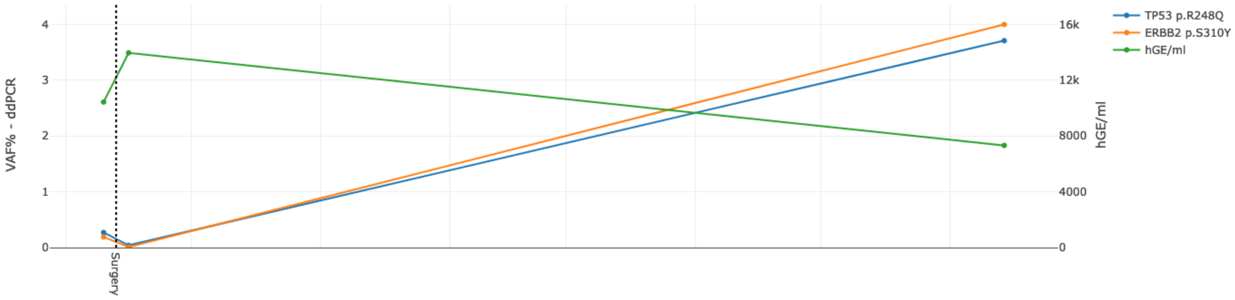

P23

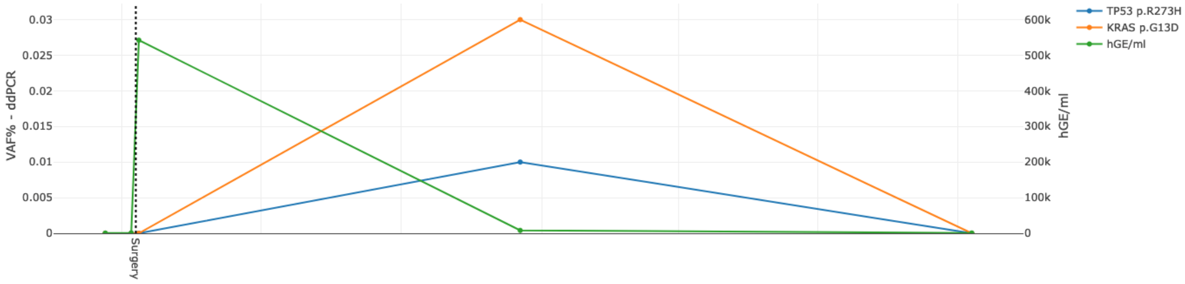

P25

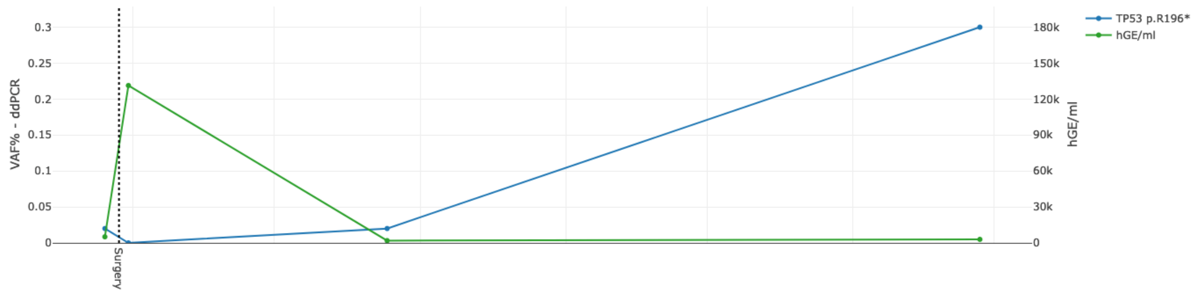

P27

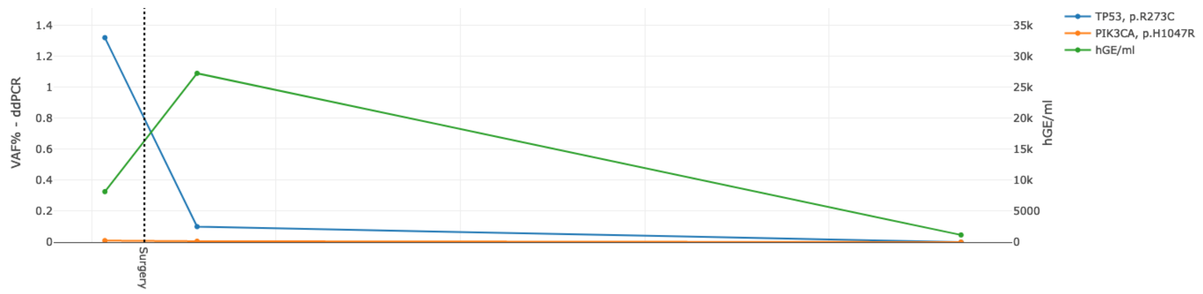

P28

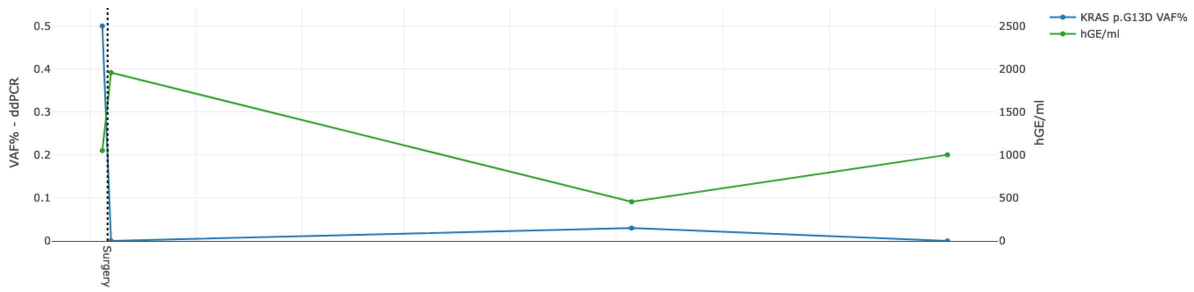

P32

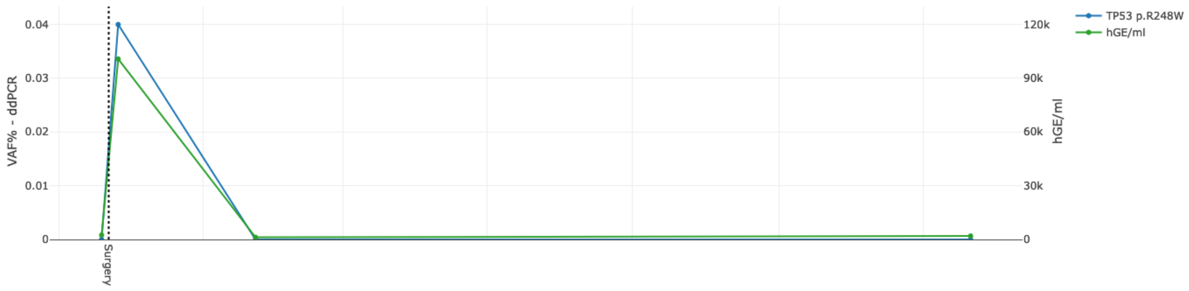

P38

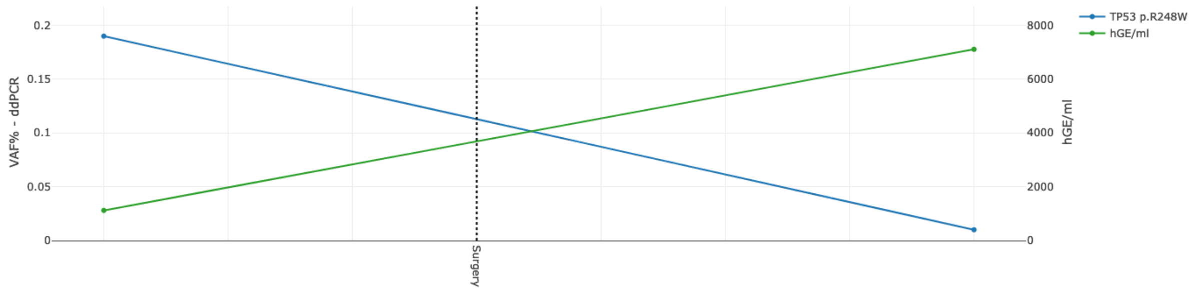

P41

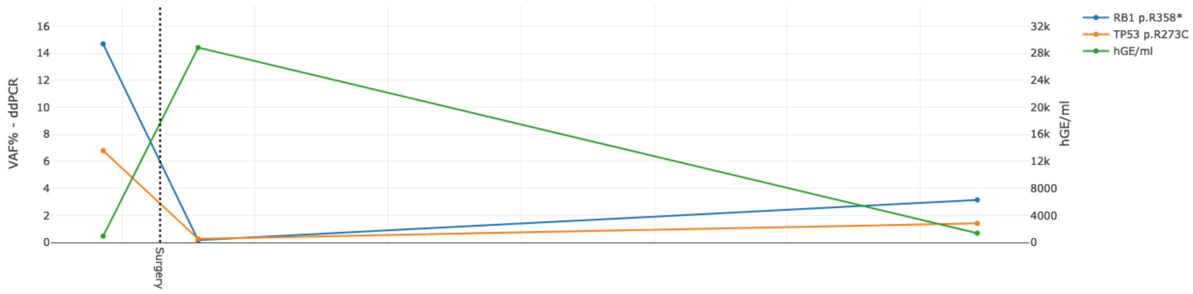

P42

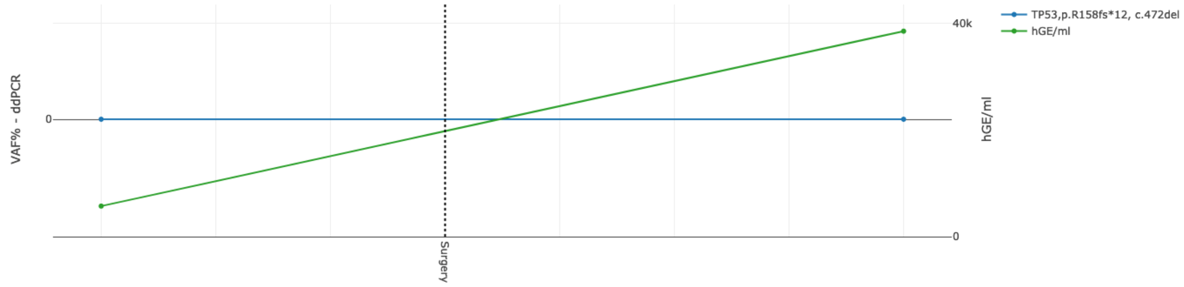

P43

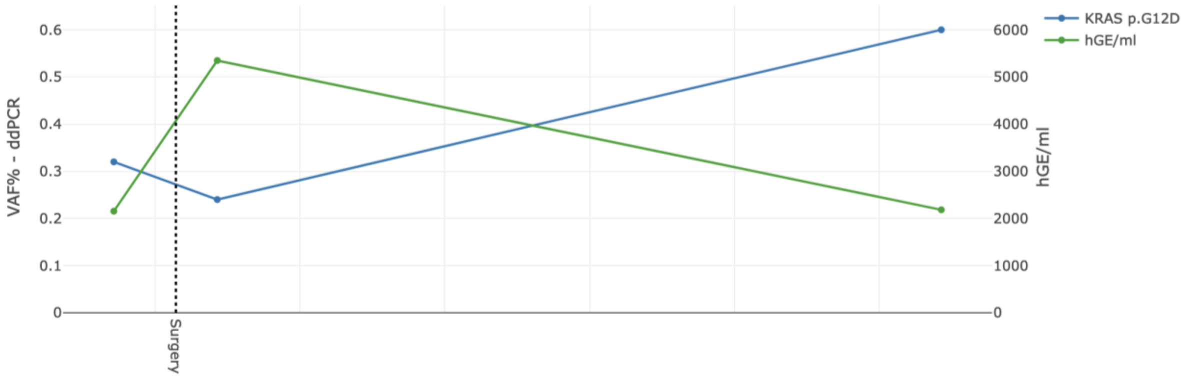

P44

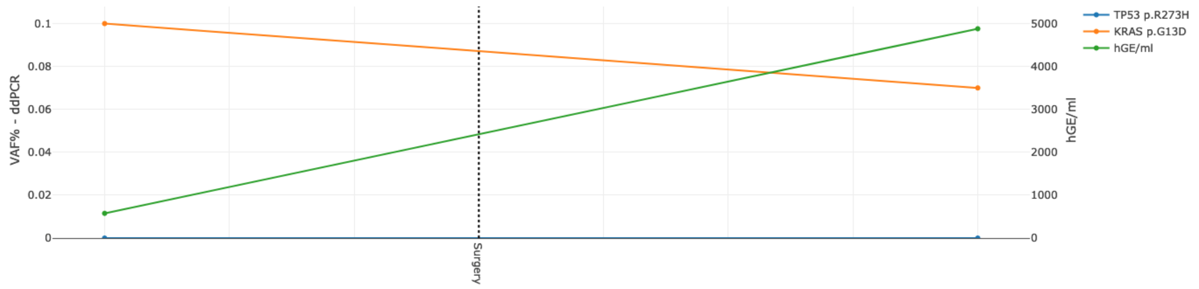

VAF% - ddPCR
0.1
0.08
0.06
0.04
0.02
0
hGE/ml
5000
4000
3000
2000
1000
0
Surgery
TP53 p.R273H
KRAS p.G13D
hGE/ml
