# Supplementary figures and images for: Sensitive Detection of Cell-Free Tumour DNA Using Optimised Targeted Sequencing Can Predict Prognosis in Gastro-Oesophageal Cancer
Source: Cancers (Basel). 2023 Feb 11;15(4):1160. doi: 10.3390/cancers15041160 (PMC9954085; doi:10.3390/cancers15041160)

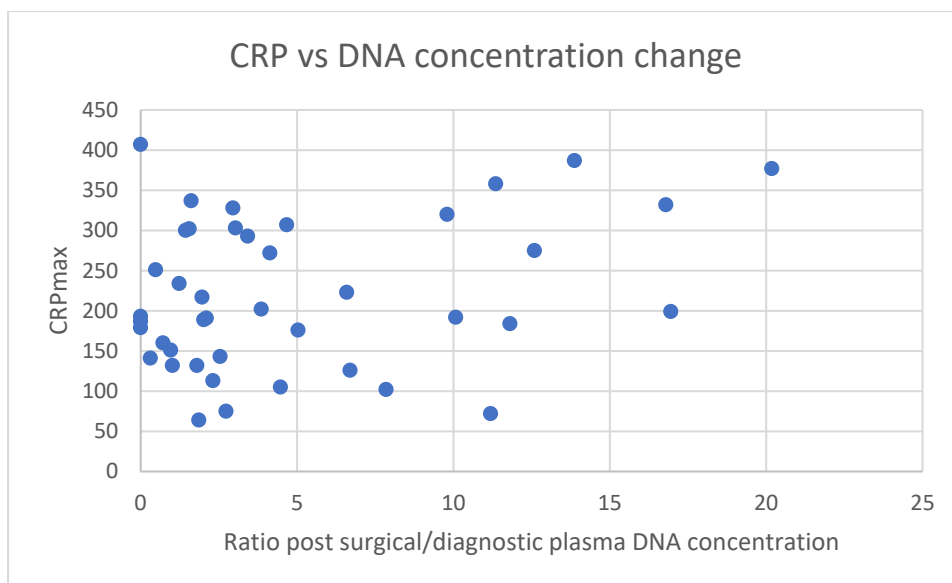

Supplement: Supplementary file 1 [file cancers-15-01160-s001.zip › FigureS3.pdf]
